# Unraveling the Potential of Vinyl Ether as an Ethylene Surrogate in Heteroarene C─H Functionalization via the Spin‐Center Shift

**DOI:** 10.1002/advs.202309800

**Published:** 2024-03-13

**Authors:** Wonjun Choi, Leejae Kim, Sungwoo Hong

**Affiliations:** ^1^ Department of Chemistry Korea Advanced Institute of Science and Technology (KAIST) Daejeon 34141 Republic of Korea; ^2^ Center for Catalytic Hydrocarbon Functionalizations Institute for Basic Science (IBS) Daejeon 34141 Republic of Korea

**Keywords:** C─H vinylation, ethylene surrogate, spin‐center shift (SCS), vinyl ether, *α*‐oxy radical

## Abstract

Despite the simplicity and abundance of ethylene, its practical application presents significant hurdles due to its nature as a highly flammable gas. Herein, a strategic use of easily handled vinyl ether is reported as a latent ethylene surrogate achieved via a spin‐center shift (SCS) pathway, enabling the successful three‐component reaction that bridges heteroarenes and various coupling partners, including sulfinates, thiols, and phosphine oxides. Through a photoredox catalytic process, *α*‐oxy radicals are generated by combining various radicals with phenyl vinyl ether, which are subsequently added to N‐heteroarenes. Subsequently, the radical‐mediated SCS pathway serves as the driving force for C─O bond cleavage, effectively engaging the phenoxy group as a leaving group. In addition, by broadening the utility of the method, a valuable synthon is provided for efficient C─H vinylation of N‐heteroarenes following sulfonyl group elimination. This approach not only enriches the toolbox of synthetic methodology but also provides a more streamlined alternative, circumventing the challenges associated with direct ethylene gas usage. The versatility of the method, particularly evident in late‐stage functionalizations of medicinally relevant molecules and peptides, underscores its capability to produce invaluable three‐component compounds and vinylated N‐heteroarene derivatives.

## Graphical abstract

Through a spin‐center shift pathway, a strategic approach using vinyl ether as a surrogate for ethylene has been established, connecting heteroarenes with various coupling partners like sulfinates, thiols, and phosphine oxides. This method also provides vinylated *N*‐heteroarene via direct C–H vinylation, particularly in a transition‐metal‐free condition.

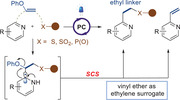

## 1 Introduction

**Scheme 1:**
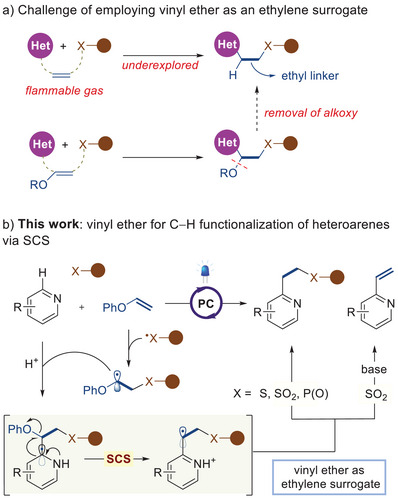
Strategy for employing vinyl ethers as ethylene surrogates via SCS.

In fields such as medicinal and synthetic chemistry, linkers serve as essential bridges between two molecular entities, allowing for the fine‐tuning of molecular properties.^[^ ^1^ ^]^ This critical role has spurred increasing efforts to devise efficient and selective methods for connecting two molecular units without making significant alterations to their basic structures. While the ethyl moiety (─CH_2_CH_2_─) may appear to be an intuitive choice as a straightforward and ideal linker, synthetic methods for appending two molecules to it remain limited. The 1,2‐difunctionalization of ethylene offers an intriguing strategy,^[^ ^2^ ^]^ yet it comes with inherent challenges, especially in complex molecular systems where optimal selectivity and reactivity are imperative (**Scheme** **1**a).^[^ ^3^ ^]^ Recently, Wu and Zhu successfully executed a three‐component 1,2‐difunctionalization of ethylene using sulfone‐based bifunctional reagents through a radical‐mediated functional group migration.^[^ ^4^ ^]^ However, despite the simplicity and abundance of ethylene, its practical implementation as a reagent comes with significant hurdles. As a highly flammable gas, ethylene demands specialized storage solutions and stringent safety measures. Furthermore, its high reactivity in radical approaches often triggers unwanted polymerization and oligomerization, further complicating its practical application.^[^ ^5^ ^]^ Therefore, the pursuit of an appropriate alkene source as a viable alternative to ethylene gas in organic reactions is pressing, yet continues to be a challenge. For example, the potential for using easily handled vinyl ethers as ethylene substitutes remains largely untapped, primarily due to the difficulty involved in removing the alkoxy group.

The spin‐center shift (SCS) mechanism, instrumental in facilitating radical relocation and hastening the removal of adjacent leaving groups, underpins a myriad of biological transformations and synthetic reactions.^[^ ^6^ ^]^ This process becomes particularly interesting when it involves the cleavage of diverse carbon‐to‐heteroatom bonds.^[^ ^7^ ^]^ By capitalizing on the fundamental principles of the SCS mechanism and the Minisci reaction, the MacMillan group has pioneered an innovative approach to the C─H alkylation reaction of *N*‐heteroarenes, marked by the use of *α*‐oxy radicals as alkyl radical precursors.^[^ ^8^ ^]^ While the two‐component radical cross‐coupling involving the SCS pathway has advanced considerably, for selected examples, see ref. [9], a synthetic method integrating a three‐component reaction with the SCS strategy,^[^ ^10^ ^]^ to the best of our knowledge, has remained unexplored. Considering the distinctive reactivities of the SCS pathway involving *α*‐oxy radicals, we speculated on the potential of easily handled vinyl ethers as ethylene surrogates, thus bridging two components with an ethyl linkage. Building upon the transformative potential of photoredox single electron transfer (SET) catalysis, we postulated the combination of various radicals with vinyl ethers to generate *α*‐oxy radicals upon visible light irradiation, which could subsequently be introduced to *N*‐heteroarenes (Scheme 1b). Importantly, by employing the SCS process to engage the phenoxy group as a departing entity, this approach would offer unique opportunities to an array of complex three‐component assemblies, with phenyl vinyl ether standing in as a practical substitute for ethylene gas.

On a parallel note, the installation of the vinyl group (─CH═CH_2_) into *N*‐heteroarenes enriches their utility as synthetic building blocks.^[^ ^11^ ^]^ Once incorporated, the vinyl moiety can be further modified into various substituents or reactive entities, thus dramatically influencing the properties of the resulting derivatives, for recent examples, see ref. [12, 13] Numerous synthetic methods exist for vinylation; however, most necessitate multistep preparations of pre‐functionalized reagents and largely depend on transition‐metal‐catalyzed reductive cross‐coupling with aryl halides, for selected examples, see ref. [14, 15] Recently, pyridine alkenylation has been accomplished by coupling pyridine with alkenes, sulfinate, and cyano–pyridine.^[^ ^16^ ^]^ Nonetheless, styrene remains the only viable choice due to the requirement for a stable radical intermediate, rendering the incorporation of a vinyl group unattainable. Given this backdrop, efficiently incorporating the vinyl group into *N*‐heteroarenes, especially with easily available reagents under transition‐metal‐free settings, remains a pivotal synthetic undertaking. Therefore, we pursued expanding our method to provide a valuable synthon for the adept C─H vinylation of *N*‐heteroarenes, following the removal of sulfonyl groups. Our unified methodology streamlines the direct incorporation of both a bridging linker and the vinyl group into *N*‐heteroarenes, highlighting the practicality and extensive versatility.

## 2 Results and Discussion

We initiated our study by assessing the feasibility of a three‐component SCS process, using isoquinoline (**1a**), phenyl vinyl ether (**2a**), and sodium 4‐chlorobenzenesulfinate (NaSO_2_(*p*‐ClPh)) as model substrates (**Table** **1**). Our comprehensive exploration culminated in the efficient formation of the three‐component product **3a** with a yield of 81%, by employing 1,3‐dicyano‐2,4,5,6‐tetrakis(diphenylamino)‐benzene (4DPAIPN) as the organic photocatalyst,^[^ ^17^ ^]^ H_2_SO_4_ as the acid, and degassed DMSO as the solvent under blue LED irradiation (entry 1). While alkyl vinyl ether (**2b**) was ineffective, vinyl esters (**2c** and **2d**) exhibited modest activity (≈20%), likely due to their propensity for C─O bond cleavage via effective leaving groups. We also tested the potential for C─N bond cleavage under the same reaction conditions (**2e**‐**2h**),^[^ ^18^ ^]^ but were not successful in generating the desired product, which can be attributed to the inherent resilience of C─N bonds. When we explored the cleavage of other bond types, only the C─S bond cleavage was found feasible (**2j**), while C─P (**2k**) and C─CN (**2l**) bonds remained intact. Upon screening various photocatalysts, we found that 3DPA2FBN (**PC1**), 4CzIPN (**PC2**), and [Ir(dF(CF_3_)ppy)_2_dtbbpy]PF_6_ (**PC3**) yielded similar results to 4DPAIPN (entry 2). However, Mes‐Acr‐MeClO_4_ (**PC4**), which is known for its high oxidation potential, was unsuccessful in producing the desired product, possibly due to its low reducing ability (entry 3).^[^ ^19^ ^]^ Replacing the H_2_SO_4_ with TFA and TsOH·H_2_O resulted in slightly lower yields (entry 4). Lewis acid‐mediated reactions were non‐productive (entry 5). We discovered that degassing the solvent was a crucial step, as residual O_2_ in the solvent could act as an oxidant during the reaction, impeding the SCS step (entry 6). Additional experiments were conducted under various conditions, including in an O_2_ atmosphere and open air, to yield the adduct that retains the PhO group (see Supporting Information). Diluting the reaction mixture led to reduced yields (entry 7). To enhance the reaction yield, we next experimented with various alternative solvents. DCE gave the trace amount of the desired product and using MeCN resulted in no yield (entry 8), while DMA produced a moderate yield (entry 9). Control experiments affirmed the indispensability of the photocatalyst, light source, and acid for the reaction's success (entry 10).

**Table 1:** Optimization of the reaction conditions for a three‐component reaction via SCS.^a^ ^)^

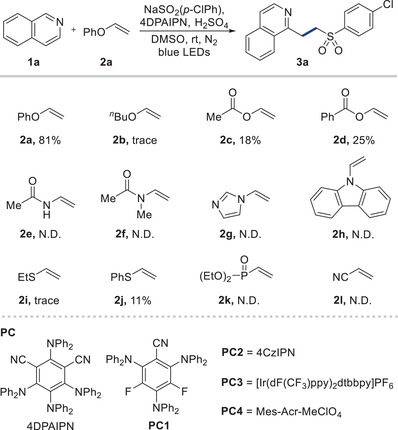		
Entry	Deviation from the standard conditions	yield 3a (%)^b^ ^)^
1	No change	81 (81)^c^ ^)^
2	PC1, PC2, PC3 instead of 4DPAIPN	68/67/74
3	PC4 instead of 4DPAIPN	N.D.
4	TFA, TsOH·H2O instead of H2SO4	63/64
5^d^ ^)^	Sc(OTf)3 instead of H2SO4	N.D.
6	non‐degassed DMSO	72
7	0.1 m instead of 0.3 m	68
8	DCE, MeCN instead of DMSO	trace/N.D.
9	DMA instead of DMSO	73
10	w/o photocatalyst, light, acid	N.D.

^a^ ^)^Reaction conditions: **1a** (0.1 mmol), **2a** (2.0 equiv), NaSO_2_(*p*‐ClPh) (1.5 equiv), 4DPAIPN (1.0 mol%), H_2_SO_4_ (2.0 equiv), and degassed DMSO (0.3 m) was irradiated under blue LEDs at rt for 16 h in a capped test tube under N_2_ atmosphere;

^b^ ^)^Yields were determined by ^1^H NMR spectroscopy;

^c^ ^)^ Isolated yield;

^d^ ^)^ Sc(OTf)_3_ (0.1 equiv) was used. N.D. = Not detected.

With optimized reaction conditions in hand, we proceeded to test the versatility of the three‐component SCS reaction using various coupling partners, as depicted in **Table** **2**. We found that non‐substituted (**3b**), 4‐methyl (**3c**), electron‐withdrawing trifluoromethyl (**3d**), and electron‐donating methoxy (**3e**) phenyl sulfinate provided the desired products. Furthermore, 2‐thiophene sulfinate (**3f**) was amenable to the reaction. We also explored alkyl sulfinate and discovered that ethyl (**3g**) and cyclopropyl (**3h**) sulfinates could generate the desired products. The reaction's scope was significantly expanded to include a range of heteroarenes. This includes phenanthridine (**3i**), and notably, phthalazine (**3j**), characterized by its two nitrogen atoms in the ring, which was efficiently processed using this method. The method also proved effective with benzothiazole (**3k**), featuring a five‐membered ring and similarly showed success in modifying pyridine (**3l**). The methodology was not limited to sulfonyl compounds but extended to other coupling entities. The incorporation of phosphine into molecules can markedly modify their chemical behavior, considering phosphine as a potent tool in both synthetic and medicinal chemistry, For selected examples, see ref. [20]. Pleasingly, we found that a range of substituted phosphine oxides and heteroarenes could be utilized to yield the desired three‐component products (**3m**‐**3r**). Thiol is a key component commonly found in various biologically active molecules, natural products, and functional materials.^[^ ^21^ ^]^ Hence, an efficient method for integrating thiols into *N*‐heteroarenes has significant implications in both medicinal and synthetic chemistry. Therefore, we conducted a three‐component reaction with thiol and discovered that our methodology enabled the synthesis of sulfides from primary (**3s**), secondary (**3t**), and tertiary (**3u**) thiols. Notably, even cysteine could be employed as a substrate, leading to the formation of the desired product **3v**. This reaction also yielded the thiolated products of quinoline (**3w**) and phenanthroline (**3x**). To showcase the broad applicability of our approach in a more complex setup, we performed late‐stage conjugation of medicinally relevant molecules. Intriguingly, a fasudil derivative exhibited efficient reactivity, leading to the formation of the sulfonylation (**3y**), phosphionylation (**3z**), and thiolation (**3aa**) products. Likewise, cinchonine readily engaged in the reaction under standard reaction conditions, resulting in the sulfonylated desired product **3ab**. Encouraged by these results, we further investigated peptide substrates composed of cysteine and other indigenous amino acids. Peptides featuring serine with its alcohol side chain (**3ac**), and aspartic acid bearing a carboxylic acid side chain (**3ad**), were successfully converted into the desired thiolation products.

**Table 2:** Substrate the scope of the three‐component reaction.^a^ ^)^

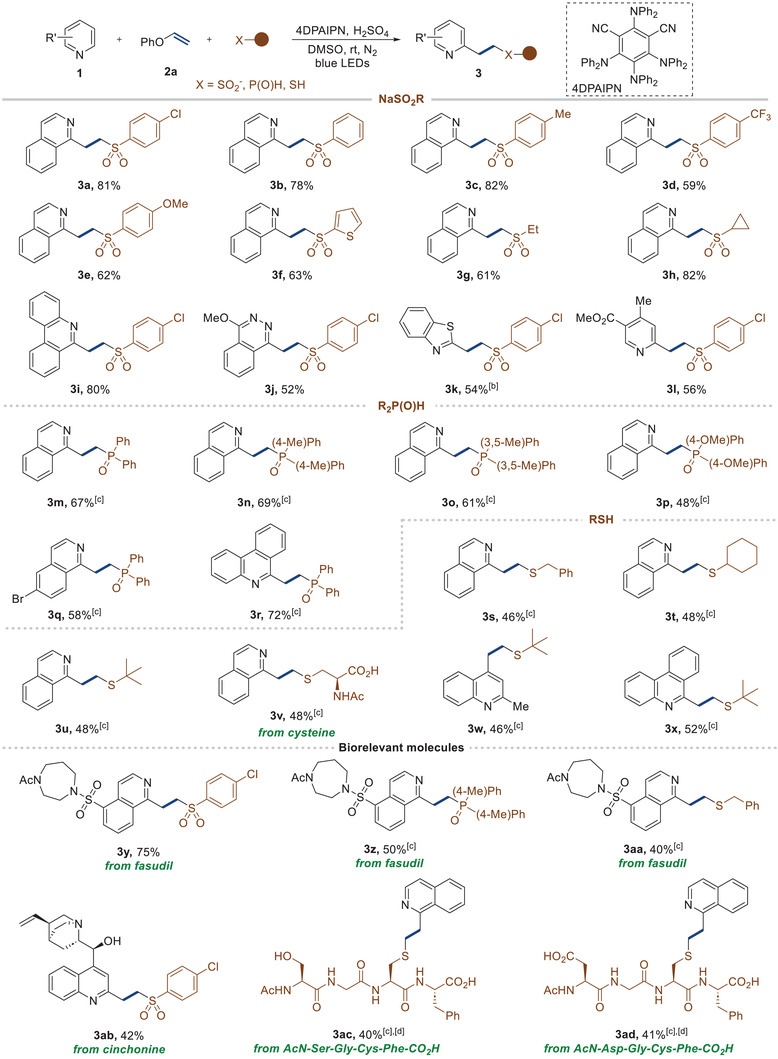

^a^ ^)^Reaction conditions: **1** (0.1 mmol), **2a** (2.0 equiv), NaSO_2_R, or R_2_P(O)H, or RSH (1.5 equiv), 4DPAIPN (1.0 mol %), H_2_SO_4_ (2.0 equiv), and degassed DMSO (0.3 m) was irradiated under blue LEDs at rt for 16 h in a capped test tube under a N_2_ atmosphere;

^b^ ^)^3DPA2FBN was used instead of 4DPAIPN;

^c^ ^)^ TFA was used instead of H_2_SO_4_;

^d^ ^)^ Reactions were performed by using **1** (0.05 mmol) in degassed DMSO (0.1 m).

While previous studies have demonstrated the generation of vinyl *N*‐heteroarenes using pre‐functionalized substrates in conjunction with transition metal catalysts, the direct C─H vinylation of these compounds using vinyl ether, especially in a metal‐free context, remains a considerable challenge. In our approach, we speculated the potential of the resulting sulfone moiety as an effective leaving group, presenting a unique opportunity to produce vinylated *N*‐heteroarenes in a one‐pot process. To explore the possibility of accessing vinyl *N*‐heteroarenes via C─H vinylation, we initiated the elimination reaction with **3a** and K_3_PO_4_ as the base, resulting in the successful conversion of **3a** into the vinylated product **4a** (see the Supporting Information). These encouraging results prompted us to further investigate the feasibility of a one‐pot C─H vinylation of *N*‐heteroarenes. To achieve this goal, we carried out further investigation of various bases (Table **3**). Pleasingly, we succeeded in obtaining the desired vinylated product **4a** with a yield of 75% in a one‐pot process (entry 1). It's worth noting that aryl sulfinates with electron deficiencies exhibited enhanced leaving group capabilities during elimination steps, while the more electron‐rich 4‐methylbenzenesulfonyl group resulted in a reduced yield of product **4a** (entry 2). Of the bases tested, potassium phosphate tribasic emerged as the most effective for the reaction (entries 3–5). The reaction proceeded slowly when the mixture's concentration diminished and the operating temperature was decreased (entries 6 and 7).

**Table 3:** Optimization of one‐pot vinylation.^a^ ^)^

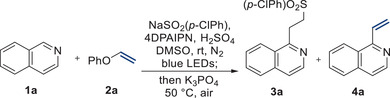		
Entry	Deviation from the standard conditions	yield 3a/4a (%)^b)^
1	No change	trace/77 (76)^c)^
2	NaSO2Tol instead of NaSO2(p‐ClPh)	55/17
3	KOtBu instead of K3PO4	trace/48
4	KOTMS instead of K3PO4	trace/70
5	DBU instead of K3PO4	trace/60
6	0.1 m instead of 0.3 m	30/41
7	30 °C instead of 50 °C	42/23

^a^ ^)^Reaction conditions: **1a** (0.1 mmol), **2a** (2.0 equiv), NaSO_2_(*p*‐ClPh) (1.5 equiv), 4DPAIPN (1.0 mol %), H_2_SO_4_ (2.0 equiv), and degassed DMSO (0.3 m) was irradiated under blue LEDs at rt for 16 h in a capped test tube under N_2_ atmosphere, then, K_3_PO_4_ (5.0 equiv) was added, and the reaction mixture was stirred at 50 °C for 6 h;

^b^ ^)^Yields are determined by ^1^H NMR spectroscopy;

^c^ ^)^Isolated yield.

After optimizing the reaction conditions, we further explored the generality of the vinylation reaction, as depicted in **Table** **4**. We began by testing isoquinoline derivatives with various substituents at the C3 position, including methyl (**4b**), phenyl (**4c**), and methyl ester (**4d**), which provided the desired products. We also successfully employed isoquinoline derivatives with substitutions at the C6 position, such as methyl (**4e**), nitrile (**4f**), and methoxy (**4g**) groups showcasing robust tolerance regardless of their electronic characteristics. Notably, the vinylation reaction showed remarkable compatibility with halides including chloride and bromide positioned at different locations: C4 (**4h**), C5 (**4i** and **4j**), C6 (**4k**), and C7 (**4l**). This compatibility offers opportunities for further functionalization such as cross‐coupling reactions. We then turned our attention to the vinylation of quinoline substrates. The reactions involving alkyl (**4m** and **4n**), phenyl (**4o**), fluoride (**4p**), and methoxy (**4q**) groups resulted in the successful formation of vinylated products. Notably, the scope of the reaction was broadened to include tricyclic rings such as acridine (**4r**) and phenanthroline (**4s**), which hold significant importance in medicinal chemistry due to their potential as emerging anticancer drug candidates, attributed to their DNA binding affinity.^[^ ^22^ ^]^ Additionally, both phthalazine (**4t**) and benzothiazole (**4u**) are efficiently processed using this method. Furthermore, pyridines were adeptly converted to the desired products using this methodology (**4v**–**4x**). Pleasingly, our method allowed for the late‐stage modification of a medically pertinent molecule, fasudil, yielding the target product **4y**.

**Table 4:** Substrate the scope of the vinylation reaction^a^ ^)^ and transformation of vinyl *N*‐heteroarenes.

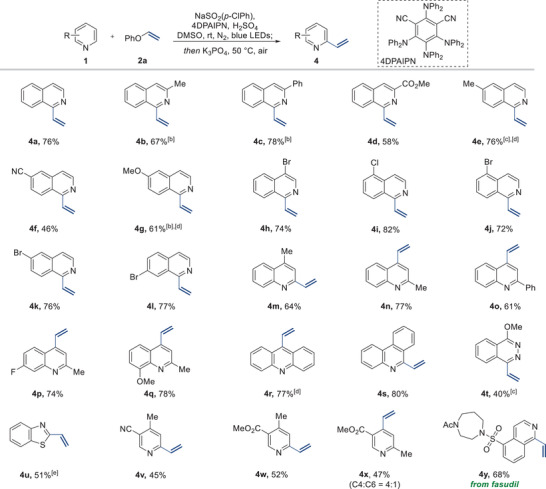

^a^ ^)^Reaction conditions: **1** (0.1 mmol), **2a** (2.0 equiv), NaSO_2_(*p*‐ClPh) (1.5 equiv), 4DPAIPN (1.0 mol%), H_2_SO_4_ (2.0 equiv), and degassed DMSO (0.3 m) was irradiated under blue LEDs at rt for 16 h in a capped test tube under N_2_ atmosphere, then K_3_PO_4_ (5.0 equiv) was added, and the reaction mixture was stirred at 50 °C for 6 h;

^b^ ^)^Stirred for 12 h after K_3_PO_4_ (5.0 equiv) was added;

^c^ ^)^Stirred for 24 h after K_3_PO_4_ (5.0 equiv) was added;

^d^ ^)^ **2a** (3.0 equiv) and NaSO_2_(*p*‐ClPh) (2.25 equiv) were used;

^e^ ^)^3DPA2FBN was used instead of 4DPAIPN. The regioisomeric ratio was determined by ^1^H NMR.

To demonstrate the utility of vinylation, we investigated the potential for subsequent transformation of the vinyl building block on the heteroarene (Table 4, down). Achieving this would make the vinylation strategy an attractive method for accessing a broad array of valuable materials. Initially, we assessed the feasibility of a multiscale (5.0 mmol) reaction under slightly modified conditions, which provided a comparable result. Subsequently, we explored [3+2] cycloaddition reactions as a means of introducing new rings. By conducting a reaction of the vinylated compounds with oxime, we successfully formed an isoxazoline ring (**5a**). A similar approach, engaging the compounds in a reaction with isocyanide led to the formation of a pyrrole ring (**5b**). We ventured into [4+2] cycloaddition reactions, which resulted in the formation of a bridged ring with an outstanding yield (**5c**). A Lewis acid‐mediated nucleophilic addition was also effective, resulting in a hydroamination product **5d**. Considering the distinct structure of the strained small rings, we successfully synthesized azetidine (**5e**) and epoxide (**5f**) derivatives, exemplifying their potential applications. Cumulatively, these outcomes underline the considerable synthetic potential of the vinyl moiety in heteroarene derivatization.

**Scheme 2:**
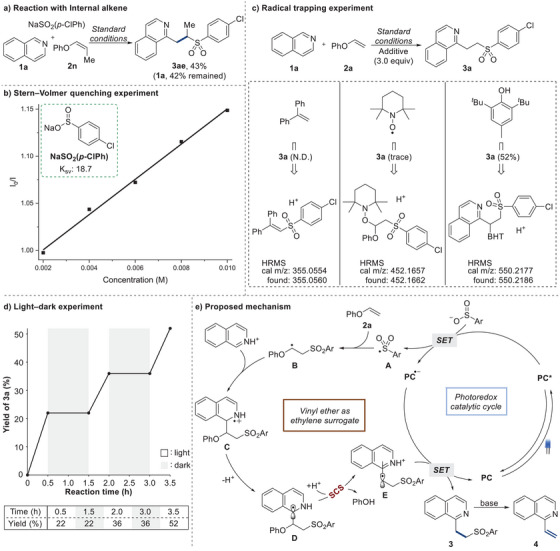
Control experiments and proposed mechanism.


To gain a mechanistic understanding of the reaction, we conducted a series of control experiments. We carried out the reaction with an internal alkenyl ether featuring a methyl group under standard conditions. This yielded a modest amount of product **3ae** (43%) and left 42% of the starting material **1a** unreacted. These results imply that reactions involving internal alkenes require further optimization (**Scheme** **2**a). Next, we initiated a Stern–Volmer luminescence quenching experiment, which revealed that only NaSO_2_(*p*‐ClPh) underwent effective quenching, which directly correlated with its concentration upon interaction with 4DPAIPN (Scheme 2b). The Stern–Volmer quenching constant (K_sv_) for NaSO_2_(*p*‐ClPh) was found to be 18.7. On the other hand, no quenching was observed for other substrates, such as phenyl vinyl ether (**2a**), isoquinoline (**1a**), and its protonated form (**1a** + H^+^) (See the Supporting Information). In an effort to capture the radical intermediate, we conducted the reactions using 1,1‐diphenylethylene, TEMPO, and BHT. All resulted in the inhibition or decrease of the desired product formation. HRMS analysis showed the direct capture of a product from the sulfonyl radical and 1,1‐diphenylethylene. A TEMPO adduct, which formed from the combination of the sulfinate radical and phenyl vinyl ether, was detected. Furthermore, the validity of the SCS pathway was confirmed by capturing the benzylic radical using BHT. These radical trapping experiments support the presence of the sulfonyl radical, a radical adduct with phenyl vinyl ether and benzylic radical (Scheme 2c). To confirm the phenol elimination step, we conducted the reaction using vinyl ether **2m**, which can readily generate isolable phenol as a byproduct. Under these conditions, we obtained the desired product with a similar yield to the optimized condition, alongside 4‐phenyl phenol (**6**) (See the Supporting Information). The observation confirmed that C–O bond cleavage readily occurs to remove the phenoxy group during the reaction. Additionally, the light‐dark experiment showed that the desired three‐component product was not formed during the dark period. The measured quantum yield (Φ = 0.13) of the model reaction indicated that the radical chain process might not play a significant role in this reaction (Scheme 2d).

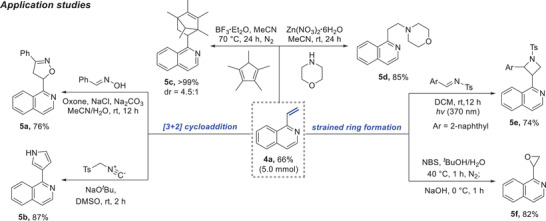


Based on the results obtained from the experiments, we propose a plausible mechanism for the three‐component reaction and vinylation (Scheme 2e). Upon exposure to visible light, the process initiates with NaSO_2_(*p*‐ClPh) undergoing single‐electron oxidation when interacting with the excited state of PC* (*E*_1/2_ [4DPAIPN*/4DPAIPN^−^] = +1.10 V vs SCE),^[^ ^23^ ^]^ leading to the formation of a sulfonyl radical **A** (PhSO_2_Na, *E*_1/2_ = +0.37 V vs SCE).^[^ ^24^ ^]^ This radical then engages in a radical addition with phenyl vinyl ether (**2a**), generating an *α*‐oxy radical intermediate **B**. This intermediate is subsequently captured by the protonated *N*‐hetereoarene, resulting in an aminyl radical cation **C**. Given its significant acidity, the α‐C─H bond of this aminyl radical cation undergoes deprotonation, transforming into an *α*‐amino radical **D**. Subsequently, C─O bond cleavage is facilitated by an SCS, yielding a benzylic radical **E**. Once the benzylic radical is reduced regenerating 4DPAIPN (E_1/2_ [4DPAIPN^−^/4DPAIPN] = −1.52 V vs SCE),^[^ ^22^ ^]^ protonation of the ensuing intermediate gives rise to three‐component product **3**. With the addition of a base, the vinylation product **4** is obtained in a streamlined, one‐pot process.

## 3 Conclusion

In summary, we have developed a three‐component reaction that seamlessly bridges heteroarenes and various coupling partners (sulfinates, thiols, and phosphine oxides) using easily‐managed vinyl ether as a latent ethylene surrogate. This method hinges on the radical‐mediated SCS pathway that drives the C─O bond cleavage, effectively leveraging the alkoxy group as a leaving entity. Moreover, our method offers a practical solution for efficient C─H vinylation of *N*‐heteroarenes after sulfonyl group elimination in a one‐pot process. Characterized by its mild reaction conditions, our approach broadens the spectrum of attainable three‐component products and vinylated *N*‐heteroarene derivatives. Overall, our strategy exhibits both practicality and versatility, alleviating the complexities intrinsic to the direct employment of ethylene gas, thereby substantially streamlining the synthesis process.

## Conflict of Interest

The authors declare no conflict of interest.

## Supporting information

Supporting Information

## Data Availability

The data that support the findings of this study are available in the supplementary material of this article.
