# Supplementary material for: Unraveling the Potential of Vinyl Ether as an Ethylene Surrogate in Heteroarene C─H Functionalization via the Spin‐Center Shift
Source: Adv Sci (Weinh). 2024 Mar 13;11(19):2309800. doi: 10.1002/advs.202309800 (PMC11109664; doi:10.1002/advs.202309800)

ADVANCED SCIENCE
Open Access

Supporting Information

for *Adv. Sci.*, DOI 10.1002/advs.202309800

Unraveling the Potential of Vinyl Ether as an Ethylene Surrogate in Heteroarene C—H Functionalization via the Spin-Center Shift

*Wonjun Choi, Leejae Kim and Sungwoo Hong**

Supplementary Information

Vinyl Ether as an Ethylene Surrogate in Heteroarene C–H Functionalization via the Spin-Center Shift

*Wonjun Choi, Leejae Kim, and Sungwoo Hong**

Department of Chemistry, Korea Advanced Institute of Science and Technology (KAIST), and Center for Catalytic Hydrocarbon Functionalizations, Institute for Basic Science (IBS), Daejeon 34141, Korea

I. General Information S2

II. Experimental Procedures S3

III. Control experiments S7

IV. Reference S15

V. Compound Characterizations S16

Appendix I

Spectral Copies of ^{1}H-, ^{13}C- and ^{19}F-NMR Data Obtained in this Study S41

I. General Information

Unless stated otherwise, reactions were performed in oven-dried glassware. Analytical thin layer chromatography (TLC) was performed on precoated silica gel 60 F^{254} plates and visualization on TLC was achieved by UV light (254 and 365 nm). Flash column chromatography was perfomed on silica gel (400-630 mesh) or Combi*Flash*® R_f^+ system with Redi*Sep*® R_f silica columns (230-400 mesh) using a proper eluent. ^{1}H NMR was recorded on Brucker Ascend 400 MHz, Brucker Avance 500 MHz or Agilent Technologies DD2 600 MHz. Chemical shifts of ^{1}H NMR were reported in ppm referenced to the singlet at 7.26 ppm of $CDCl_3$ or 3.31 ppm of MeOD. ^{13}C NMR was recorded on Brucker Ascend 101 MHz or Brucker Avance 126 MHz and was fully decoupled by broad band proton decoupling. Chemical shifts of ^{13}C NMR were reported in ppm referenced to the centerline of a triplet at 77.16 ppm of $CDCl_3$ or centerline of a septet at 49.00 ppm of MeOD. ^{19}F NMR was recorded on Brucker Ascend 376 MHz was fully coupled by broad band proton. The following abbreviations were used to describe peak splitting patterns when appropriate: br = broad, s = singlet, d = doublet, t = triplet, q = quartet, m = multiplet, dd = doublet of doublet, dt = doublet of triplet, ddd = doublet of doublet of doublet. Coupling constants, *J*, were reported in hertz unit (Hz). High-resolution mass spectra were obtained by using ESI method from Korea Basic Science Institute (Ochang). We have used commercially available blue LEDs as light sources from Kessil PR160-440. The Absorption spectra were measured by a spectrophotometer (V-530 UV/Vis Spectrophotometer, Jasco, Inc.). The emission spectra were measured by a fluorimeter (RF-5301PC Spectrofluorophotometer, SHIMADZU Corp.). Commercial grade reagents and solvents were used without further purification except as indicated below.

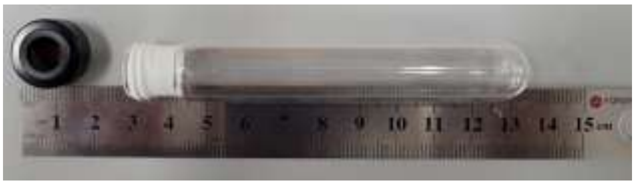

Supplementary Fig. 1 Reaction test tube (12 mL, 16 mm × 100 mm). (Samwoo Kurex, Borosilicate, Type 1 glass)

II. Experimental Procedures

Compounds **1t**[1], **1y**[2], and **2m**[3] were prepared as reported previously.

i) General procedure for Three-Component Reaction (GP1)

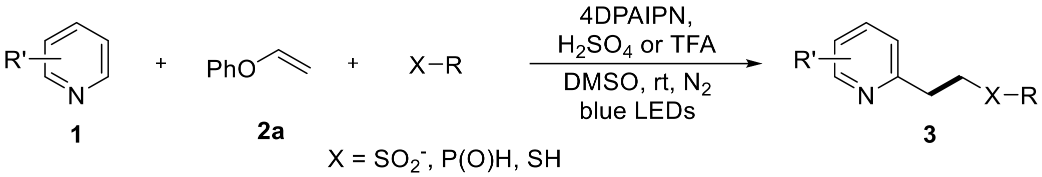

An oven-dried tube (12 mL) was charged with N-heteroarene **1** (1.0 equiv, 0.1 mmol, if solid), X–R (1.5 equiv, 0.15 mmol, if solid) and 4DPAIPN (1.0 mol %, 0.001 mmol). The tube was evacuated and backfilled with nitrogen (repeated three times). H_2SO_4 or TFA (2.0 equiv, 0.2 mmol), N-heteroarene **1** (1.0 equiv, 0.1 mmol, if liquid), X–R (1.5 equiv, 0.15 mmol, if liquid), phenyl vinyl ether **2a** (2.0 equiv, 0.2 mmol) and degassed DMSO (bubbled by argon balloon for 15 min) (0.33 mL, 0.3 M) was added into the reaction tube via syringe. The reaction mixture was stirred under a Kessil blue LED (440 nm, 25%). After being stirred for 16 h, the reaction mixture was quenched with NaOH (1.0 N) solution. The aqueous layer was extracted with EtOAc (three times). The combined organic layer dried over sodium sulfate, filtered and concentrated in the reduced pressure and purified by flash column chromatography on silica gel to afford final product **3**.

ii) General procedures for Vinylation Reaction (GP2)

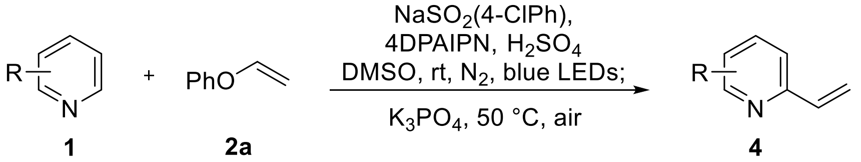

An oven-dried tube (12 mL) was charged with N-heteroarene **1** (1.0 equiv, 0.1 mmol, if solid), $NaSO_2$(4-ClPh) (1.5 equiv, 0.15 mmol) and 4DPAIPN (1.0 mol %, 0.001 mmol). The tube was evacuated and backfilled with nitrogen (repeated three times). H_2SO_4 (2.0 equiv, 0.2 mmol), N-heteroarene **1** (1.0 equiv, 0.1 mmol, if liquid), phenyl vinyl ether **2a** (2.0 equiv, 0.2 mmol) and degassed DMSO (bubbled by argon balloon for 15 min) (0.33 mL, 0.3 M) was added into the reaction tube via syringe. The reaction mixture was stirred under a Kessil blue LED (440 nm, 25%). After being stirred for 16 h, K_3PO_4 (5.0 equiv, 0.5 mmol) was added to the reaction mixture and stirred at 50 °C for 6 h. After termination of reaction, saturated $NaHCO_3$ solution was added for quenching, then the reaction mixture was extracted with EtOAc (three times). The combined organic layer dried over sodium sulfate, filtered and concentrated in the reduced pressure and purified by flash column chromatography on silica gel to

afford final product **4**.

iii) Procedures for Vinylation Reaction in 5 mmol Scale

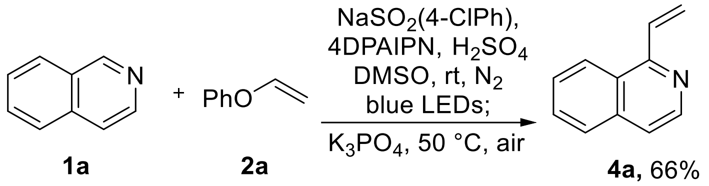

An oven-dried round bottom flask (25 mL) was charged with $NaSO_2$(4-ClPh) (1.5 equiv, 7.5 mmol) and 4DPAIPN (1.0 mol %, 0.05 mmol). The round bottom flask was evacuated and backfilled with nitrogen (repeated three times). H_2SO_4 (2.0 equiv, 10.0 mmol), isoquinoline **1** (1.0 equiv, 5.0 mmol), phenyl vinyl ether **2a** (2.0 equiv, 10.0 mmol) and degassed DMSO (bubbled by argon balloon for 15 min) (16.67 mL, 0.3 M) was added into the round bottom flask via syringe. The reaction mixture was stirred under the two Kessil blue LED (440 nm, 25%). After being stirred for 16 h, K_3PO_4 (5.0 equiv, 25.0 mmol) was added to the reaction mixture and stirred at 50 °C for 24 h. After termination of reaction, saturated $NaHCO_3$ solution was added for quenching, then the reaction mixture was extracted with EtOAc (three times). The combined organic layer dried over sodium sulfate, filtered and concentrated in the reduced pressure and purified by flash column chromatography on silica gel to afford final product **4a** (66%).

iv) Procedures for Synthetic Application Studies

1. Synthesis of 5a

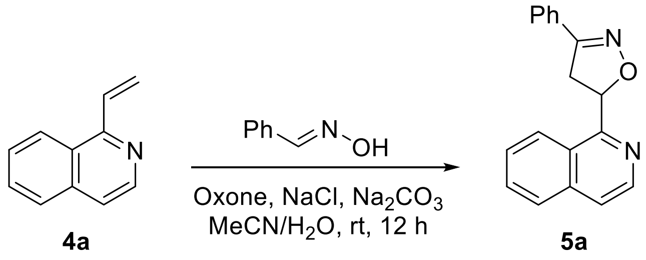

An oven-dried tube (12 mL) was charged with 1-vinylisoquinoline **4a** (1.3 equiv, 0.26 mmol), oxime (1.0 equiv, 0.2 mmol), oxone (1.1 equiv, 0.22 mmol), NaCl (0.7 equiv, 0.14 mmol), Na_2CO_3 (1.5 equiv, 0.3 mmol) and MeCN/H_2O (20:1) (0.87 mL, 0.23 M) was added into the reaction tube via syringe. After being stirred for 12 h, the reaction mixture was filtrated and washed by EtOAc. The combined filtrate was washed with saturated $NaHCO_3$ solution and brine. The combined organic layer dried over sodium sulfate, filtered and concentrated in the reduced pressure and purified by flash column chromatography on silica gel to afford final product **5a**.

2. Synthesis of 5b

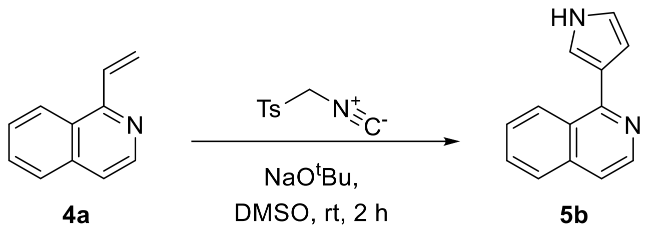

An oven-dried tube (12 mL) was charged with NaO^{t}Bu (2.0 equiv, 0.4 mmol) and DMSO (0.5 mL). A solution of 1-vinylisoquinoline **4a** (1.0 equiv, 0.2 mmol) and TosMIC (1.3 equiv, 0.26 mmol) in DMSO (0.5 mL) was added into the reaction tube via syringe. After being stirred for 2 h, brine was added for quenching, then the reaction mixture was extracted with EtOAc (three times). The combined organic layer dried over sodium sulfate, filtered and concentrated in the reduced pressure and purified by flash column chromatography on silica gel to afford final product **5b**.

3. Synthesis of 5c

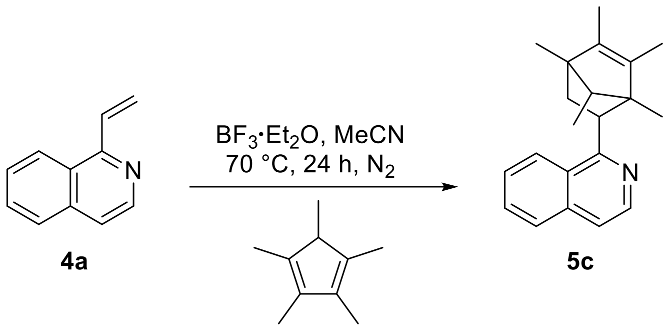

An oven-dried tube (12 mL) was evacuated and backfilled with nitrogen (repeated three times). 1-Vinylisoquinoline **4a** (1.0 equiv, 0.2 mmol), BF_3·Et_2O (0.5 equiv, 0.1 mmol), diene (2.0 equiv, 0.4 mmol) and MeCN (0.4 mL, 0.5 M) was added into the reaction tube via syringe. After being stirred for 24 h at 70 °C, saturated brine solution was added for quenching, then the reaction mixture was extracted with Et_2O (three times). The combined organic layer dried over sodium sulfate, filtered and concentrated in the reduced pressure and purified by flash column chromatography on silica gel to afford final product **5c**.

4. Synthesis of 5d

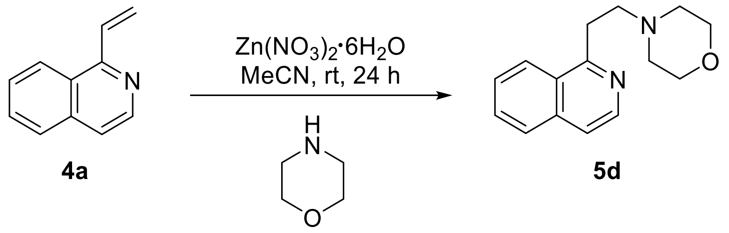

An oven-dried tube (12 mL) was charged with 1-vinylisoquinoline **4a** (1.0 equiv, 0.2 mmol), $Zn(NO_3)_2 \cdot 6H_2O$ (0.05 equiv, 0.01 mmol), morpholine (1.0 equiv, 0.2 mmol) and MeCN (0.2 mL, 1.0 M) was added into the reaction tube via syringe. After stirring for 24 h, the reaction mixture was concentrated in the reduced pressure and purified by flash column chromatography on silica gel to afford final product **5d**.

5. Synthesis of 5e

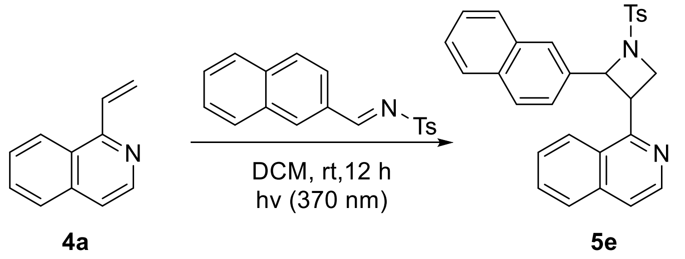

An oven-dried tube (12 mL) was charged with imine (1.0 equiv, 0.2 mmol). The tube was evacuated and backfilled with nitrogen (repeated three times). 1-Vinylisoquinoline **4a** (3.0 equiv, 0.6 mmol) and DCM (2.0 mL, 0.1 M) was added into the reaction tube via syringe. The reaction mixture was stirred under the Kessil blue LED (370 nm, 25%). After being stirred for 12 h, the reaction mixture was concentrated in the reduced pressure and purified by flash column chromatography on silica gel to afford final product **5e**.

6. Synthesis of 5f

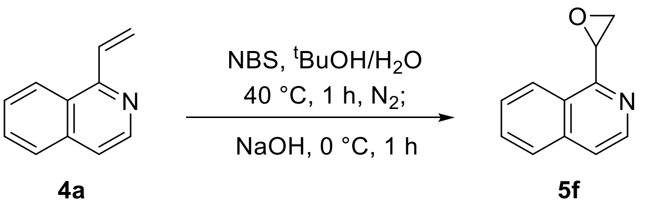

An oven-dried tube (12 mL) was charged with N-bromosuccimide (NBS) (1.2 equiv, 0.24 mmol). The tube was evacuated and backfilled with nitrogen (repeated three times). 1-Vinylisoquinoline **4a** (1.0

equiv, 0.2 mmol) and ${}^tBuOH/H_2O$ (1:3) (0.8 mL, 0.25 M) was added into the reaction tube via syringe. After being stirred for 1 h at 40 °C, NaOH (3.0 N, 0.2 mL) was added to the reaction mixture at 0 °C and stirred for 1 h under same temperature. After termination of reaction, saturated $NaHCO_3$ solution was added for quenching, then the reaction mixture was extracted with Et_2O (three times). The combined organic layer dried over sodium sulfate, filtered and concentrated in the reduced pressure and purified by flash column chromatography on silica gel to afford final product **5f**.

III. Control Experiments

i) Effect of O_2

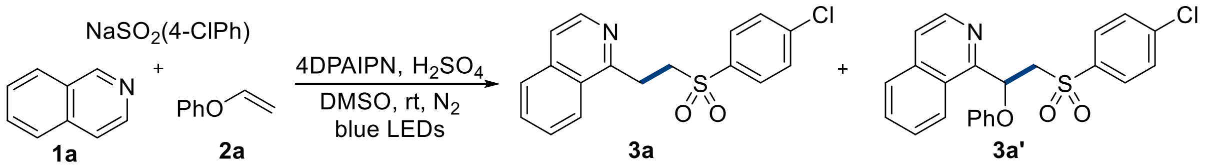

Entry	Deviation from the standard conditions	yield **3a** (%)	yield **3a'** (%)	Remained **1a** (%)
1	Degassed solvent / N_2 atmosphere	81	trace	trace
2	Non-degassed solvent / N_2 atmosphere	72	trace	trace
3	Non-degassed solvent / air atmosphere	53	trace	7
4	Non-degassed solvent / O_2 atmosphere	15	10	55

Supplementary Table 1. Inhibition effects of O_2. Experiments were conducted using DMSO under several conditions to highlight the critical importance of excluding oxygen during the reaction. We performed these tests with and without degassing, in open air, and under an oxygen atmosphere. This approach emphasizes how the presence of oxygen, which can act as an oxidant, interferes with the SCS step.

ii) Deuteration experiment

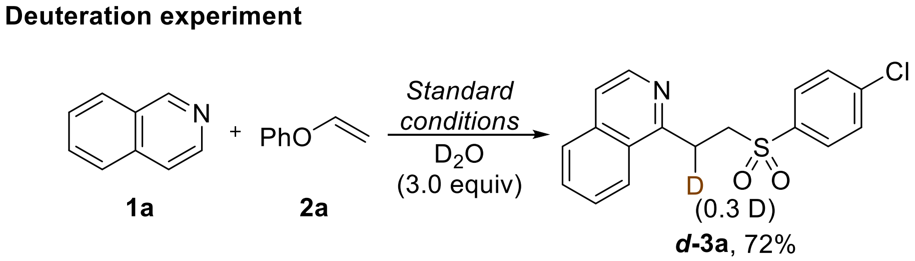

An oven-dried tube (12 mL) was charged with $NaSO_2$(*p*-ClPh) (1.5 equiv, 0.15 mmol) and 4DPAIPN (1.0 mol %, 0.001 mmol). The tube was evacuated and backfilled with nitrogen (repeated three times). H_2SO_4 (2.0 equiv, 0.2 mmol), **1a** (1.0 equiv, 0.1 mmol), D_2O (3.0 equiv, 0.3 mmol), phenyl vinyl ether **2a** (2.0 equiv, 0.2 mmol) and degassed DMSO (bubbled by argon balloon for 15 min) (0.33 mL, 0.3 M) was added into the reaction tube via syringe. The reaction mixture was stirred under a Kessil blue LED (440 nm, 25%). After being stirred for 16 h, the reaction mixture was quenched with NaOH (1.0 N) solution. The aqueous layer was extracted with EtOAc (three times). The combined organic layer dried over sodium sulfate, filtered and concentrated in the reduced pressure and purified by flash column chromatography on silica gel to afford final product ***d*-3a**.

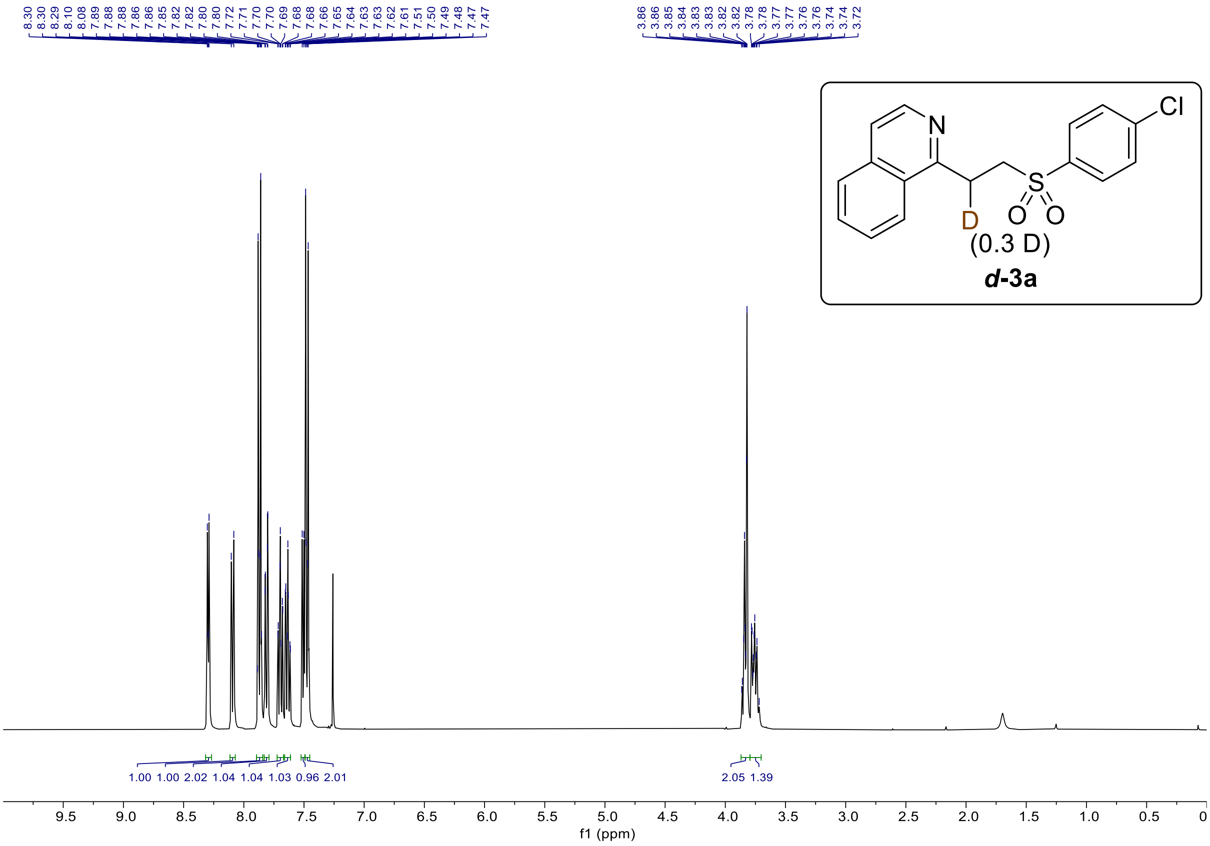

iii) Desulfonylation reaction

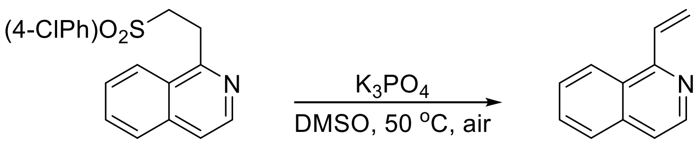

3a **4a,** 82%

An oven-dried tube (12 mL) was charged with **3a** (1.0 equiv, 0.1 mmol), K_3PO_4 (3.0 equiv, 0.3 mmol) and DMSO (0.33 mL, 0.3 M) was added into the reaction tube via syringe. The reaction mixture was

stirred at 50 °C for 6 h. After termination of reaction, saturated $NaHCO_3$ solution was added for quenching, then the reaction mixture was extracted with EtOAc (three times). The combined organic layer dried over sodium sulfate, filtered and concentrated in the reduced pressure and purified by flash column chromatography on silica gel to afford final product **4a**.

iv) Comparison of sufonyl leaving group

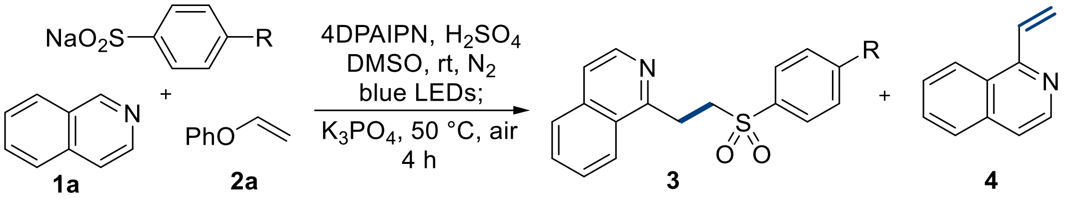

	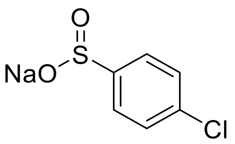	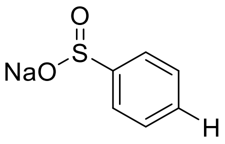	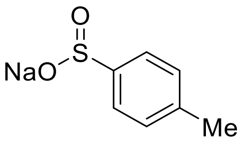
Hammet constant (σ_p)	0.24	0	-0.14
3-Component product (**3**)	4%	25%	50%
Vinylated product **(4)**	67%	46%	22%

Supplementary Table 2. Comparison of leaving group in the one-pot vinylation. The vinylation results showed that the conversion efficiency from intermediate **3** to product **4** enhanced as the electron-withdrawing ability of the substituents increased. This correlation is evident from the increasing values of the Hammett constant.

v) Stern–Volmer Quenching Experiment

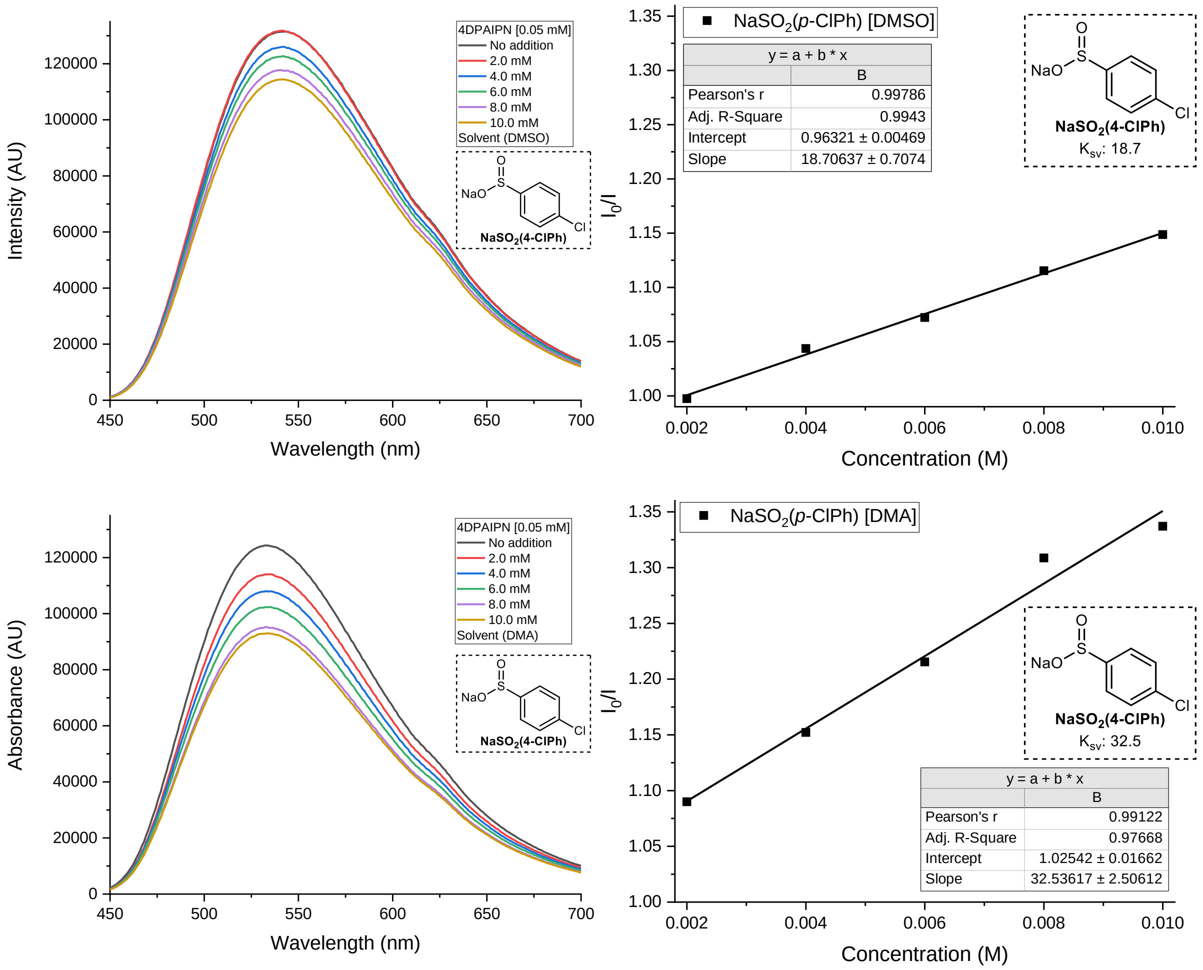

Supplementary Fig. 2. Fluorescence quenching of sodium sulfinate by the 4DPAIPN dissolved in different solvents (DMSO or DMA).

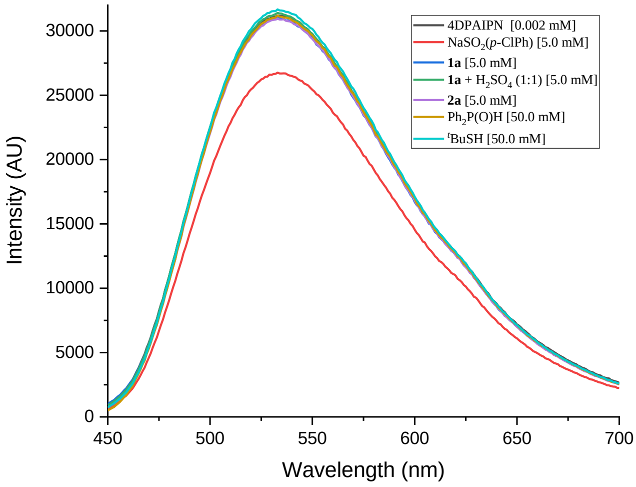

Supplementary Fig. 3 Fluorescence quenching by the 4DPAIPN dissolved in DMA.

vi) Investigation of potential for radical formation from $R_2P(O)H$ and RSH

Despite the thermodynamic feasibility of the oxidation of phosphine oxides (E_{ox} = +1.0 V vs SCE) and thiols (E_{ox} = +0.85 V vs SCE) by 4DPAIPN (E_{ox}^* = +1.10 V vs SCE), negligible quenching was observed in our by Stern-Volmer quenching experiments, while previous research has highlighted the generation of radicals from phosphine oxides and thiols under photocatalysis. Thus, we considered potential explanations for the observed lack of quenching, rapid back electron-transfer (BET). We speculate that the reaction proceeding through a similar mechanism as with sulfinate, despite the absence of quenching by BET.

To provide evidence of radical formation from phosphine oxides and thiols under our reaction conditions, we conducted control experiments. The absence of PC resulted in no formation of radical intermediates, highlighting PC's essential role in generating radicals. Conversely, the presence of PC facilitated the reaction, indicating radical addition to alkenes. This behavior persisted even in the presence of TFA and isoquinoline, suggesting that the reactivity observed is primarily due to the interaction between PC and $Ph_2P(O)H$ and *ᵗ*BuSH.

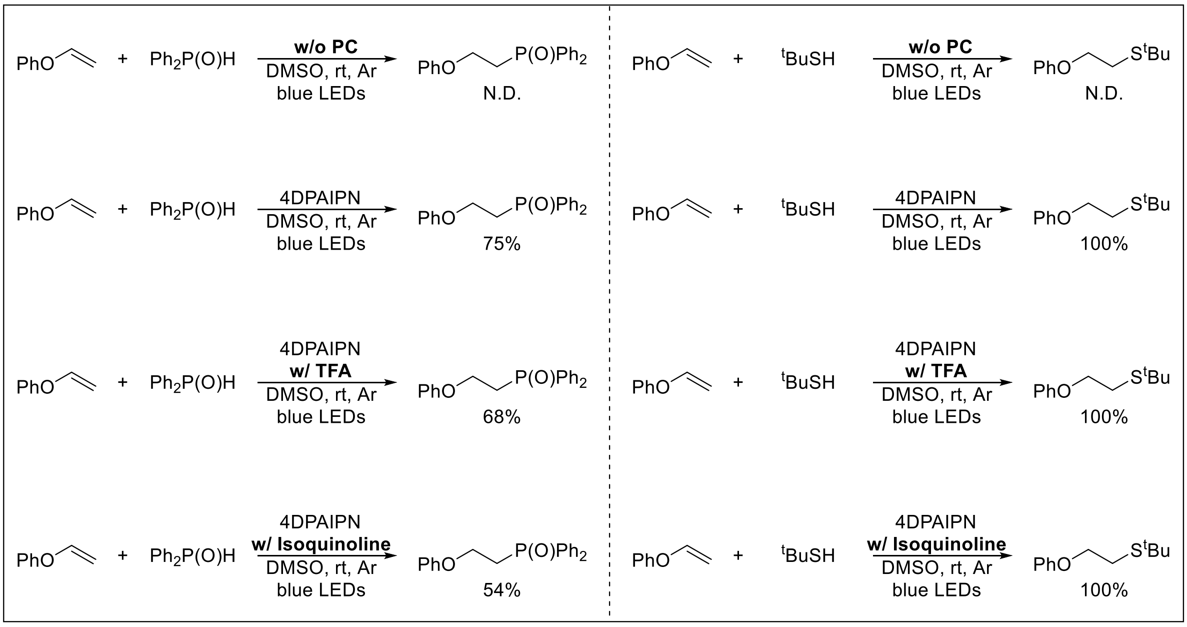

Supplementary Fig. 4 Control experiments.

vii) Radical Trapping Experiments

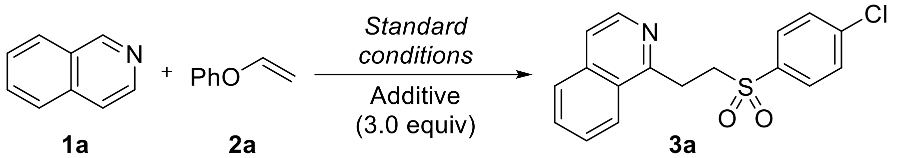

1. With 1,1-diphenylethene

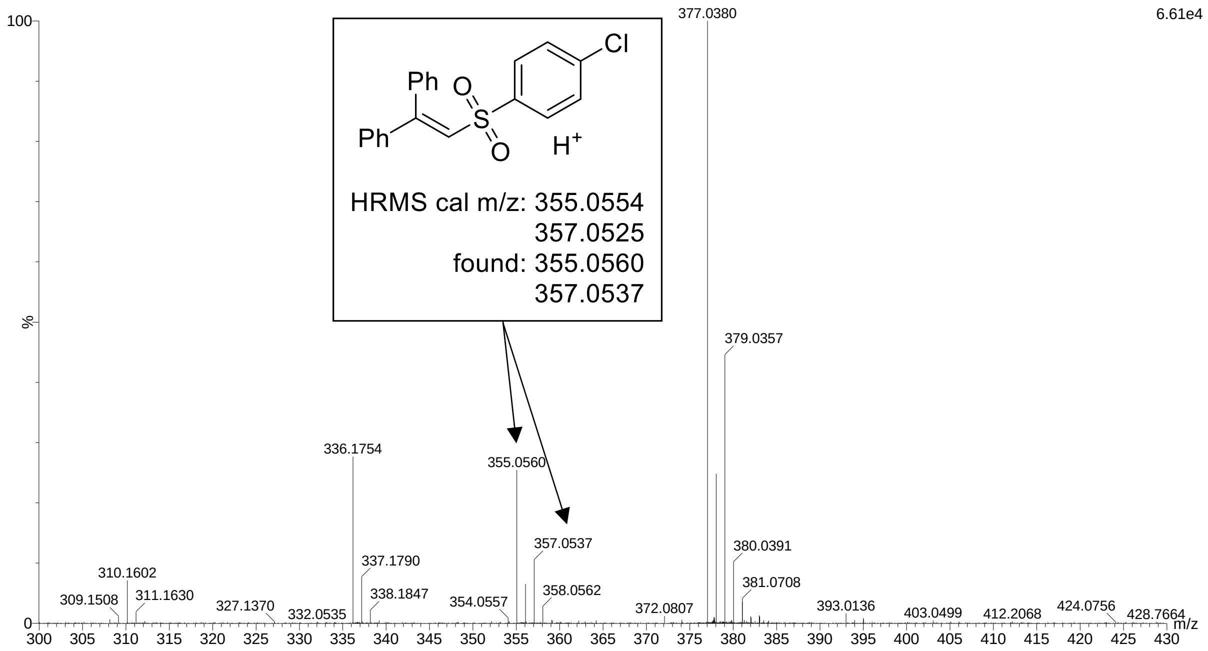

Supplementary Fig. 5 HR-MS analysis of radical trapping experiment with 1,1-diphenylethene.

2. With TEMPO

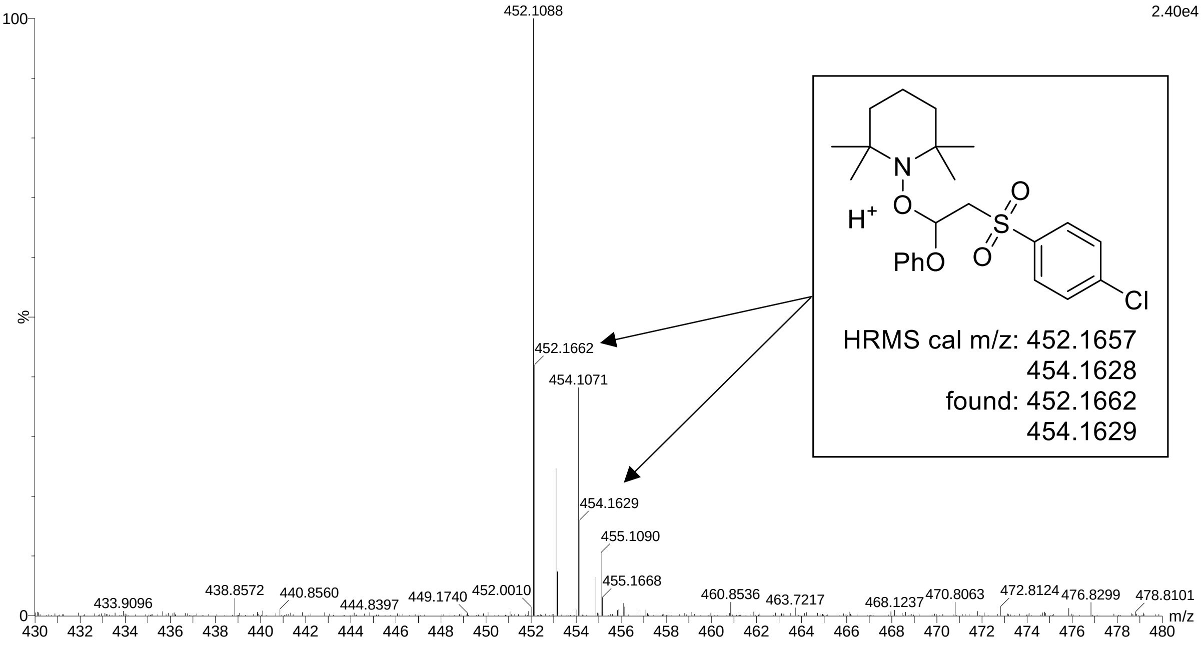

Supplementary Fig. 6 HR-MS analysis of radical trapping experiment with TEMPO.

3. With BHT

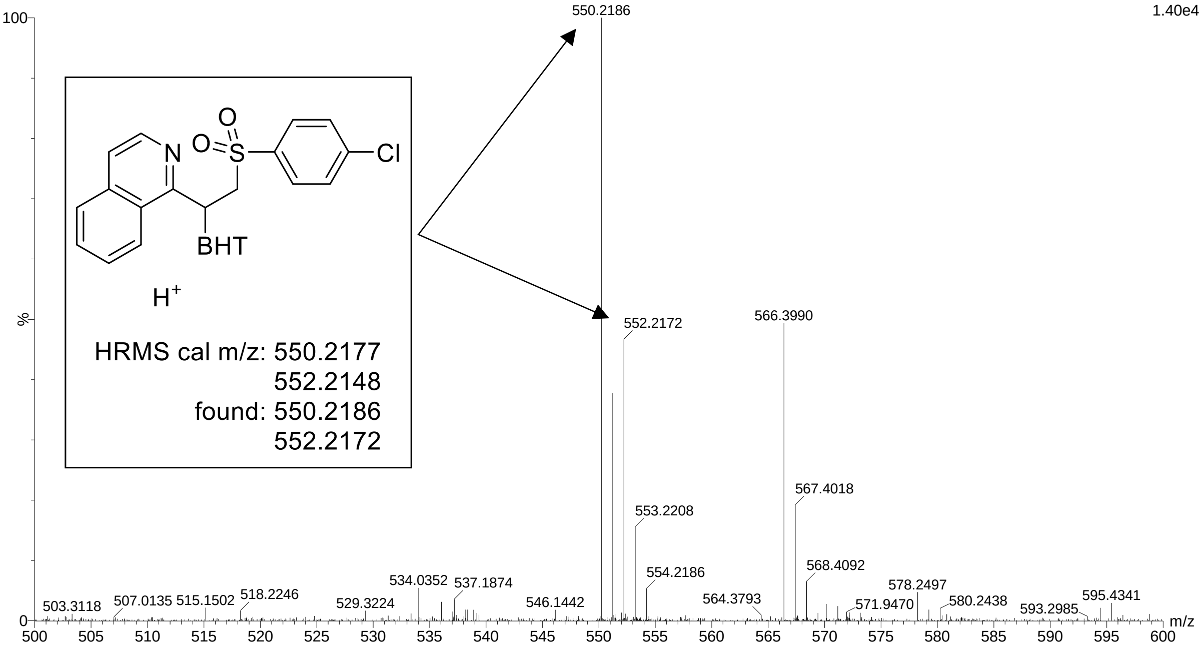

Supplementary Fig. 7 HR-MS analysis of radical trapping experiment with BHT.

viii) Leaving Group Detection Experiment

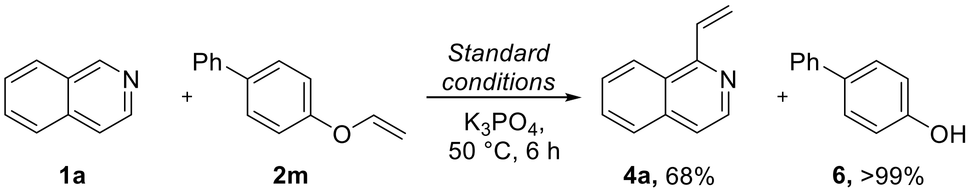

An oven-dried tube (12 mL) was charged with (4-phenyl)phenyl vinyl ether **2m** (2.0 equiv, 0.2 mmol), $NaSO_2$(4-ClPh) (1.5 equiv, 0.15 mmol) and 4DPAIPN (1 mol%, 0.001 mmol). The tube was evacuated and backfilled with nitrogen (repeated three times). H_2SO_4 (2.0 equiv, 0.2 mmol), isoquinoline **1a** (1.0 equiv, 0.1 mmol) and degassed DMSO-d_6 (bubbled by argon balloon for 15 min) (0.33 mL, 0.3 M) was added into the reaction tube via syringe. The reaction mixture was stirred under a Kessil blue LED (440 nm, 25%). After being stirred for 16 h, K_3PO_4 (5.0 equiv, 0.5 mmol) was added to the reaction mixture and stirred at 50 °C for 6 h. After termination of reaction, yields are determined by ^{1}H NMR spectroscopy.

ix) Measurement of Reaction Quantum Yield

Blue LED (λ_{max} = 440 nm) was used for measurement of quantum yield. The light intensity at 440 nm of blue LED was determined already in our previous paper.[4]

$$\text{Photon flux} = 2.78 \times 10^{-7} \text{ einstein/s}$$

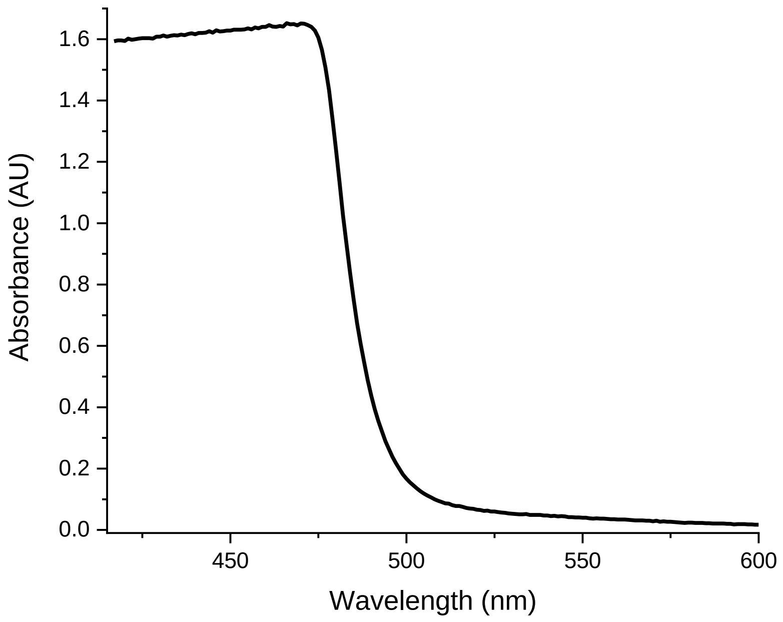

Supplementary Fig. 8 Absorption spectra for reaction mixture (0.3 M in DMSO).

The reaction mixture (0.3 mmol scale, 1.0 mL (0.3 M)) was stirred upon irradiation using blue LED (λ_{max} = 440 nm) for 600 s. The yield of product **3a** was measured to be 7% (2.1×10^{-5} mol of **3a**) by ^{1}H NMR analysis. The reaction quantum yield (Φ) was calculated using Supplementary Equation (1).

Photon flux = 2.78×10^{-7} einstein·s^{-1}

t = reaction time

f = fraction of incident light absorbed by the catalyst, determined using Supplementary Equation (2).

An absorption spectrum of the reaction mixture gave an absorbance value of 1.616 at 440 nm (Abs_{440nm}) (Figure S5), indicating that the fraction of light absorbed by the photosensitizer (f) is 0.976.

$$\Phi = \frac{mol\ of\ product}{photon\ flux \cdot t \cdot f} \quad \text{Supplementary Equation (1)}$$

$$f = 1 - 10^{-Abs_{440nm}} \quad \text{Supplementary Equation (2)}$$

$$\Phi = \frac{2.1 \times 10^{-5}}{2.78 \times 10^{-7} \times 600 \times 0.976} = 0.13$$

The reaction quantum yield (Φ) was calculated to be 0.13.

IV. Reference.

1. M. A. J. Duncton, M. A. Estiarte, R. J. Johnson, M. Cox, D. J. R. O'Mahony, W. T. Edwards, M. G. Kelly, *J. Org. Chem.* **2009,** *74*, 6354–6357.

2. R. A. Garza-Sanchez, A. Tlahuext-Aca, G. Tavakoli, F. Glorius, *ACS Catal.* **2017**, *7*, 4057–4061.

3. Q.-Q. Zhao, X.-S. Zhou, S.-H. Xu, Y.-L. Wu, W.-J. Xiao, J.-R. Chen, *Org. Lett.* **2020**, *22*, 2470–2475.
4. C. Kim, J. Jeong, M. Vellakkaran, S. Hong, *ACS Catal.* **2022**, *12*, 13225–13233.

V. Compound Characterizations

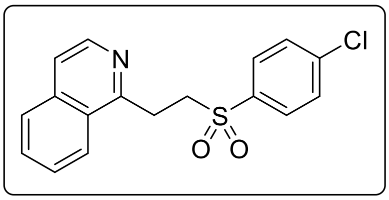

1-(2-((4-chlorophenyl)sulfonyl)ethyl)isoquinoline (3a).

Prepared according to **GP1**. Purified by flash chromatography on silica gel (dichloromethane/methanol = 10:1), compound **3a** (27.0 mg, 81%) was obtained. Yellow liquid. ^{1}H NMR (400 MHz, Chloroform-*d*) δ 8.30 (d, *J* = 5.7 Hz, 1H), 8.14 – 8.07 (m, 1H), 7.90 – 7.85 (m, 2H), 7.84 – 7.79 (m, 1H), 7.67 (dddd, *J* = 24.8, 8.3, 6.9, 1.3 Hz, 2H), 7.60 – 7.40 (m, 3H), 3.84 (ddd, *J* = 10.2, 6.2, 2.0 Hz, 2H), 3.80 – 3.72 (m, 2H). ^{13}C NMR (101 MHz, Chloroform-*d*) δ 156.4, 141.6, 140.6, 137.9, 136.2, 130.4, 129.72, 129.68, 127.9, 127.7, 126.9, 124.4, 120.3, 54.5, 27.2. HRMS (ESI) m/z calcd. For $[C_{17}H_{15}ClNO_2S]^+$: 332.0507, found : 332.0512.

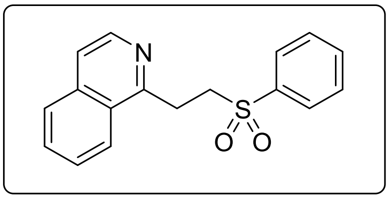

1-(2-(phenylsulfonyl)ethyl)isoquinoline (3b).

Prepared according to **GP1**. Purified by flash chromatography on silica gel (hexane/ethyl acetate = 4:1), compound **3b** (23.1 mg, 78%) was obtained. Pale yellow solid. ^{1}H NMR (400 MHz, Chloroform-*d*) δ 8.30 (d, *J* = 5.7 Hz, 1H), 8.10 (dd, *J* = 8.4, 1.2 Hz, 1H), 8.04 – 7.93 (m, 2H), 7.86 – 7.76 (m, 1H), 7.75 – 7.66 (m, 1H), 7.65 – 7.59 (m, 2H), 7.58 – 7.52 (m, 2H), 7.50 (d, *J* = 5.7 Hz, 1H), 3.98 – 3.61 (m, 4H). ^{13}C NMR (101 MHz, Chloroform-*d*) δ 156.6, 141.5, 139.4, 136.2, 133.8, 130.4, 129.4, 128.2, 127.8, 127.6, 126.9, 124.4, 120.2, 54.5, 27.2. HRMS (ESI) m/z calcd. For $[C_{17}H_{16}NO_2S]^+$: 298.0896. found : 298.0902.

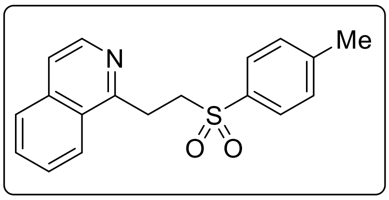

1-(2-tosylethyl)isoquinoline (3c).

Prepared according to **GP1**. Purified by flash chromatography on silica gel (dichloromethane/methanol = 10:1), compound **3c** (25.5 mg, 82%) was obtained. Yellow liquid. ^{1}H NMR (400 MHz, Chloroform-*d*) δ 8.31 (dd, *J* = 5.7, 0.8 Hz, 1H), 8.10 (dt, *J* = 8.3, 1.2 Hz, 1H), 7.89 – 7.77 (m, 3H), 7.69 (ddt, *J* = 8.2, 6.8, 1.3 Hz, 1H), 7.62 (ddt, *J* = 8.3, 6.9, 1.4 Hz, 1H), 7.50 (d, *J* = 5.8 Hz, 1H), 7.33 (d, *J* = 8.0 Hz, 2H), 3.84 – 3.70 (m, 4H), 2.43 (s, 3H). ^{13}C NMR (101 MHz, Chloroform-*d*) δ 156.8, 144.8, 141.6, 136.4, 136.2, 130.4, 130.0, 128.3, 127.8, 127.6, 126.9, 124.5, 120.2, 54.6, 27.4, 21.7. HRMS (ESI) m/z calcd. For $[C_{18}H_{18}NO_2S]^+$: 312.1053, found : 312.1059.

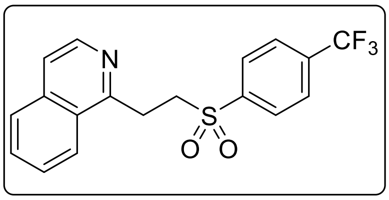

1-(2-((4-(trifluoromethyl)phenyl)sulfonyl)ethyl)isoquinoline (3d).

Prepared according to **GP1**. Purified by flash chromatography on silica gel (hexane/ethyl acetate = 4:1), compound **3d** (21.5 mg, 59%) was obtained. White solid. ^{1}H NMR (600 MHz, Chloroform-*d*) δ 8.26 (d, *J* = 5.7 Hz, 1H), 8.10 (dt, *J* = 8.5, 1.0 Hz, 1H), 8.04 (d, *J* = 8.1 Hz, 2H), 7.80 (d, *J* = 8.1 Hz, 1H), 7.74 (d, *J* = 8.2 Hz, 2H), 7.70 (ddd, *J* = 8.2, 6.9, 1.1 Hz, 1H), 7.64 (ddd, *J* = 8.2, 6.9, 1.2 Hz, 1H), 7.48 (d, *J* = 5.7 Hz, 1H), 3.93 – 3.88 (m, 2H), 3.79 (dd, *J* = 9.4, 6.2 Hz, 2H). ^{13}C NMR (101 MHz, Chloroform-*d*) δ 156.1, 142.9 (d, *J* = 1.5 Hz), 141.5, 136.2, 135.4 (q, *J* = 33.1 Hz), 130.5, 128.8, 127.9, 127.7, 126.9, 126.4 (q, *J* = 3.7 Hz), 124.3, 123.2 (q, *J* = 273.1 Hz), 120.3, 54.3, 27.0. ^{19}F NMR (376 MHz, Chloroform-*d*) δ -63.27. HRMS (ESI) m/z calcd. For $[C_{18}H_{15}F_3NO_2S]^+$: 366.0770, found : 366.0777.

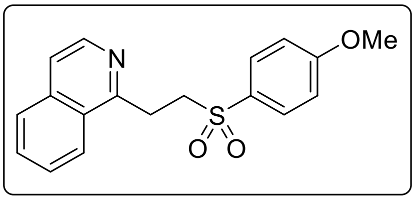

1-(2-((4-methoxyphenyl)sulfonyl)ethyl)isoquinoline (3e).

Prepared according to **GP1**. Purified by flash chromatography on silica gel (hexane/ethyl acetate = 4:1), compound **3e** (20.3 mg, 62%) was obtained. Colorless liquid. ^{1}H NMR (600 MHz, Chloroform-*d*) δ 8.31 (d, *J* = 5.7 Hz, 1H), 8.18 – 8.07 (m, 1H), 7.99 – 7.85 (m, 2H), 7.80 (dt, *J* = 8.2, 0.9 Hz, 1H), 7.69 (ddd, *J* = 8.2, 6.9, 1.2 Hz, 1H), 7.62 (ddd, *J* = 8.3, 6.9, 1.3 Hz, 1H), 7.50 (d, *J* = 5.7 Hz, 1H), 7.08 – 6.82 (m, 2H), 3.86 (s, 3H), 3.82 – 3.70 (m, 4H). ^{13}C NMR (101 MHz, Chloroform-*d*) δ 163.9, 156.8, 141.6, 136.2, 130.9, 130.39, 130.37, 127.8, 127.6, 126.9, 124.5, 120.2, 114.6, 55.8, 54.8, 27.5. HRMS (ESI) m/z calcd. For $[C_{18}H_{18}NO_3S]^+$: 328.1002, found : 328.1008.

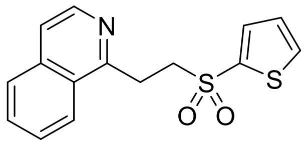

1-(2-(thiophen-2-ylsulfonyl)ethyl)isoquinoline (3f).

Prepared according to **GP1**. Purified by flash chromatography on silica gel (hexane/ethyl acetate = 4:1), compound **3f** (19.1 mg, 63%) was obtained. White solid. ^{1}H NMR (600 MHz, Chloroform-*d*) δ 8.34 (d, *J* = 5.7 Hz, 1H), 8.11 (dt, *J* = 8.5, 1.0 Hz, 1H), 7.91 – 7.79 (m, 1H), 7.75 (dd, *J* = 3.7, 1.4 Hz, 1H), 7.73 – 7.68 (m, 2H), 7.64 (ddd, *J* = 8.4, 6.9, 1.3 Hz, 1H), 7.53 (d, *J* = 5.7 Hz, 1H), 7.14 (dd, *J* = 5.0, 3.8 Hz, 1H), 3.97 – 3.91 (m, 2H), 3.85 – 3.79 (m, 2H). ^{13}C NMR (101 MHz, Chloroform-*d*) δ 156.5, 141.6, 140.4, 136.2, 134.3, 134.2, 130.4, 128.1, 127.9, 127.7, 126.9, 124.4, 120.3, 55.9, 27.7. HRMS (ESI) m/z calcd. For $[C_{15}H_{14}NO_2S_2]^+$: 304.0460, found : 304.0465.

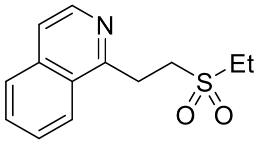

1-(2-(ethylsulfonyl)ethyl)isoquinoline (3g).

Prepared according to **GP1**. Purified by flash chromatography on silica gel (hexane/ethyl acetate = 3:1), compound **3g** (15.2 mg, 61%) was obtained. Colorless solid. ^{1}H NMR (400 MHz, Chloroform-*d*) δ 8.40 (d, *J* = 5.7 Hz, 1H), 8.17 (dt, *J* = 8.3, 1.0 Hz, 1H), 7.99 – 7.81 (m, 1H), 7.71 (ddd, *J* = 8.2, 6.9, 1.3 Hz, 1H), 7.65 (ddd, *J* = 8.2, 6.8, 1.4 Hz, 1H), 7.57 (d, *J* = 5.7 Hz, 1H), 3.93 – 3.82 (m, 2H), 3.79 – 3.65 (m, 2H), 3.07 (q, *J* = 7.5 Hz, 2H), 1.45 (t, *J* = 7.5 Hz, 3H). ^{13}C NMR (101 MHz, Chloroform-*d*) δ 156.7, 141.5, 136.2, 130.5, 127.9, 127.6, 127.0, 124.5, 120.4, 49.8, 48.0, 26.4, 6.8. HRMS (ESI) m/z calcd. For $[C_{13}H_{16}NO_2S]^+$: 250.0896, found : 250.0902.

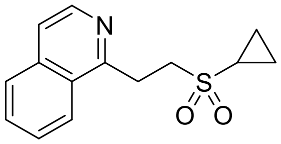

1-(2-(cyclopropylsulfonyl)ethyl)isoquinoline (3h).

Prepared according to **GP1**. Purified by flash chromatography on silica gel (dichloromethane/methanol = 10:1), compound **3h** (21.4 mg, 82%) was obtained. Yellow liquid. ^{1}H NMR (400 MHz, Chloroform-*d*) δ 8.42 (d, *J* = 5.7 Hz, 1H), 8.18 (dd, *J* = 8.5, 1.3 Hz, 1H), 7.88 – 7.82 (m, 1H), 7.69 (dddd, *J* = 25.0, 8.2, 6.9, 1.3 Hz, 2H), 7.58 (d, *J* = 5.7 Hz, 1H), 3.94 – 3.84 (m, 2H), 3.84 – 3.72 (m, 2H), 2.46 (tt, *J* =

8.0, 4.8 Hz, 1H), 1.35 – 1.21 (m, 2H), 1.09 – 0.94 (m, 2H). ^{13}C NMR (101 MHz, Chloroform-*d*) δ 156.9, 141.6, 136.2, 130.5, 127.9, 127.7, 127.0, 124.6, 120.4, 52.0, 29.9, 26.8, 5.0. HRMS (ESI) m/z calcd. For $[C_{14}H_{16}NO_2S]^+$: 262.0896, found : 262.0903.

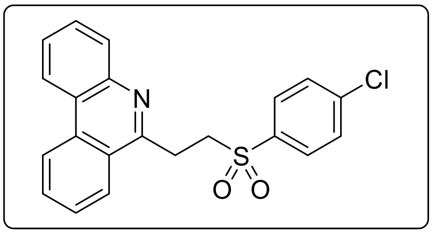

6-(2-((4-chlorophenyl)sulfonyl)ethyl)phenanthridine (3i).

Prepared according to **GP1**. Purified by flash chromatography on silica gel (hexane/ethyl acetate = 4:1), compound **3i** (30.5 mg, 80%) was obtained. White solid. ^{1}H NMR (500 MHz, Chloroform-*d*) δ 8.63 (dd, *J* = 8.2, 1.1 Hz, 1H), 8.52 (dd, *J* = 8.2, 1.4 Hz, 1H), 8.18 (dd, *J* = 8.3, 1.2 Hz, 1H), 7.95 (dd, *J* = 8.1, 1.4 Hz, 1H), 7.92 – 7.82 (m, 3H), 7.79 – 7.67 (m, 2H), 7.63 (ddd, *J* = 8.3, 7.0, 1.4 Hz, 1H), 7.51 – 7.41 (m, 2H), 4.06 – 3.96 (m, 2H), 3.90 – 3.79 (m, 2H). ^{13}C NMR (101 MHz, Chloroform-*d*) δ 156.2, 143.2, 140.5, 138.0, 132.9, 130.9, 129.72, 129.71, 129.6, 128.9, 127.8, 127.1, 125.3, 125.1, 123.8, 122.8, 122.1, 54.1, 27.8. HRMS (ESI) m/z calcd. For $[C_{21}H_{17}ClNO_2S]^+$: 382.0663, found : 382.0668.

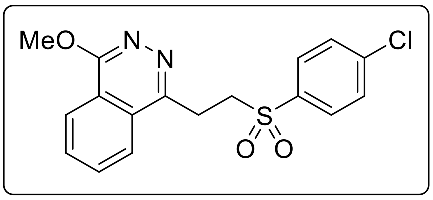

1-(2-((4-chlorophenyl)sulfonyl)ethyl)-4-methoxyphthalazine (3j).

Prepared according to **GP1**. Purified by flash chromatography on silica gel (hexane/ethyl acetate = 2:1), compound **3j** (18.7 mg, 52%) was obtained. Yellow liquid. ^{1}H NMR (400 MHz, Chloroform-*d*) δ 8.28 – 8.14 (m, 1H), 8.05 – 7.94 (m, 1H), 7.94 – 7.81 (m, 4H), 7.59 – 7.42 (m, 2H), 4.21 (s, 3H), 3.98 – 3.84 (m, 2H), 3.84 – 3.62 (m, 2H). ^{13}C NMR (101 MHz, Chloroform-*d*) δ 160.6, 151.6, 140.7, 137.8, 132.7, 132.2, 129.7, 129.6, 127.7, 123.9, 123.2, 119.9, 54.9, 53.8, 25.0. HRMS (ESI) m/z calcd. For $[C_{17}H_{16}ClN_2O_3S]^+$: 363.0565, found : 363.0571.

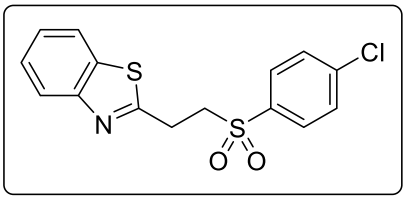

2-(2-((4-chlorophenyl)sulfonyl)ethyl)benzo[d]thiazole (3k).

Prepared according to **GP1**. Purified by flash chromatography on silica gel (hexane/ethyl acetate = 5:1), compound **3k** (18.2 mg, 54%) was obtained. White solid. ^{1}H NMR (400 MHz, Chloroform-*d*) δ 7.70 –

7.59 (m, 4H), 7.32 – 7.12 (m, 4H), 3.60 – 3.52 (m, 2H), 3.40 – 3.30 (m, 2H). ^{13}C NMR (101 MHz, Chloroform-*d*) δ 166.4, 152.5, 141.0, 137.3, 135.0, 129.82, 129.79, 126.6, 125.6, 122.7, 121.8, 54.6, 27.4. HRMS (ESI) m/z calcd. For $[C_{15}H_{12}ClNO_2S_2]^+$: 338.0071, found : 338.0075.

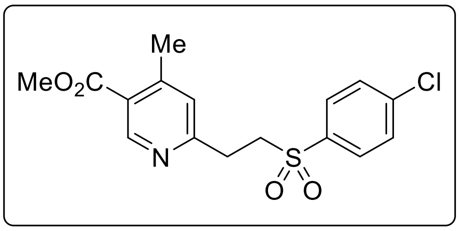

methyl 6-(2-((4-chlorophenyl)sulfonyl)ethyl)-4-methylnicotinate (3l).

Prepared according to **GP1**. Purified by flash chromatography on silica gel ((hexane/ethyl acetate = 5:1), compound **3l** (19.9 mg, 56%) was obtained. Yellow liquid. ^{1}H NMR (400 MHz, Chloroform-*d*) δ 8.86 (s, 1H), 7.86 – 7.75 (m, 2H), 7.54 – 7.44 (m, 2H), 7.02 (s, 1H), 3.91 (s, 3H), 3.68 – 3.58 (m, 2H), 3.26 – 3.17 (m, 2H), 2.57 (s, 3H). ^{13}C NMR (101 MHz, Chloroform-*d*) δ 166.4, 159.9, 151.5, 150.5, 140.7, 137.7, 129.8, 129.7, 126.2, 124.3, 54.8, 52.3, 30.7, 21.4. HRMS (ESI) m/z calcd. For $[C_{16}H_{17}ClNO_4S]^+$: 354.0561, found : 354.0568.

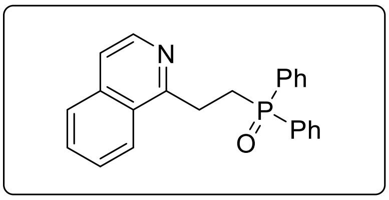

(2-(isoquinolin-1-yl)ethyl)diphenylphosphine oxide (3m).

Prepared according to **GP1**. Purified by flash chromatography on silica gel (dichloromethane/methanol = 10:1), compound **3m** (23.9 mg, 67%) was obtained. Yellow liquid. ^{1}H NMR (400 MHz, Chloroform-*d*) δ 8.39 (d, *J* = 5.7 Hz, 1H), 8.14 (dd, *J* = 8.4, 1.1 Hz, 1H), 7.88 – 7.73 (m, 5H), 7.65 (ddd, *J* = 8.2, 6.9, 1.2 Hz, 1H), 7.57 (ddd, *J* = 8.4, 6.9, 1.4 Hz, 1H), 7.53 – 7.42 (m, 7H), 3.70 – 3.59 (m, 2H), 3.02 – 2.90 (m, 2H). ^{13}C NMR (101 MHz, Chloroform-*d*) δ 159.7 (d, *J* = 14.4 Hz), 141.5, 136.2, 133.4, 132.4, 131.9 (d, *J* = 2.8 Hz), 131.0 (d, *J* = 9.5 Hz), 130.3, 128.8 (d, *J* = 11.7 Hz), 127.5 (d, *J* = 17.9 Hz), 126.9, 125.1, 120.0, 28.2 (d, *J* = 71.8 Hz), 26.4 (d, *J* = 2.0 Hz). HRMS (ESI) m/z calcd. For $[C_{23}H_{21}NOP]^+$: 358.1355, found : 358.1362.

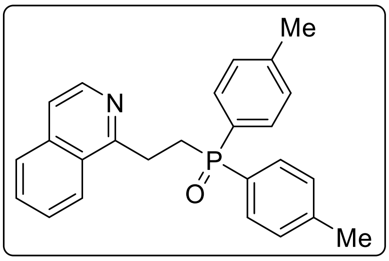

(2-(isoquinolin-1-yl)ethyl)di-p-tolylphosphine oxide (3n).

Prepared according to **GP1**. Purified by flash chromatography on silica gel (dichloromethane/methanol = 10:1), compound **3n** (26.6 mg, 69%) was obtained. Yellow liquid. ^{1}H NMR (400 MHz, Chloroform-*d*) δ 8.38 (d, *J* = 5.8 Hz, 1H), 8.18 (dd, *J* = 8.5, 1.1 Hz, 1H), 7.83 – 7.77 (m, 1H), 7.71 (d, *J* = 1.8 Hz, 1H), 7.70 – 7.65 (m, 4H), 7.59 (ddd, *J* = 8.4, 6.9, 1.4 Hz, 1H), 7.53 (d, *J* = 5.8 Hz, 1H), 7.25 – 7.22 (m, 4H), 3.69 – 3.60 (m, 2H), 2.96 – 2.88 (m, 2H), 2.36 (s, 6H). ^{13}C NMR (101 MHz, Chloroform-*d*) δ 159.9 (d, *J* = 14.3 Hz), 142.3 (d, *J* = 2.8 Hz), 140.7, 136.4, 131.0 (d, *J* = 9.8 Hz), 130.7, 129.6 (d, *J* = 101.3 Hz), 129.5 (d, *J* = 12.1 Hz), 127.8, 127.4, 126.9, 125.4, 120.1, 28.5 (d, *J* = 71.7 Hz), 26.3 (d, *J* = 2.0 Hz), 21.7 (d, *J* = 1.3 Hz). HRMS (ESI) m/z calcd. For $[C_{25}H_{25}NOP]^+$: 386.1668, found : 386.1674.

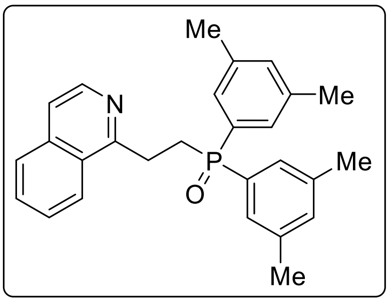

bis(3,5-dimethylphenyl)(2-(isoquinolin-1-yl)ethyl)phosphine oxide (3o).

Prepared according to **GP1**. Purified by flash chromatography on silica gel (dichloromethane/methanol = 10:1), compound **3o** (25.2 mg, 61%) was obtained. Yellow liquid. ^{1}H NMR (400 MHz, Chloroform-*d*) δ 8.39 (d, *J* = 5.9 Hz, 1H), 8.24 (d, *J* = 8.5 Hz, 1H), 7.81 (d, *J* = 8.1 Hz, 1H), 7.71 (t, *J* = 7.5 Hz, 1H), 7.62 (t, *J* = 7.7 Hz, 1H), 7.56 (d, *J* = 5.9 Hz, 1H), 7.41 (dd, *J* = 11.7, 1.6 Hz, 4H), 7.09 (s, 2H), 3.69 (q, *J* = 8.8 Hz, 2H), 2.99 – 2.88 (m, 2H), 2.31 (s, 12H). ^{13}C NMR (101 MHz, Chloroform-*d*) δ 160.1 (d, *J* = 13.9 Hz), 138.5 (d, *J* = 12.4 Hz), 136.5, 133.6 (d, *J* = 2.9 Hz), 132.6 (d, *J* = 97.9 Hz), 128.4 (d, *J* = 9.4 Hz), 128.1, 127.5, 126.8, 125.7, 120.4, 28.5 (d, *J* = 70.6 Hz), 26.0, 21.4. HRMS (ESI) m/z calcd. For $[C_{27}H_{29}NOP]^+$: 414.1981, found : 414.1986.

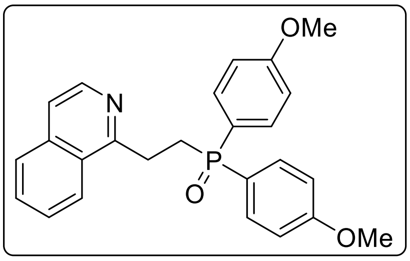

(2-(isoquinolin-1-yl)ethyl)bis(4-methoxyphenyl)phosphine oxide (3p).

Prepared according to **GP1**. Purified by flash chromatography on silica gel (dichloromethane/methanol = 10:1), compound **3p** (19.9 mg, 48%) was obtained. Yellow liquid. ^{1}H NMR (400 MHz, Chloroform-*d*) δ 8.38 (dd, *J* = 5.7, 1.7 Hz, 1H), 8.17 (d, *J* = 8.4 Hz, 1H), 7.78 (dd, *J* = 8.2, 1.9 Hz, 1H), 7.75 – 7.63 (m, 5H), 7.61 – 7.55 (m, 1H), 7.51 (d, *J* = 5.7 Hz, 1H), 6.94 (dt, *J* = 8.8, 1.8 Hz, 4H), 3.81 (t, *J* = 1.7 Hz, 6H), 3.67 – 3.59 (m, 2H), 2.93 – 2.85 (m, 2H). ^{13}C NMR (101 MHz, Chloroform-*d*) δ 162.4 (d, *J* =

2.8 Hz), 160.0 (d, *J* = 14.4 Hz), 140.9, 136.3, 132.8 (d, *J* = 10.7 Hz), 130.5, 127.7, 127.4, 126.9, 125.3, 124.3 (d, *J* = 105.3 Hz), 120.1, 114.3 (d, *J* = 12.7 Hz), 55.4, 28.8 (d, *J* = 72.3 Hz), 26.4 (d, *J* = 2.0 Hz). HRMS (ESI) m/z calcd. For $[C_{25}H_{25}NO_3P]^+$: 418.1567, found : 418.1573.

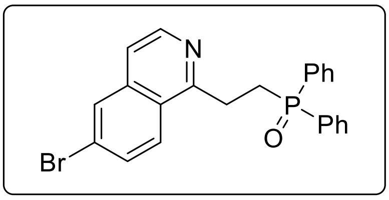

(2-(6-bromoisoquinolin-1-yl)ethyl)diphenylphosphine oxide (3q).

Prepared according to **GP1**. Purified by flash chromatography on silica gel (dichloromethane/methanol = 10:1), compound **3q** (25.3 mg, 58%) was obtained. Yellow liquid. ^{1}H NMR (400 MHz, Chloroform-*d*) δ 8.40 (d, *J* = 5.8 Hz, 1H), 8.04 (d, *J* = 9.0 Hz, 1H), 7.96 (d, *J* = 2.0 Hz, 1H), 7.81 (ddt, *J* = 11.6, 6.7, 1.6 Hz, 4H), 7.66 (dd, *J* = 9.0, 2.0 Hz, 1H), 7.47 (ddq, *J* = 11.9, 7.9, 5.8 Hz, 7H), 3.69 – 3.58 (m, 2H), 3.01 – 2.90 (m, 2H). ^{13}C NMR (101 MHz, Chloroform-*d*) δ 160.0 (d, *J* = 13.8 Hz), 137.5, 133.3, 132.8 (d, *J* = 98.9 Hz), 132.0 (d, *J* = 2.8 Hz), 131.3, 130.9 (d, *J* = 9.5 Hz), 129.6, 128.9 (d, *J* = 11.7 Hz), 127.1, 125.3, 119.1, 28.2 (d, *J* = 71.6 Hz), 26.2. HRMS (ESI) m/z calcd. For $[C_{23}H_{20}BrNOP]^+$: 436.0460, found : 436.0466.

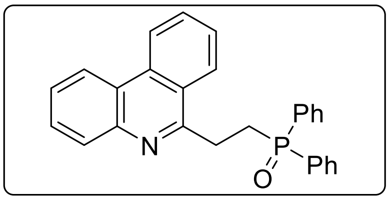

(2-(phenanthridin-6-yl)ethyl)diphenylphosphine oxide (3r).

Prepared according to **GP1**. Purified by flash chromatography on silica gel (hexane/ethyl acetate = 4:1), compound **3r** (29.3 mg, 72%) was obtained. White solid. ^{1}H NMR (400 MHz, Chloroform-*d*) δ 8.58 (dd, *J* = 8.3, 1.1 Hz, 1H), 8.50 (dd, *J* = 8.2, 1.4 Hz, 1H), 8.21 (dd, *J* = 8.3, 1.2 Hz, 1H), 8.09 (dd, *J* = 8.3, 1.4 Hz, 1H), 7.95 – 7.81 (m, 4H), 7.79 (ddd, *J* = 8.4, 7.0, 1.3 Hz, 1H), 7.69 (ddd, *J* = 8.3, 7.0, 1.4 Hz, 1H), 7.62 (dddd, *J* = 14.2, 8.3, 7.0, 1.3 Hz, 2H), 7.55 – 7.43 (m, 6H), 3.84 – 3.50 (m, 2H), 3.44 – 2.92 (m, 2H). ^{13}C NMR (101 MHz, Chloroform-*d*) δ 159.5 (d, *J* = 14.0 Hz), 143.5, 133.5, 132.8, 132.5, 131.9 (d, *J* = 2.8 Hz), 131.0 (d, *J* = 9.4 Hz), 130.1 (d, *J* = 91.3 Hz), 128.8 (d, *J* = 11.7 Hz), 128.7, 127.6, 126.7, 125.9, 125.1, 123.9, 122.5, 122.1, 27.6 (d, *J* = 72.2 Hz), 27.1 (d, *J* = 1.9 Hz). HRMS (ESI) m/z calcd. For $[C_{27}H_{23}NOP]^+$: 408.1512, found : 408.1516.

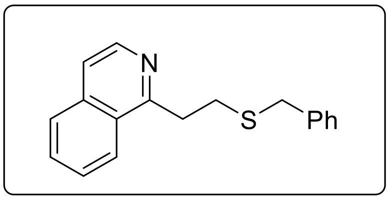

1-(2-(benzylthio)ethyl)isoquinoline (3s).

Prepared according to **GP1**. Purified by flash chromatography on silica gel (hexane/ethyl acetate = 20:1), compound **3s** (12.8 mg, 46%) was obtained. Pale yellow liquid. ^{1}H NMR (600 MHz, Chloroform-*d*) δ 8.44 (d, *J* = 5.7 Hz, 1H), 8.00 (dq, *J* = 8.5, 1.0 Hz, 1H), 7.81 (dd, *J* = 8.2, 1.1 Hz, 1H), 7.66 (ddd, *J* = 8.1, 6.8, 1.1 Hz, 1H), 7.56 (ddd, *J* = 8.3, 6.9, 1.3 Hz, 1H), 7.52 (d, *J* = 5.7 Hz, 1H), 7.35 – 7.33 (m, 2H), 7.31 (dd, *J* = 8.5, 6.7 Hz, 2H), 7.26 – 7.20 (m, 1H), 3.79 (s, 2H), 3.65 – 3.48 (m, 2H), 3.29 – 2.96 (m, 2H). ^{13}C NMR (101 MHz, Chloroform-*d*) δ 160.0, 141.9, 138.6, 136.3, 130.1, 129.0, 128.6, 127.5, 127.3, 127.10, 127.07, 125.1, 119.8, 36.8, 35.4, 30.7. HRMS (ESI) m/z calcd. For $[C_{18}H_{18}NS]^+$: 280.1154, found : 280.1161.

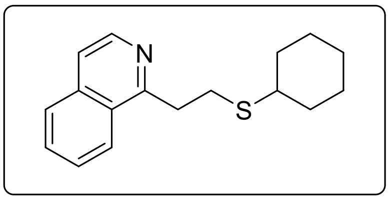

1-(2-(cyclohexylthio)ethyl)isoquinoline (3t).

Prepared according to **GP1**. Purified by flash chromatography on silica gel (hexane/ethyl acetate = 20:1), compound **3t** (13.1 mg, 48%) was obtained. Pale yellow liquid. ^{1}H NMR (600 MHz, Chloroform-*d*) δ 8.45 (d, *J* = 5.7 Hz, 1H), 8.16 (dd, *J* = 8.5, 1.1 Hz, 1H), 7.96 – 7.79 (m, 1H), 7.68 (ddd, *J* = 8.2, 6.8, 1.2 Hz, 1H), 7.61 (ddd, *J* = 8.3, 6.9, 1.3 Hz, 1H), 7.53 (d, *J* = 5.7 Hz, 1H), 3.66 – 3.47 (m, 2H), 3.32 – 2.96 (m, 2H), 2.73 (tt, *J* = 10.7, 3.7 Hz, 1H), 2.14 – 1.89 (m, 2H), 1.76 (dt, *J* = 12.4, 4.1 Hz, 2H), 1.70 – 1.57 (m, 1H), 1.50 – 1.12 (m, 5H). ^{13}C NMR (101 MHz, Chloroform-*d*) δ 160.2, 141.9, 136.4, 130.1, 127.6, 127.4, 127.1, 125.1, 119.8, 44.0, 36.0, 33.9, 29.3, 26.3, 26.0. HRMS (ESI) m/z calcd. For $[C_{17}H_{22}NS]^+$: 272.1467, found : 272.1474.

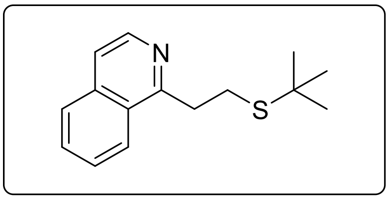

1-(2-(tert-butylthio)ethyl)isoquinoline (3u).

Prepared according to **GP1**. Purified by flash chromatography on silica gel (hexane/ethyl acetate = 20:1), compound **3u** (11.8 mg, 48%) was obtained. Pale yellow liquid. ^{1}H NMR (600 MHz, Chloroform-*d*) δ 8.46 (d, *J* = 5.7 Hz, 1H), 8.16 (dt, *J* = 8.5, 1.0 Hz, 1H), 7.82 (dt, *J* = 8.2, 0.9 Hz, 1H), 7.68 (ddd, *J* = 8.1, 6.8, 1.2 Hz, 1H), 7.61 (ddd, *J* = 8.3, 6.8, 1.3 Hz, 1H), 7.53 (d, *J* = 5.8 Hz, 1H), 3.61 – 3.46 (m, 2H), 3.39 – 3.06 (m, 2H), 1.36 (s, 9H). ^{13}C NMR (101 MHz, Chloroform-*d*) δ 160.3, 141.9, 136.4, 130.1, 127.6, 127.4, 127.1, 125.1, 119.8, 42.5, 35.5, 31.2, 27.6. HRMS (ESI) m/z calcd. For $[C_{15}H_{20}NS]^+$: 246.1311, found : 246.1316.

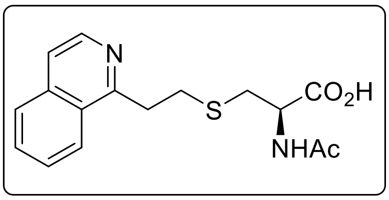

N-acetyl-S-(2-(isoquinolin-1-yl)ethyl)-L-cysteine (3v).

Prepared according to **GP1**. Purified by HPLC (water/methanol = 1:4), compound **3v** (15.2 mg, 48%) was obtained. Colorless liquid. ^{1}H NMR (600 MHz, Chloroform-*d*) δ 8.35 (d, *J* = 6.0 Hz, 1H), 8.22 (d, *J* = 8.5 Hz, 1H), 7.91 (d, *J* = 8.2 Hz, 1H), 7.86 – 7.78 (m, 2H), 7.77 – 7.69 (m, 2H), 5.01 (ddd, *J* = 8.9, 5.9, 3.5 Hz, 1H), 4.02 – 3.66 (m, 1H), 3.56 (td, *J* = 12.6, 5.3 Hz, 1H), 3.39 (td, *J* = 11.8, 5.3 Hz, 1H), 3.29 (dd, *J* = 14.5, 5.9 Hz, 1H), 3.20 (dd, *J* = 14.5, 3.6 Hz, 1H), 2.98 (td, *J* = 11.9, 5.0 Hz, 1H), 1.98 (s, 3H). ^{13}C NMR (101 MHz, Chloroform-*d*) δ 175.0, 170.8, 159.5, 137.7, 137.3, 132.2, 128.9, 127.9, 127.0, 125.8, 121.6, 54.3, 34.6, 33.9, 33.2, 23.1. HRMS (ESI) m/z calcd. For $[C_{16}H_{19}N_2O_3S]^+$: 319.1111, found : 319.1117.

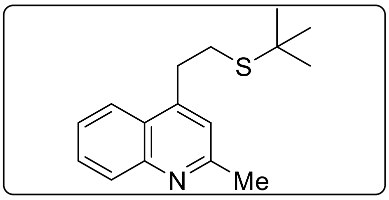

4-(2-(tert-butylthio)ethyl)-2-methylquinoline (3w).

Prepared according to **GP1**. Purified by flash chromatography on silica gel (hexane/ethyl acetate = 10:1), compound **3w** (11.9 mg, 46%) was obtained. Colorless liquid. ^{1}H NMR (600 MHz, Chloroform-*d*) δ 8.05 (dd, *J* = 8.3, 1.3 Hz, 1H), 7.97 (dd, *J* = 8.4, 1.4 Hz, 1H), 7.68 (ddd, *J* = 8.4, 6.8, 1.3 Hz, 1H), 7.52 (ddd, *J* = 8.2, 6.8, 1.3 Hz, 1H), 7.18 (s, 1H), 3.34 – 3.15 (m, 2H), 2.95 – 2.84 (m, 2H), 2.73 (s, 3H), 1.34 (s, 9H). ^{13}C NMR (101 MHz, Chloroform-*d*) δ 158.8, 148.1, 146.8, 129.5, 129.4, 125.9, 125.6, 123.2, 122.0, 42.7, 32.8, 31.1, 28.5, 25.4. HRMS (ESI) m/z calcd. For $[C_{16}H_{22}NS]^+$: 260.1467, found : 260.1473.

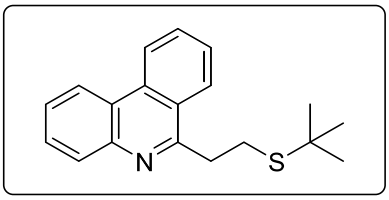

6-(2-(tert-butylthio)ethyl)phenanthridine (3x).

Prepared according to **GP1**. Purified by flash chromatography on silica gel (hexane/ethyl acetate = 20:1), compound **3x** (15.3 mg, 52%) was obtained. Colorless liquid. ^{1}H NMR (600 MHz, Chloroform-*d*) δ 8.65 (d, *J* = 8.4 Hz, 1H), 8.55 (dd, *J* = 8.1, 1.4 Hz, 1H), 8.25 (d, *J* = 8.2 Hz, 1H), 8.15 (d, *J* = 8.2

Hz, 1H), 7.85 (ddt, *J* = 8.3, 7.0, 1.2 Hz, 1H), 7.72 (dddd, *J* = 8.1, 7.3, 6.0, 1.2 Hz, 2H), 7.64 (ddd, *J* = 8.2, 7.2, 1.4 Hz, 1H), 3.73 – 3.48 (m, 2H), 3.42 – 3.04 (m, 2H), 1.39 (s, 9H). ^{13}C NMR (101 MHz, Chloroform-*d*) δ 160.3, 143.7, 133.1, 130.6, 129.8, 128.8, 127.6, 126.7, 126.0, 125.3, 123.8, 122.7, 122.1, 42.6, 36.1, 31.2, 27.2. HRMS (ESI) m/z calcd. For $[C_{19}H_{22}NS]^+$: 296.1467, found : 296.1472.

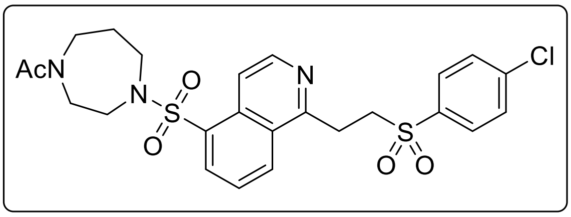

1-(4-((1-(2-((4-chlorophenyl)sulfonyl)ethyl)isoquinolin-5-yl)sulfonyl)-1,4-diazepan-1-yl)ethan-1-one (3y).

Prepared according to **GP1**. Purified by flash chromatography on silica gel (dichloromethane/methanol = 10:1), compound **3y** (40.2 mg, 75%) was obtained. Yellow liquid. ^{1}H NMR (400 MHz, Chloroform-*d*) δ 8.50 – 8.40 (m, 2H), 8.34 (ddd, *J* = 7.5, 2.9, 1.1 Hz, 2H), 7.93 – 7.86 (m, 2H), 7.75 (ddd, *J* = 8.6, 7.4, 1.3 Hz, 1H), 7.57 – 7.50 (m, 2H), 3.87 (s, 4H), 3.75 – 3.57 (m, 4H), 3.52 – 3.35 (m, 4H), 2.06 (d, *J* = 5.3 Hz, 3H), 1.98 (q, *J* = 5.9 Hz, 2H). ^{13}C NMR (101 MHz, Chloroform-*d*) δ 170.4, 170.2, 157.5, 157.4, 140.9, 137.7, 135.4, 133.2, 133.1, 132.3, 130.3, 129.9, 129.7, 127.6, 126.6, 117.2, 54.31, 54.28, 50.9, 50.2, 49.3, 48.5, 48.0, 47.8, 47.0, 44.6, 29.1, 27.8, 27.2, 21.7, 21.2. HRMS (ESI) m/z calcd. For $[C_{24}H_{27}ClN_3O_5S_2]^+$: 536.1075, found : 536.1082.

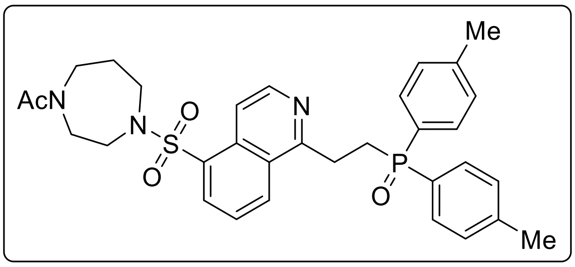

1-(4-((1-(2-(di-p-tolylphosphoryl)ethyl)isoquinolin-5-yl)sulfonyl)-1,4-diazepan-1-yl)ethan-1-one (3z).

Prepared according to **GP1**. Purified by flash chromatography on silica gel (dichloromethane/methanol = 20:1), compound **3z** (29.4 mg, 50%) was obtained. Colorless liquid. ^{1}H NMR (400 MHz, Chloroform-*d*) δ 8.53 (dd, *J* = 6.1, 3.3 Hz, 1H), 8.44 (dd, *J* = 8.5, 5.5 Hz, 1H), 8.35 – 8.18 (m, 2H), 7.74 – 7.56 (m, 5H), 7.25 (dd, *J* = 8.0, 2.7 Hz, 4H), 3.81 – 3.53 (m, 6H), 3.53 – 3.29 (m, 4H), 3.21 – 2.83 (m, 2H), 2.37 (s, 6H), 2.04 (s, 3H), 1.95 (p, *J* = 6.4 Hz, 2H). ^{13}C NMR (101 MHz, $CDCl_3$) δ 170.3, 170.1, 161.05 (d, *J* = 14.0 Hz), 160.99 (d, *J* = 14.0 Hz), 143.7, 142.44, 142.42, 142.39, 134.8, 134.7, 132.8, 132.7, 132.0, 131.0, 130.9 (d, *J* = 9.7 Hz), 130.6 (d, *J* = 92.5 Hz), 129.6 (d, *J* = 12.1 Hz), 129.1, 127.6, 127.6, 125.9, 116.3, 116.2, 50.9, 50.1, 49.3, 48.4, 48.0, 47.7, 46.9, 44.5, 29.0, 28.4 (d, *J* = 71.7 Hz), 28.3 (d, *J* = 71.6

Hz), 27.8, 27.1 (d, *J* = 1.6 Hz), 21.7, 21.2. HRMS (ESI) m/z calcd. For $[C_{32}H_{37}N_3O_4PS]^+$: 590.2237, found : 590.2241.

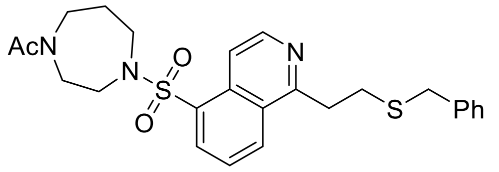

1-(4-((1-(2-(benzylthio)ethyl)isoquinolin-5-yl)sulfonyl)-1,4-diazepan-1-yl)ethan-1-one (3aa).

Prepared according to **GP1**. Purified by flash chromatography on silica gel (dichloromethane/methanol = 20:1), compound **3aa** (19.3 mg, 40%) was obtained. Colorless liquid. ^{1}H NMR (600 MHz, Chloroform-*d*) δ 8.58 (t, *J* = 5.6 Hz, 1H), 8.29 (ddd, *J* = 10.9, 7.4, 1.1 Hz, 1H), 8.25 (d, *J* = 6.1 Hz, 1H), 8.22 (dd, *J* = 8.3, 6.9 Hz, 1H), 7.61 (ddd, *J* = 8.5, 7.4, 4.9 Hz, 1H), 7.36 – 7.32 (m, 2H), 7.31 (dd, *J* = 8.5, 6.6 Hz, 2H), 7.26 – 7.22 (m, 1H), 3.79 (s, 2H), 3.74 – 3.69 (m, 1H), 3.66 – 3.57 (m, 3H), 3.54 (ddd, *J* = 8.1, 6.7, 1.8 Hz, 2H), 3.51 – 3.46 (m, 1H), 3.46 – 3.33 (m, 3H), 2.99 (ddd, *J* = 9.2, 6.5, 1.5 Hz, 2H), 2.05 (d, *J* = 5.4 Hz, 3H), 2.02 – 1.88 (m, 2H). ^{13}C NMR (101 MHz, Chloroform-*d*) δ 170.4, 170.2, 161.05, 160.99, 144.11, 144.08, 138.4, 134.8, 132.7, 132.1, 130.94, 130.90, 129.0, 128.7, 127.78, 127.75, 127.2, 125.7, 116.2, 116.1, 50.9, 50.2, 49.3, 48.4, 48.0, 47.8, 46.9, 44.6, 36.9, 35.78, 35.75, 30.6, 29.0, 27.7, 21.7, 21.2. HRMS (ESI) m/z calcd. For $[C_{25}H_{30}N_3O_3S_2]^+$: 484.1723, found : 484.1729.

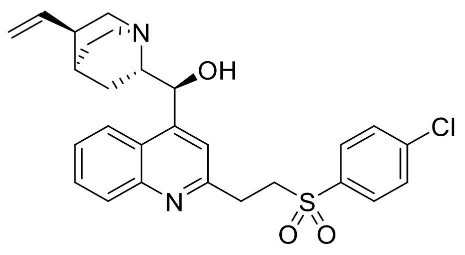

(S)-(2-(2-((4-chlorophenyl)sulfonyl)ethyl)quinolin-4-yl)((1S,2S,4S,5R)-5-vinylquinuclidin-2-yl)methanol (3ab).

Prepared according to **GP1**. Purified by HPLC (water/methanol = 1:4), compound **3ab** (20.8 mg, 42%) was obtained. White solid. ^{1}H NMR (400 MHz, Chloroform-*d*) δ 7.91 (ddd, *J* = 16.1, 8.5, 1.3 Hz, 2H), 7.84 – 7.75 (m, 2H), 7.63 (ddd, *J* = 8.4, 6.8, 1.3 Hz, 1H), 7.54 – 7.35 (m, 4H), 5.99 (ddd, *J* = 17.4, 10.5, 7.4 Hz, 1H), 5.69 (d, *J* = 4.7 Hz, 1H), 5.31 – 4.96 (m, 1H), 5.04 (dt, *J* = 11.0, 1.5 Hz, 1H), 3.88 – 3.69 (m, 2H), 3.48 – 3.33 (m, 2H), 3.33 – 3.25 (m, 1H), 3.09 (td, *J* = 9.3, 4.6 Hz, 1H), 3.00 – 2.85 (m, 2H), 2.77 (dt, *J* = 13.3, 8.8 Hz, 1H), 2.26 (q, *J* = 8.5 Hz, 1H), 2.12 – 1.86 (m, 1H), 1.79 (s, 1H), 1.63 – 1.42 (m, 2H), 1.33 – 1.08 (m, 1H). ^{13}C NMR (101 MHz, Chloroform-*d*) δ 156.9, 149.5, 148.0, 140.5, 140.3, 137.8, 129.83, 129.76, 129.6, 129.4, 126.6, 124.6, 123.0, 118.7, 115.1, 71.7, 60.3, 54.9, 50.1, 49.6, 39.9, 31.7, 28.2, 26.3, 21.5. HRMS (ESI) m/z calcd. For $[C_{27}H_{30}ClN_2O_3S]^+$: 497.1660, found : 497.1667.

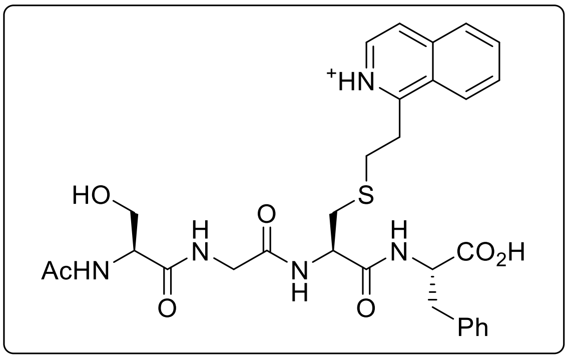

N-acetyl-L-serylglycyl-S-(2-(isoquinolin-1-yl)ethyl)-L-cysteinyl-L-phenylalanine (3ac).

Prepared according to **GP1**. Purified by HPLC (water/methanol = 1:2 with TFA 0.1%), compound **3ac** (40% NMRY) was obtained. Colorless liquid. ^{1}H NMR (600 MHz, Methanol-*d*$_4$) δ 8.67 (d, *J* = 8.6 Hz, 1H), 8.47 (d, *J* = 6.6 Hz, 1H), 8.35 (d, *J* = 6.6 Hz, 1H), 8.29 (d, *J* = 8.2 Hz, 1H), 8.22 (dd, *J* = 8.3, 7.1 Hz, 1H), 8.08 (ddd, *J* = 8.4, 6.9, 1.2 Hz, 1H), 7.27 – 7.22 (m, 2H), 7.22 – 7.19 (m, 2H), 7.17 (td, *J* = 6.9, 1.6 Hz, 1H), 4.64 (dd, *J* = 8.6, 5.1 Hz, 1H), 4.57 (dd, *J* = 8.5, 5.4 Hz, 1H), 4.34 (t, *J* = 5.2 Hz, 1H), 3.92 (d, *J* = 16.9 Hz, 1H), 3.89 – 3.81 (m, 4H), 3.78 (dd, *J* = 11.1, 5.3 Hz, 1H), 3.19 (dd, *J* = 14.0, 5.2 Hz, 1H), 3.16 – 3.04 (m, 2H), 3.04 – 2.91 (m, 2H), 2.78 (dd, *J* = 14.2, 8.5 Hz, 1H), 2.01 (s, 3H). ^{13}C NMR (101 MHz, Methanol-*d*$_4$) δ 174.2, 173.9, 173.7, 172.1, 171.7, 160.3, 140.3, 138.2, 137.7, 132.5, 131.7, 130.4, 129.6, 129.5, 128.9, 127.8, 127.7, 125.8, 62.8, 57.5, 55.2, 54.1, 43.8, 38.2, 34.7, 32.9, 32.2, 22.6. HRMS (ESI) m/z calcd. For $[C_{30}H_{36}N_5O_7S]^+$: 610.2330, found : 610.2335.

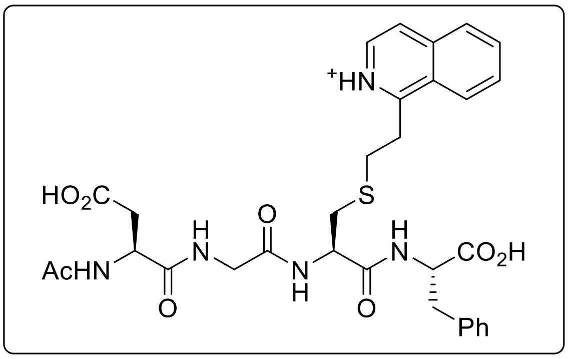

(2S,5R,11S)-2-benzyl-11-(carboxymethyl)-5-(((2-(isoquinolin-1-yl)ethyl)thio)methyl)-4,7,10,13-tetraoxo-3,6,9,12-tetraazatetradecanoic acid (3ad).

Prepared according to **GP1**. Purified by HPLC (water/methanol = 1:2 with TFA 0.1%), compound **3ad** (41% NMRY) was obtained. White solid. ^{1}H NMR (600 MHz, Methanol-*d*$_4$) δ 8.68 (d, *J* = 8.6 Hz, 1H), 8.47 (d, *J* = 6.5 Hz, 1H), 8.36 (d, *J* = 6.6 Hz, 1H), 8.30 (d, *J* = 8.2 Hz, 1H), 8.22 (dd, *J* = 8.2, 7.0 Hz, 1H), 8.08 (ddd, *J* = 8.4, 6.9, 1.2 Hz, 1H), 7.27 – 7.23 (m, 2H), 7.22 – 7.19 (m, 2H), 7.18 (td, *J* = 6.9, 1.6 Hz, 1H), 4.73 – 4.61 (m, 2H), 4.58 (dd, *J* = 8.8, 5.2 Hz, 1H), 3.94 (d, *J* = 17.0 Hz, 1H), 3.85 (dq, *J* = 12.8, 7.3 Hz, 2H), 3.75 (d, *J* = 16.9 Hz, 1H), 3.20 (dd, *J* = 14.0, 5.2 Hz, 1H), 3.11 (hept, *J* = 6.7 Hz, 2H), 2.99 (ddd, *J* = 14.2, 13.2, 6.9 Hz, 2H), 2.89 – 2.78 (m, 2H), 2.75 (dd, *J* = 17.1, 6.8 Hz, 1H), 1.99

(s, 3H). ^{13}C NMR (101 MHz, Methanol-d_4) δ 175.14, 175.11, 174.9, 174.7, 173.0, 172.6, 161.3, 141.3, 139.1, 138.6, 133.5, 132.6, 131.3, 130.5, 130.4, 129.8, 128.7, 128.6, 126.7, 56.1, 55.0, 52.5, 44.8, 39.2, 37.4, 35.6, 33.9, 33.1, 23.5. HRMS (ESI) m/z calcd. For $[C_{31}H_{36}N_5O_8S]^+$: 638.2279, found : 638.2288.

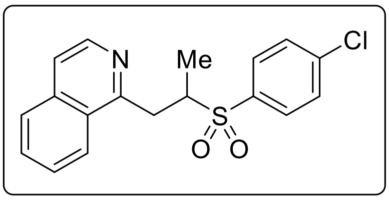

1-(2-((4-chlorophenyl)sulfonyl)propyl)isoquinoline (3ae).

Prepared according to **GP1**. Purified by flash chromatography on silica gel (hexane/ethyl acetate = 4:1), compound **3ae** (14.8 mg, 43%) was obtained. Colorless liquid. ^{1}H NMR (500 MHz, Chloroform-*d*) δ 8.35 (d, *J* = 5.7 Hz, 1H), 8.10 (dt, *J* = 8.6, 1.0 Hz, 1H), 7.98 – 7.84 (m, 2H), 7.84 – 7.78 (m, 1H), 7.69 (ddd, *J* = 8.1, 6.8, 1.2 Hz, 1H), 7.62 (ddd, *J* = 8.3, 6.9, 1.3 Hz, 1H), 7.52 (d, *J* = 5.7 Hz, 1H), 7.50 – 7.46 (m, 2H), 4.13 – 3.90 (m, 2H), 3.46 – 3.17 (m, 1H), 1.32 (d, *J* = 6.9 Hz, 3H). ^{13}C NMR (101 MHz, Chloroform-*d*) δ 156.4, 141.7, 140.6, 136.3, 135.9, 130.6, 130.3, 129.5, 127.8, 127.7, 127.3, 124.6, 120.1, 59.2, 33.9, 13.8. HRMS (ESI) m/z calcd. For $[C_{18}H_{17}ClNO_2S]^+$: 346.0663, found : 346.0669.

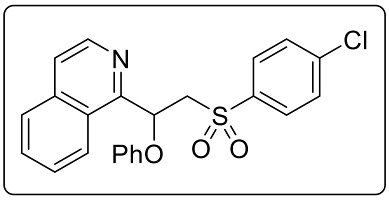

1-(2-((4-chlorophenyl)sulfonyl)-1-phenoxyethyl)isoquinoline (3a’).

Prepared according to **GP1**. Purified by flash chromatography on silica gel (hexane/ethyl acetate = 4:1), compound **3a’** (4.2 mg, 10%) was obtained. Colorless liquid. ^{1}H NMR (600 MHz, Chloroform-*d*) δ 8.41 (d, *J* = 5.6 Hz, 1H), 8.33 (d, *J* = 8.5 Hz, 1H), 7.86 (d, *J* = 8.2 Hz, 1H), 7.76 (d, *J* = 8.4 Hz, 2H), 7.72 (t, *J* = 7.5 Hz, 1H), 7.66 (dd, *J* = 8.8, 6.6 Hz, 1H), 7.59 (d, *J* = 5.6 Hz, 1H), 7.51 – 7.32 (m, 2H), 7.15 (t, *J* = 7.9 Hz, 2H), 6.90 (t, *J* = 7.3 Hz, 1H), 6.69 (d, *J* = 8.1 Hz, 2H), 6.54 (dd, *J* = 8.0, 4.0 Hz, 1H), 4.15 (dd, *J* = 15.0, 8.0 Hz, 1H), 4.01 (dd, *J* = 15.0, 4.0 Hz, 1H). ^{13}C NMR (101 MHz, Chloroform-*d*) δ 156.6, 155.0, 142.1, 140.5, 138.4, 136.9, 130.7, 129.9, 129.7, 129.4, 128.4, 128.1, 125.9, 123.7, 122.0, 121.7, 115.5, 73.6, 60.2. HRMS (ESI) m/z calcd. For $[C_{23}H_{19}ClNO_3S]^+$: 424.0769, found : 424.0774.

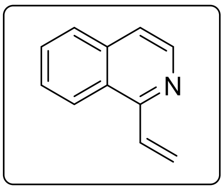

1-vinylisoquinoline (4a).

Prepared according to **GP2**. Purified by flash chromatography on silica gel (hexane/ethyl acetate = 20:1), compound **4a** (11.8 mg, 76%) was obtained. Yellow liquid. ^{1}H NMR (400 MHz, Chloroform-*d*) δ 8.51 (d, *J* = 5.6 Hz, 1H), 8.19 (dd, *J* = 8.6, 1.2 Hz, 1H), 7.74 (dd, *J* = 8.1, 1.3 Hz, 1H), 7.64 – 7.57 (m, 2H), 7.54 – 7.49 (m, 2H), 6.53 (dd, *J* = 17.0, 2.1 Hz, 1H), 5.69 (dd, *J* = 10.8, 2.1 Hz, 1H). ^{13}C NMR (101 MHz, Chloroform-*d*) δ 154.8, 142.3, 136.6, 132.2, 129.8, 127.19, 127.17, 126.4, 124.6, 121.7, 120.3. HRMS (ESI) m/z calcd. For $[C_{11}H_{10}N]^+$: 156.0808, found : 156.0814.

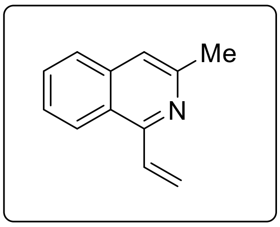

3-methyl-1-vinylisoquinoline (4b).

Prepared according to **GP2**. Purified by flash chromatography on silica gel (hexane/ethyl acetate = 10:1), compound **4b** (11.3 mg, 67%) was obtained. Colorless liquid. ^{1}H NMR (400 MHz, Chloroform-*d*) δ 8.20 (dq, *J* = 8.5, 1.0 Hz, 1H), 7.72 (dt, *J* = 8.2, 1.0 Hz, 1H), 7.65 – 7.55 (m, 2H), 7.50 (ddd, *J* = 8.3, 6.8, 1.4 Hz, 1H), 7.41 (s, 1H), 6.50 (dd, *J* = 17.0, 2.1 Hz, 1H), 5.71 (dd, *J* = 10.8, 2.1 Hz, 1H), 2.70 (s, 3H). ^{13}C NMR (101 MHz, Chloroform-*d*) δ 154.6, 151.1, 137.5, 132.6, 129.9, 126.7, 126.3, 124.8, 124.7, 121.8, 118.6, 24.6. HRMS (ESI) m/z calcd. For $[C_{12}H_{12}N]^+$: 170.0964, found : 170.0970.

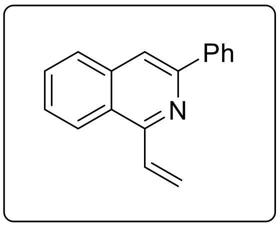

3-phenyl-1-vinylisoquinoline (4c).

Prepared according to **GP2**. Purified by flash chromatography on silica gel (hexane/ethyl acetate = 30:1), compound **4c** (18.0 mg, 78%) was obtained. Colorless liquid. ^{1}H NMR (600 MHz, Chloroform-*d*) δ 8.29 – 8.26 (m, 1H), 8.24 (dt, *J* = 6.6, 1.3 Hz, 2H), 8.06 – 7.98 (m, 1H), 7.87 (d, *J* = 8.2 Hz, 1H), 7.74 – 7.64 (m, 2H), 7.57 (ddd, *J* = 8.3, 6.9, 1.5 Hz, 1H), 7.52 (dd, *J* = 8.5, 7.0 Hz, 2H), 7.43 (tt, *J* = 7.0, 1.2 Hz, 1H), 6.78 (dd, *J* = 16.9, 2.2 Hz, 1H), 5.77 (dd, *J* = 10.7, 2.1 Hz, 1H). ^{13}C NMR (101 MHz, Chloroform-*d*) δ 154.3, 150.1, 139.8, 137.8, 132.1, 130.1, 128.8, 128.6, 127.8, 127.13, 127.10, 125.6, 124.7, 122.1, 116.3. HRMS (ESI) m/z calcd. For $[C_{17}H_{14}N]^+$: 232.1121, found : 232.1124.

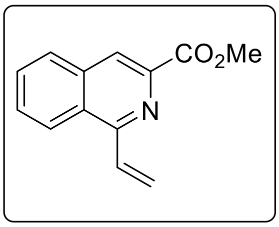

methyl 1-vinylisoquinoline-3-carboxylate (4d).

Prepared according to **GP2**. Purified by flash chromatography on silica gel (hexane/ethyl acetate = 10:1), compound **4d** (12.3 mg, 58%) was obtained. Yellow liquid. ^{1}H NMR (600 MHz, Chloroform-*d*) δ 8.50 (s, 1H), 8.42 – 8.29 (m, 1H), 7.97 (dd, *J* = 7.6, 1.6 Hz, 1H), 7.86 – 7.68 (m, 2H), 7.60 (dd, *J* = 17.0, 10.8 Hz, 1H), 6.62 (dd, *J* = 17.0, 1.7 Hz, 1H), 5.82 (dd, *J* = 10.8, 1.7 Hz, 1H), 4.05 (s, 3H). ^{13}C NMR (101 MHz, Chloroform-*d*) δ 166.7, 155.7, 141.0, 136.6, 131.8, 130.8, 129.6, 128.9, 127.9, 125.2, 123.7, 123.6, 53.0. HRMS (ESI) m/z calcd. For $[C_{13}H_{12}NO_2]^+$: 214.0863, found : 214.0868.

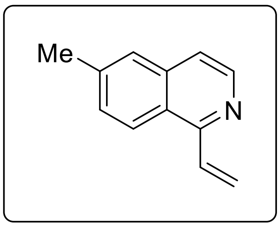

6-methyl-1-vinylisoquinoline (4e).

Prepared according to **GP2**. Purified by flash chromatography on silica gel (hexane/ethyl acetate = 10:1), compound **4e** (12.8 mg, 76%) was obtained. Pale yellow liquid. ^{1}H NMR (600 MHz, Chloroform-*d*) δ 8.48 (d, *J* = 5.6 Hz, 1H), 8.14 (d, *J* = 8.7 Hz, 1H), 7.84 – 7.54 (m, 2H), 7.48 (d, *J* = 5.6 Hz, 1H), 7.42 (dd, *J* = 8.7, 1.8 Hz, 1H), 6.51 (dd, *J* = 17.0, 1.9 Hz, 1H), 5.70 (dd, *J* = 10.8, 1.9 Hz, 1H), 2.53 (s, 3H). ^{13}C NMR (101 MHz, Chloroform-*d*) δ 154.6, 142.5, 140.3, 137.1, 132.4, 129.6, 126.2, 125.0, 124.6, 121.6, 120.0, 22.0. HRMS (ESI) m/z calcd. For $[C_{12}H_{12}N]^+$: 170.0964, found : 170.0968.

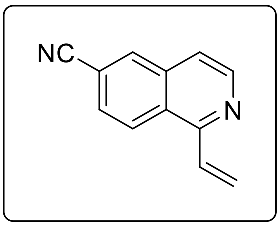

1-vinylisoquinoline-6-carbonitrile (4f).

Prepared according to **GP2** (**1**, 0.3 mmol). Purified by flash chromatography on silica gel (hexane/ethyl acetate = 20:1), compound **4f** (24.5 mg, 46%) was obtained. White solid. ^{1}H NMR (400 MHz, Chloroform-*d*) δ 8.66 (d, *J* = 5.6 Hz, 1H), 8.35 (d, *J* = 8.8 Hz, 1H), 8.21 (d, *J* = 1.6 Hz, 1H), 7.74 (dd, *J* = 8.8, 1.7 Hz, 1H), 7.65 – 7.53 (m, 2H), 6.58 (dd, *J* = 16.9, 1.8 Hz, 1H), 5.80 (dd, *J* = 10.8, 1.8 Hz,

1H). ^{13}C NMR (101 MHz, Chloroform-*d*) δ 155.5, 144.3, 135.7, 133.4, 131.3, 127.9, 127.0, 126.3, 123.5, 120.1, 118.3, 113.8. HRMS (ESI) m/z calcd. For $[C_{12}H_9N_2]^+$: 181.0760, found : 181.0768.

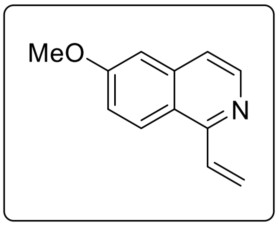

6-methoxy-1-vinylisoquinoline (4g).

Prepared according to **GP2**. Purified by flash chromatography on silica gel (hexane/ethyl acetate = 20:1), compound **4g** (11.2 mg, 61%) was obtained. Pale yellow solid. ^{1}H NMR (600 MHz, Chloroform-*d*) δ 8.45 (d, *J* = 5.7 Hz, 1H), 8.16 (d, *J* = 9.2 Hz, 1H), 7.55 (dd, *J* = 17.0, 10.8 Hz, 1H), 7.48 (d, *J* = 5.7 Hz, 1H), 7.23 (dd, *J* = 9.3, 2.6 Hz, 1H), 7.06 (d, *J* = 2.6 Hz, 1H), 6.51 (dd, *J* = 17.0, 1.9 Hz, 1H), 5.71 (dd, *J* = 10.8, 1.9 Hz, 1H), 3.95 (s, 3H). ^{13}C NMR (101 MHz, Chloroform-*d*) δ 160.7, 154.3, 142.9, 138.9, 132.3, 126.7, 122.3, 121.9, 120.3, 119.8, 104.7, 55.6. HRMS (ESI) m/z calcd. For $[C_{12}H_{12}NO]^+$: 186.0913, found : 186.0918.

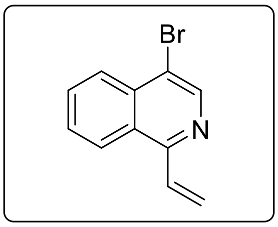

4-bromo-1-vinylisoquinoline (4h).

Prepared according to **GP2**. Purified by flash chromatography on silica gel (hexane/ethyl acetate = 20:1), compound **4h** (17.3 mg, 74%) was obtained. Colorless liquid. ^{1}H NMR (400 MHz, Chloroform-*d*) δ 8.71 (s, 1H), 8.25 (d, *J* = 8.5 Hz, 1H), 8.19 (d, *J* = 8.5 Hz, 1H), 7.79 (ddd, *J* = 8.3, 6.9, 1.2 Hz, 1H), 7.67 (ddd, *J* = 8.4, 6.9, 1.2 Hz, 1H), 7.56 (dd, *J* = 16.9, 10.8 Hz, 1H), 6.53 (dd, *J* = 16.9, 1.9 Hz, 1H), 5.75 (dd, *J* = 10.8, 1.9 Hz, 1H). ^{13}C NMR (101 MHz, Chloroform-*d*) δ 154.5, 144.3, 135.3, 131.7, 131.3, 128.2, 127.7, 126.7, 125.2, 122.7, 119.0. HRMS (ESI) m/z calcd. For $[C_{11}H_9BrN]^+$: 233.9913, found : 233.9917.

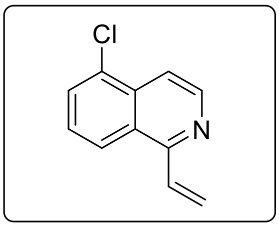

5-chloro-1-vinylisoquinoline (4i).

Prepared according to **GP2**. Purified by flash chromatography on silica gel (hexane/ethyl acetate = 20:1), compound **4i** (15.5 mg, 82%) was obtained. White solid. ^{1}H NMR (400 MHz, Chloroform-*d*) δ 8.63 (d, *J* = 5.8 Hz, 1H), 8.19 (dt, *J* = 8.6, 1.0 Hz, 1H), 7.98 (dd, *J* = 5.9, 1.0 Hz, 1H), 7.76 (dd, *J* = 7.5, 1.0 Hz, 1H), 7.59 (dd, *J* = 16.9, 10.8 Hz, 1H), 7.51 (dd, *J* = 8.5, 7.5 Hz, 1H), 6.55 (dd, *J* = 16.9, 1.9 Hz, 1H), 5.75 (dd, *J* = 10.8, 1.9 Hz, 1H). ^{13}C NMR (101 MHz, Chloroform-*d*) δ 155.3, 143.6, 134.7, 132.1, 131.8, 130.0, 127.4, 127.0, 123.9, 122.8, 116.7. HRMS (ESI) m/z calcd. For $[C_{11}H_{9}ClN]^{+}$: 290.0418, found : 190.0425.

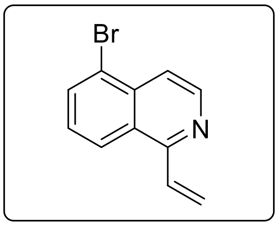

5-bromo-1-vinylisoquinoline (4j).

Prepared according to **GP2**. Purified by flash chromatography on silica gel (hexane/ethyl acetate = 20:1), compound **4j** (16.8 mg, 72%) was obtained. White solid. ^{1}H NMR (600 MHz, Chloroform-*d*) δ 8.63 (dd, *J* = 5.9, 1.2 Hz, 1H), 8.24 (dd, *J* = 8.4, 1.3 Hz, 1H), 7.96 (t, *J* = 6.3 Hz, 2H), 7.60 (ddd, *J* = 16.9, 10.8, 1.2 Hz, 1H), 7.45 (ddd, *J* = 8.5, 7.2, 1.3 Hz, 1H), 6.55 (dt, *J* = 16.9, 1.6 Hz, 1H), 5.76 (dt, *J* = 10.7, 1.6 Hz, 1H). ^{13}C NMR (101 MHz, Chloroform-*d*) δ 155.4, 143.9, 135.9, 133.9, 132.0, 127.60, 127.56, 124.6, 122.8, 122.4, 119.3. HRMS (ESI) m/z calcd. For $[C_{11}H_{9}BrN]^{+}$: 233.9913, found : 233.9916.

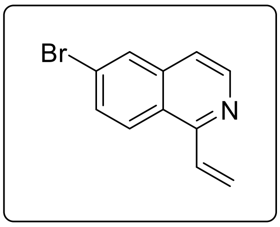

6-bromo-1-vinylisoquinoline (4k).

Prepared according to **GP2**. Purified by flash chromatography on silica gel (hexane/ethyl acetate = 20:1), compound **4k** (17.8 mg, 76%) was obtained. Colorless liquid. ^{1}H NMR (400 MHz, Chloroform-*d*) δ 8.54 (d, *J* = 5.7 Hz, 1H), 8.11 (d, *J* = 9.0 Hz, 1H), 7.98 (d, *J* = 2.0 Hz, 1H), 7.66 (dd, *J* = 9.0, 2.1 Hz, 1H), 7.54 (dd, *J* = 16.9, 10.8 Hz, 1H), 7.47 (dd, *J* = 5.7, 0.9 Hz, 1H), 6.53 (dd, *J* = 16.9, 1.9 Hz, 1H), 5.74 (dd, *J* = 10.8, 1.9 Hz, 1H). ^{13}C NMR (101 MHz, Chloroform-*d*) δ 155.2, 143.6, 137.9, 131.9, 130.9, 129.4, 126.6, 124.9, 124.8, 122.6, 119.3. HRMS (ESI) m/z calcd. For $[C_{11}H_{9}BrN]^{+}$: 233.9913, found : 233.9919.

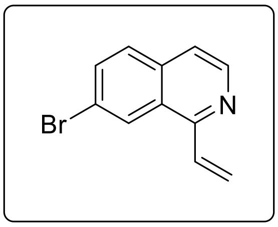

7-bromo-1-vinylisoquinoline (4l).

Prepared according to **GP2**. Purified by flash chromatography on silica gel (hexane/ethyl acetate = 20:1), compound **4l** (18.0 mg, 77%) was obtained. Pale yellow liquid. ^{1}H NMR (400 MHz, Chloroform-*d*) δ 8.54 (d, *J* = 5.6 Hz, 1H), 8.47 – 8.29 (m, 1H), 7.73 (dd, *J* = 8.7, 1.8 Hz, 1H), 7.68 (d, *J* = 8.7 Hz, 1H), 7.58 – 7.43 (m, 2H), 6.52 (dd, *J* = 16.9, 1.9 Hz, 1H), 5.74 (dd, *J* = 10.8, 1.9 Hz, 1H). ^{13}C NMR (101 MHz, Chloroform-*d*) δ 154.2, 143.0, 135.2, 133.5, 131.8, 129.0, 127.4, 127.3, 122.6, 121.2, 120.1. HRMS (ESI) m/z calcd. For $[C_{11}H_{9}BrN]^{+}$: 233.9913, found : 233.9919.

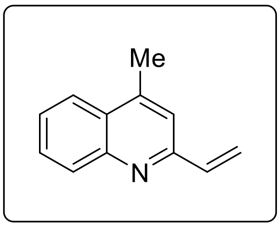

4-methyl-2-vinylquinoline (4m).

Prepared according to **GP2**. Purified by flash chromatography on silica gel (hexane/ethyl acetate = 20:1), compound **4m** (10.8 mg, 64%) was obtained. Colorless liquid. ^{1}H NMR (400 MHz, Chloroform-*d*) δ 8.07 (dt, *J* = 8.4, 0.9 Hz, 1H), 7.95 (dd, *J* = 8.3, 1.4 Hz, 1H), 7.68 (ddd, *J* = 8.4, 6.8, 1.5 Hz, 1H), 7.52 (ddd, *J* = 8.3, 6.9, 1.3 Hz, 1H), 7.45 (d, *J* = 1.2 Hz, 1H), 7.00 (dd, *J* = 17.7, 10.9 Hz, 1H), 6.26 (dd, *J* = 17.7, 0.9 Hz, 1H), 5.64 (dd, *J* = 10.9, 0.9 Hz, 1H), 2.71 (d, *J* = 1.0 Hz, 3H). ^{13}C NMR (101 MHz, Chloroform-*d*) δ 155.8, 147.9, 144.6, 138.2, 130.0, 129.5, 127.7, 126.2, 123.8, 119.7, 119.2, 19.0. HRMS (ESI) m/z calcd. For $[C_{12}H_{12}N]^{+}$: 170.0964, found : 170.0972.

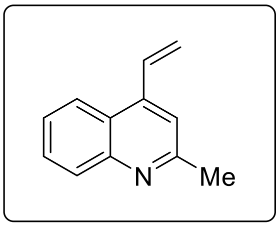

2-methyl-4-vinylquinoline (4n).

Prepared according to **GP2**. Purified by flash chromatography on silica gel (hexane/ethyl acetate = 20:1), compound **4n** (13.1 mg, 77%) was obtained. Colorless liquid. ^{1}H NMR (400 MHz, Chloroform-*d*) δ 8.15 – 7.94 (m, 2H), 7.68 (ddd, *J* = 8.5, 6.9, 1.4 Hz, 1H), 7.50 (ddd, *J* = 8.1, 6.8, 1.3 Hz, 1H), 7.46 – 7.34 (m, 2H), 5.96 (dd, *J* = 17.4, 1.3 Hz, 1H), 5.64 (dd, *J* = 11.1, 1.2 Hz, 1H), 2.75 (s, 3H). ^{13}C NMR

(101 MHz, Chloroform-*d*) δ 158.9, 148.3, 143.7, 132.4, 129.5, 129.2, 125.8, 124.7, 123.5, 120.6, 118.4, 25.5. HRMS (ESI) m/z calcd. For $[C_{12}H_{12}N]^+$: 170.0964, found : 170.0970.

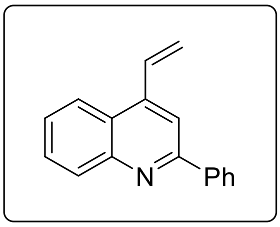

2-phenyl-4-vinylquinoline (4o).

Prepared according to **GP2**. Purified by flash chromatography on silica gel (hexane/ethyl acetate = 30:1), compound **4o** (14.1 mg, 61%) was obtained. Colorless liquid. ^{1}H NMR (400 MHz, Chloroform-*d*) δ 8.23 – 8.15 (m, 3H), 8.10 (dd, *J* = 8.5, 1.4 Hz, 1H), 7.95 (s, 1H), 7.73 (ddd, *J* = 8.4, 6.8, 1.4 Hz, 1H), 7.68 – 7.38 (m, 5H), 6.06 (dd, *J* = 17.3, 1.2 Hz, 1H), 5.71 (dd, *J* = 11.1, 1.2 Hz, 1H). ^{13}C NMR (101 MHz, Chloroform-*d*) δ 157.4, 148.8, 144.4, 140.0, 132.8, 130.4, 129.6, 129.4, 129.0, 127.7, 126.4, 125.3, 123.5, 120.7, 115.7. HRMS (ESI) m/z calcd. For $[C_{17}H_{14}N]^+$: 232.1121, found : 232.1126.

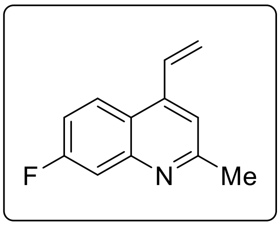

7-fluoro-2-methyl-4-vinylquinoline (4p).

Prepared according to **GP2**. Purified by flash chromatography on silica gel (hexane/ethyl acetate = 20:1), compound **4p** (13.8 mg, 74%) was obtained. White solid. ^{1}H NMR (600 MHz, Chloroform-*d*) δ 8.02 (dd, *J* = 9.2, 6.0 Hz, 1H), 7.65 (dd, *J* = 10.2, 2.6 Hz, 1H), 7.38 – 7.30 (m, 2H), 7.30 – 7.22 (m, 1H), 5.96 (dt, *J* = 17.4, 1.0 Hz, 1H), 5.66 (dt, *J* = 11.1, 1.0 Hz, 1H), 2.73 (s, 3H). ^{13}C NMR (101 MHz, Chloroform-*d*) δ 163.1 (d, *J* = 249.4 Hz), 160.3, 149.5 (d, *J* = 12.4 Hz), 143.8, 132.2, 125.7 (d, *J* = 9.9 Hz), 121.7, 121.1, 117.9 (d, *J* = 2.5 Hz), 116.0 (d, *J* = 24.9 Hz), 112.9 (d, *J* = 20.0 Hz), 25.5. ^{19}F NMR (376 MHz, Chloroform-*d*) δ -110.57. HRMS (ESI) m/z calcd. For $[C_{12}H_{11}FN]^+$: 188.0870, found : 188.0876.

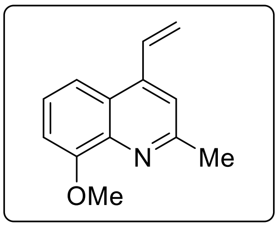

8-methoxy-2-methyl-4-vinylquinoline (4q).

Prepared according to **GP2**. Purified by flash chromatography on silica gel (hexane/ethyl acetate = 3:1), compound **4q** (15.5 mg, 78%) was obtained. White solid. ^{1}H NMR (400 MHz, Chloroform-*d*) δ 7.60 (dd, *J* = 8.6, 1.1 Hz, 1H), 7.49 – 7.20 (m, 3H), 7.03 (d, *J* = 7.7 Hz, 1H), 5.93 (dd, *J* = 17.4, 1.2 Hz, 1H), 5.61 (dd, *J* = 11.0, 1.3 Hz, 1H), 4.07 (s, 3H), 2.79 (s, 3H). ^{13}C NMR (101 MHz, Chloroform-*d*) δ 157.9, 155.2, 143.7, 140.2, 132.8, 125.7, 120.4, 119.1, 115.3, 107.6, 56.2, 25.8. HRMS (ESI) m/z calcd. For $[C_{13}H_{14}NO]^{+}$: 200.1070, found : 200.1075.

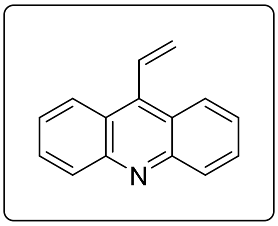

9-vinylacridine (4r).

Prepared according to **GP2**. Purified by flash chromatography on silica gel (hexane/ethyl acetate = 30:1), compound **4r** (15.8 mg, 77%) was obtained. Yellow solid. ^{1}H NMR (400 MHz, Chloroform-*d*) δ 8.29 (d, *J* = 8.7 Hz, 2H), 8.24 (d, *J* = 8.8 Hz, 2H), 7.78 (ddd, *J* = 8.6, 6.6, 1.4 Hz, 2H), 7.61 – 7.37 (m, 3H), 6.12 (dd, *J* = 11.7, 1.7 Hz, 1H), 5.74 (dd, *J* = 17.9, 1.7 Hz, 1H). ^{13}C NMR (126 MHz, Chloroform-*d*) δ 148.6, 144.3, 131.3, 130.3, 129.7, 126.0, 125.8, 125.2, 124.2. HRMS (ESI) m/z calcd. For $[C_{15}H_{12}N]^{+}$: 206.0964, found : 206.0968.

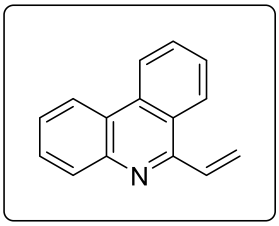

6-vinylphenanthridine (4s).

Prepared according to **GP2**. Purified by flash chromatography on silica gel (hexane/ethyl acetate = 20:1), compound **4s** (16.4 mg, 80%) was obtained. Colorless liquid. ^{1}H NMR (400 MHz, Chloroform-*d*) δ 8.76 – 8.61 (m, 1H), 8.56 (dd, *J* = 8.2, 1.4 Hz, 1H), 8.47 – 8.32 (m, 1H), 8.20 (d, *J* = 8.1 Hz, 1H), 7.86 (ddd, *J* = 8.3, 7.0, 1.3 Hz, 1H), 7.79 – 7.55 (m, 4H), 6.59 (dd, *J* = 16.9, 2.0 Hz, 1H), 5.82 (dd, *J* = 10.8, 2.0 Hz, 1H). ^{13}C NMR (101 MHz, Chloroform-*d*) δ 155.7, 144.0, 133.3, 133.1, 130.6, 130.4, 128.9, 127.5, 126.9, 126.1, 125.1, 124.2, 123.0, 122.5, 122.1. HRMS (ESI) m/z calcd. For $[C_{15}H_{12}N]^{+}$: 206.0964, found : 206.0967.

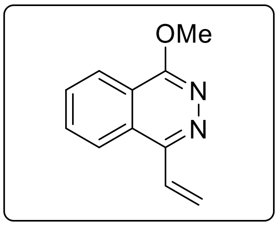

1-methoxy-4-vinylphthalazine (4t).

Prepared according to **GP2** (**1**, 0.3 mmol). Purified by flash chromatography on silica gel (hexane/ethyl acetate = 5:1), compound **4t** (22.1 mg, 40%) was obtained. White solid. ^{1}H NMR (400 MHz, Chloroform-*d*) δ 8.31 – 8.15 (m, 1H), 8.14 – 8.01 (m, 1H), 7.83 (tt, *J* = 7.2, 5.4 Hz, 2H), 7.36 (dd, *J* = 17.1, 10.9 Hz, 1H), 6.52 (dd, *J* = 17.1, 1.8 Hz, 1H), 5.69 (dd, *J* = 10.9, 1.8 Hz, 1H), 4.27 (s, 3H). ^{13}C NMR (101 MHz, Chloroform-*d*) δ 160.2, 151.6, 132.2, 131.6, 129.6, 127.4, 123.6, 123.4, 121.9, 119.8, 54.9. HRMS (ESI) m/z calcd. For $[C_{11}H_{11}N_2O]^+$: 187.0866, found : 187.0870.

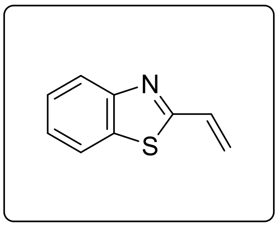

2-vinylbenzo[d]thiazole (4u).

Prepared according to **GP2** (**1**, 0.3 mmol). Purified by flash chromatography on silica gel (hexane/ethyl acetate = 20:1), compound **4u** (24.5 mg, 51%) was obtained. Pale yellow liquid. ^{1}H NMR (400 MHz, Chloroform-*d*) δ 8.00 (dt, *J* = 8.2, 0.9 Hz, 1H), 7.91 – 7.72 (m, 1H), 7.47 (ddd, *J* = 8.3, 7.2, 1.3 Hz, 1H), 7.38 (ddd, *J* = 8.2, 7.2, 1.2 Hz, 1H), 7.05 (dd, *J* = 17.5, 10.9 Hz, 1H), 6.19 (d, *J* = 17.5 Hz, 1H), 5.77 (d, *J* = 10.9 Hz, 1H). ^{13}C NMR (101 MHz, Chloroform-*d*) δ 167.3, 153.7, 134.4, 131.5, 126.4, 125.7, 123.40, 123.39, 121.7. HRMS (ESI) m/z calcd. For $[C_9H_8NS]^+$: 162.0372, found : 162.0378.

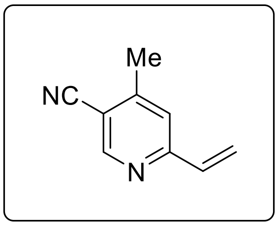

4-methyl-6-vinylnicotinonitrile (4v).

Prepared according to **GP2** (**1**, 0.3 mmol). Purified by flash chromatography on silica gel (hexane/ethyl acetate = 20:1), compound **4v** (19.3 mg, 45%) was obtained. Colorless liquid. ^{1}H NMR (400 MHz, Chloroform-*d*) δ 8.72 (s, 1H), 7.25 (s, 1H), 6.78 (dd, *J* = 17.4, 10.7 Hz, 1H), 6.36 (dd, *J* = 17.4, 1.1 Hz, 1H), 5.65 (dd, *J* = 10.6, 1.1 Hz, 1H), 2.54 (s, 3H). ^{13}C NMR (101 MHz, Chloroform-*d*) δ 158.5, 152.7,

151.3, 135.7, 122.4, 122.3, 116.5, 109.1, 20.3. HRMS (ESI) m/z calcd. For $[C_9H_9N_2]^+$: 145.0760, found : 145.0770.

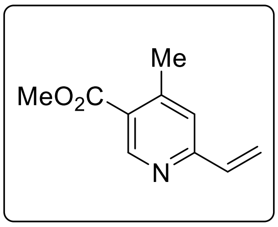

methyl 4-methyl-6-vinylnicotinate (4w).

Prepared according to **GP2** (**1**, 0.3 mmol). Purified by flash chromatography on silica gel (hexane/ethyl acetate = 20:1), compound **4w** (27.9 mg, 52%) was obtained. Colorless liquid. ^{1}H NMR (400 MHz, Chloroform-*d*) δ 9.04 (s, 1H), 7.19 (s, 1H), 6.80 (dd, *J* = 17.4, 10.8 Hz, 1H), 6.32 (dd, *J* = 17.5, 1.2 Hz, 1H), 5.59 (dd, *J* = 10.8, 1.2 Hz, 1H), 3.92 (s, 3H), 2.62 (s, 3H). ^{13}C NMR (101 MHz, Chloroform-*d*) δ 166.6, 158.0, 151.7, 150.4, 136.1, 124.3, 124.1, 120.9, 52.2, 21.6. HRMS (ESI) m/z calcd. For $[C_{10}H_{12}NO_2]^+$: 178.0863, found : 178.0871.

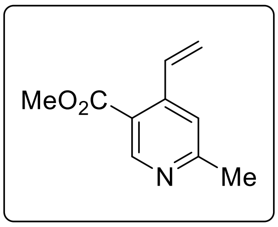

methyl 6-methyl-4-vinylnicotinate (4x-C4).

Prepared according to **GP2** (**1**, 0.3 mmol). Purified by flash chromatography on silica gel (hexane/ethyl acetate = 4:1), compound **4x-C4** (20.2 mg, 38%) was obtained. Colorless liquid. ^{1}H NMR (400 MHz, Chloroform-*d*) δ 8.98 (s, 1H), 7.50 (dd, *J* = 17.5, 11.0 Hz, 1H), 7.31 (s, 1H), 5.83 (dd, *J* = 17.5, 1.1 Hz, 1H), 5.52 (dd, *J* = 11.0, 1.1 Hz, 1H), 3.92 (s, 3H), 2.60 (s, 3H). ^{13}C NMR (101 MHz, Chloroform-*d*) δ 166.6, 162.3, 151.6, 147.4, 134.1, 121.3, 120.5, 120.0, 52.3, 24.7. HRMS (ESI) m/z calcd. For $[C_{10}H_{12}NO_2]^+$: 178.0863, found : 178.0866.

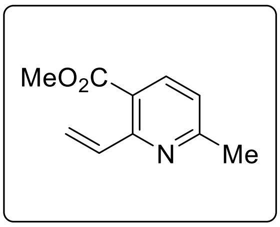

methyl 6-methyl-2-vinylnicotinate (4x-C6).

Prepared according to **GP2** (**1**, 0.3 mmol). Purified by flash chromatography on silica gel (hexane/ethyl acetate = 20:1), compound **4x-C6** (4.8 mg, 9%) was obtained. Colorless liquid. ^{1}H NMR (400 MHz, Chloroform-*d*) δ 8.06 (d, *J* = 8.1 Hz, 1H), 7.64 (dd, *J* = 17.0, 10.7 Hz, 1H), 7.09 (d, *J* = 8.1 Hz, 1H),

6.50 (dd, *J* = 17.0, 2.3 Hz, 1H), 5.57 (dd, *J* = 10.7, 2.3 Hz, 1H), 3.91 (s, 3H), 2.60 (s, 3H). ^{13}C NMR (101 MHz, Chloroform-*d*) δ 167.2, 161.8, 154.9, 138.9, 134.2, 121.8, 121.6, 121.4, 52.4, 25.0. HRMS (ESI) m/z calcd. For $[C_{10}H_{12}NO_2]^+$: 178.0863, found : 178.0871.

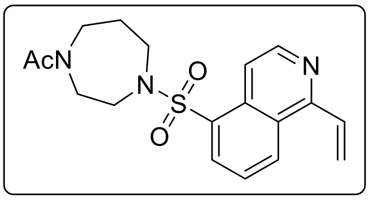

1-(4-((1-vinylisoquinolin-5-yl)sulfonyl)-1,4-diazepan-1-yl)ethan-1-one (4y).

Prepared according to **GP2**. Purified by flash chromatography on silica gel (hexane/ethyl acetate = 20:1), compound **4y** (24.4 mg, 68%) was obtained. Yellow liquid. ^{1}H NMR (400 MHz, Chloroform-*d*) δ 8.67 (dd, *J* = 6.0, 2.9 Hz, 1H), 8.56 – 8.48 (m, 1H), 8.31 (ddd, *J* = 7.4, 6.3, 1.2 Hz, 2H), 7.72 – 7.53 (m, 2H), 6.55 (dt, *J* = 16.8, 2.2 Hz, 1H), 5.79 (dt, *J* = 10.8, 2.0 Hz, 1H), 3.74 – 3.55 (m, 4H), 3.54 – 3.32 (m, 4H), 2.05 (d, *J* = 5.7 Hz, 3H), 1.97 (pd, *J* = 6.3, 3.4 Hz, 2H). ^{13}C NMR (101 MHz, Chloroform-*d*) δ 170.3, 170.1, 156.14, 156.10, 144.59, 144.57, 134.62, 134.60, 132.88, 132.86, 132.4, 132.0, 131.9, 130.8, 130.7, 127.1, 125.7, 123.7, 123.6, 116.84, 116.78, 51.0, 50.2, 49.3, 48.4, 48.0, 47.8, 46.9, 44.6, 29.1, 27.7, 21.7, 21.2. HRMS (ESI) m/z calcd. For $[C_{18}H_{22}N_3O_3S]^+$: 360.1376, found : 360.1380.

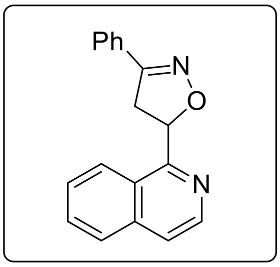

5-(isoquinolin-1-yl)-3-phenyl-4,5-dihydroisoxazole (5a).

Prepared according to **(II-iv-1)**. Purified by flash chromatography on silica gel (hexane/ethyl acetate = 10:1), compound **5a** (20.8 mg, 76%) was obtained. White solid. ^{1}H NMR (400 MHz, Chloroform-*d*) δ 8.47 (d, *J* = 5.7 Hz, 1H), 8.43 (dd, *J* = 8.5, 1.2 Hz, 1H), 7.98 – 7.84 (m, 1H), 7.83 – 7.78 (m, 2H), 7.71 (ddd, *J* = 8.2, 6.9, 1.4 Hz, 1H), 7.69 – 7.63 (m, 2H), 7.49 – 7.38 (m, 3H), 6.51 (dd, *J* = 11.0, 8.7 Hz, 1H), 4.66 (dd, *J* = 16.6, 8.7 Hz, 1H), 3.67 (dd, *J* = 16.6, 11.0 Hz, 1H). ^{13}C NMR (101 MHz, Chloroform-*d*) δ 157.5, 155.4, 141.3, 136.9, 130.3, 130.2, 129.7, 128.8, 127.8, 127.39, 127.37, 127.1, 125.6, 121.8, 81.5, 37.6. HRMS (ESI) m/z calcd. For $[C_{18}H_{15}N_2O]^+$: 275.1179, found : 275.1186.

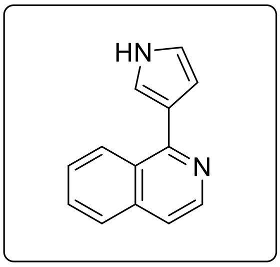

1-(1H-pyrrol-3-yl)isoquinoline (5b).

Prepared according to **(II-iv-2)**. Purified by flash chromatography on silica gel (hexane/ethyl acetate = 1:1), compound **5b** (16.9 mg, 87%) was obtained. Yellow liquid. ^{1}H NMR (400 MHz, Chloroform-*d*) δ 8.76 (br, 1H), 8.56 – 8.51 (m, 2H), 7.86 – 7.81 (m, 1H), 7.66 (ddd, *J* = 8.2, 6.8, 1.3 Hz, 1H), 7.56 (ddd, *J* = 8.3, 6.8, 1.3 Hz, 1H), 7.51 (dd, *J* = 5.7, 0.9 Hz, 1H), 7.35 (q, *J* = 2.1 Hz, 1H), 6.94 (q, *J* = 2.5 Hz, 1H), 6.79 (td, *J* = 2.7, 1.5 Hz, 1H). ^{13}C NMR (101 MHz, Chloroform-*d*) δ 156.4, 142.4, 137.1, 129.8, 127.8, 127.1, 127.0, 126.9, 123.8, 119.5, 118.6, 118.5, 110.5. HRMS (ESI) m/z calcd. For $[C_{13}H_{11}N_2]^+$: 195.0917, found : 195.0923.

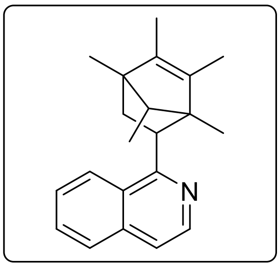

1-(1,4,5,6,7-pentamethylbicyclo[2.2.1]hept-5-en-2-yl)isoquinoline (5c).

Prepared according to **(II-iv-3)**. Purified by flash chromatography on silica gel (hexane/dichloromethane = 8:1), compound **5c** (29.1 mg, 100% (dr = 4.5:1)) was obtained. White solid. ^{1}H NMR (600 MHz, Chloroform-*d*) δ 8.42 – 8.28 (m, 2H), 7.77 (dd, *J* = 8.2, 1.3 Hz, 1H), 7.62 (ddd, *J* = 8.0, 6.7, 1.1 Hz, 1H), 7.55 (ddd, *J* = 8.3, 6.8, 1.4 Hz, 1H), 7.41 (d, *J* = 5.6 Hz, 1H), 4.16 (dd, *J* = 8.9, 4.5 Hz, 1H), 2.26 (dd, *J* = 11.0, 4.6 Hz, 1H), 1.87 (dd, *J* = 11.0, 8.9 Hz, 1H), 1.70 – 1.64 (m, 1H), 1.68 (s, 3H), 1.18 (s, 3H), 1.00 (s, 3H), 0.84 (s, 3H), 0.66 (d, *J* = 6.4 Hz, 3H). ^{13}C NMR (101 MHz, Chloroform-*d*) δ 162.2, 141.9, 136.0, 135.2, 130.9, 129.1, 127.3, 126.20, 126.18, 118.5, 62.7, 61.1, 53.3, 49.6, 41.3, 15.9, 15.7, 11.7, 10.2, 8.5. HRMS (ESI) m/z calcd. For $[C_{21}H_{26}N]^+$: 292.2060, found : 292.2065.

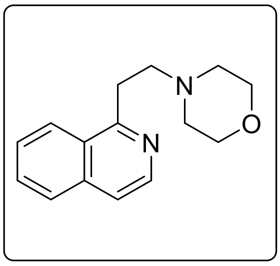

4-(2-(isoquinolin-1-yl)ethyl)morpholine (5d).

Prepared according to **(II-iv-4)**. Purified by flash chromatography on silica gel (dichloromethane/methanol = 10:1), compound **5d** (20.6 mg, 85%) was obtained. Pale yellow solid. ^{1}H NMR (400 MHz, Chloroform-*d*) δ 8.40 (d, *J* = 5.8 Hz, 1H), 8.13 (d, *J* = 8.4 Hz, 1H), 7.77 (d, *J* = 8.1 Hz, 1H), 7.65 – 7.60 (m, 1H), 7.56 (ddd, *J* = 8.3, 6.8, 1.4 Hz, 1H), 7.47 (d, *J* = 5.7 Hz, 1H), 3.76 – 3.69 (m, 4H), 3.51 – 3.45 (m, 2H), 2.93 – 2.85 (m, 2H), 2.58 (t, *J* = 4.7 Hz, 4H). ^{13}C NMR (101 MHz, Chloroform-*d*) δ 160.0, 141.9, 136.2, 129.9, 127.5, 127.2, 127.1, 125.1, 119.5, 67.1, 58.2, 53.8, 32.6. HRMS (ESI) m/z calcd. For $[C_{15}H_{19}N_2O]^+$: 243.1492, found : 243.1497.

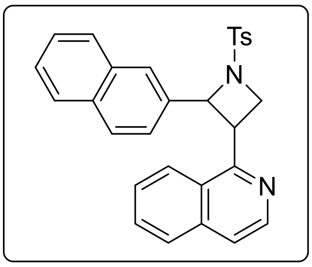

1-(2-(naphthalen-2-yl)-1-tosylazetidin-3-yl)isoquinoline (5e).

Prepared according to **(II-iv-5)**. Purified by flash chromatography on silica gel (hexane/ethyl acetate = 3:1), compound **5e** (27.9 mg, 74%) was obtained. Pale yellow solid. ^{1}H NMR (600 MHz, Chloroform-*d*) δ 8.41 (d, *J* = 5.6 Hz, 1H), 8.03 – 7.70 (m, 2H), 7.70 – 7.58 (m, 1H), 7.49 (s, 1H), 7.47 (dd, *J* = 7.9, 1.5 Hz, 1H), 7.34 – 7.28 (m, 2H), 7.27 – 7.19 (m, 7H), 7.15 (d, *J* = 8.5 Hz, 1H), 7.00 (dd, *J* = 8.5, 1.8 Hz, 1H), 5.76 (d, *J* = 9.7 Hz, 1H), 5.24 (dd, *J* = 7.9, 4.7 Hz, 1H), 4.82 (td, *J* = 9.2, 4.6 Hz, 1H), 4.31 (dd, *J* = 8.9, 7.9 Hz, 1H), 2.36 (s, 3H). ^{13}C NMR (101 MHz, Chloroform-*d*) δ 155.0, 144.0, 141.4, 135.6, 133.9, 133.4, 132.7, 132.4, 129.7, 129.6, 128.4, 127.9, 127.3, 127.2, 127.1, 126.8, 126.6, 125.7, 125.6, 125.1, 124.3, 120.1, 69.0, 49.2, 38.0, 21.6. HRMS (ESI) m/z calcd. For $[C_{29}H_{25}N_2O_2S]^+$: 465.1631, found : 465.1635.

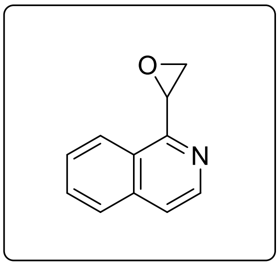

1-(oxiran-2-yl)isoquinoline (5f).

Prepared according to **(II-iv-6)**. Purified by flash chromatography on silica gel (hexane/ethyl acetate = 4:1), compound **5f** (14.0 mg, 82%) was obtained. Pale yellow liquid. ^{1}H NMR (400 MHz, Chloroform-*d*) δ 8.48 (d, *J* = 5.7 Hz, 1H), 8.37 (dq, *J* = 8.4, 1.0 Hz, 1H), 7.83 (dt, *J* = 8.2, 1.0 Hz, 1H), 7.69 (ddd, *J* = 8.2, 6.9, 1.3 Hz, 1H), 7.62 (ddd, *J* = 8.3, 6.9, 1.4 Hz, 1H), 7.58 (dd, *J* = 5.7, 1.0 Hz, 1H), 4.62 (dd, *J*

= 4.1, 2.6 Hz, 1H), 3.33 (dd, *J* = 6.0, 2.6 Hz, 1H), 3.28 (dd, *J* = 6.0, 4.1 Hz, 1H). ^{13}C NMR (101 MHz, Chloroform-*d*) δ 155.0, 142.2, 136.3, 130.3, 127.7, 127.6, 127.5, 124.2, 120.9, 51.0, 48.6. HRMS (ESI) m/z calcd. For $[C_{11}H_{10}NO]^+$: 172.0757, found : 172.0765.

Appendix I

Spectral Copies of ^{1}H-, ^{13}C-, ^{19}F-NMR Data Obtained in this Study

1-(2-((4-chlorophenyl)sulfonyl)ethyl) isoquinoline (3a).

400 MHz, ^{1}H NMR in Chloroform-*d*

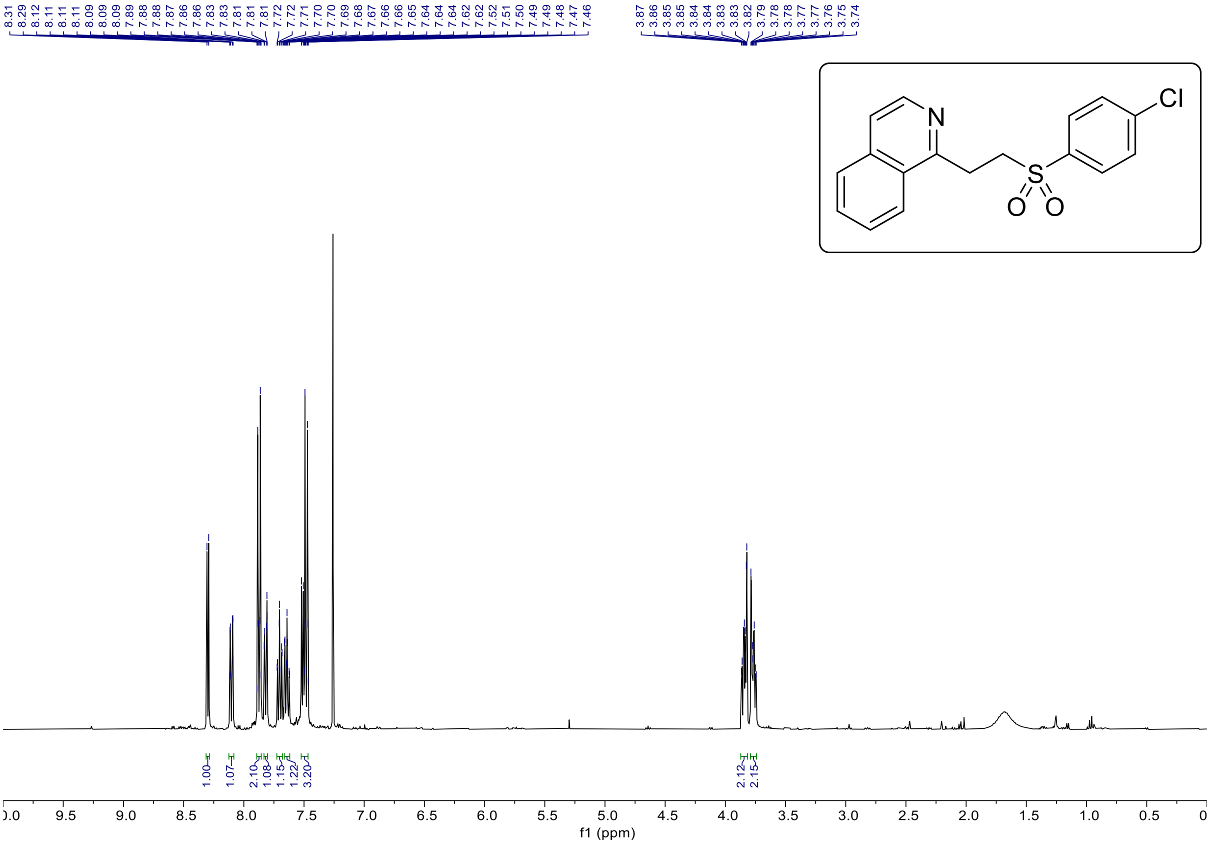

101 MHz, ^{13}C NMR in Chloroform-*d*

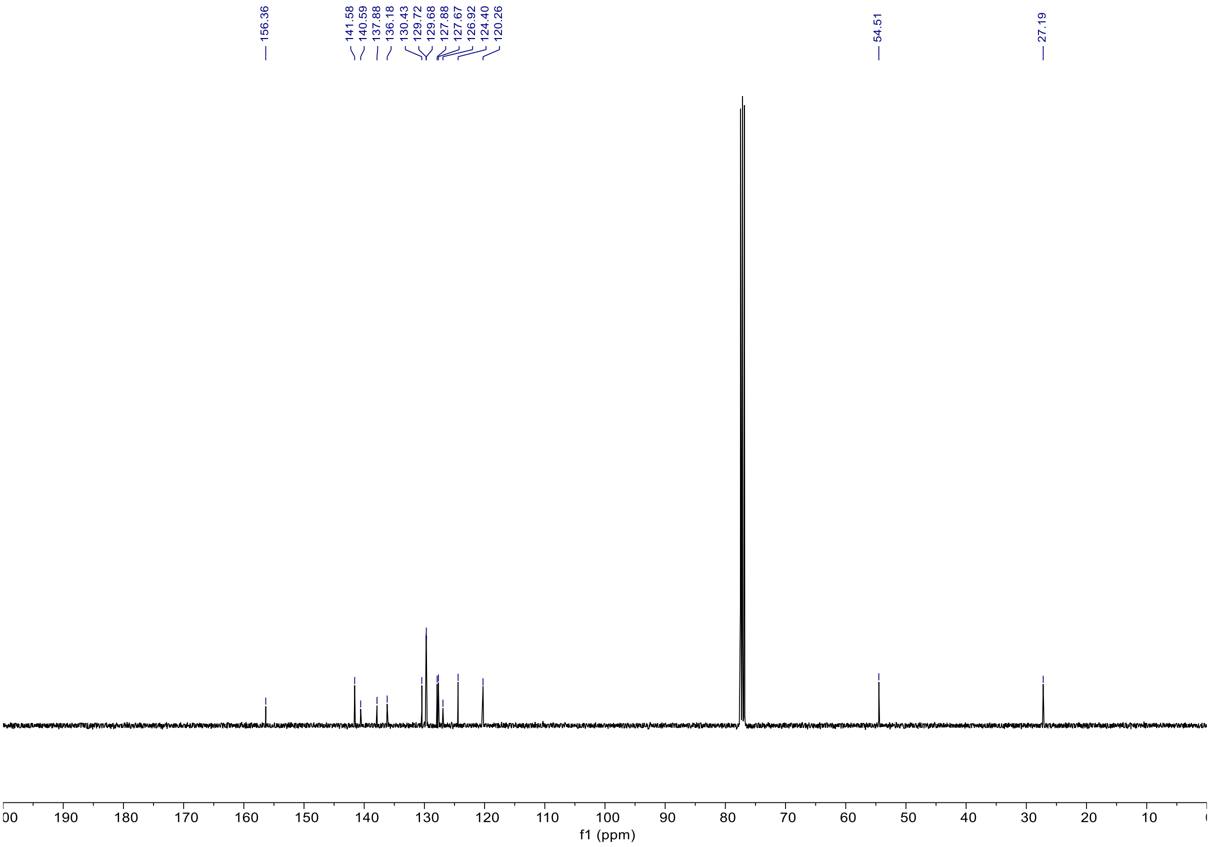

1-(2-(phenylsulfonyl)ethyl)isoquinoline (3b).

400 MHz, ^{1}H NMR in Chloroform-*d*

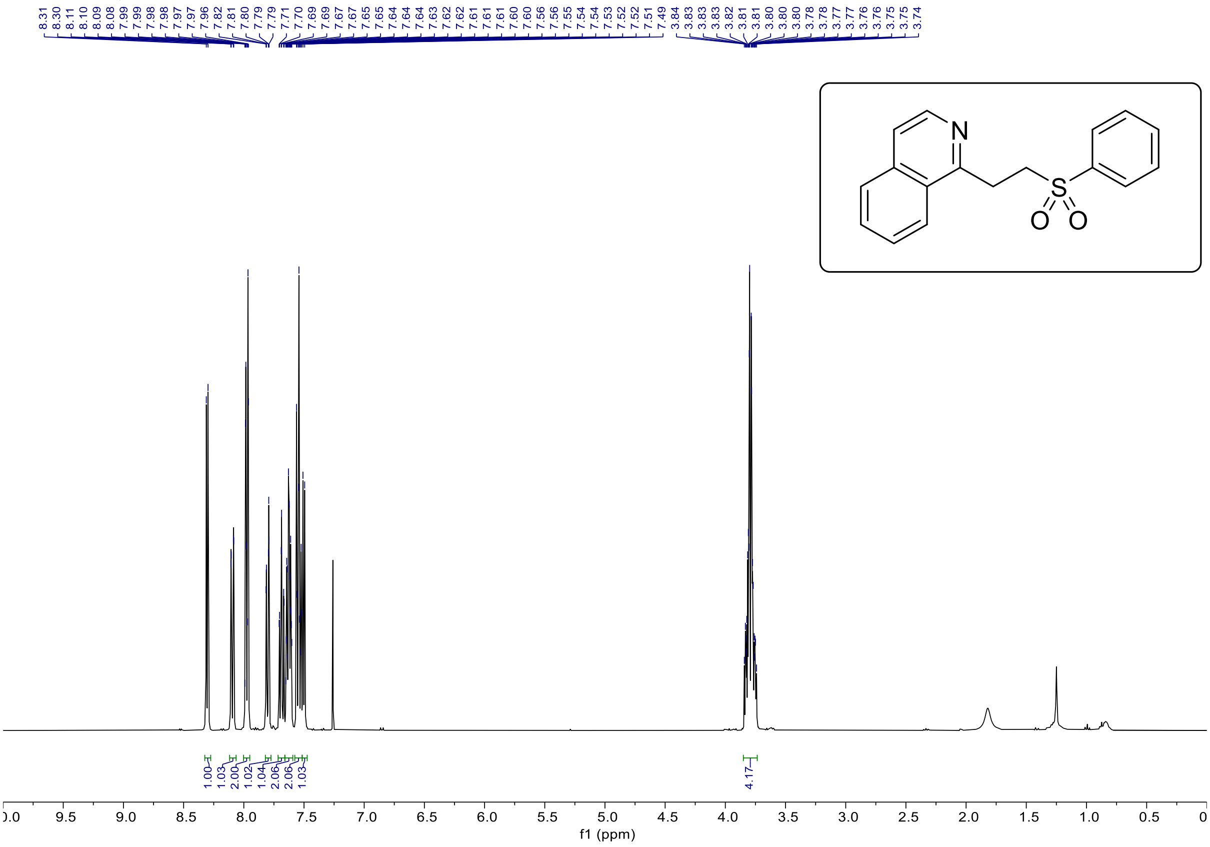

101 MHz, ^{13}C NMR in Chloroform-*d*

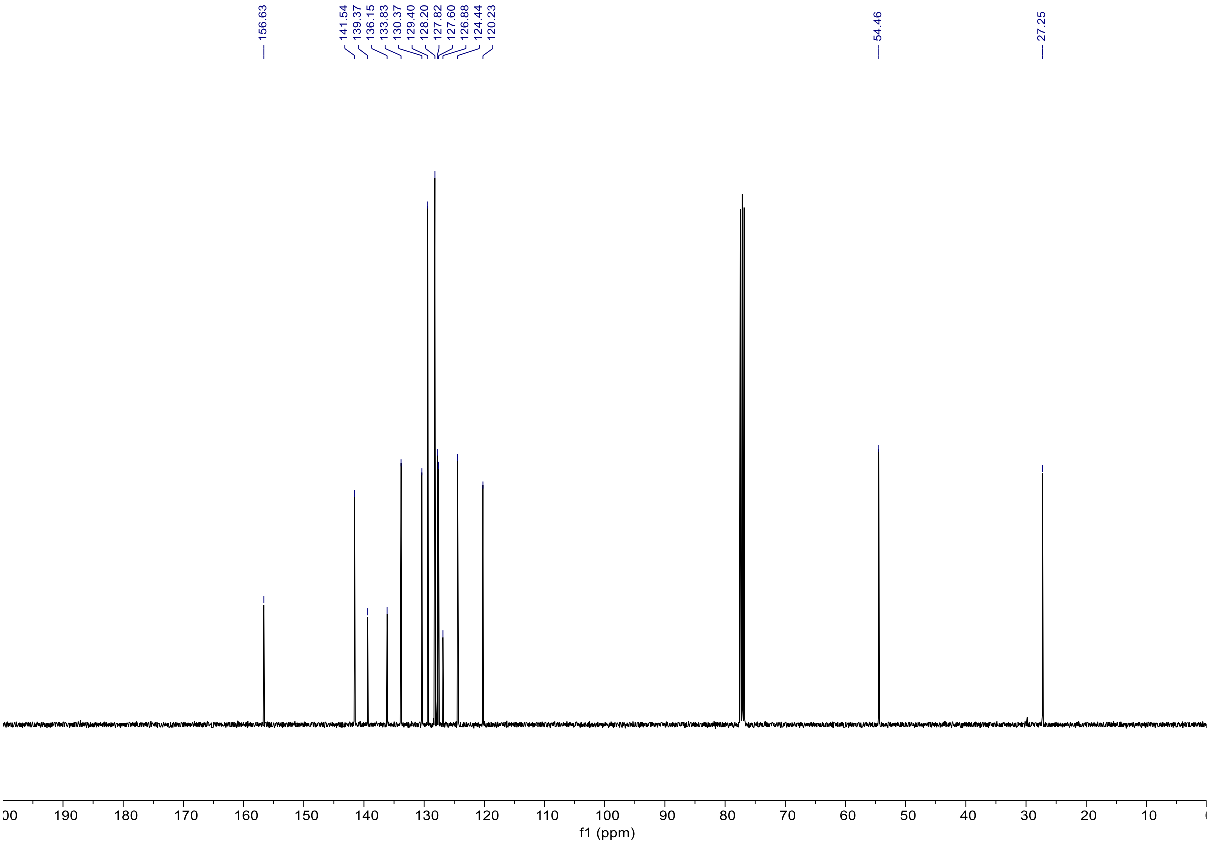

1-(2-tosylethyl)isoquinoline (3c).

400 MHz, ^{1}H NMR in Chloroform-*d*

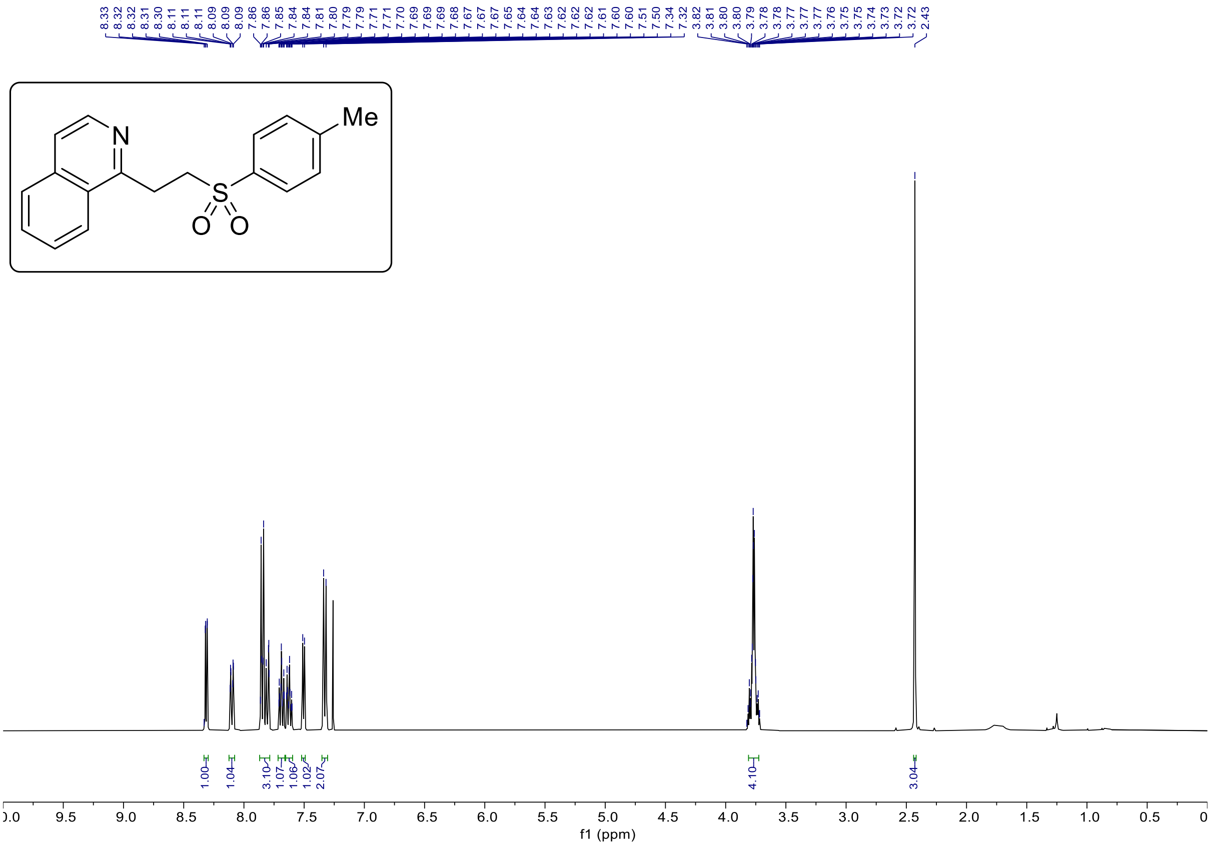

101 MHz, ^{13}C NMR in Chloroform-*d*

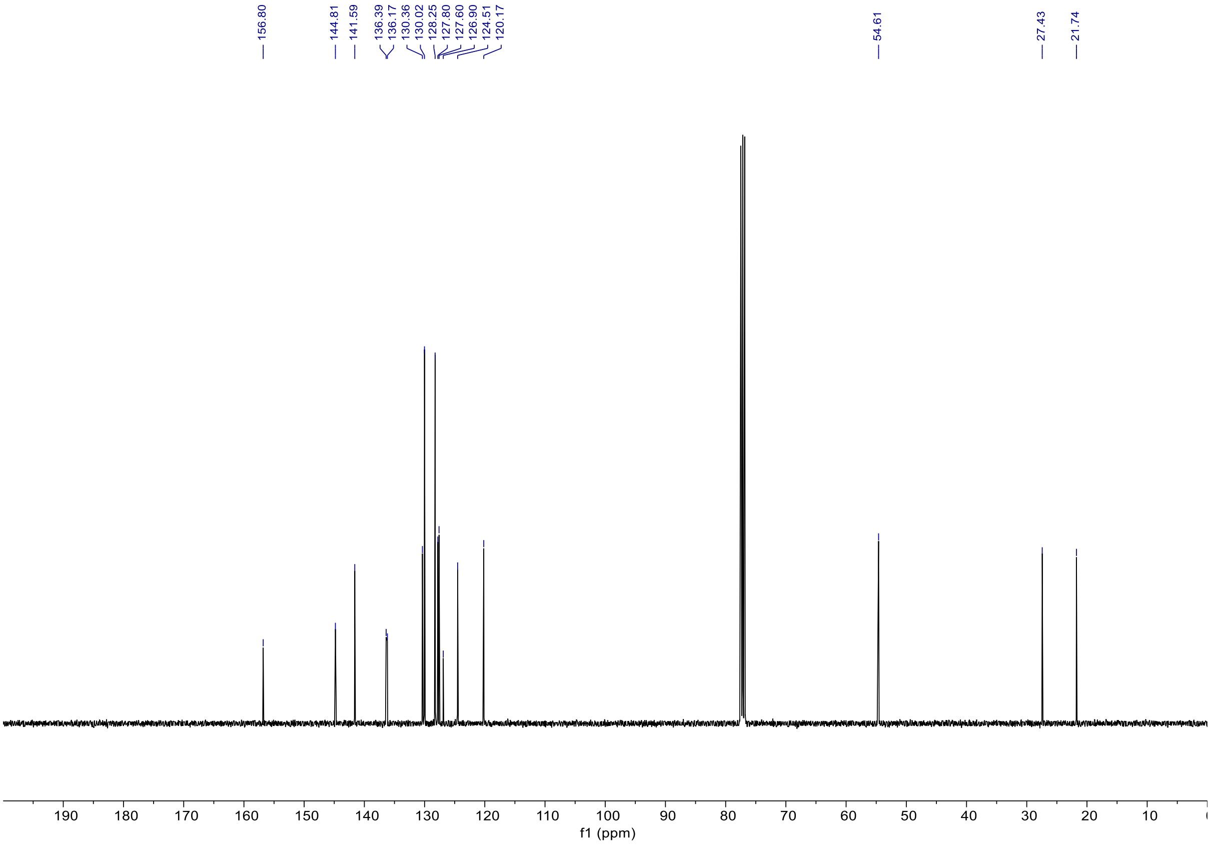

1-(2-((4-(trifluoromethyl)phenyl)sulfonyl)ethyl)isoquinoline (3d).

600 MHz, ^{1}H NMR in Chloroform-*d*

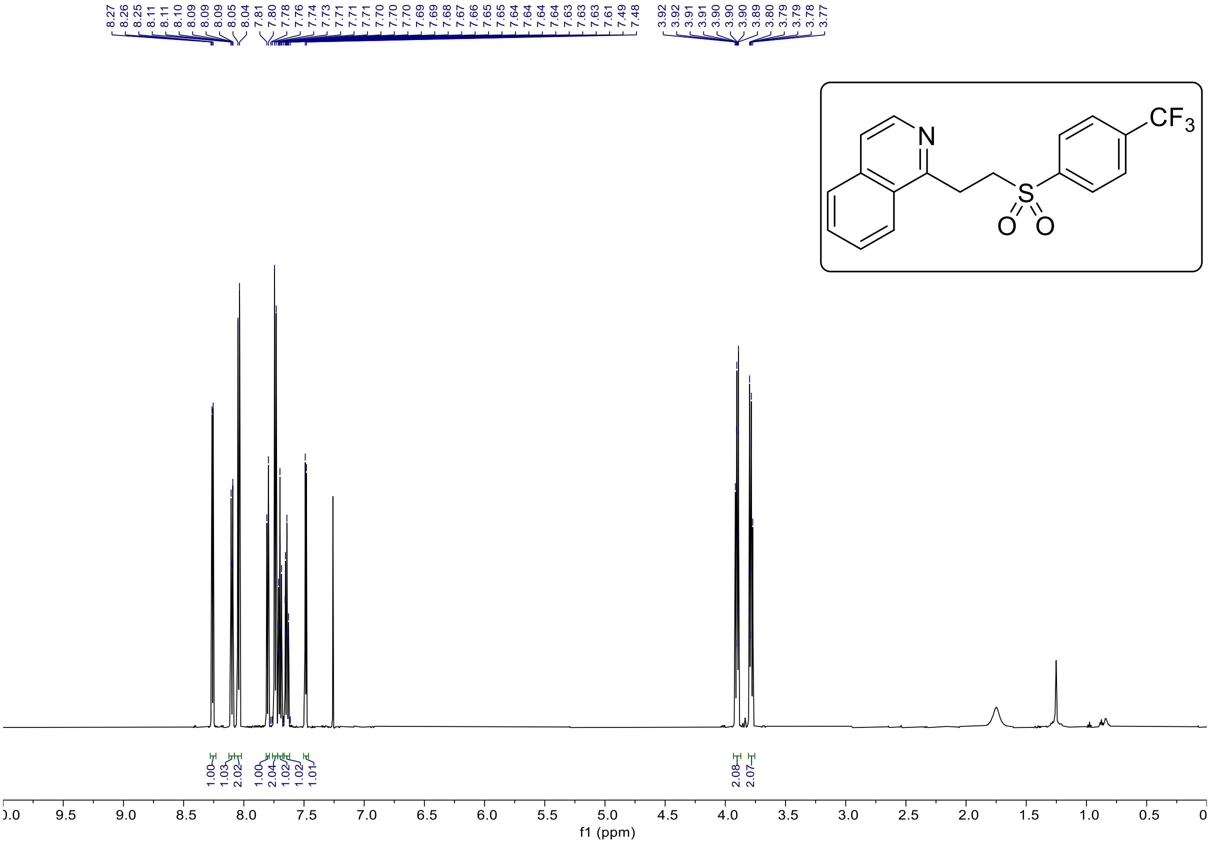

101 MHz, ^{13}C NMR in Chloroform-*d*

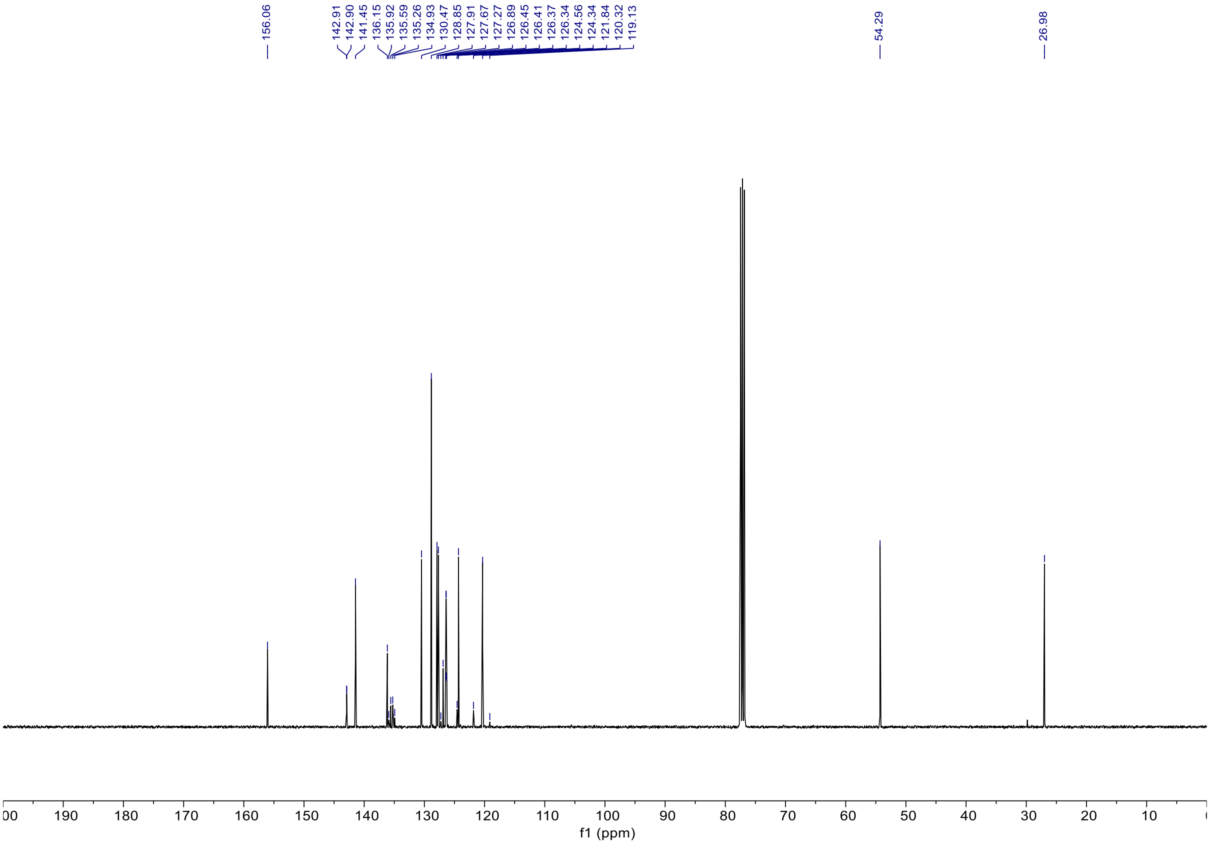

376 MHz, ^{19}F NMR in Chloroform-*d*

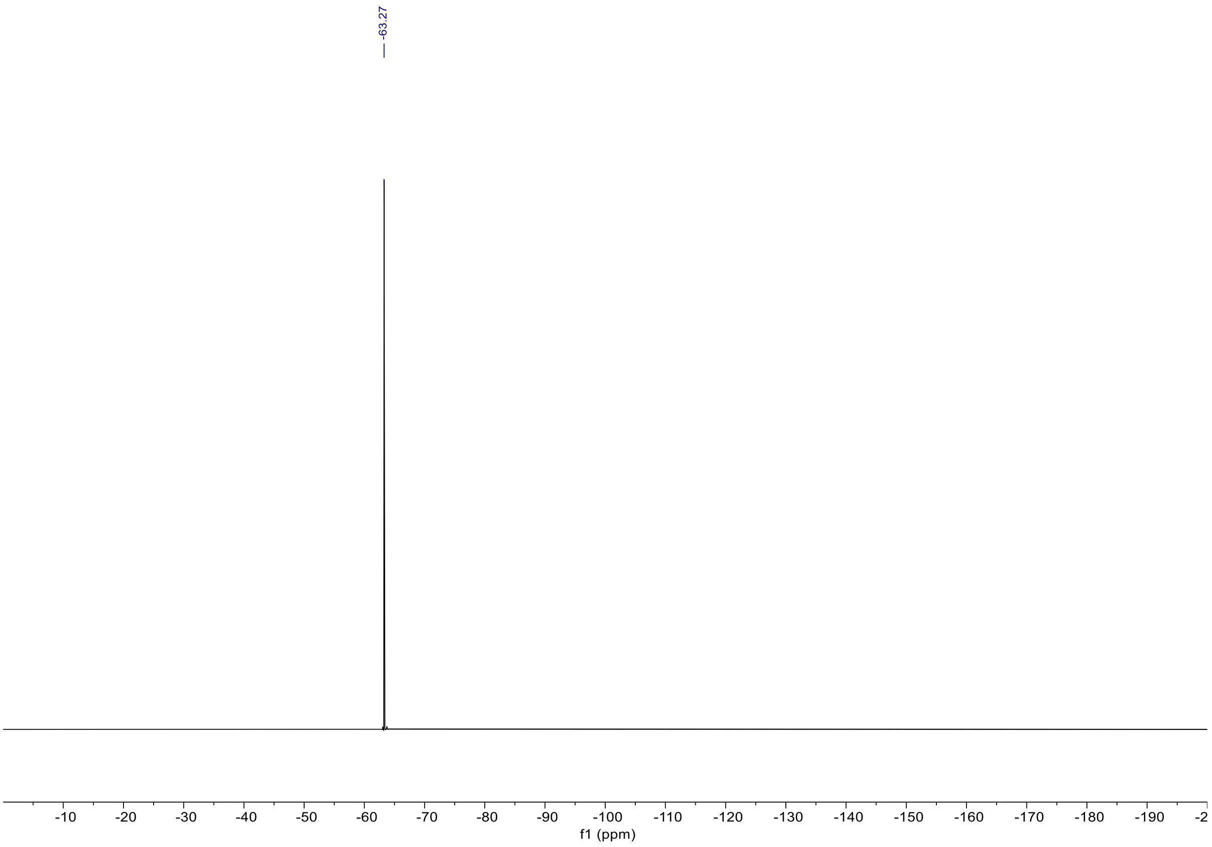

1-(2-((4-methoxyphenyl)sulfonyl)ethyl)isoquinoline (3e).

600 MHz, ^{1}H NMR in Chloroform-*d*

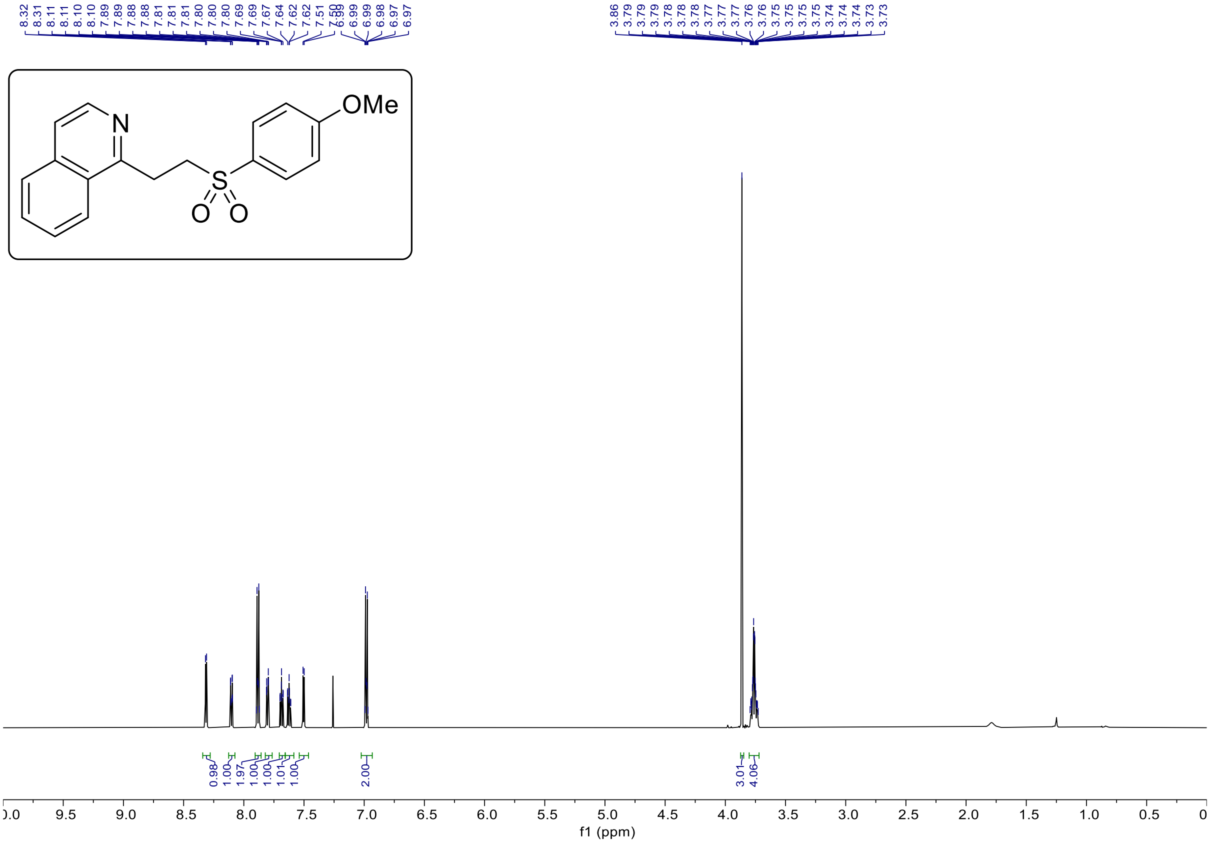

101 MHz, ^{13}C NMR in Chloroform-*d*

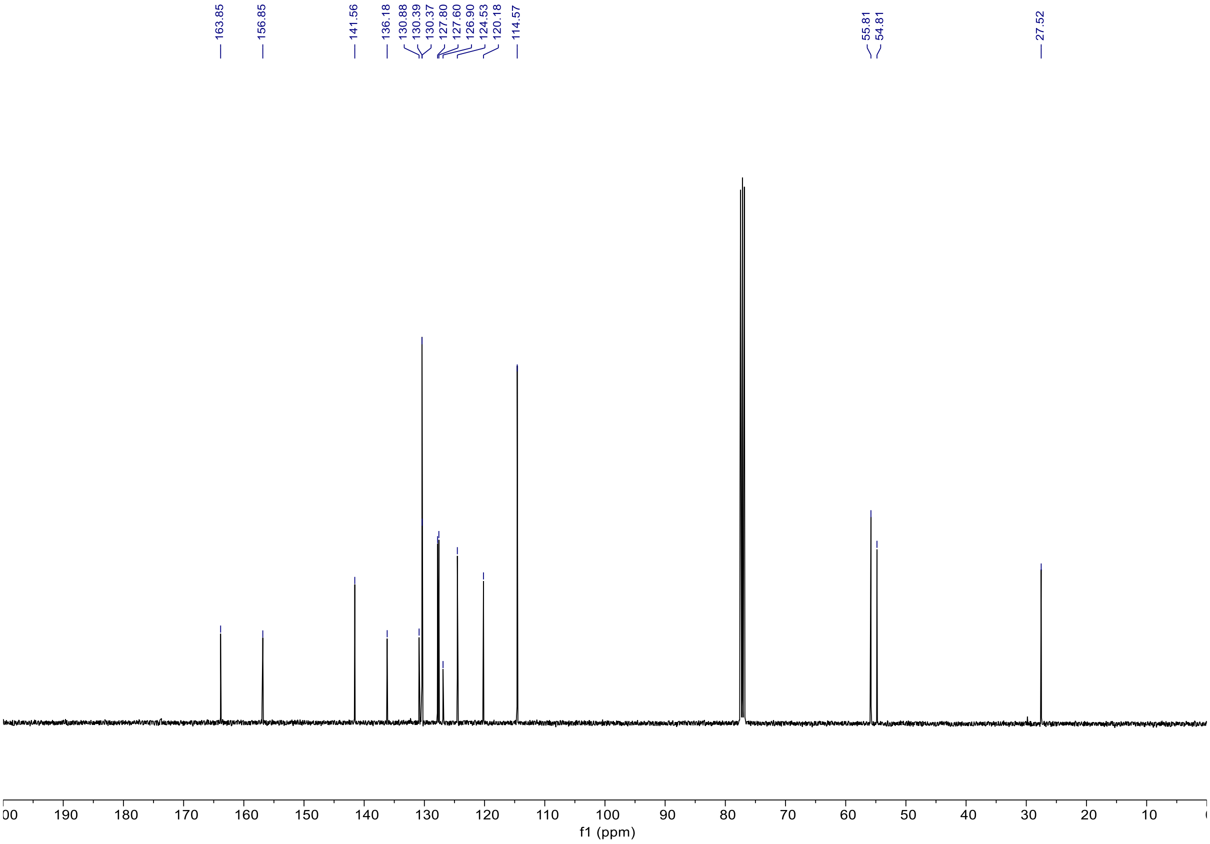

1-(2-(thiophen-2-ylsulfonyl)ethyl)isoquinoline (3f).

600 MHz, ^{1}H NMR in Chloroform-*d*

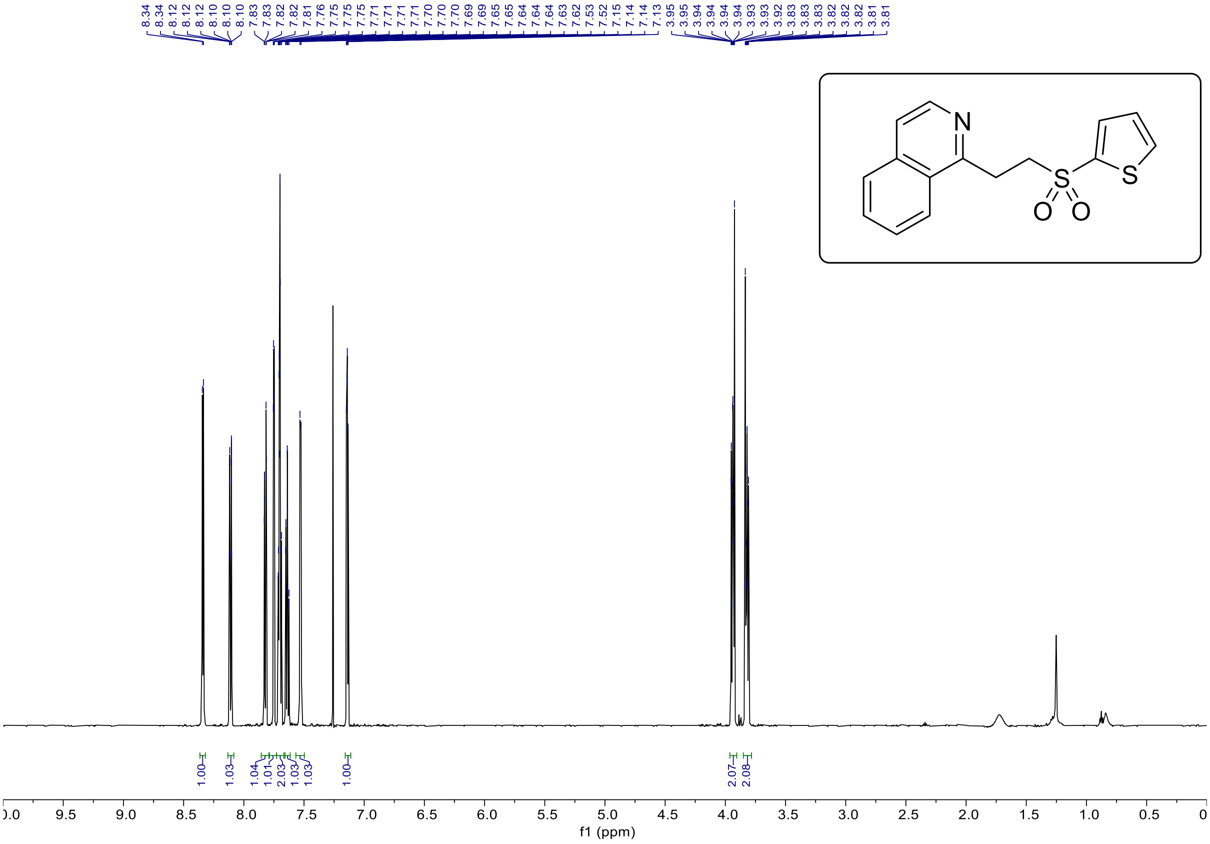

101 MHz, ^{13}C NMR in Chloroform-*d*

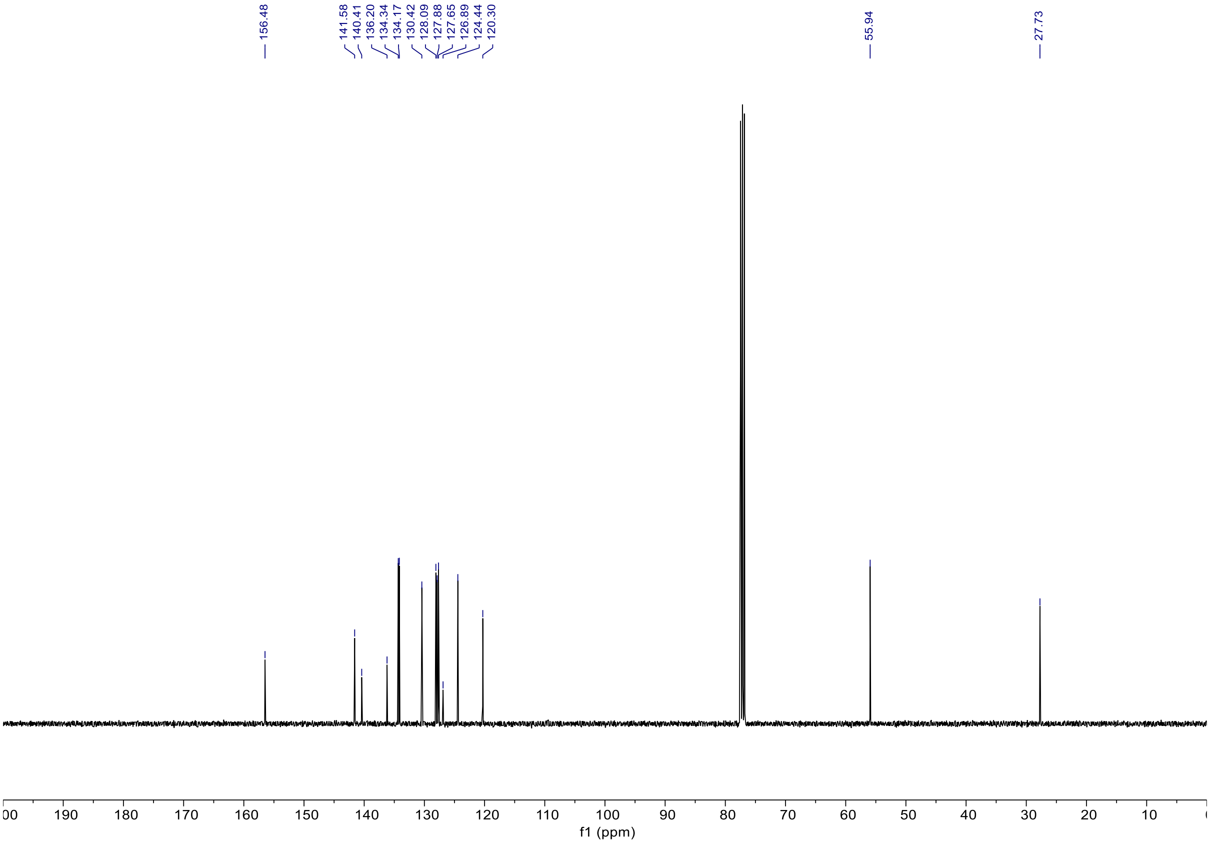

1-(2-(ethylsulfonyl)ethyl)isoquinoline (3g).

400 MHz, ^{1}H NMR in Chloroform-*d*

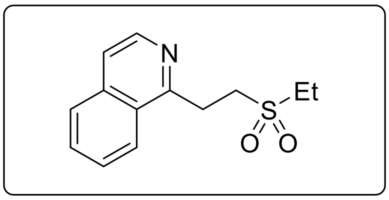

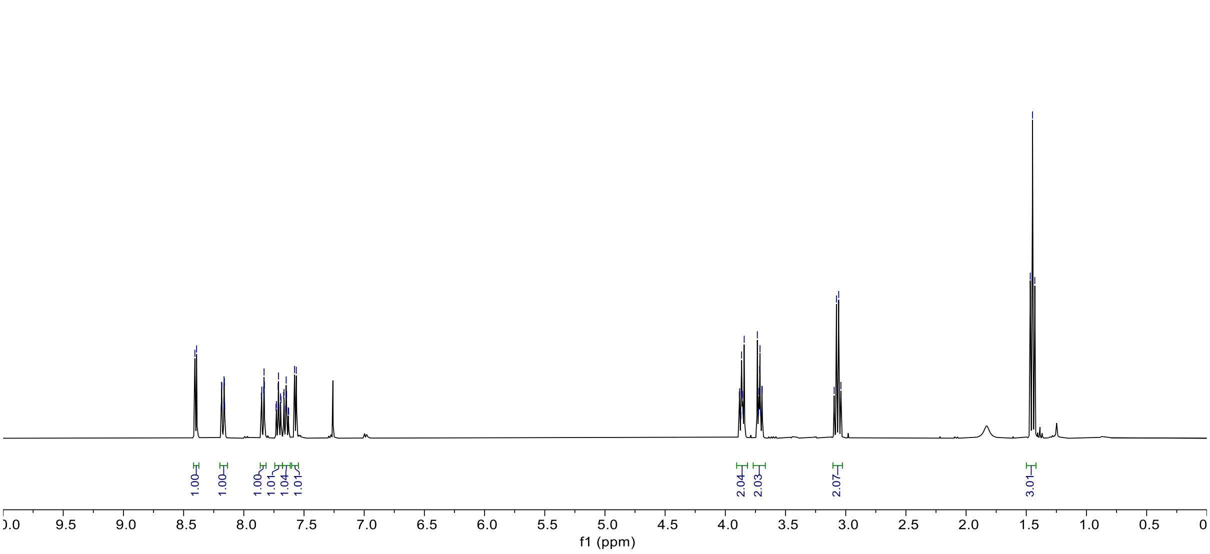

101 MHz, ^{13}C NMR in Chloroform-*d*

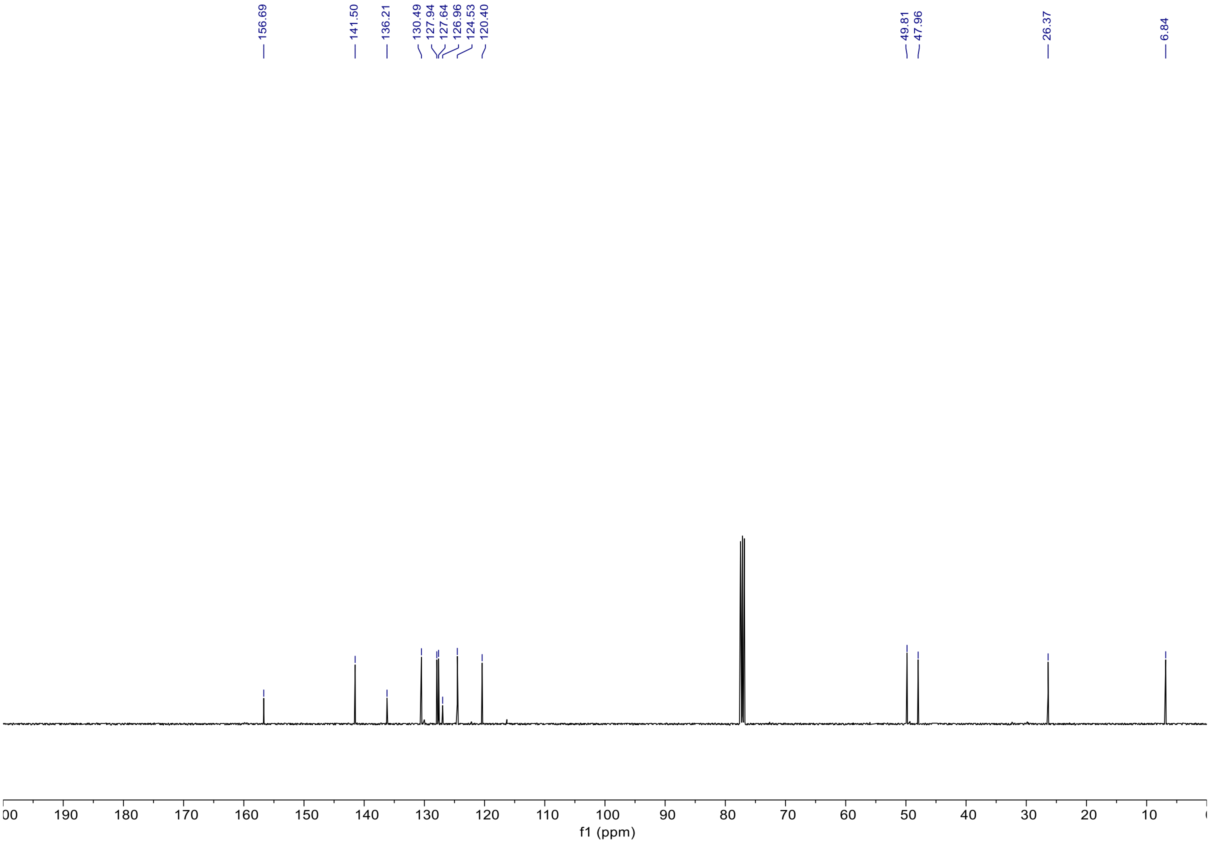

1-(2-(cyclopropylsulfonyl)ethyl)isoquinoline (3h).

400 MHz, ¹H NMR in Chloroform-*d*

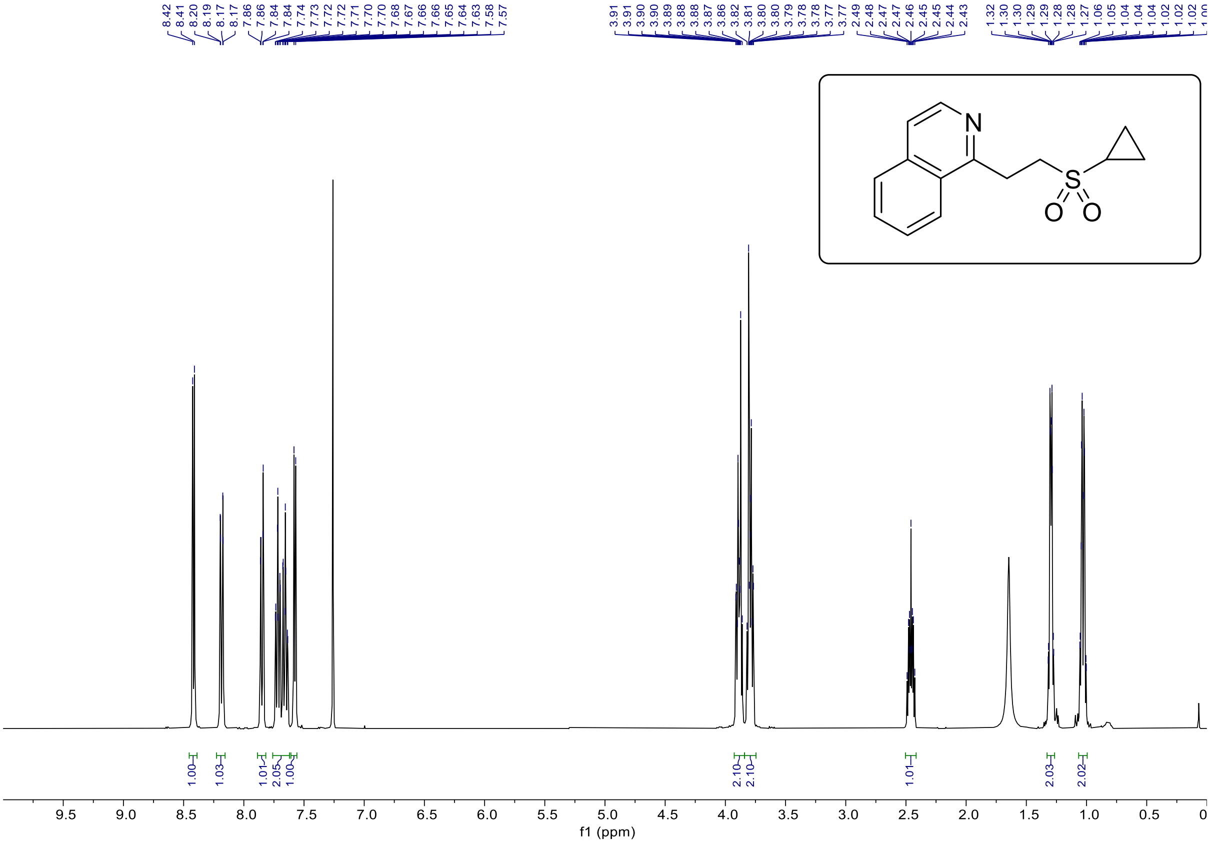

101 MHz, ¹³C NMR in Chloroform-*d*

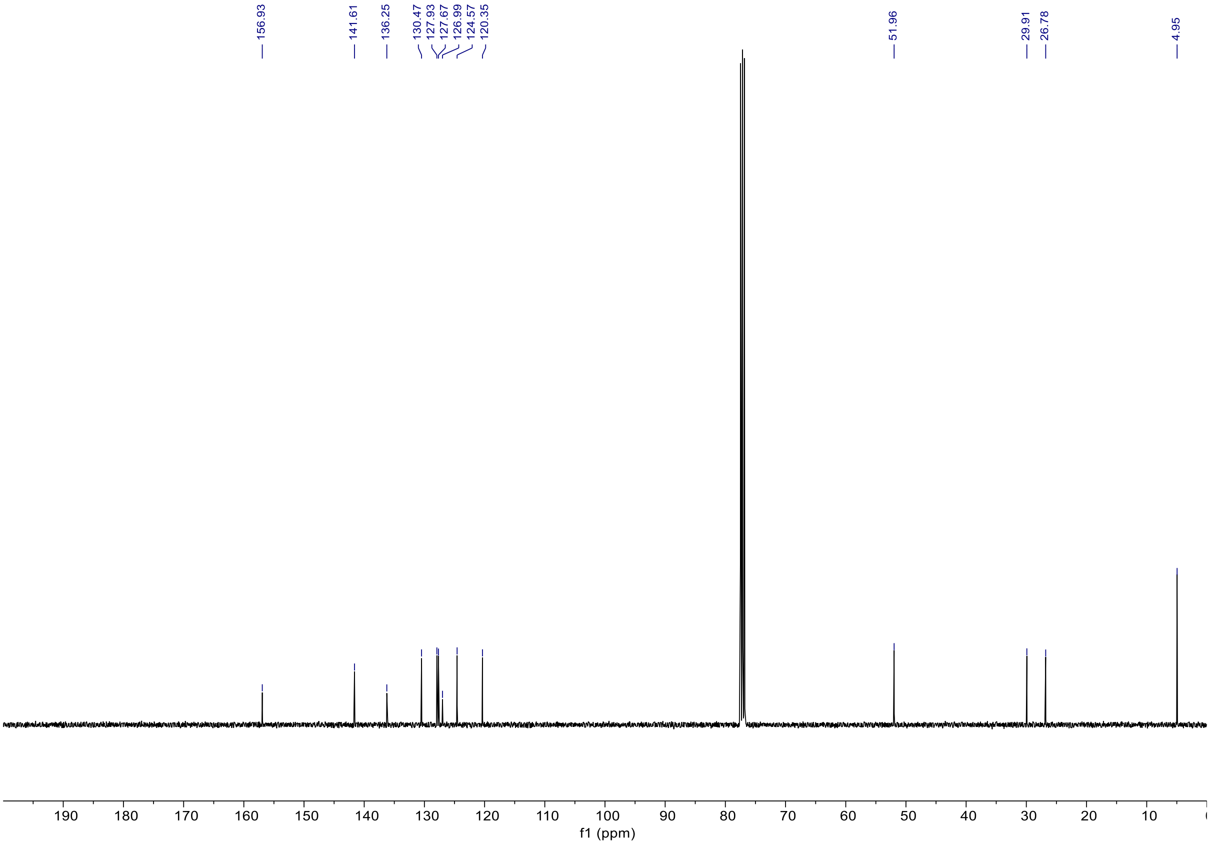

6-(2-((4-chlorophenyl)sulfonyl)ethyl)phenanthridine (3i).

500 MHz, ^{1}H NMR in Chloroform-*d*

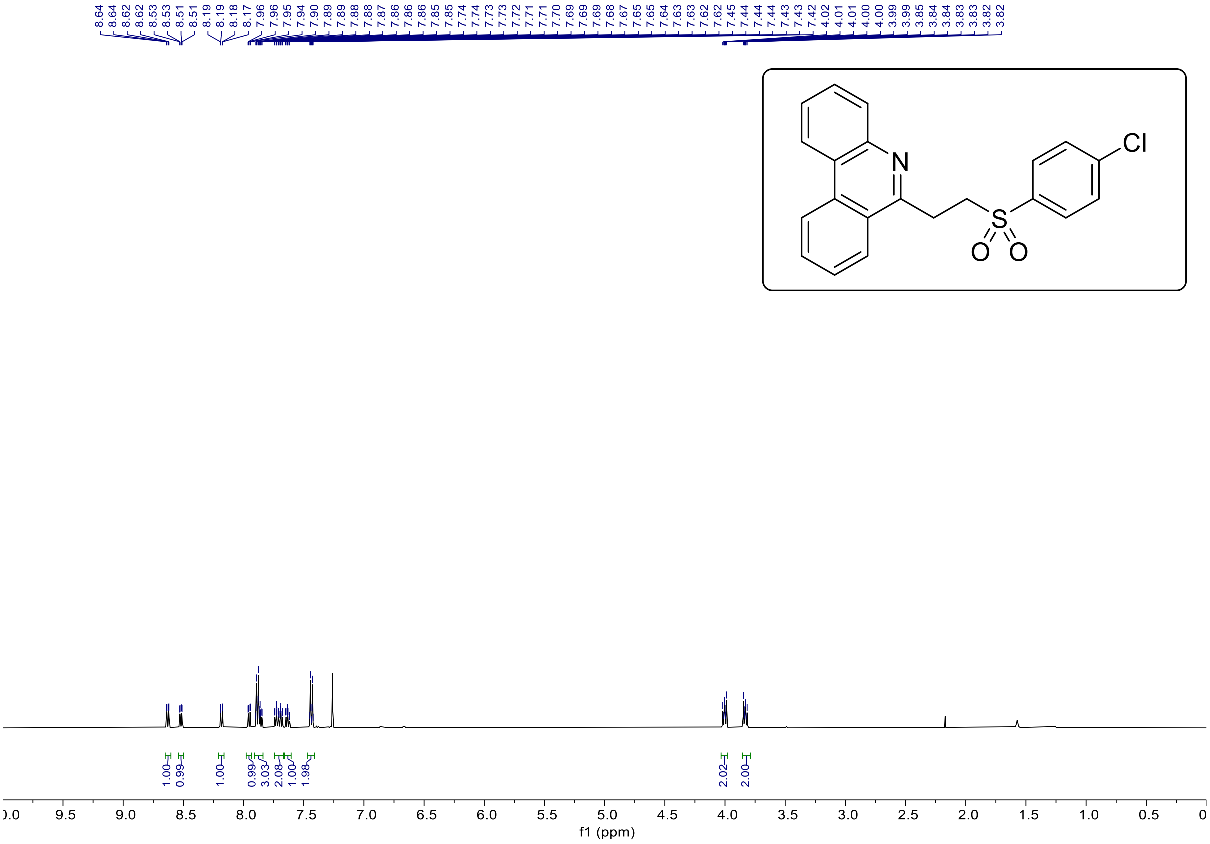

101 MHz, ^{13}C NMR in Chloroform-*d*

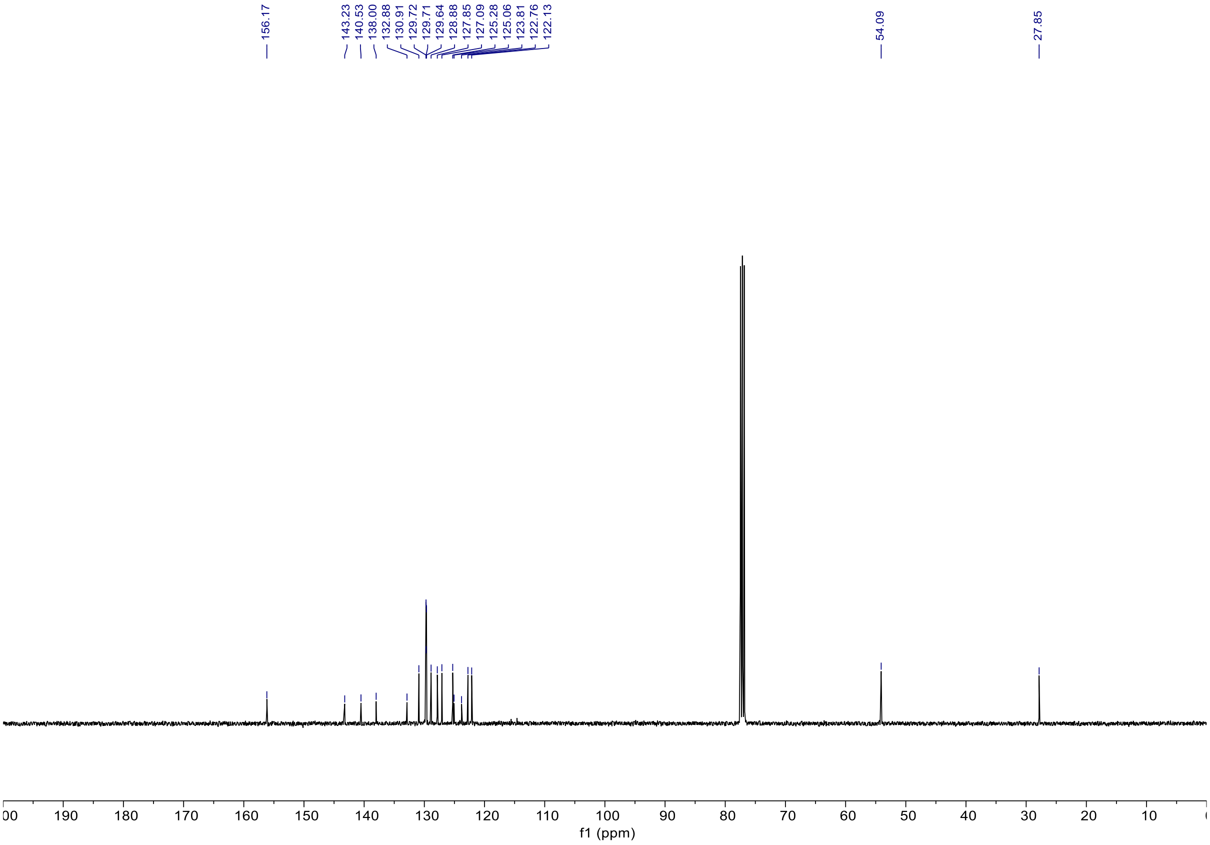

1-(2-((4-chlorophenyl)sulfonyl)ethyl)-4-methoxyphthalazine (3j).

400 MHz, ^{1}H NMR in Chloroform-*d*

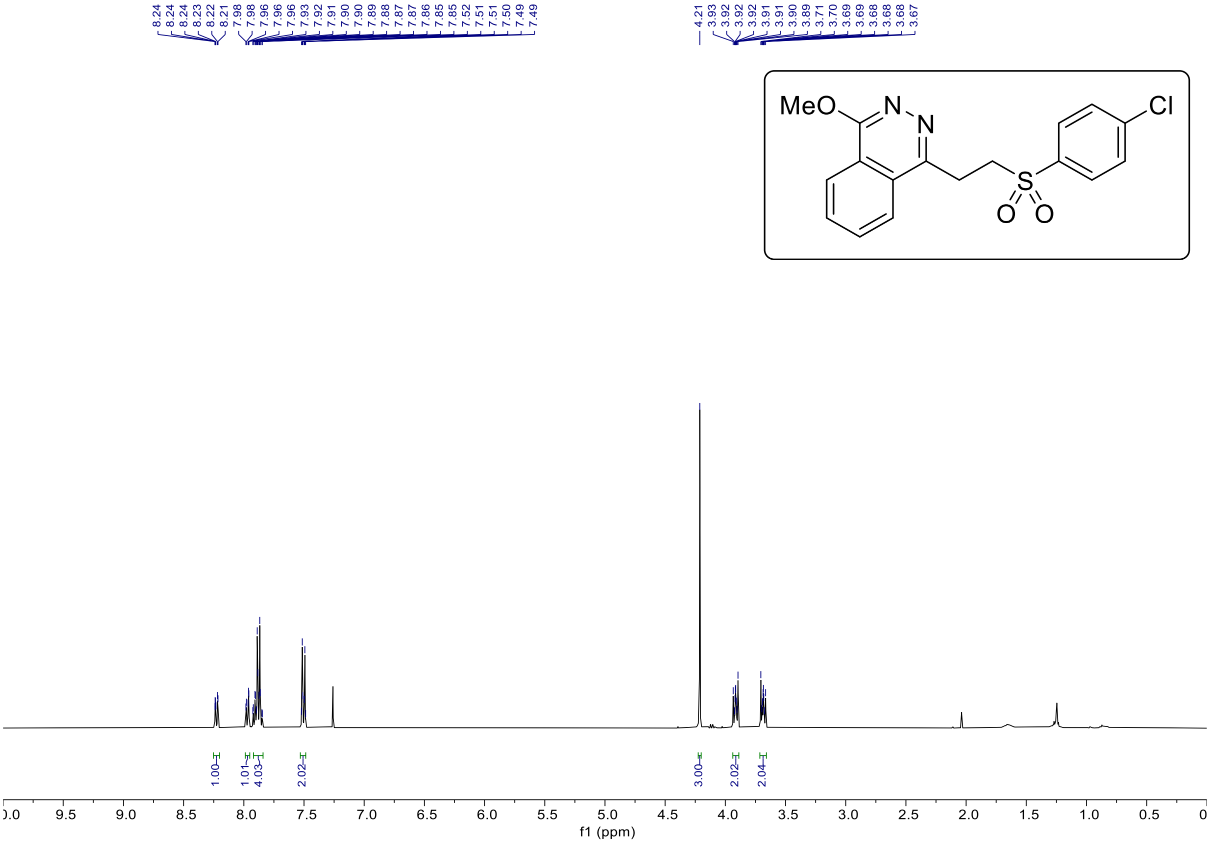

101 MHz, ^{13}C NMR in Chloroform-*d*

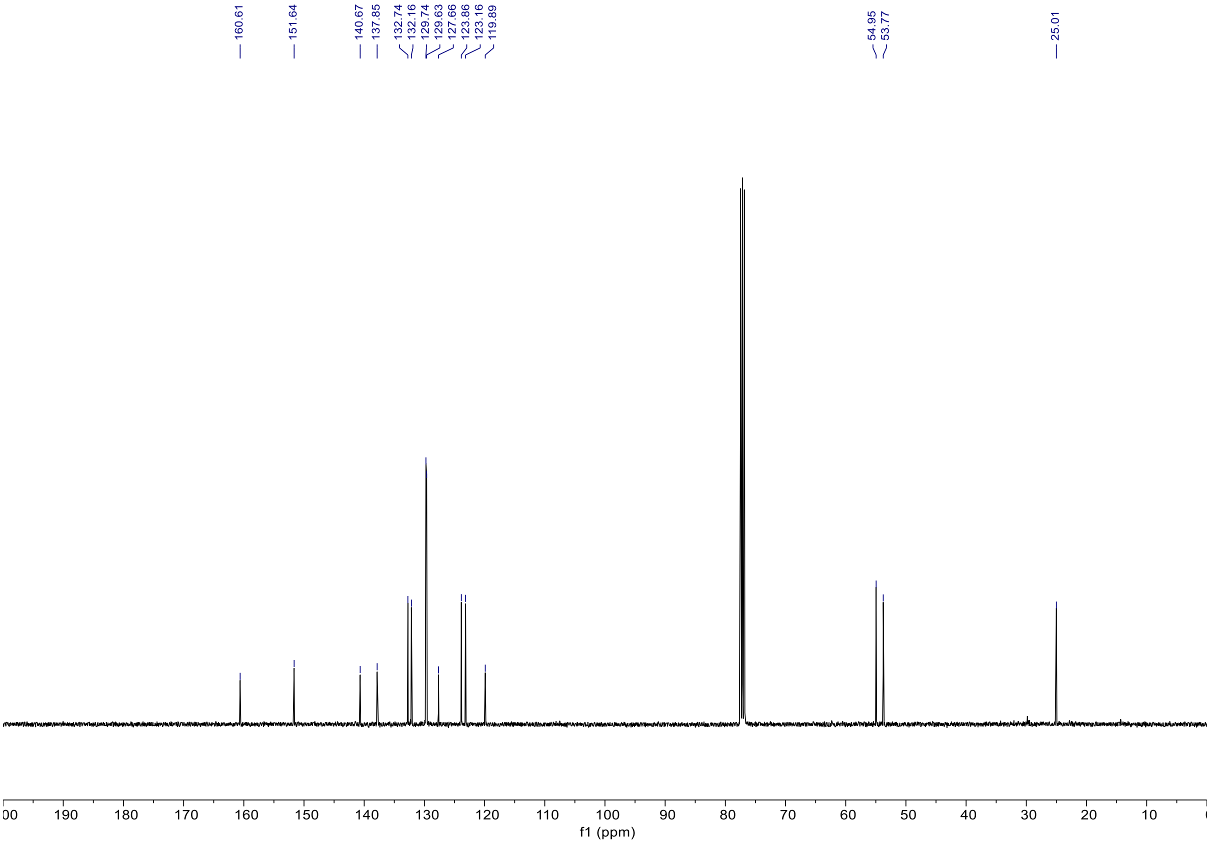

2-(2-((4-chlorophenyl)sulfonyl)ethyl)benzo[d]thiazole (3k).

400 MHz, ^{1}H NMR in Chloroform-*d*

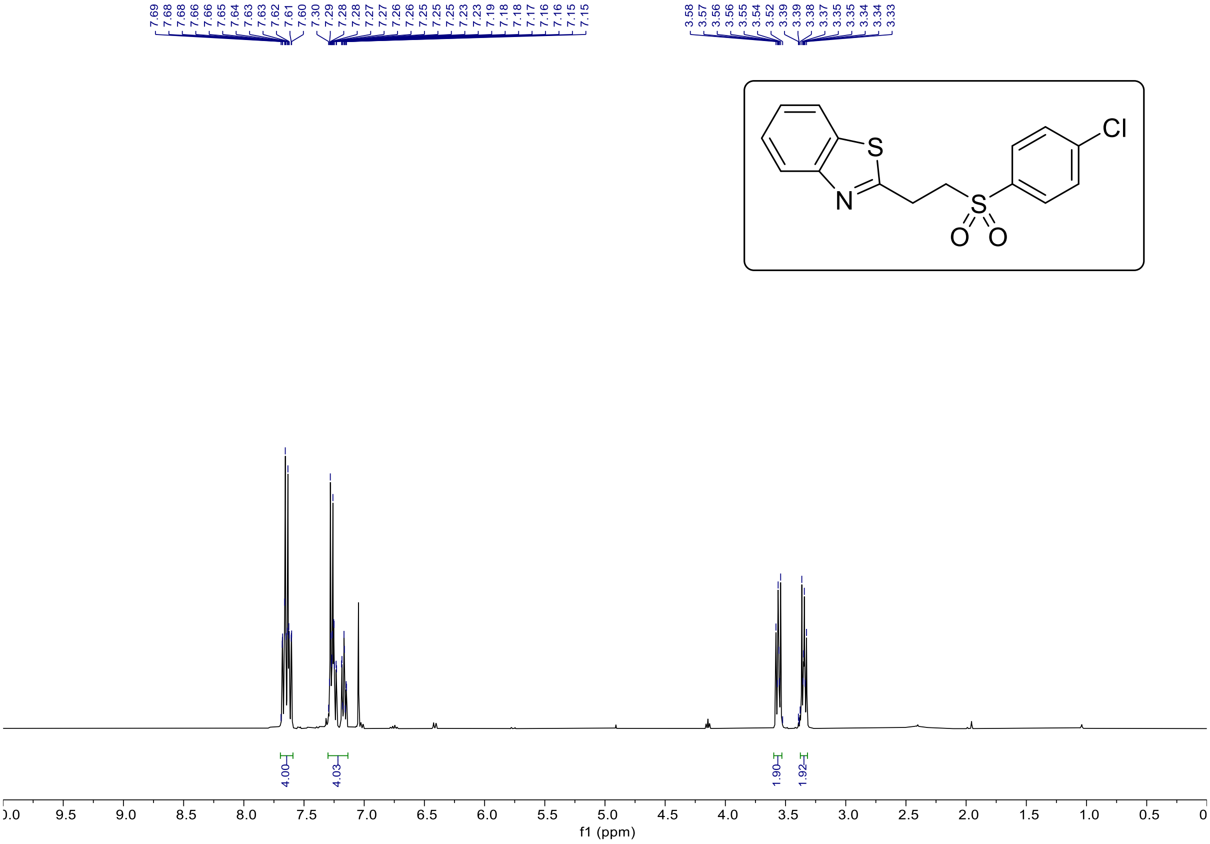

101 MHz, ^{13}C NMR in Chloroform-*d*

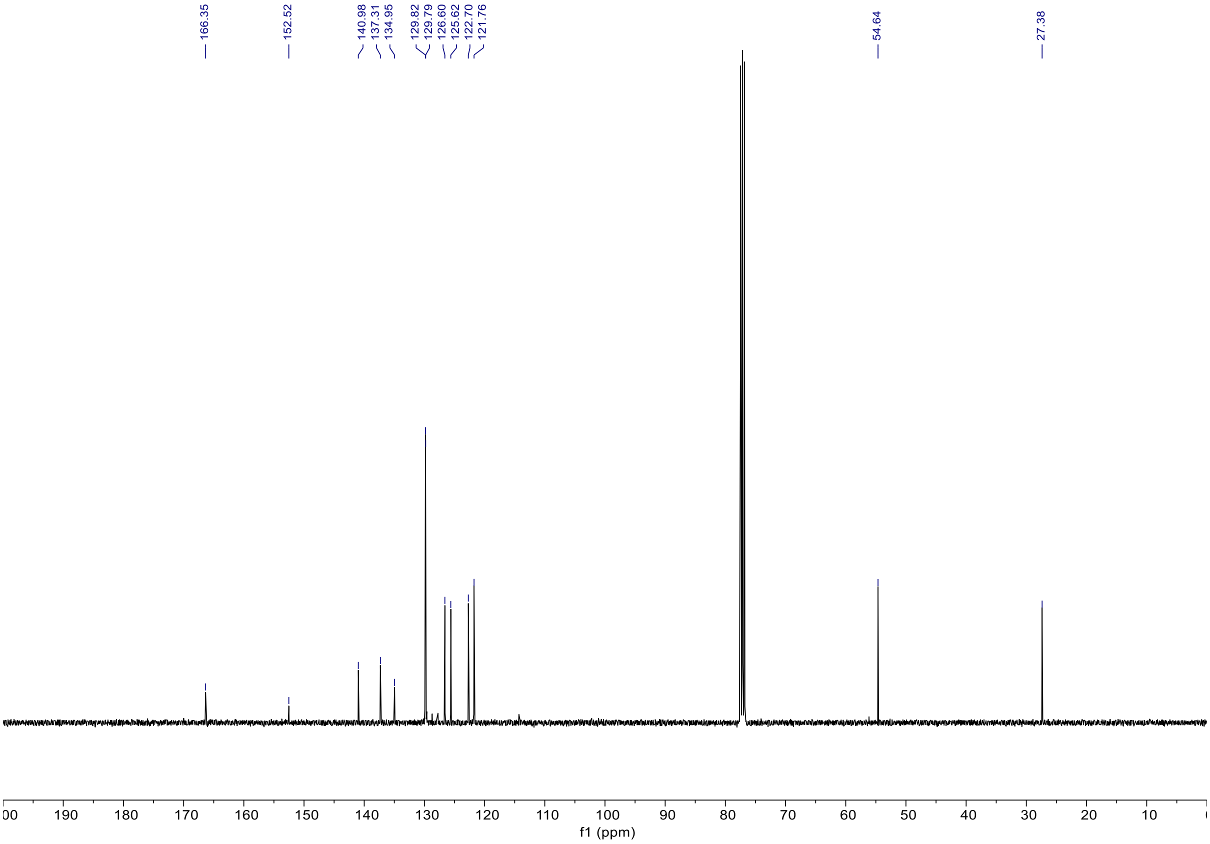

methyl 6-(2-((4-chlorophenyl)sulfonyl)ethyl)-4-methylnicotinate (3l).

400 MHz, ^{1}H NMR in Chloroform-*d*

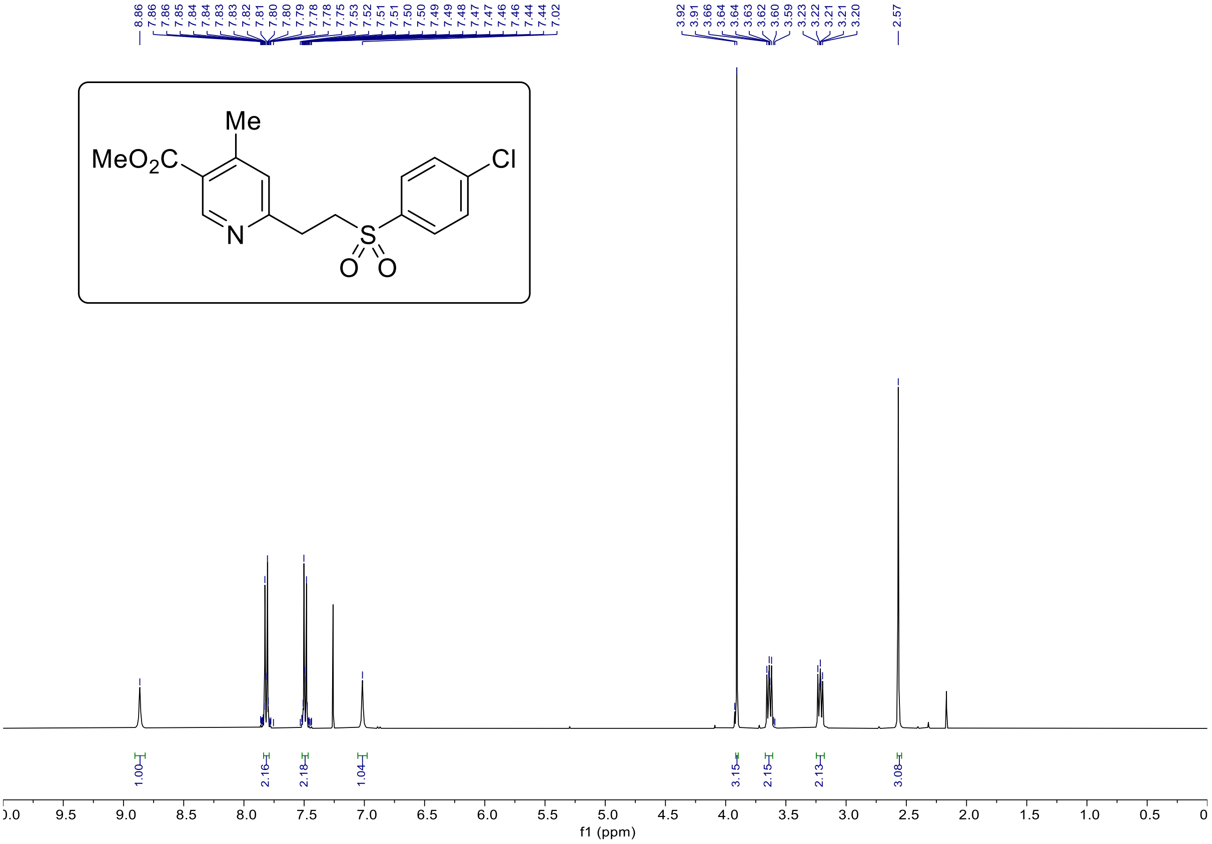

101 MHz, ^{13}C NMR in Chloroform-*d*

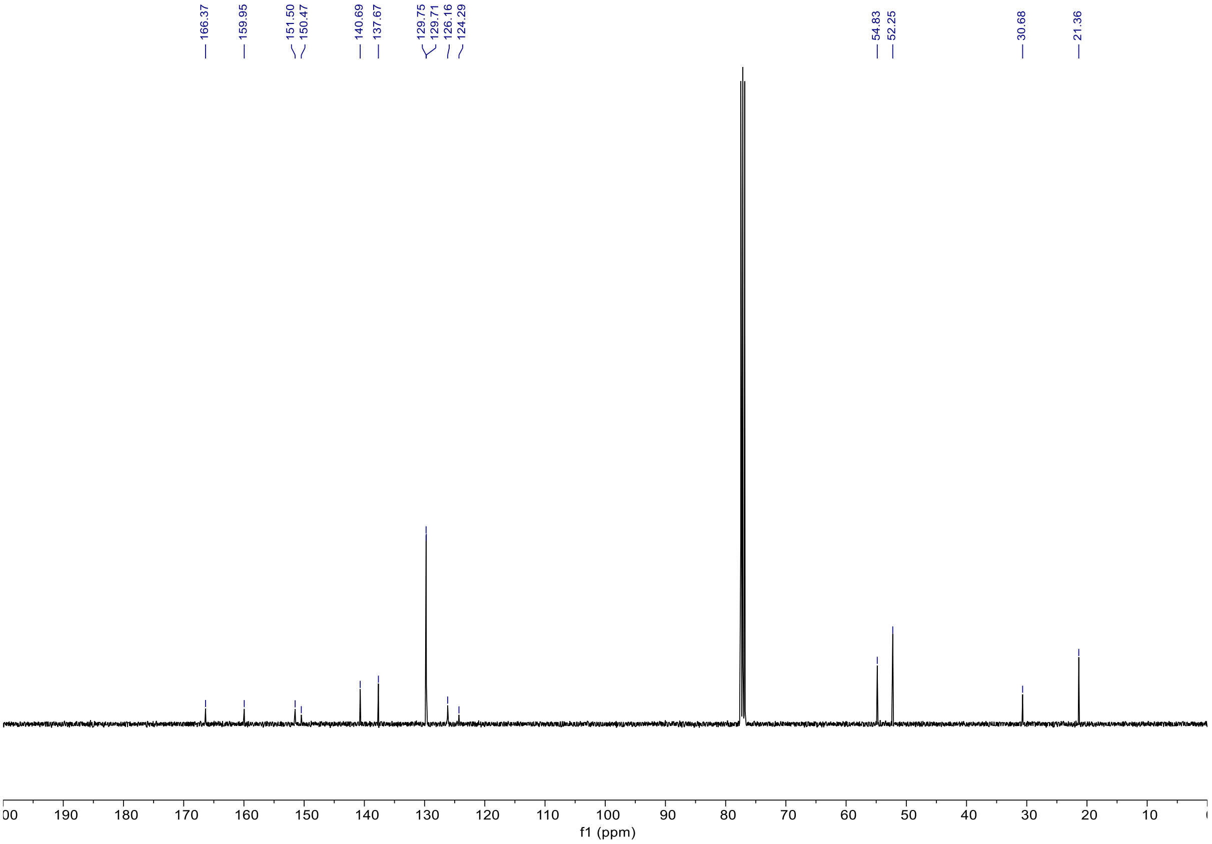

(2-(isoquinolin-1-yl)ethyl)diphenylphosphine oxide (3m).

400 MHz, ^{1}H NMR in Chloroform-*d*

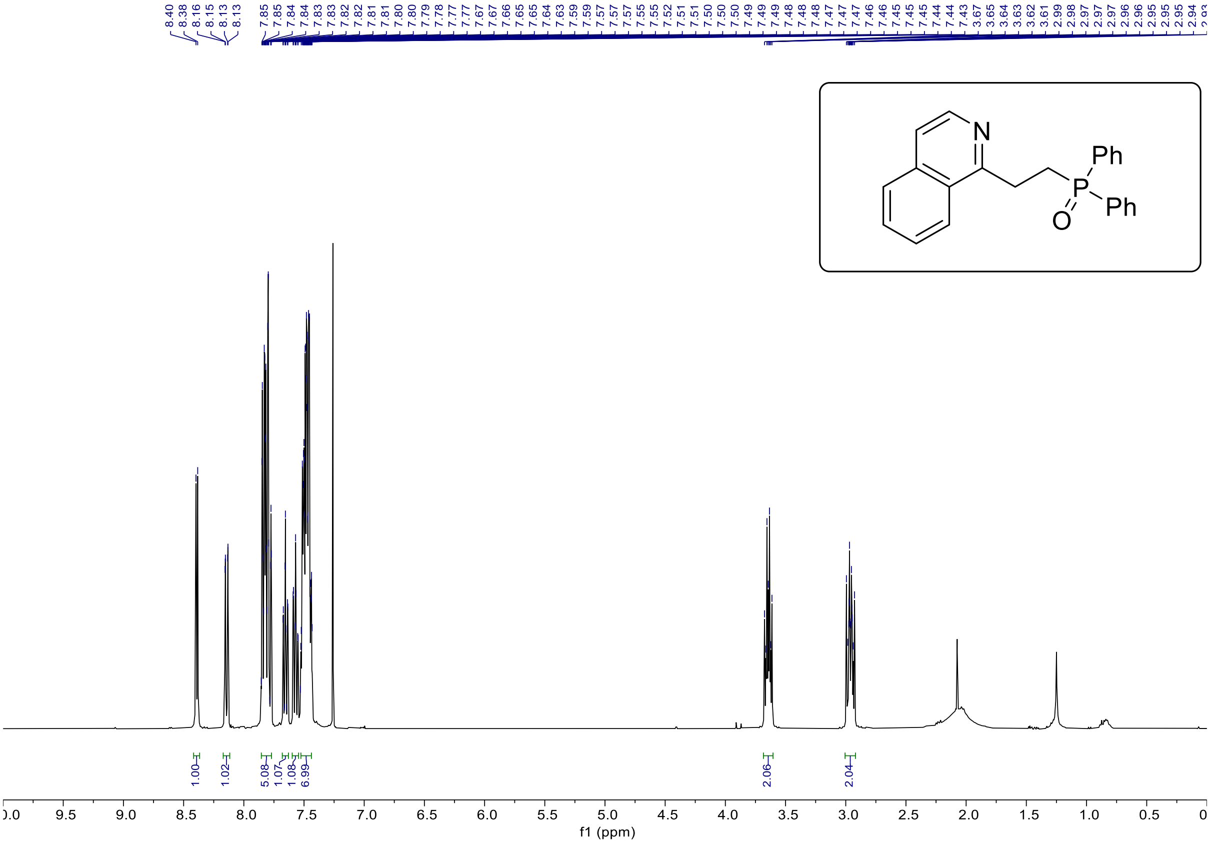

101 MHz, ^{13}C NMR in Chloroform-*d*

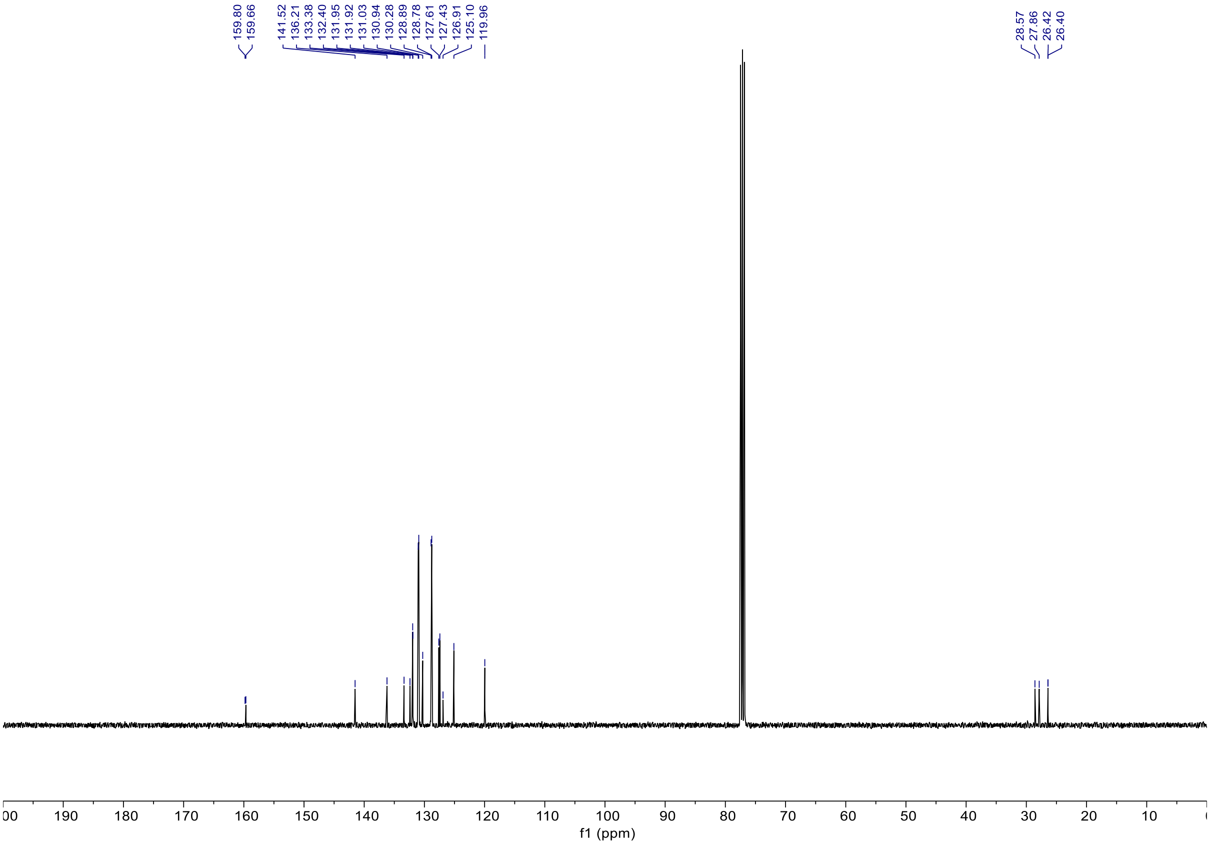

(2-(isoquinolin-1-yl)ethyl)di-p-tolylphosphine oxide (3n).

400 MHz, ^{1}H NMR in Chloroform-*d*

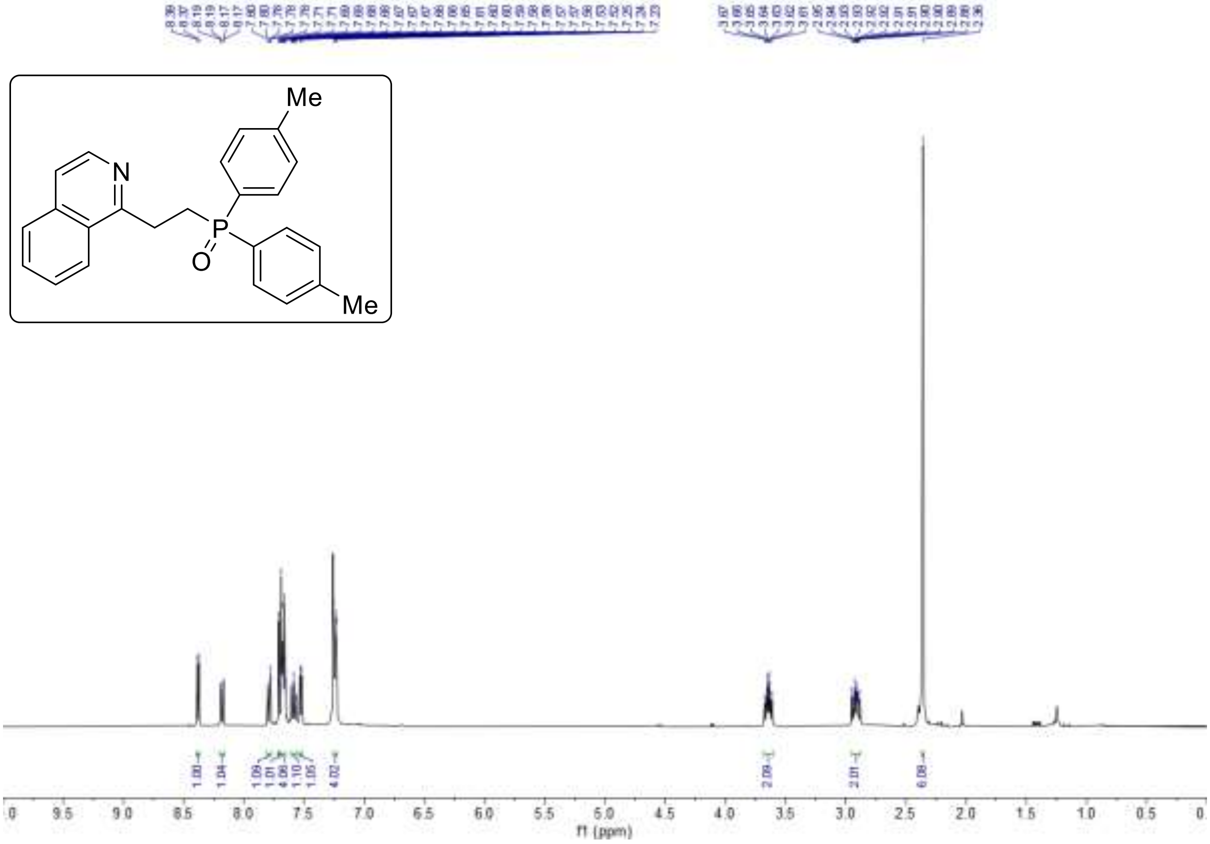

101 MHz, ^{13}C NMR in Chloroform-*d*

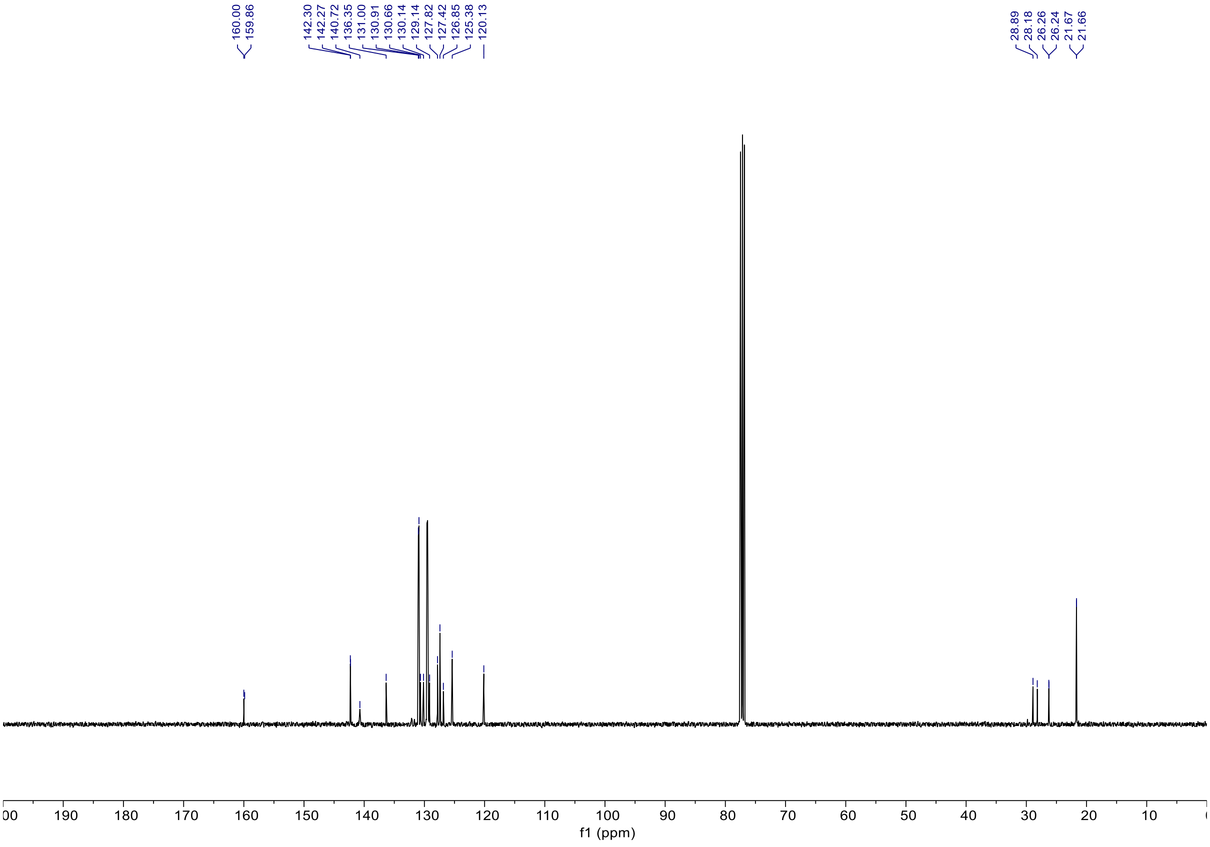

bis(3,5-dimethylphenyl)(2-(isoquinolin-1-yl)ethyl)phosphine oxide (3o).

400 MHz, ^{1}H NMR in Chloroform-*d*

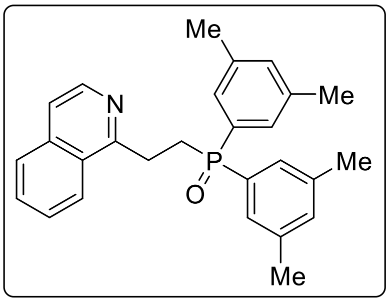

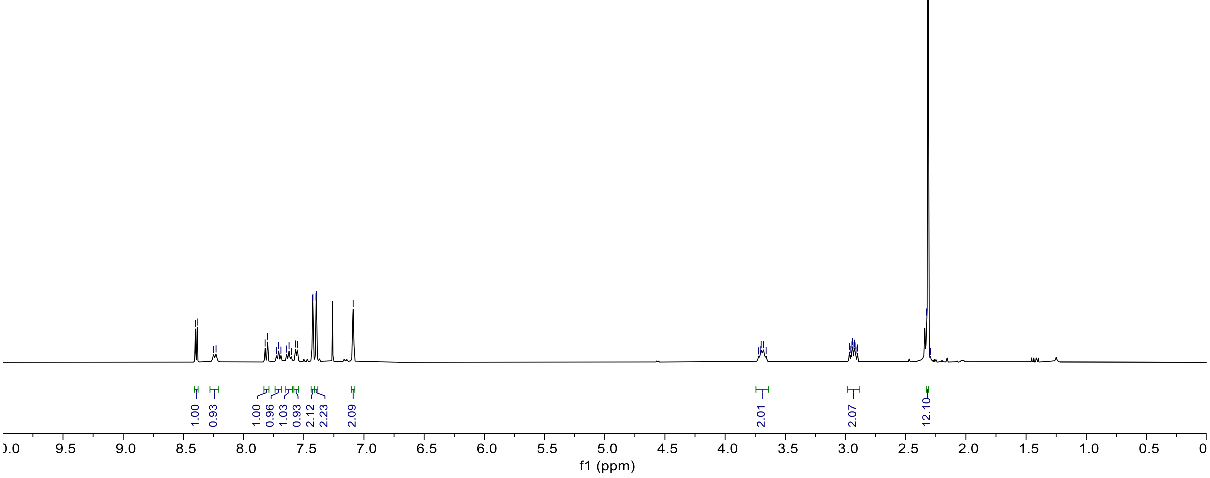

101 MHz, ^{13}C NMR in Chloroform-*d*

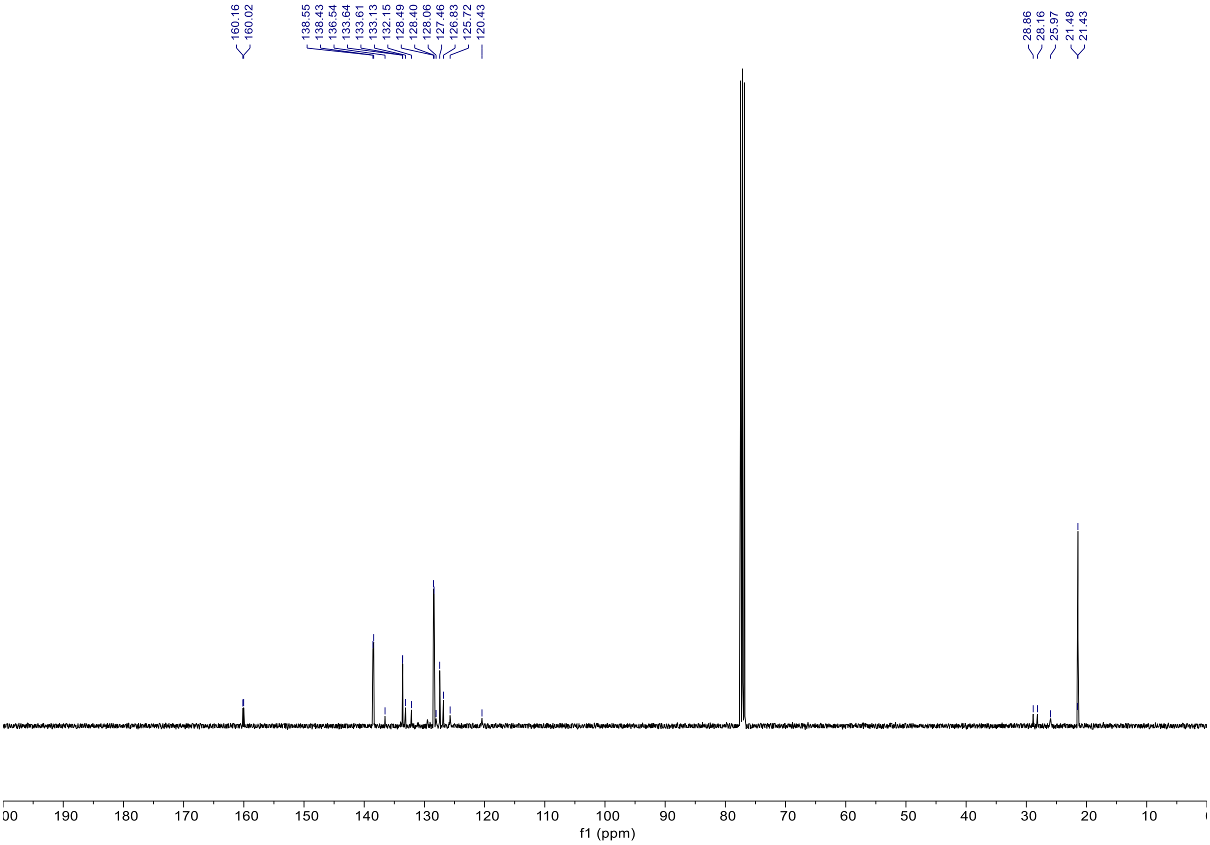

(2-(isoquinolin-1-yl)ethyl)bis(4-methoxyphenyl)phosphine oxide (3p).

400 MHz, ^{1}H NMR in Chloroform-*d*

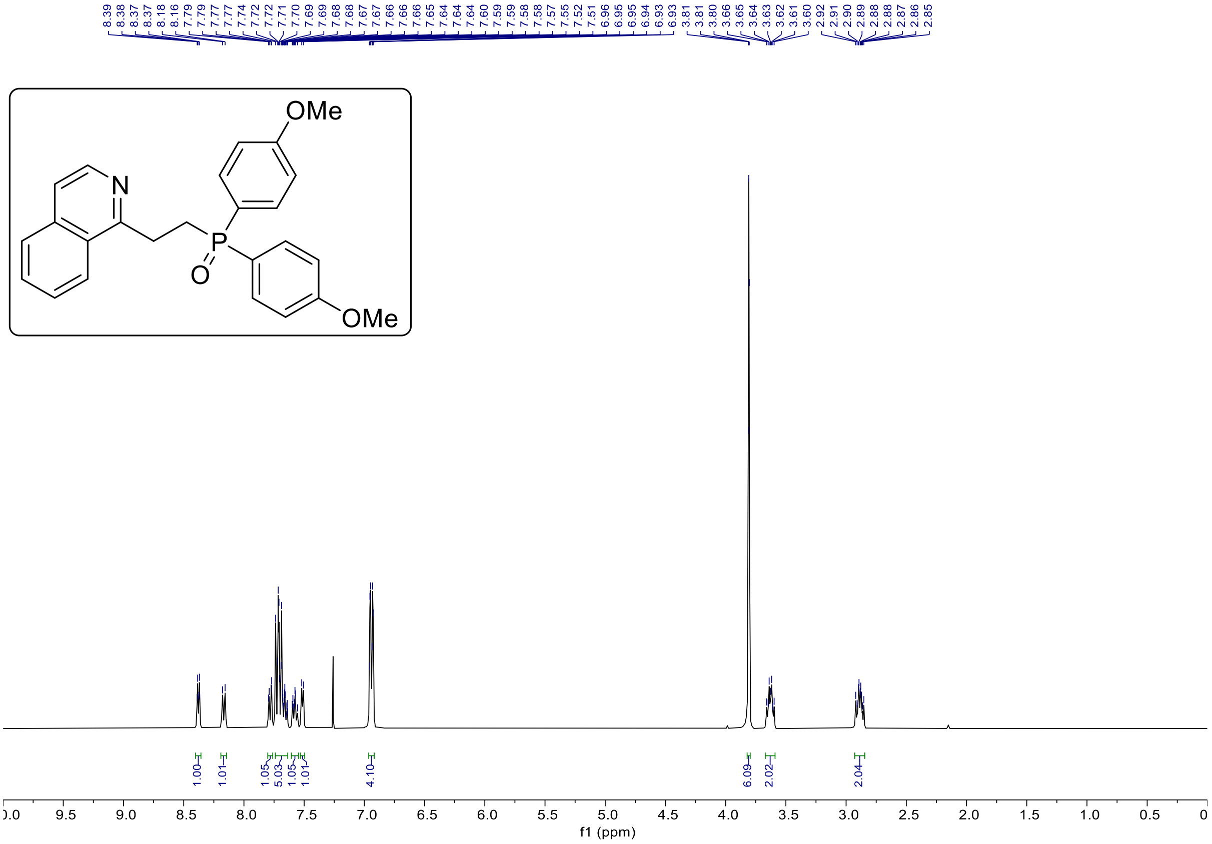

101 MHz, ^{13}C NMR in Chloroform-*d*

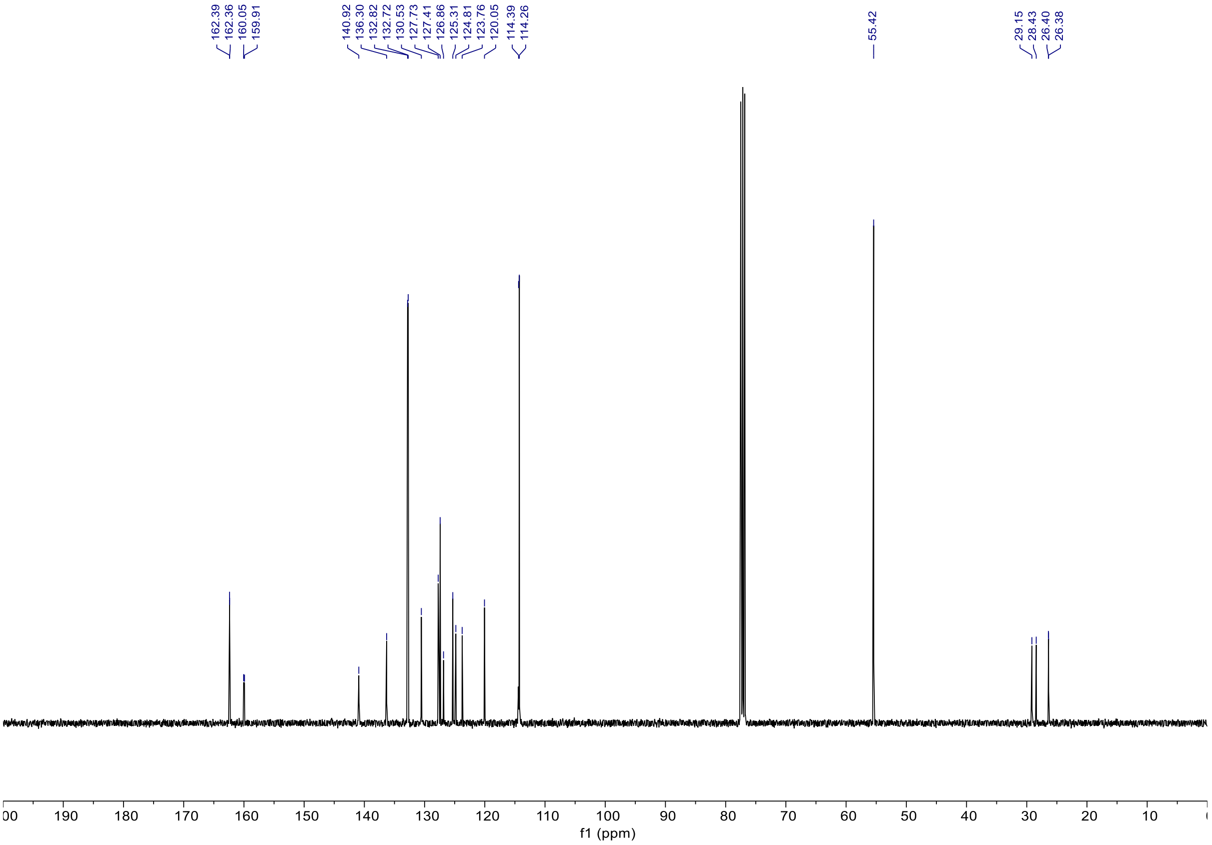

(2-(6-bromoisoquinolin-1-yl)ethyl)diphenylphosphine oxide (3q).

400 MHz, ^{1}H NMR in Chloroform-*d*

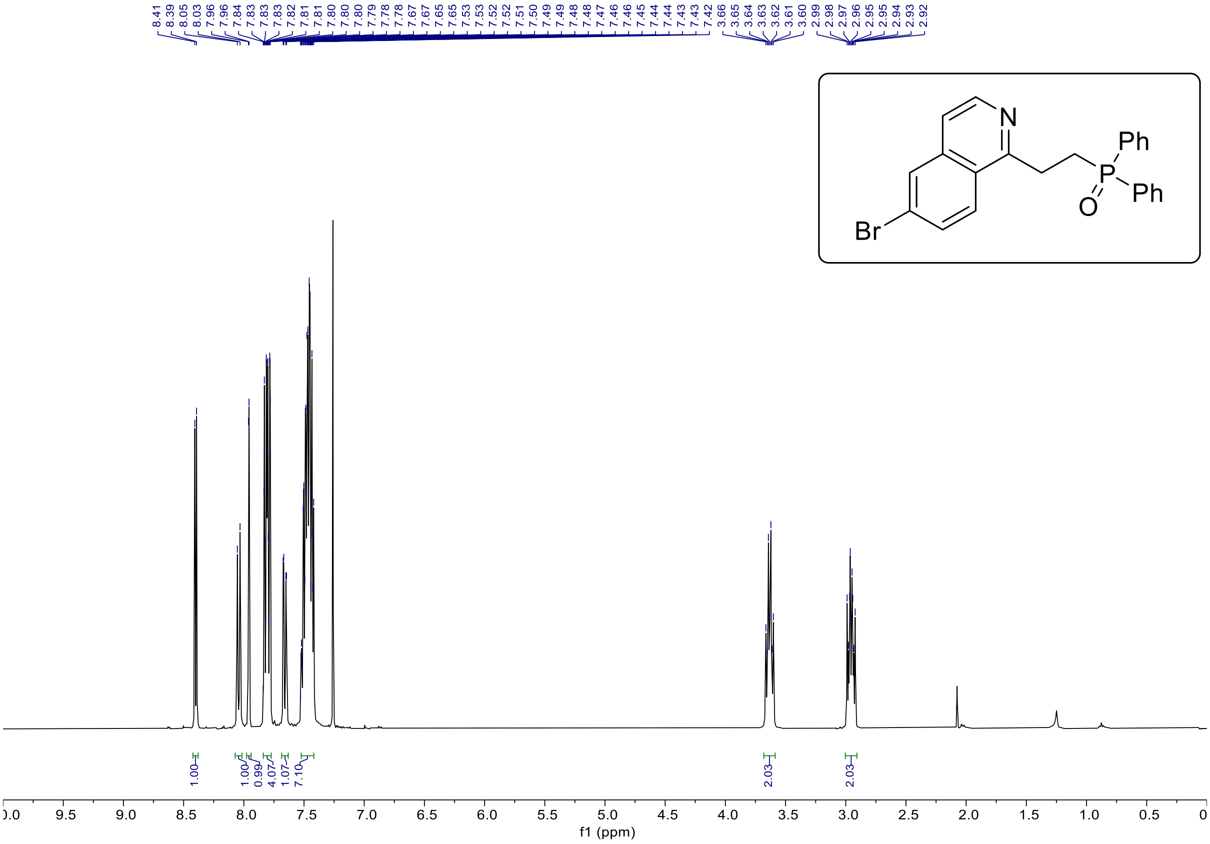

101 MHz, ^{13}C NMR in Chloroform-*d*

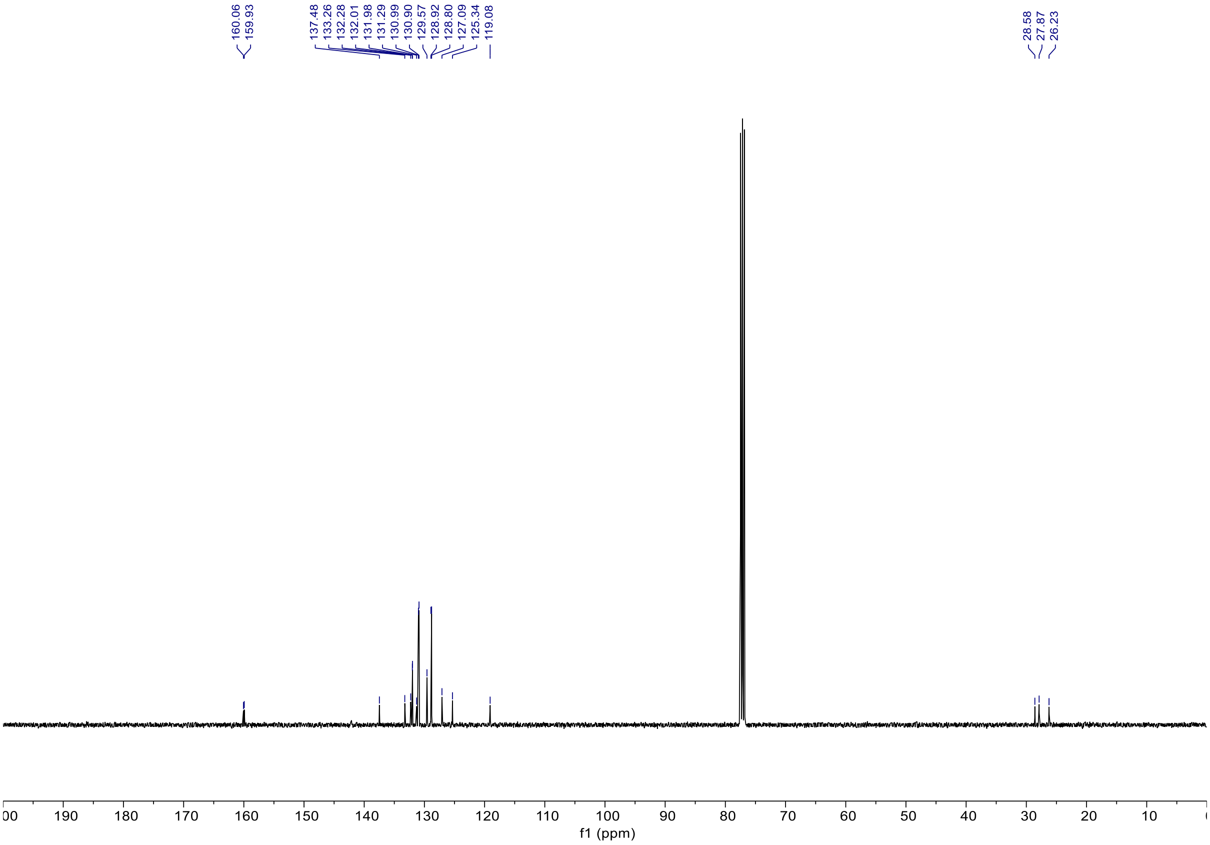

(2-(phenanthridin-6-yl)ethyl)diphenylphosphine oxide (3r).

400 MHz, ^{1}H NMR in Chloroform-*d*

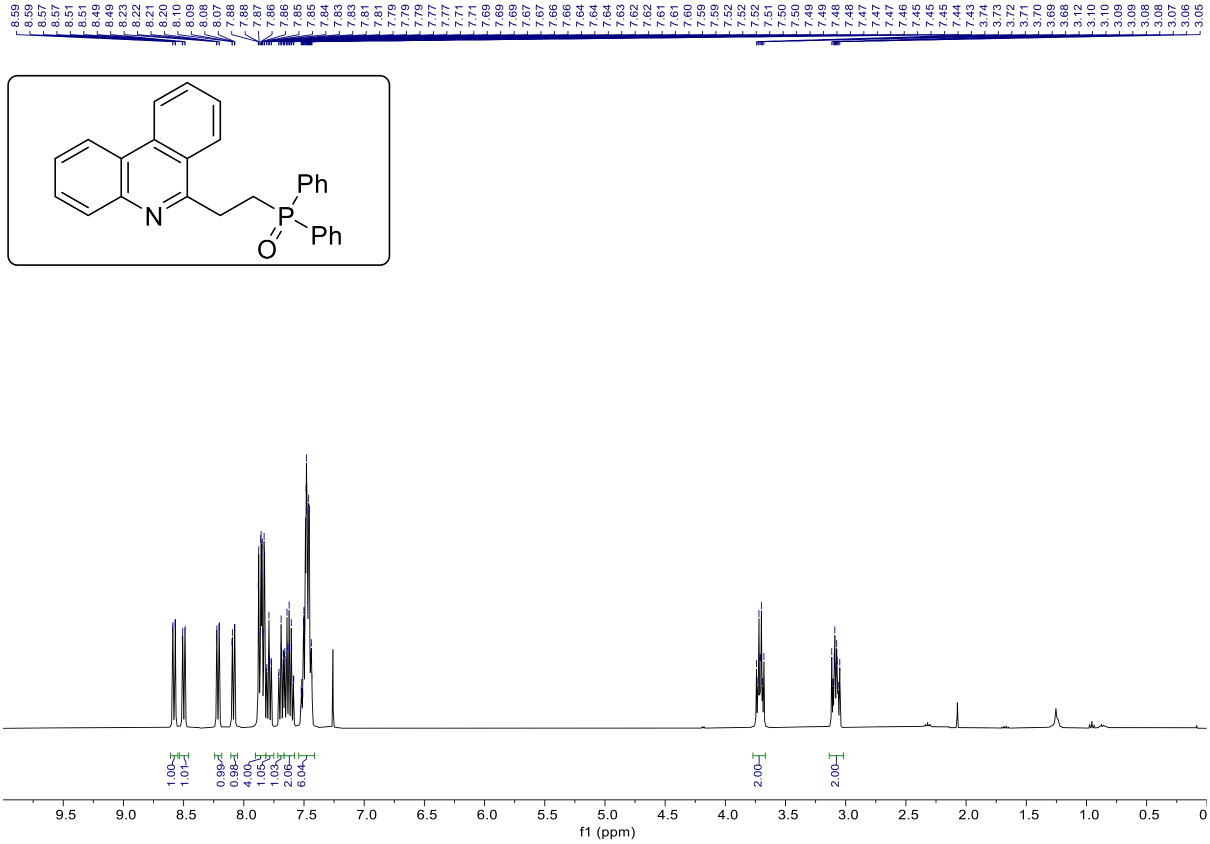

101 MHz, ^{13}C NMR in Chloroform-*d*

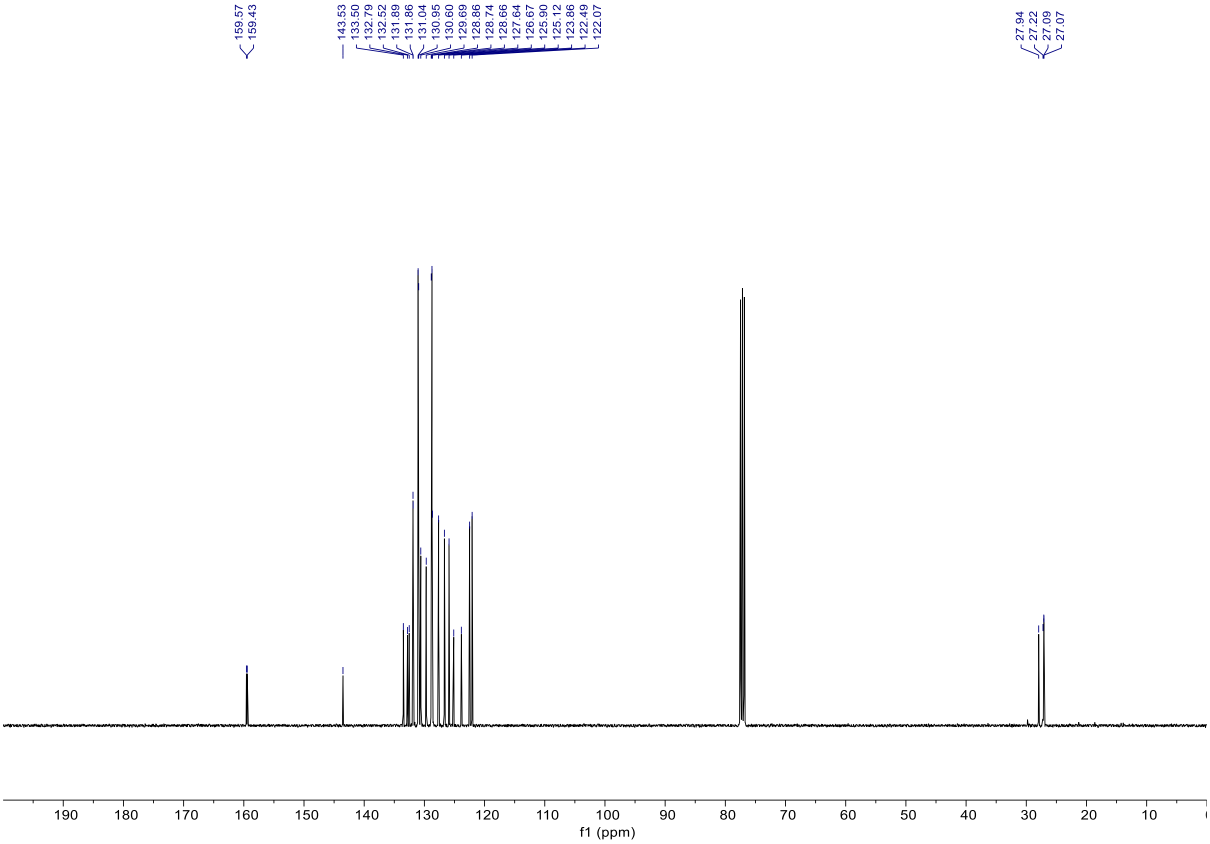

1-(2-(benzylthio)ethyl)isoquinoline (3s).

600 MHz, ^{1}H NMR in Chloroform-*d*

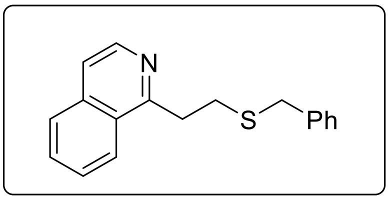

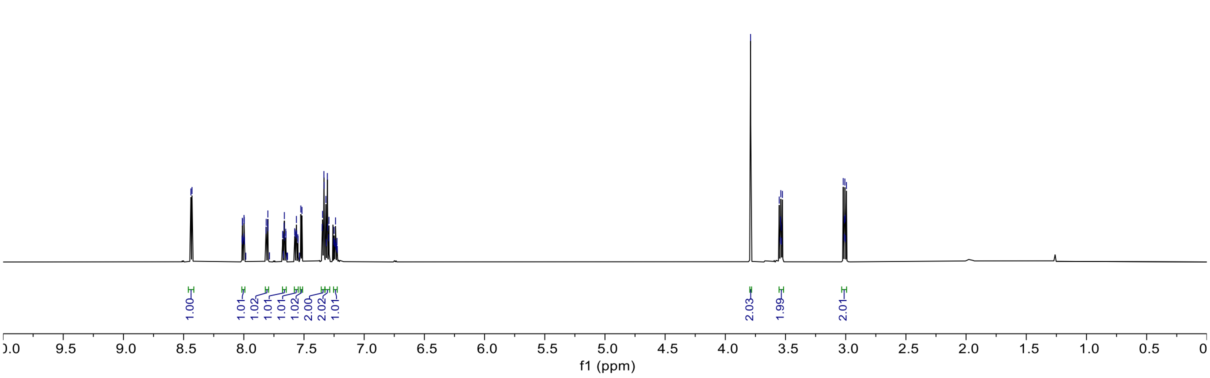

101 MHz, ^{13}C NMR in Chloroform-*d*

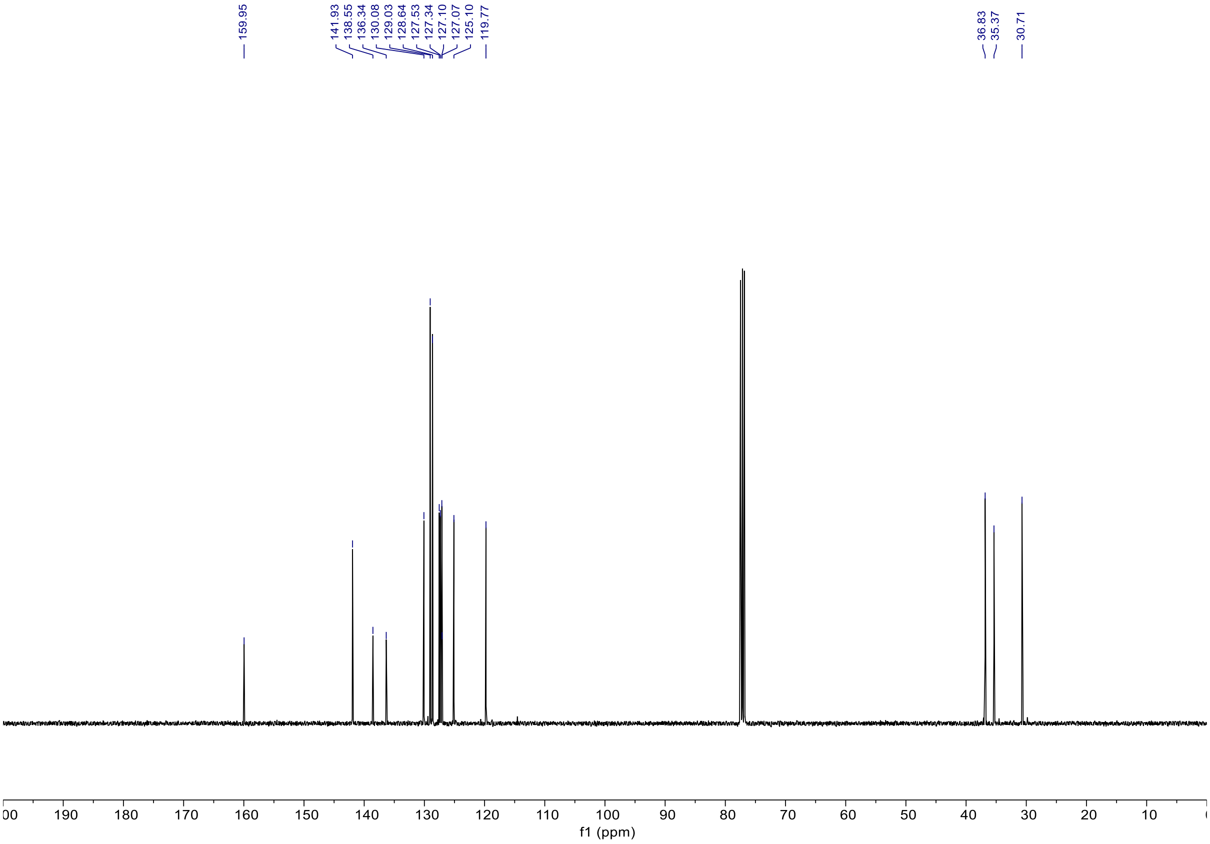

1-(2-(cyclohexylthio)ethyl)isoquinoline (3t).

600 MHz, ^{1}H NMR in Chloroform-*d*

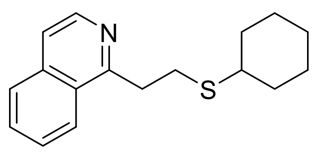

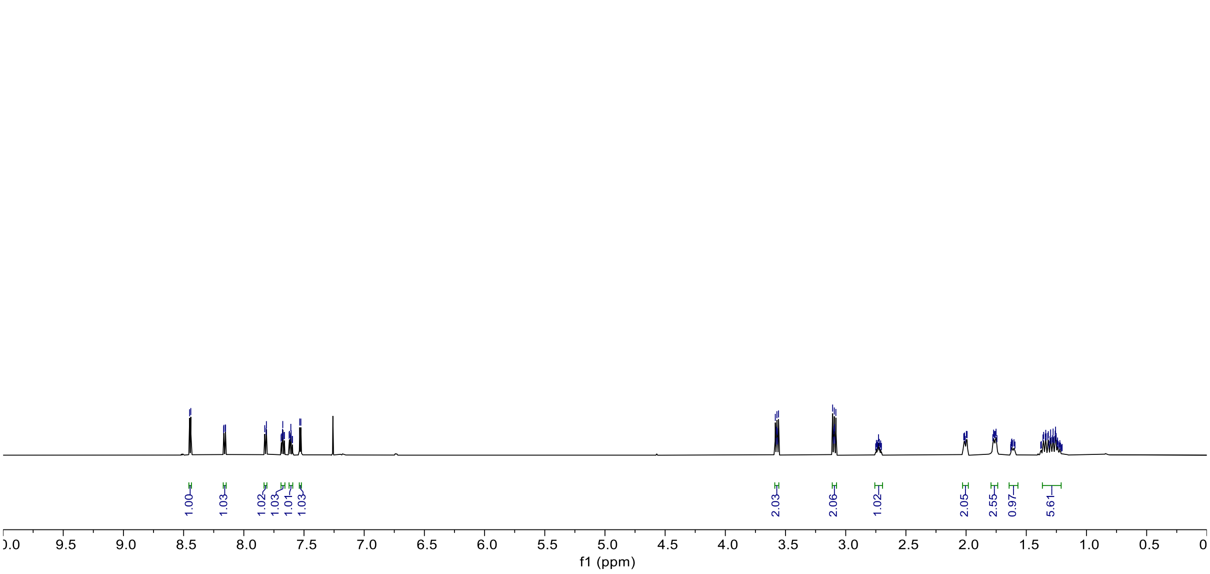

101 MHz, ^{13}C NMR in Chloroform-*d*

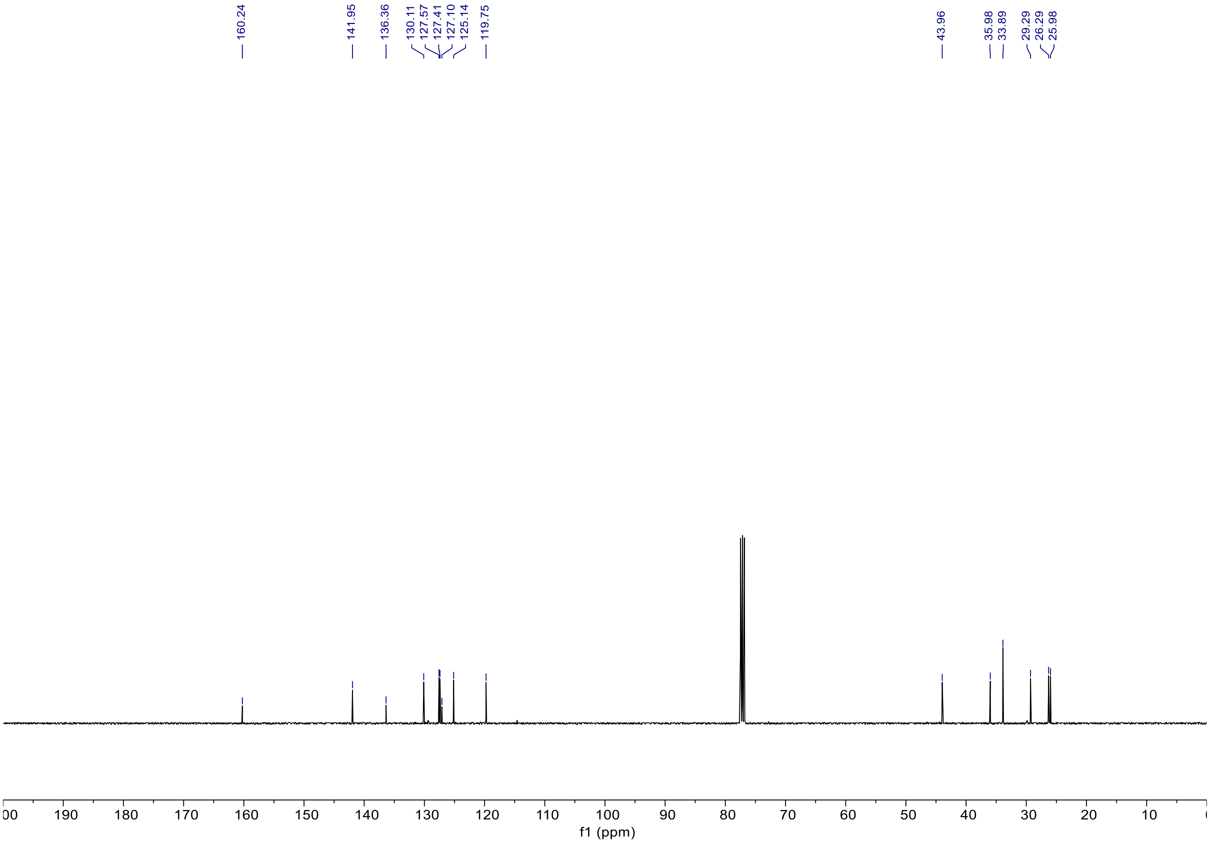

1-(2-(tert-butylthio)ethyl)isoquinoline (3u).

600 MHz, ^{1}H NMR in Chloroform-*d*

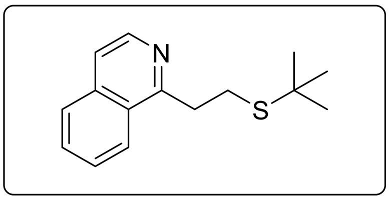

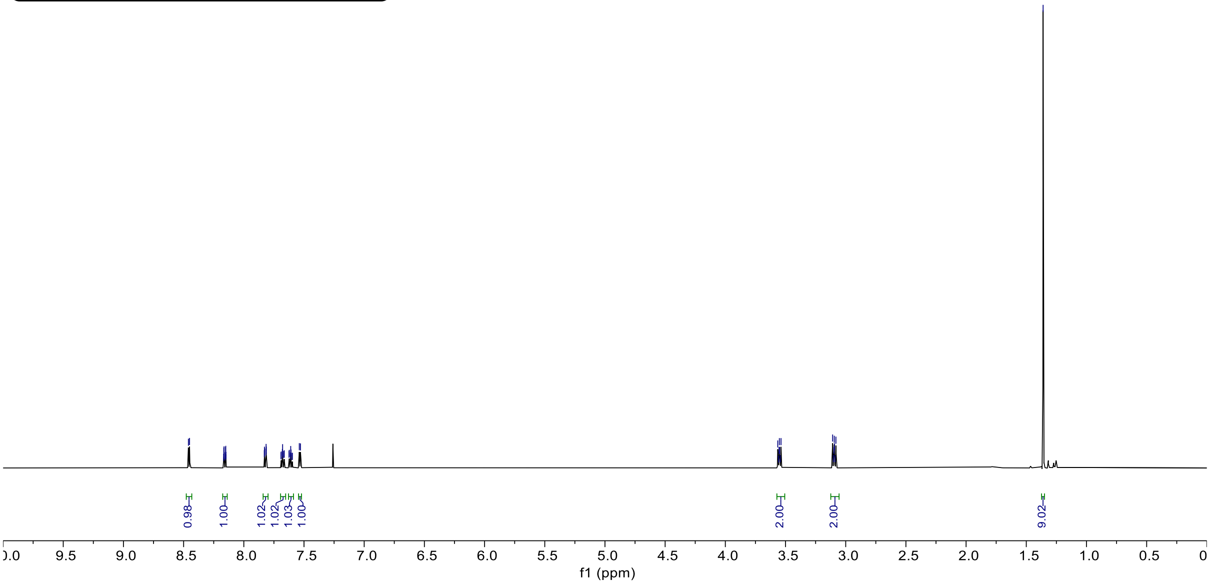

101 MHz, ^{13}C NMR in Chloroform-*d*

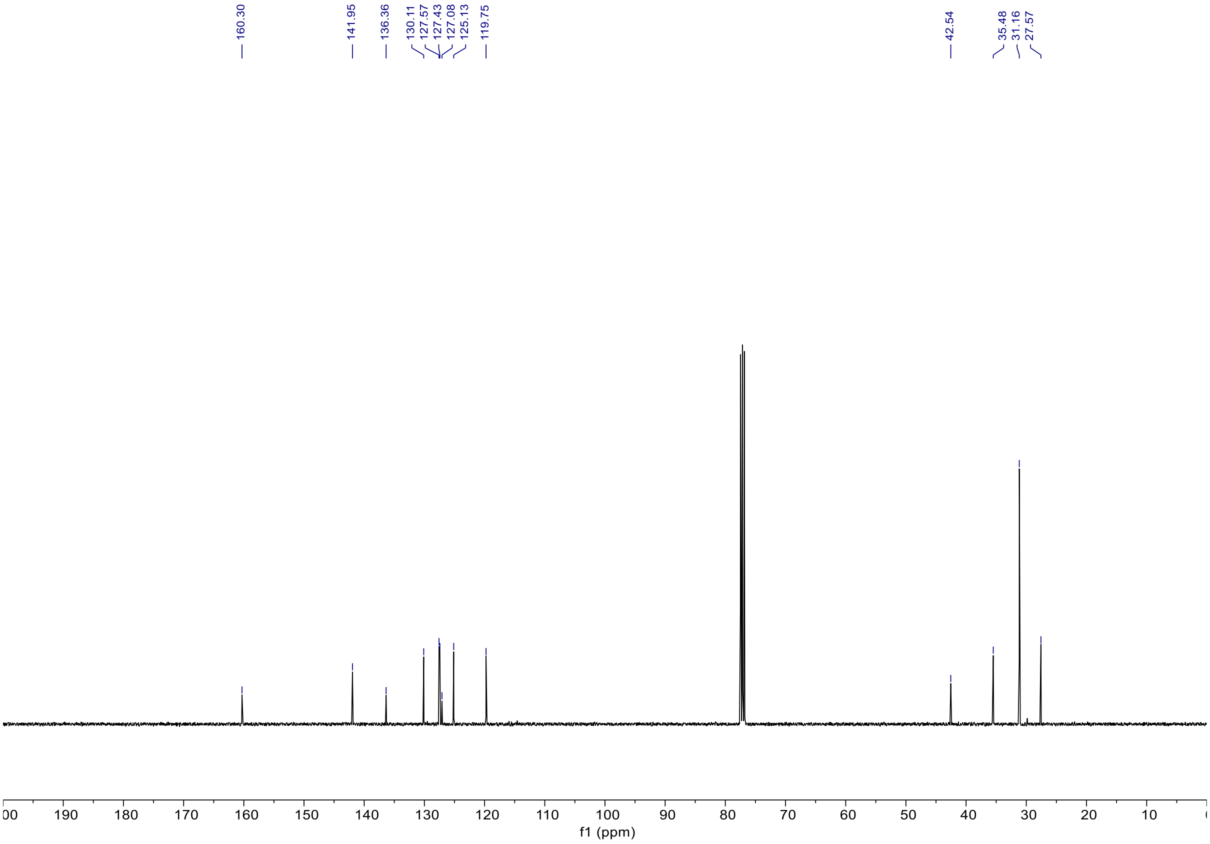

N-acetyl-S-(2-(isoquinolin-1-yl)ethyl)-L-cysteine (3v).

600 MHz, ^{1}H NMR in Chloroform-*d*

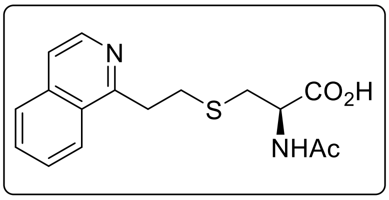

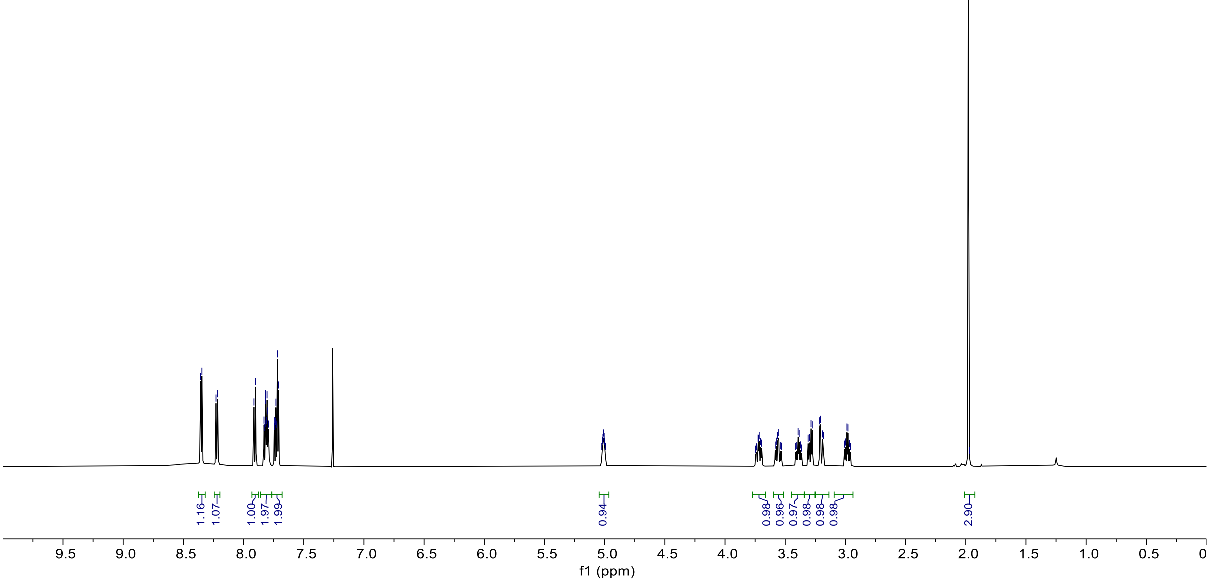

101 MHz, ^{13}C NMR in Chloroform-*d*

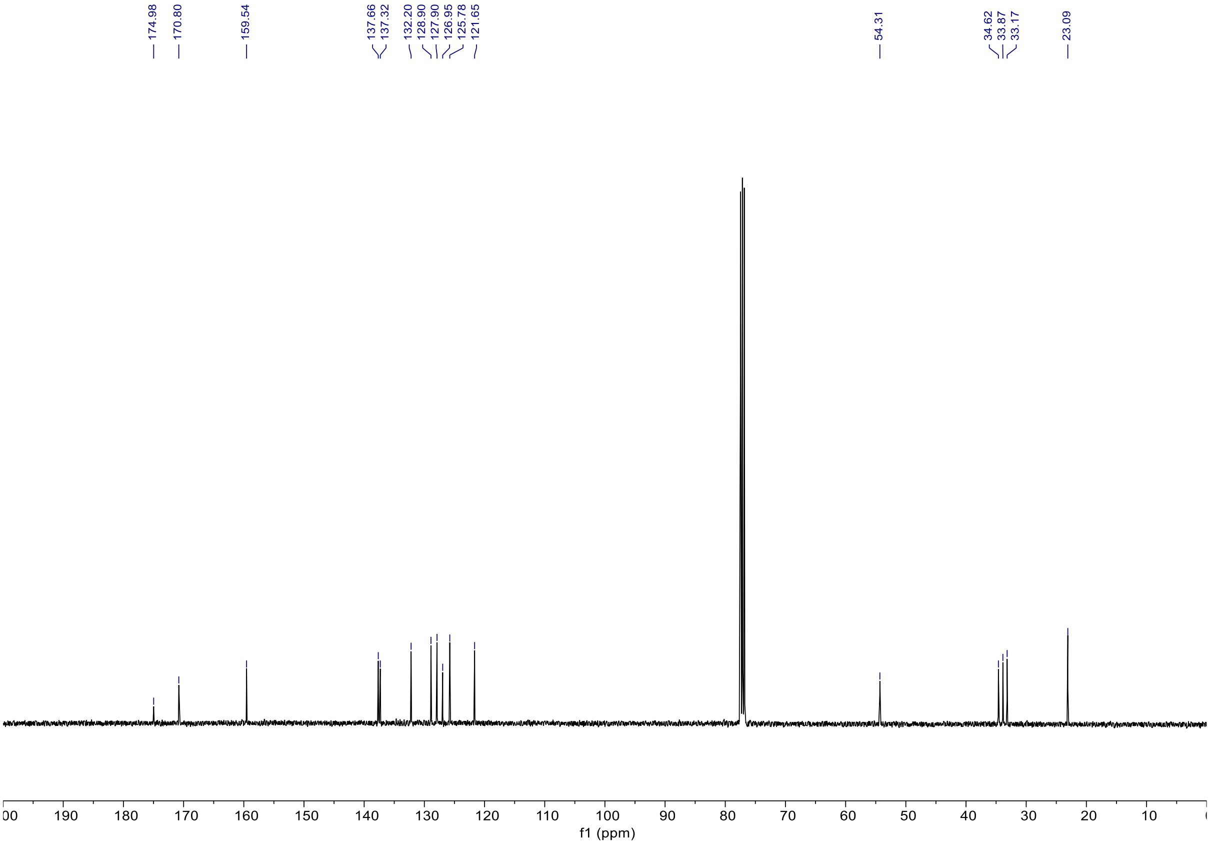

4-(2-(tert-butylthio)ethyl)-2-methylquinoline (3w).

600 MHz, ^{1}H NMR in Chloroform-*d*

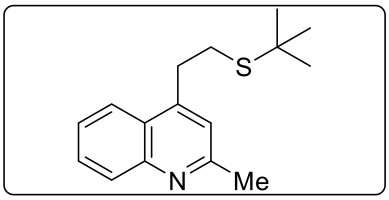

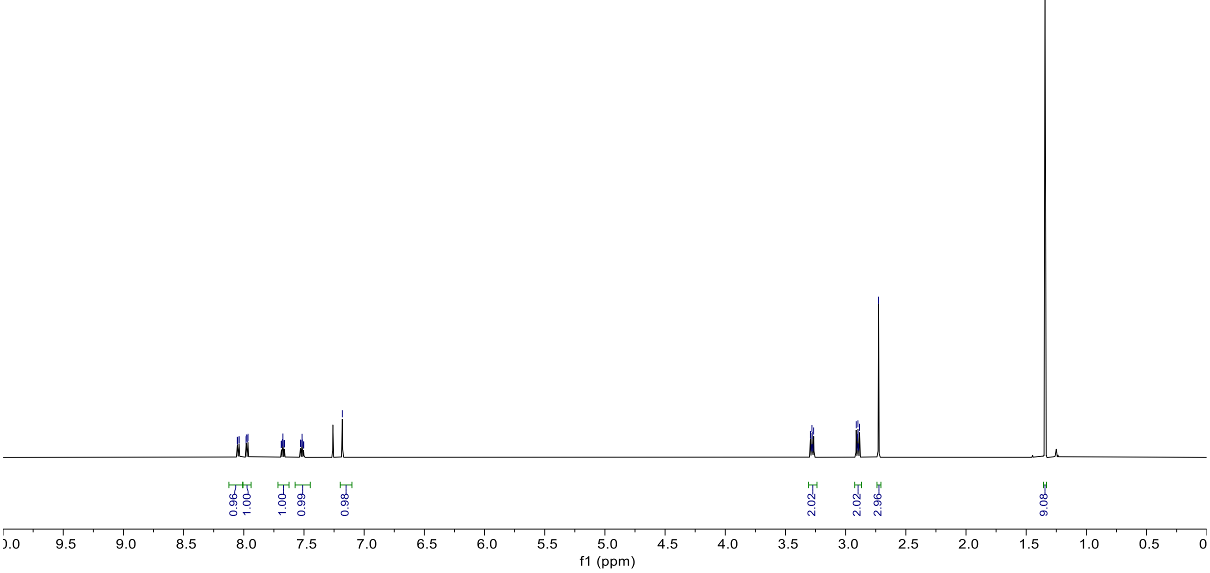

101 MHz, ^{13}C NMR in Chloroform-*d*

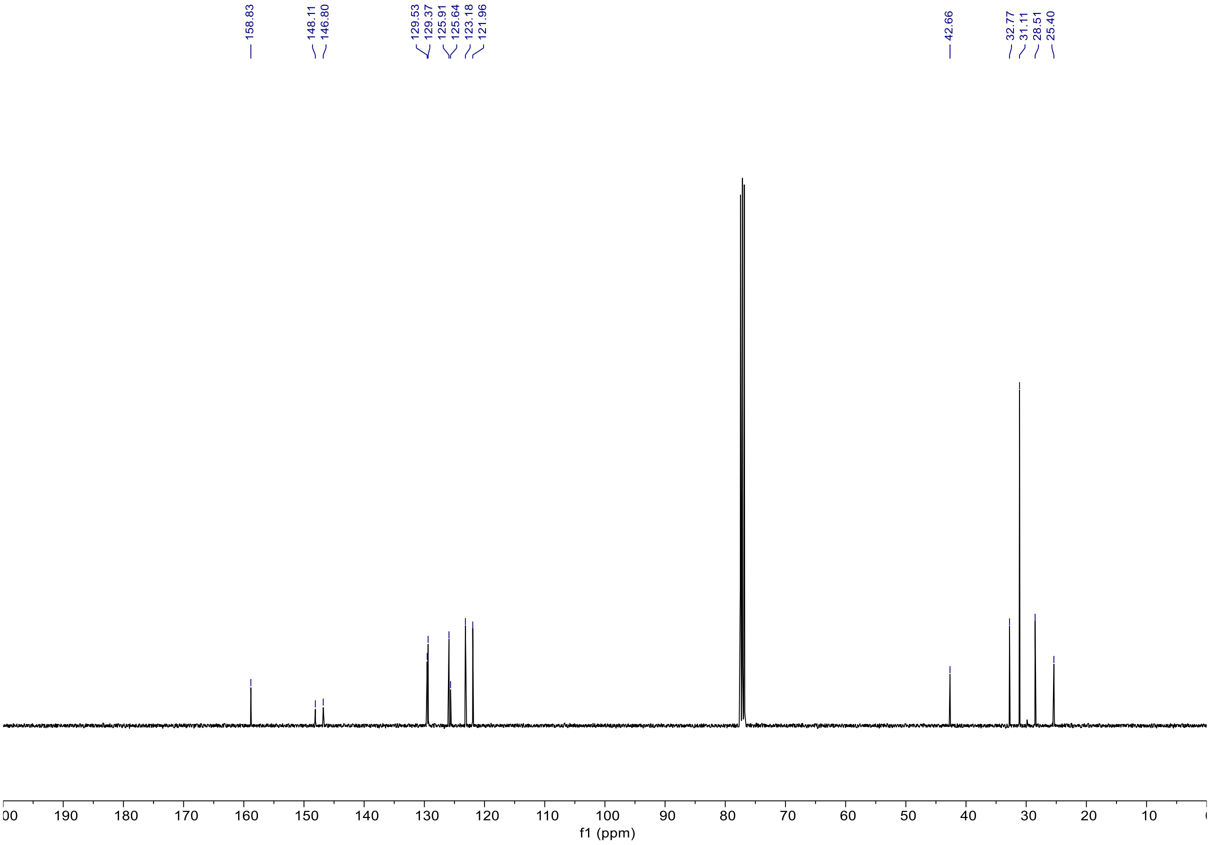

6-(2-(tert-butylthio)ethyl)phenanthridine (3x).

600 MHz, ^{1}H NMR in Chloroform-*d*

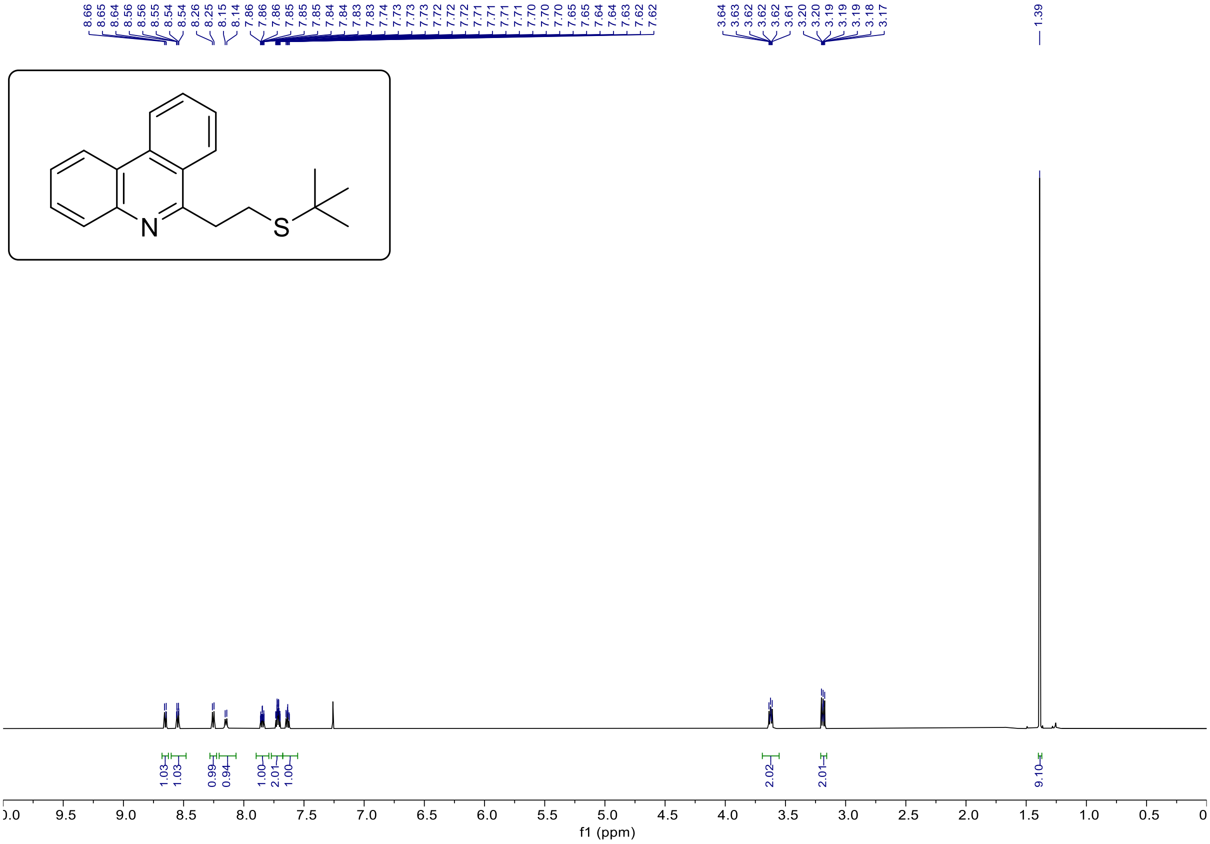

101 MHz, ^{13}C NMR in Chloroform-*d*

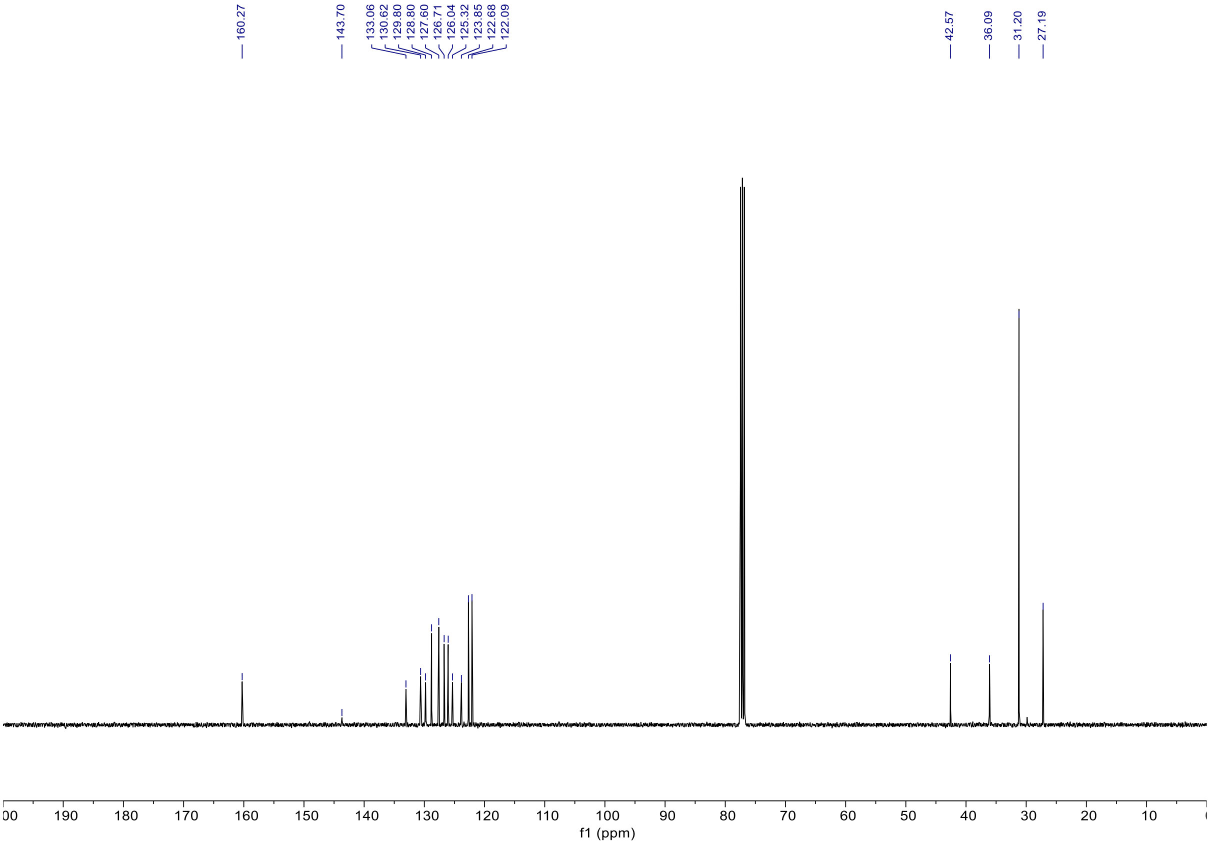

1-(4-((1-(2-((4-chlorophenyl)sulfonyl)ethyl)isoquinolin-5-yl)sulfonyl)-1,4-diazepan-1-yl)ethan-1-one (3y).

400 MHz, ^{1}H NMR in Chloroform-*d*

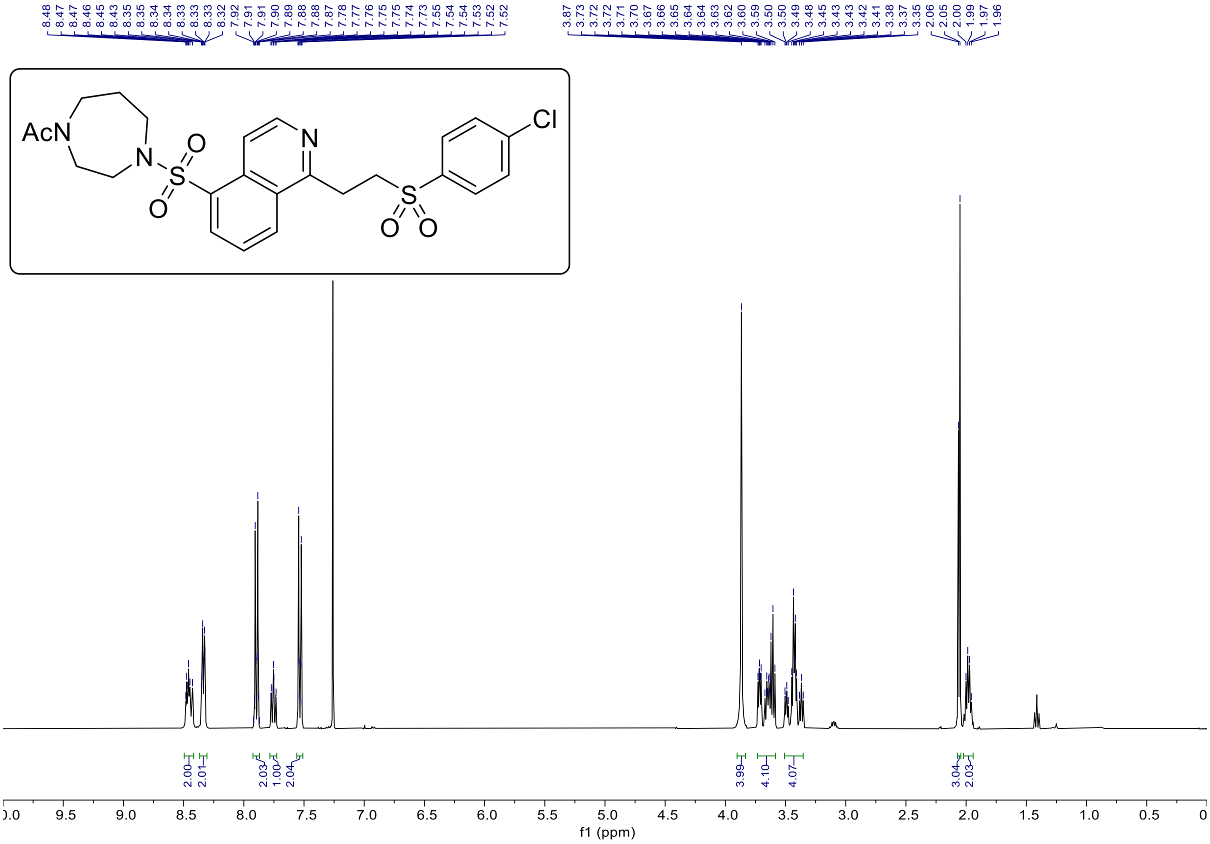

101 MHz, ^{13}C NMR in Chloroform-*d*

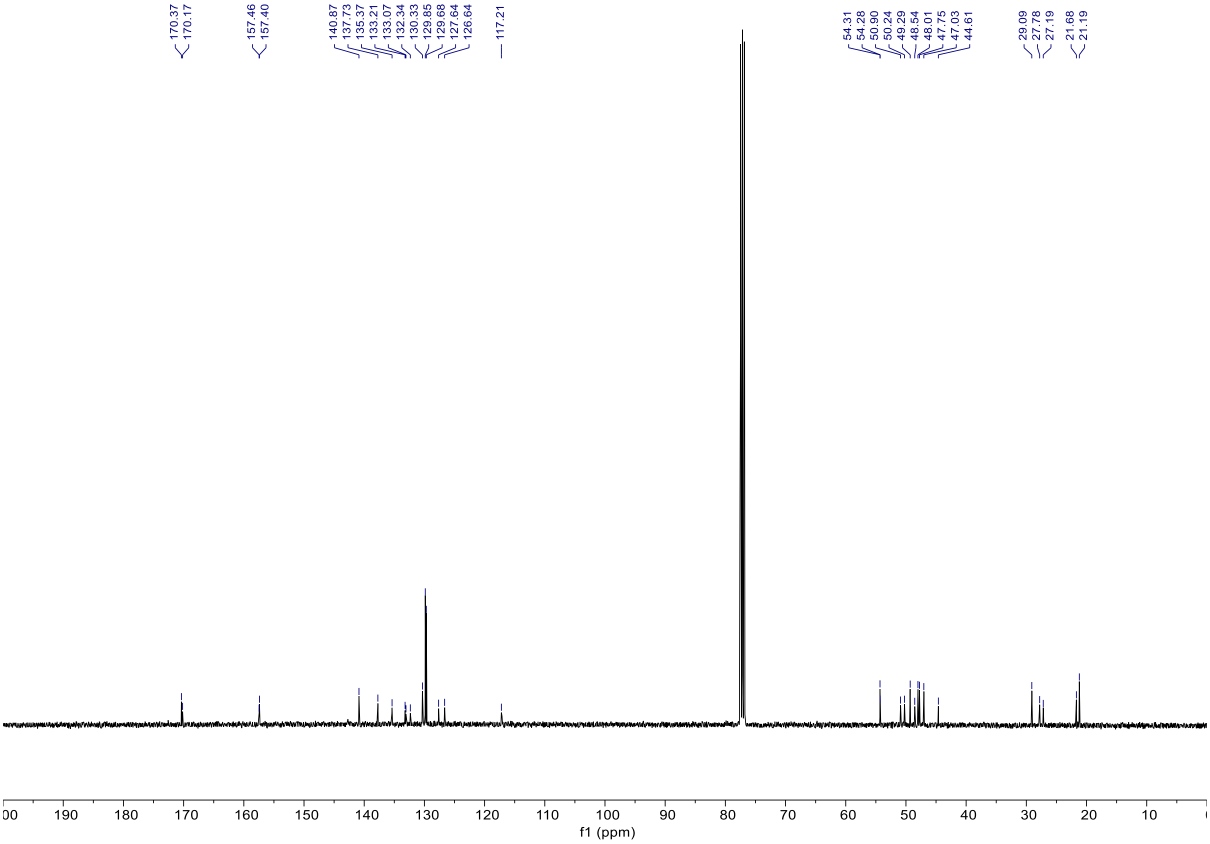

1-(4-((1-(2-(di-p-tolylphosphoryl)ethyl)isoquinolin-5-yl)sulfonyl)-1,4-diazepan-1-yl)ethan-1-one (3z).

400 MHz, ^{1}H NMR in Chloroform-*d*

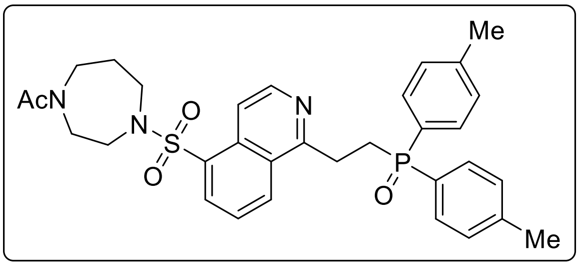

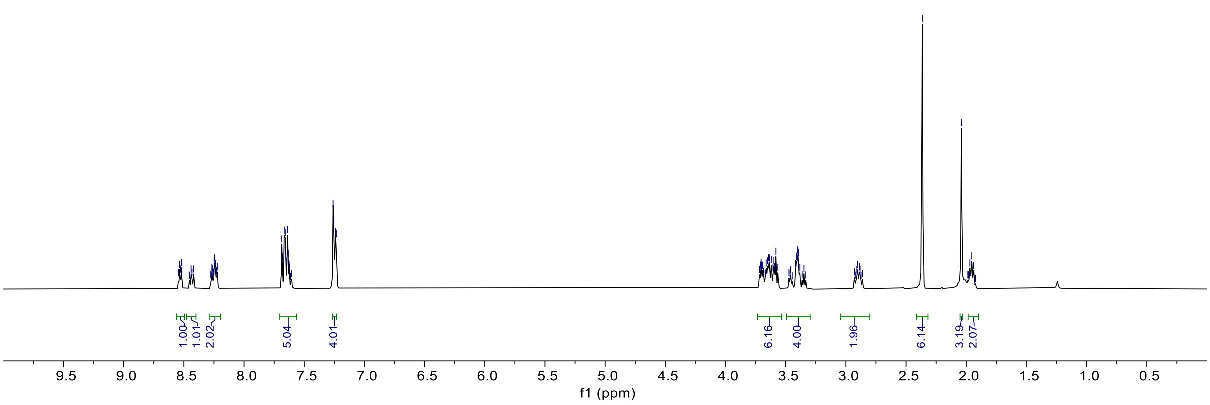

101 MHz, ^{13}C NMR in Chloroform-*d*

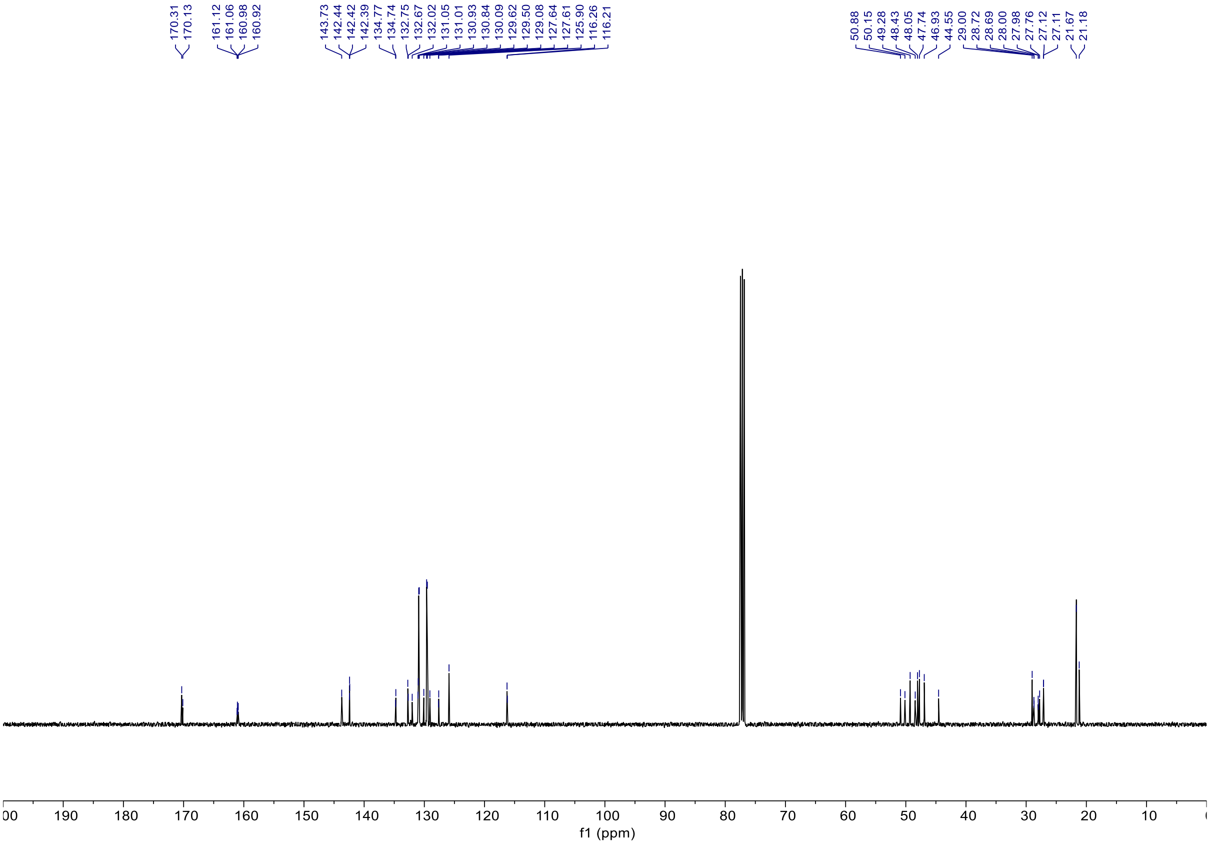

1-(4-((1-(2-(benzylthio)ethyl)isoquinolin-5-yl)sulfonyl)-1,4-diazepan-1-yl)ethan-1-one (3aa).

600 MHz, ^{1}H NMR in Chloroform-*d*

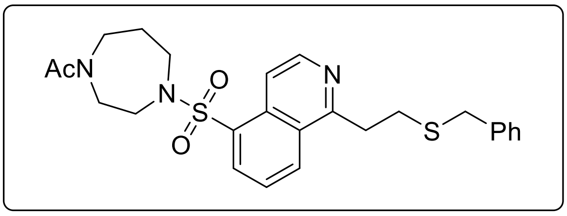

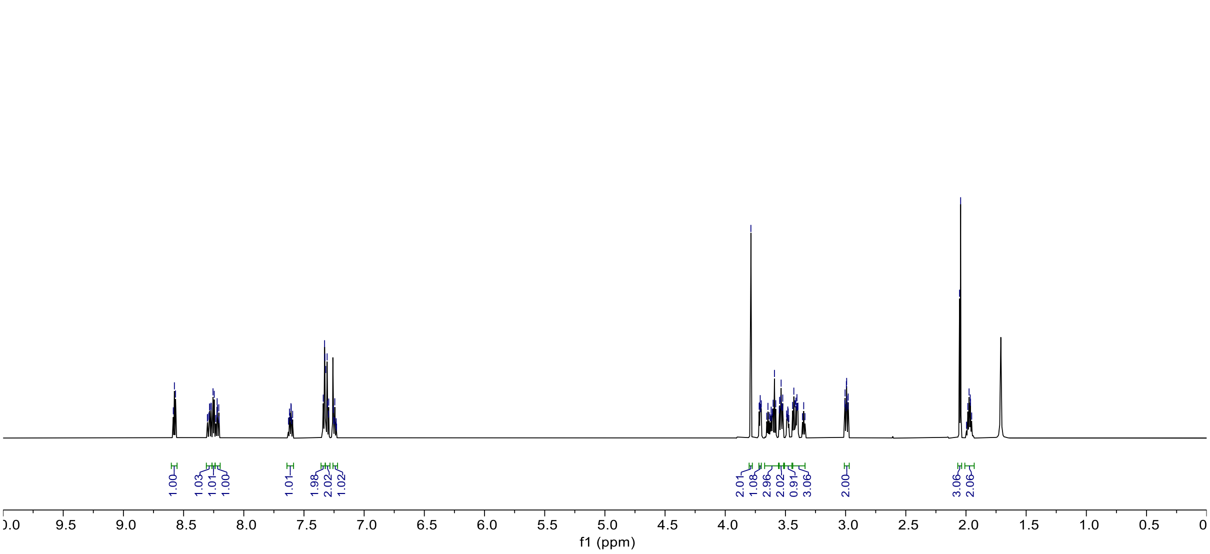

101 MHz, ^{13}C NMR in Chloroform-*d*

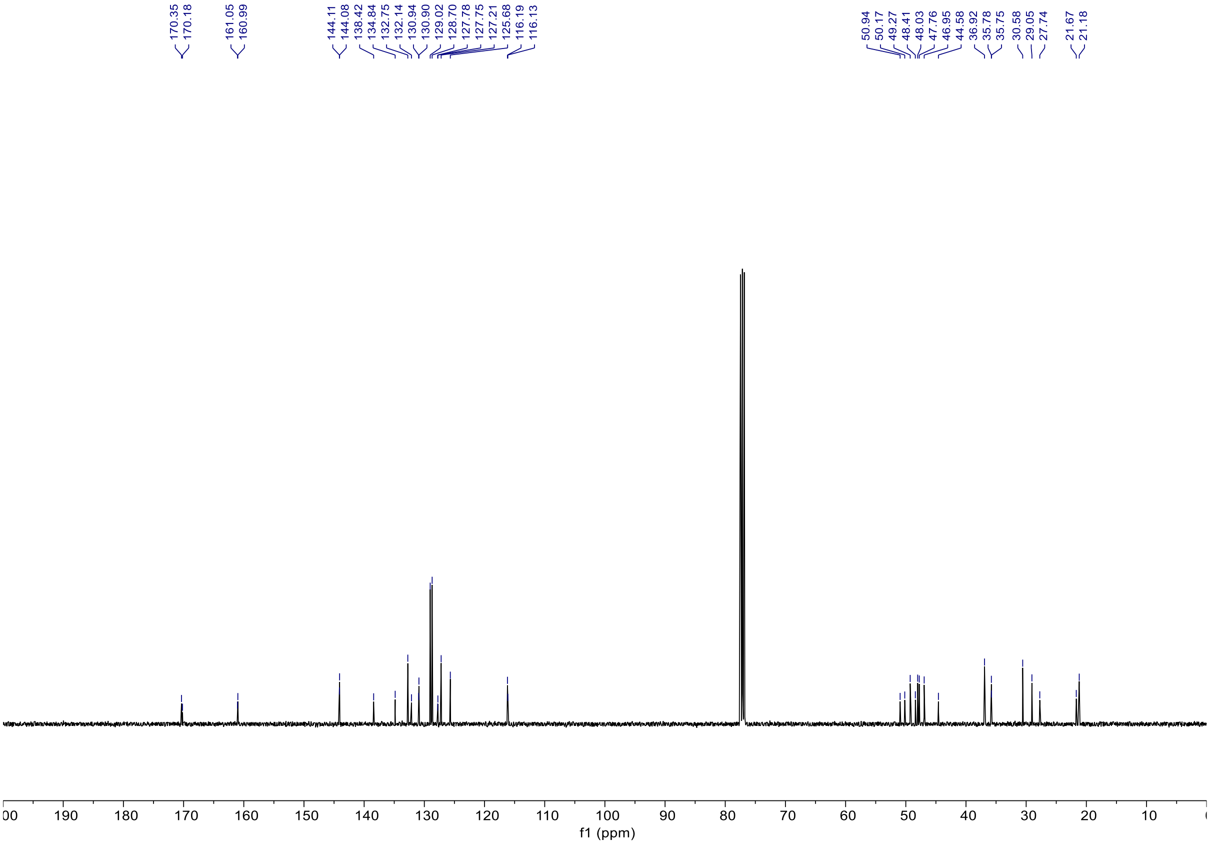

(S)-(2-(2-((4-chlorophenyl)sulfonyl)ethyl)quinolin-4-yl)((1S,2S,4S,5R)-5-vinylquinuclidin-2-yl)methanol (3ab).

400 MHz, ^{1}H NMR in Chloroform-*d*

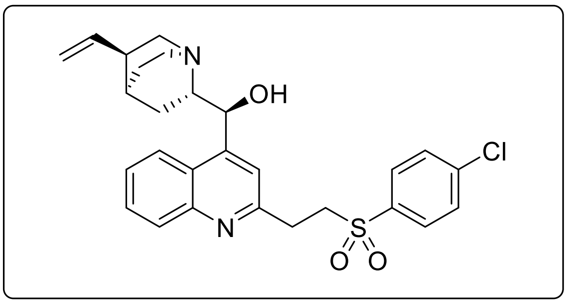

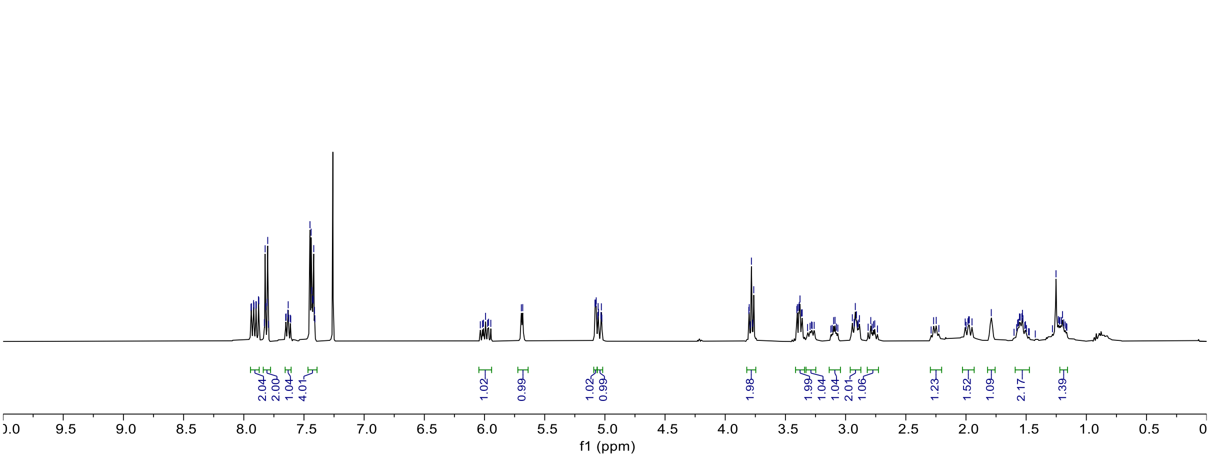

101 MHz, ^{13}C NMR in Chloroform-*d*

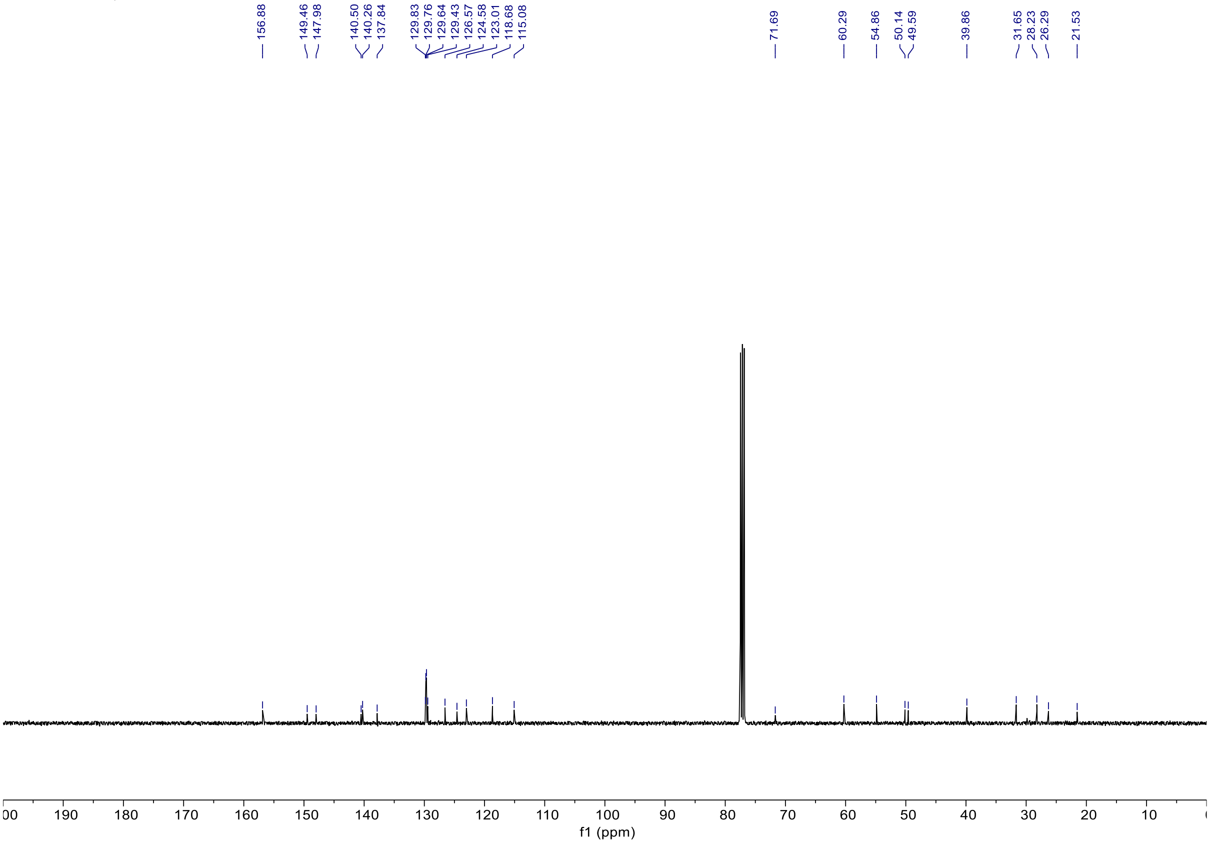

N-acetyl-L-serylglycyl-S-(2-(isoquinolin-1-yl)ethyl)-L-cysteinyl-L-phenylalanine (3ac).

600 MHz, ^{1}H NMR in Methanol-d_4

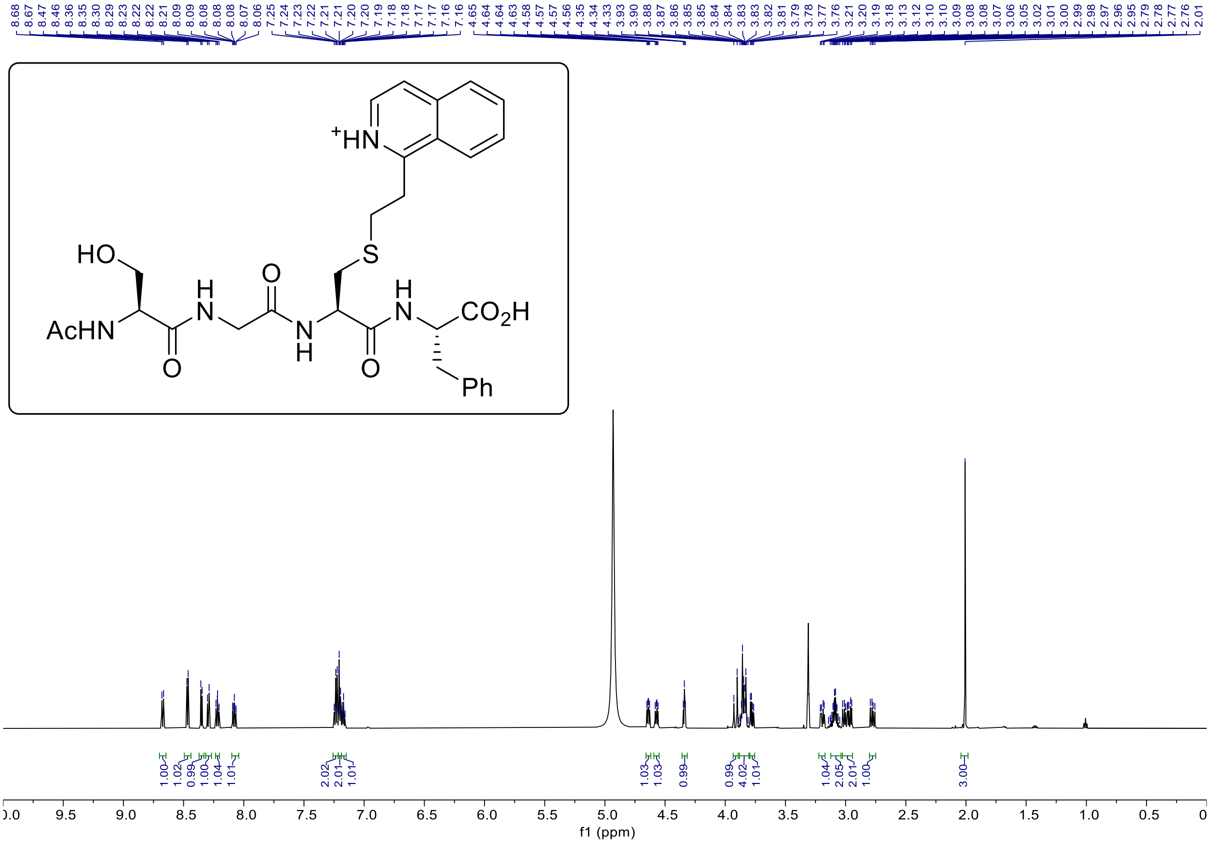

101 MHz, ^{13}C NMR in Methanol-d_4

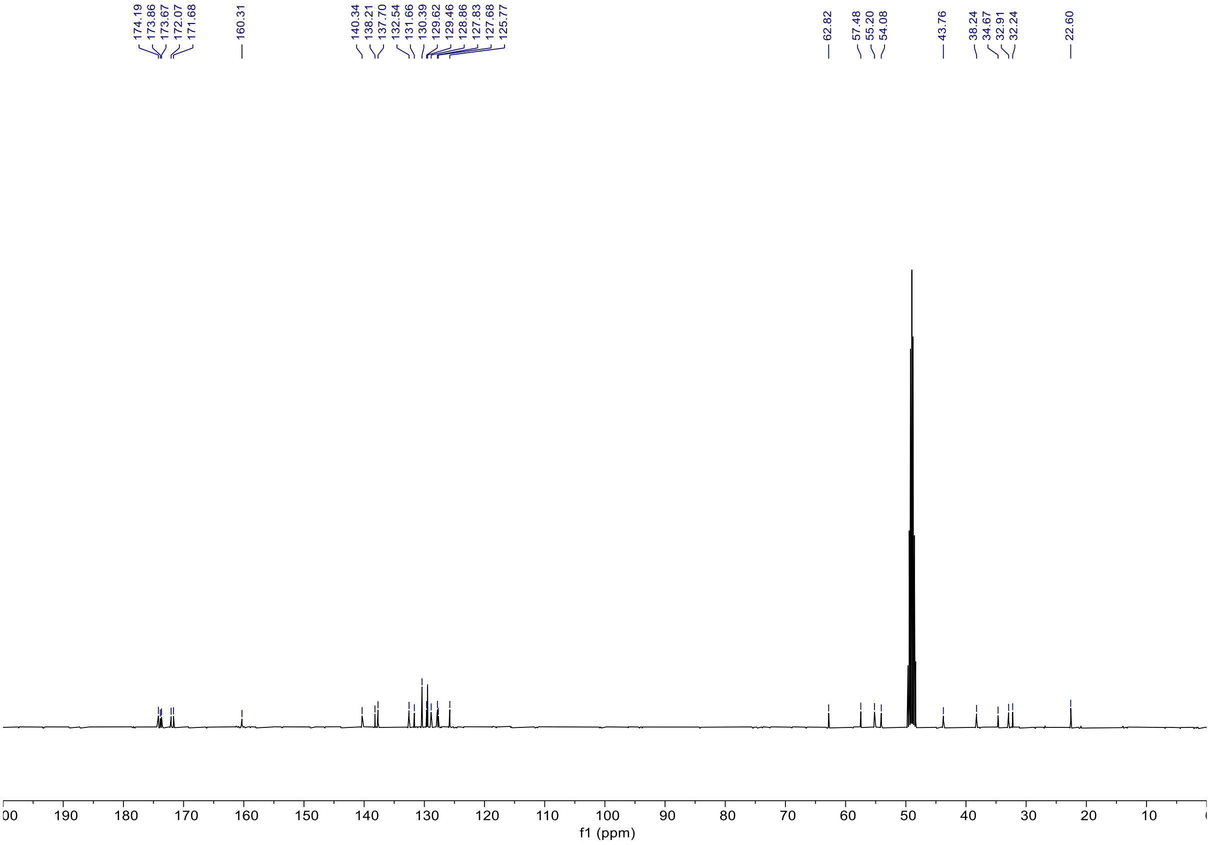

(2S,5R,11S)-2-benzyl-11-(carboxymethyl)-5-(((2-(isoquinolin-1-yl)ethyl)thio)methyl)-4,7,10,13-tetraoxo-3,6,9,12-tetraazatetradecanoic acid (3ad).

600 MHz, ^{1}H NMR in Methanol-d_4

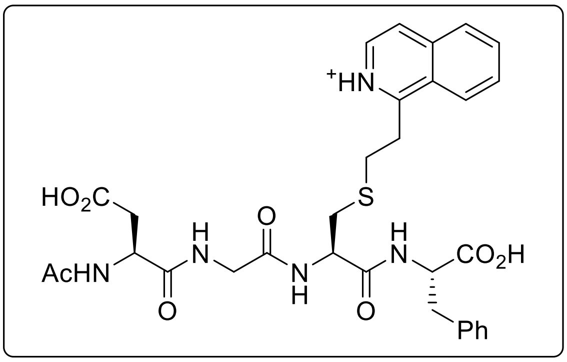

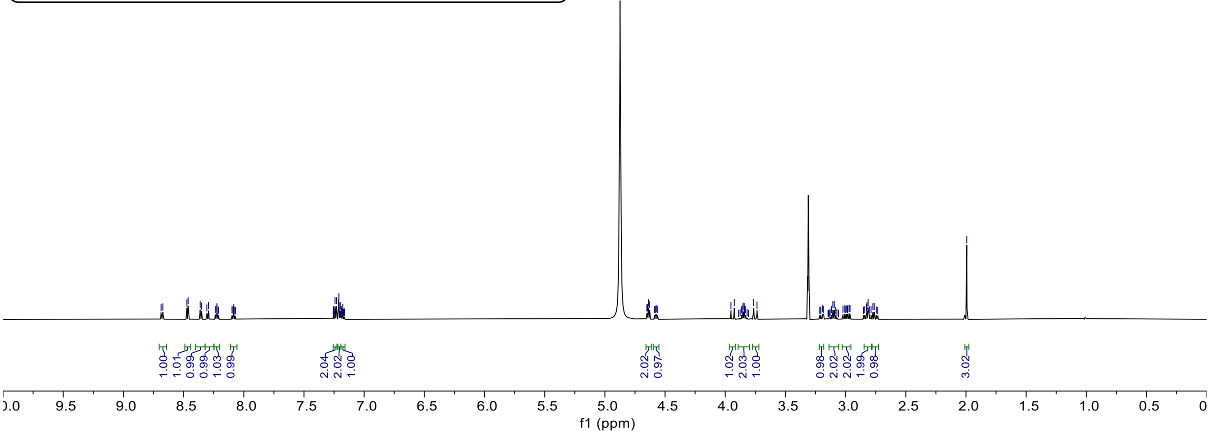

101 MHz, ^{13}C NMR in Methanol-d_4

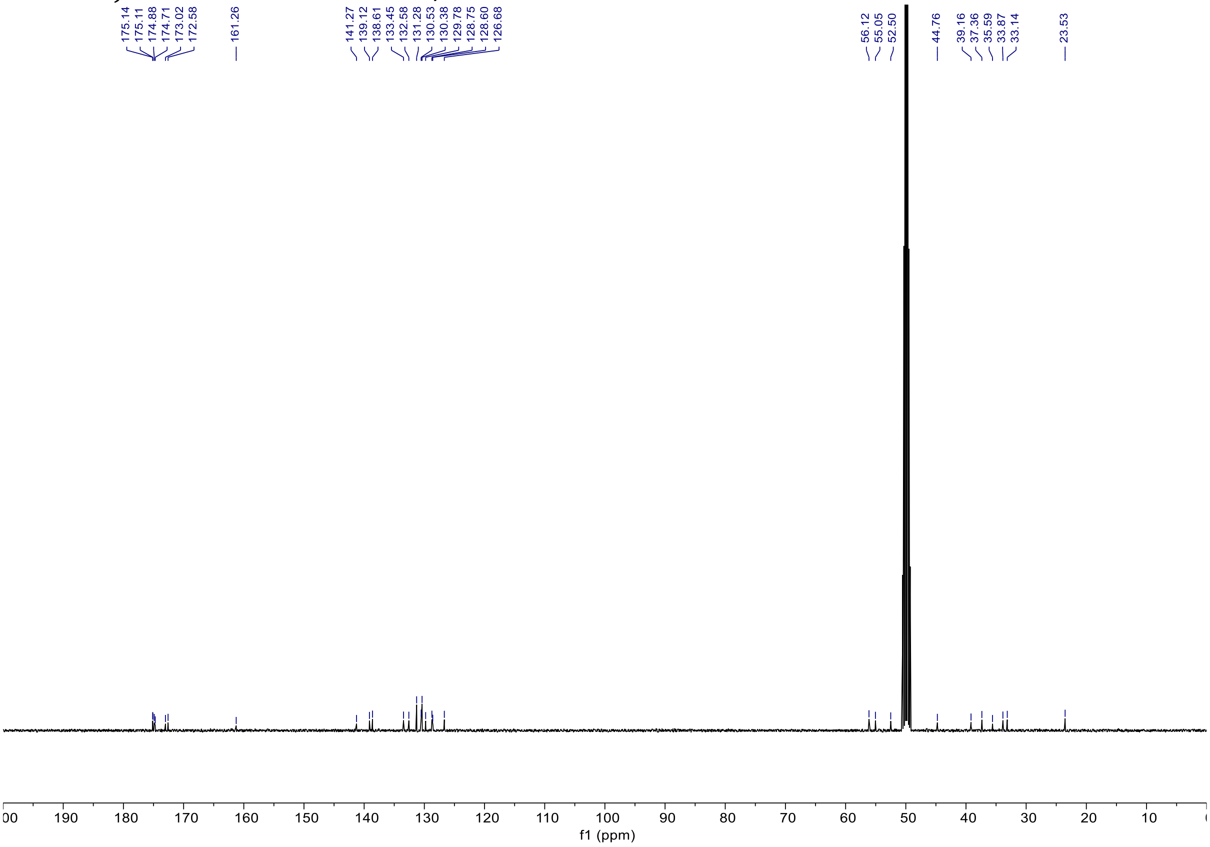

1-(2-((4-chlorophenyl)sulfonyl)propyl)isoquinoline (3ae).

500 MHz, ^{1}H NMR in Chloroform-*d*

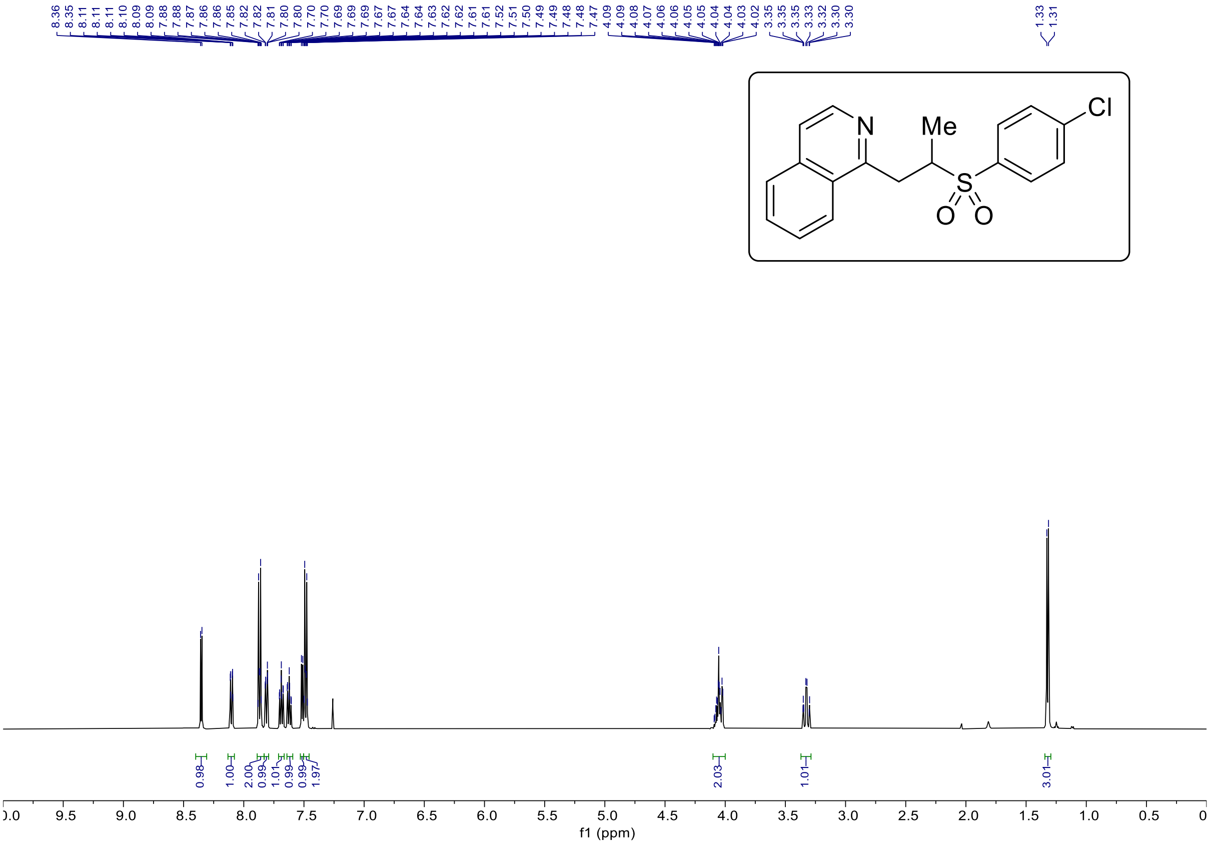

101 MHz, ^{13}C NMR in Chloroform-*d*

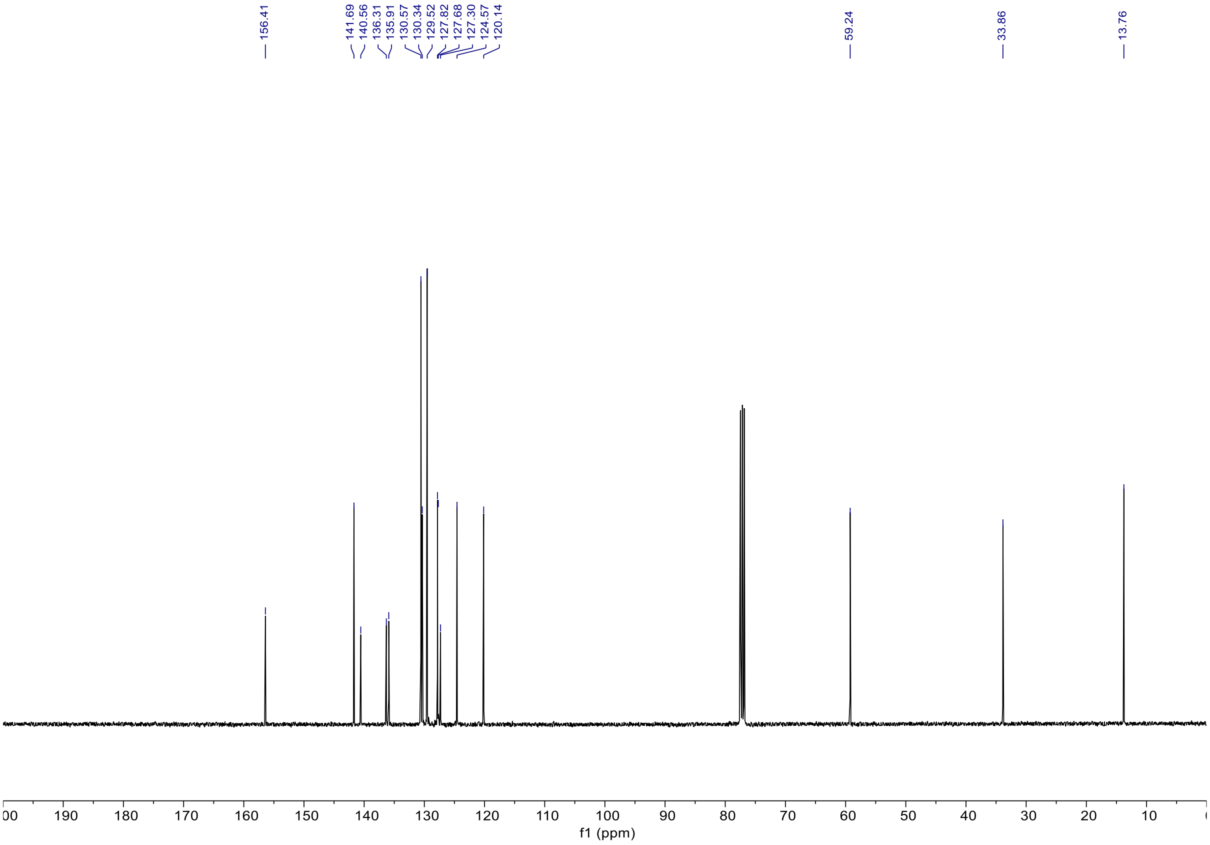

1-(2-((4-chlorophenyl)sulfonyl)-1-phenoxyethyl)isoquinoline (3a’).

600 MHz, ^{1}H NMR in Chloroform-*d*

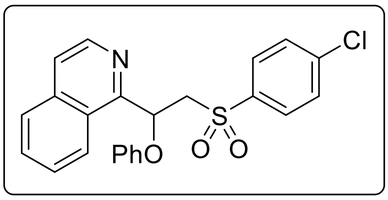

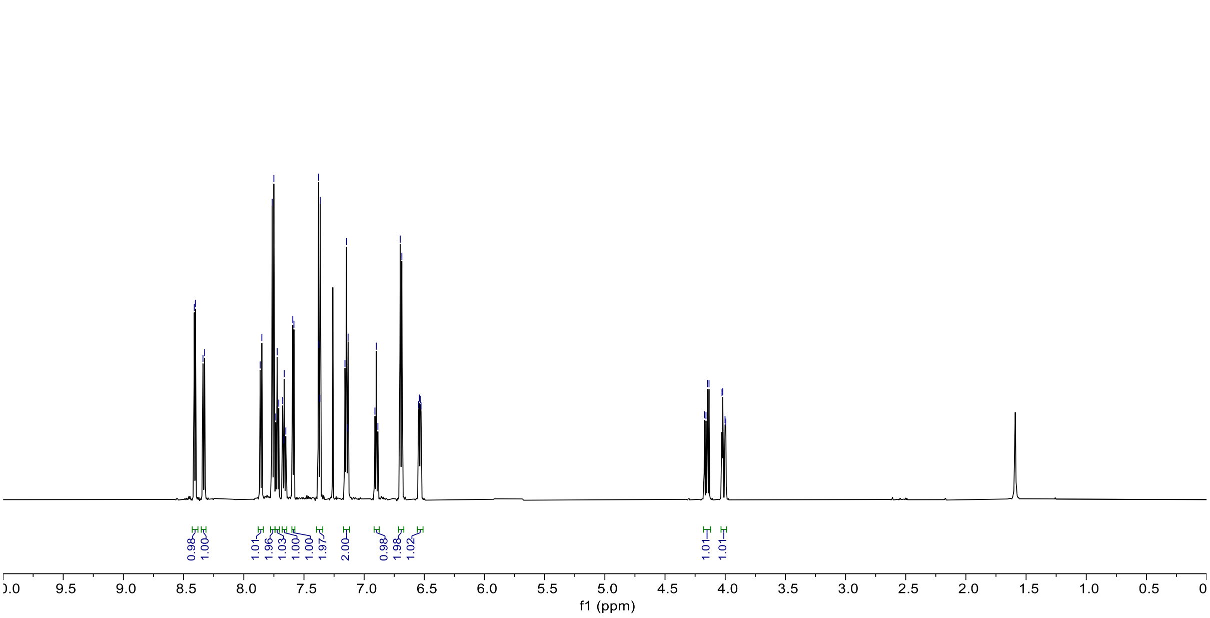

101 MHz, ^{13}C NMR in Chloroform-*d*

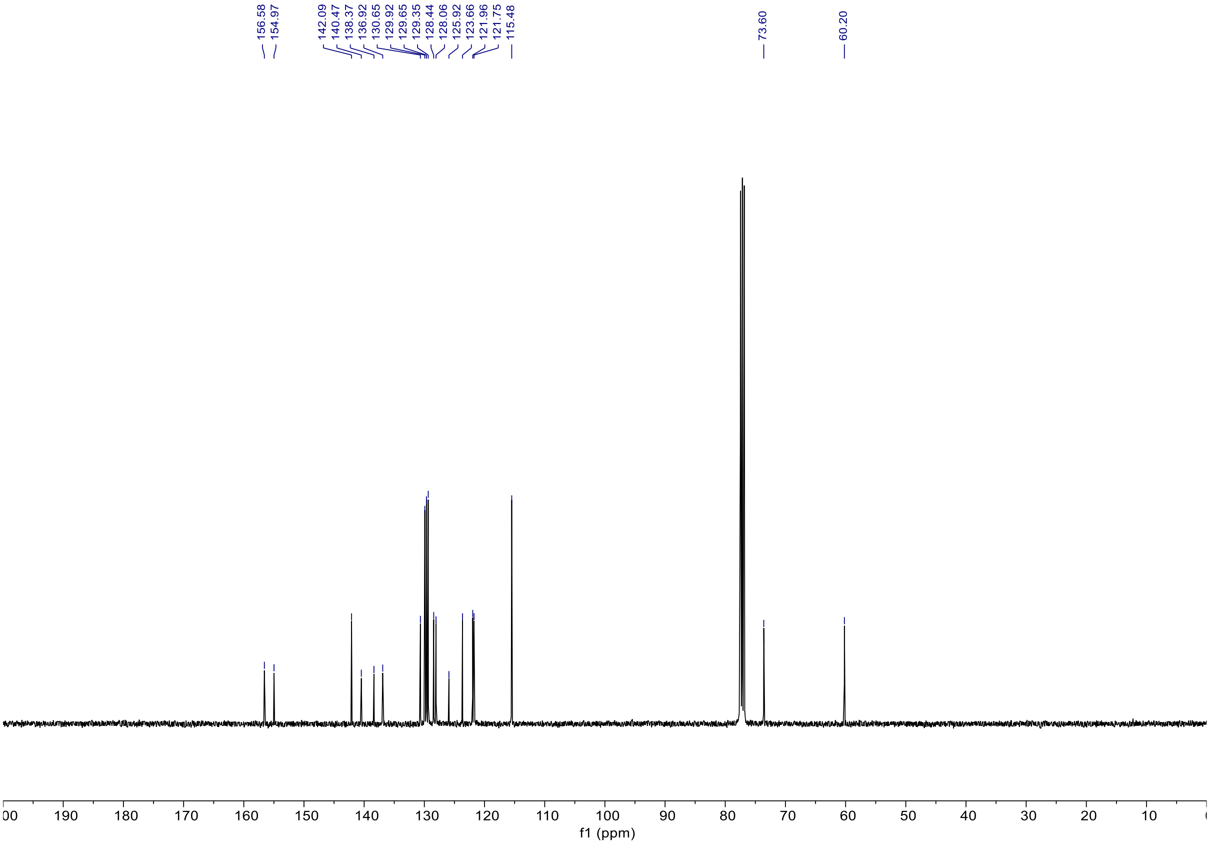

1-vinylisoquinoline (4a).

400 MHz, ^{1}H NMR in Chloroform-*d*

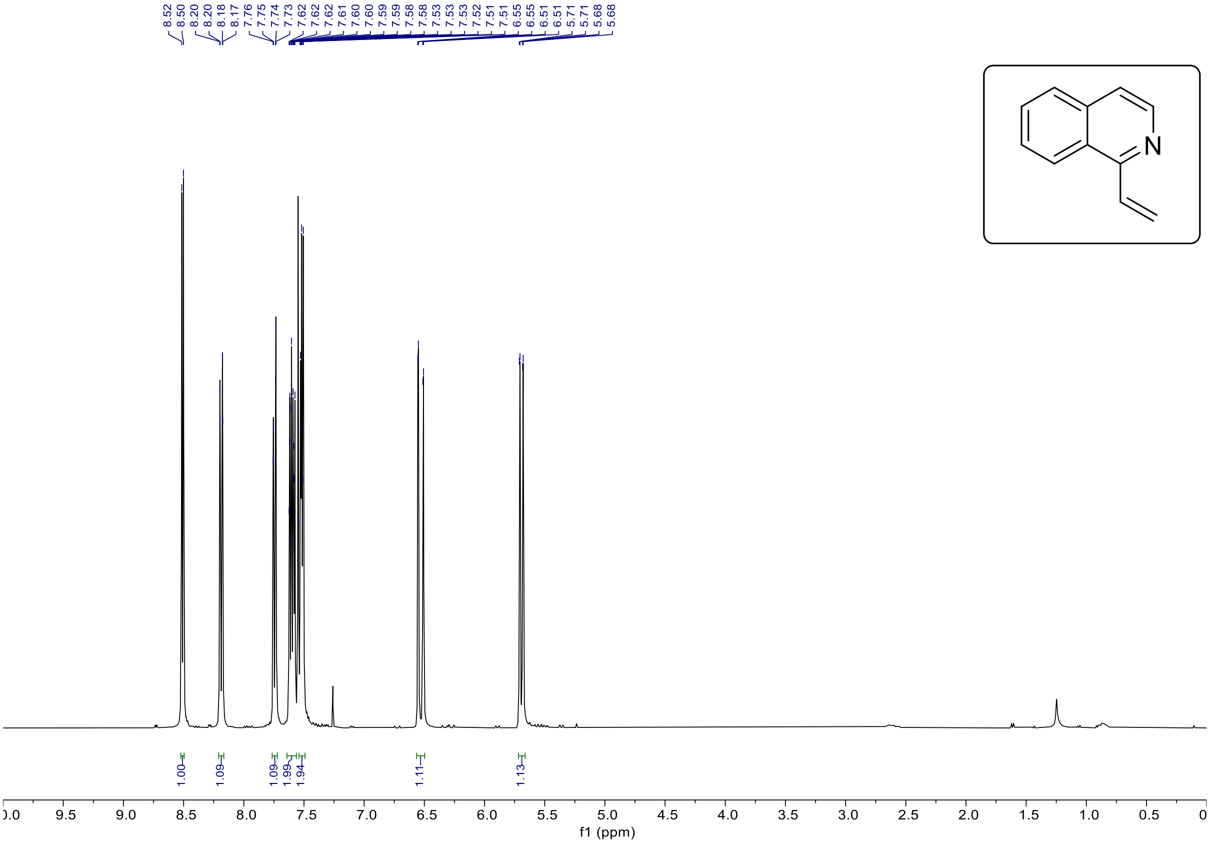

101 MHz, ^{13}C NMR in Chloroform-*d*

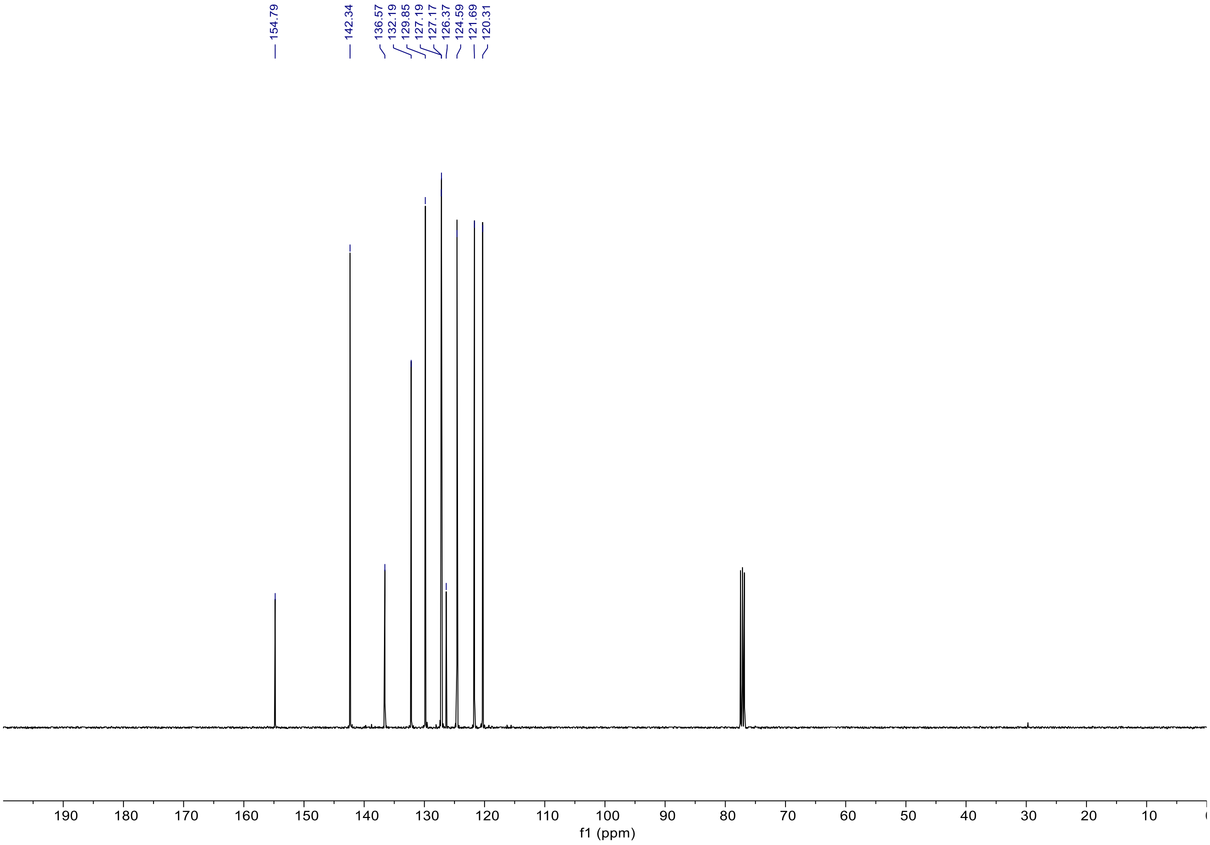

3-methyl-1-vinylisoquinoline (4b).

400 MHz, ^{1}H NMR in Chloroform-*d*

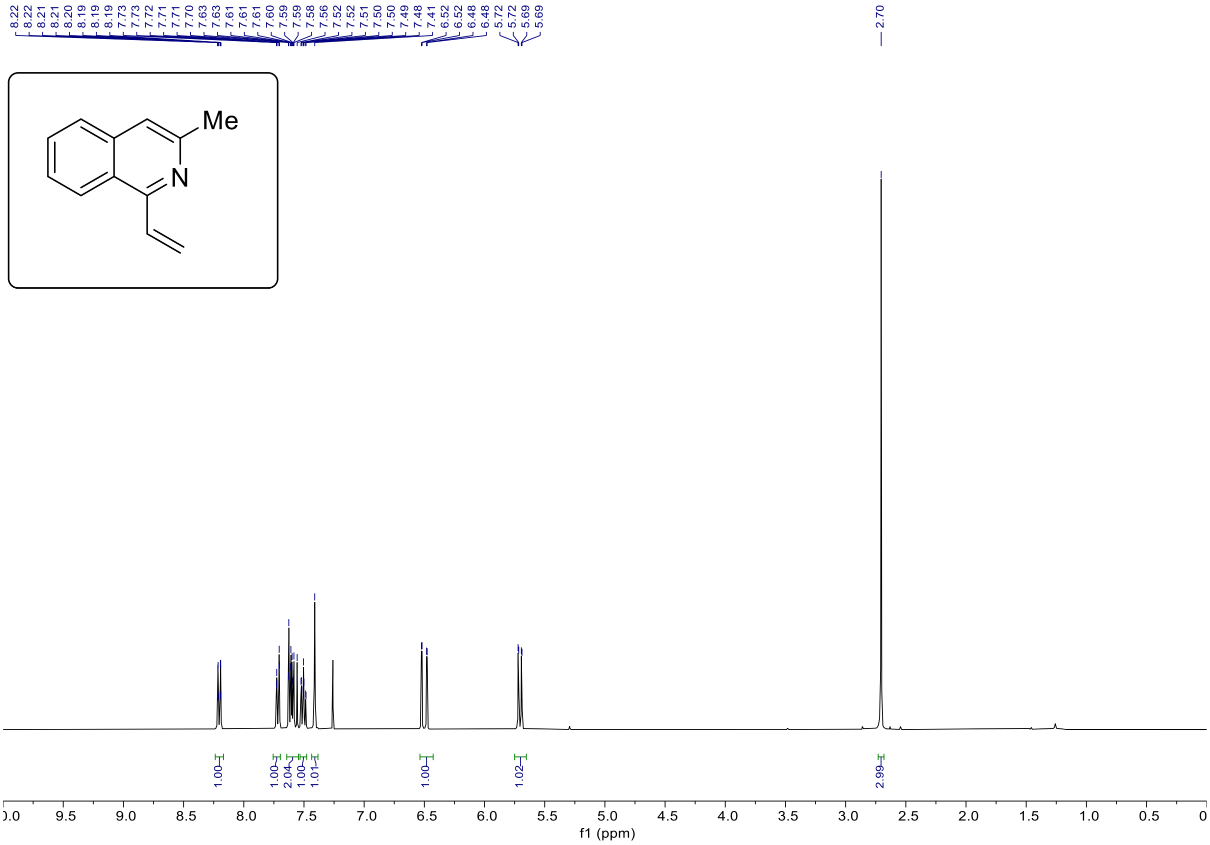

101 MHz, ^{13}C NMR in Chloroform-*d*

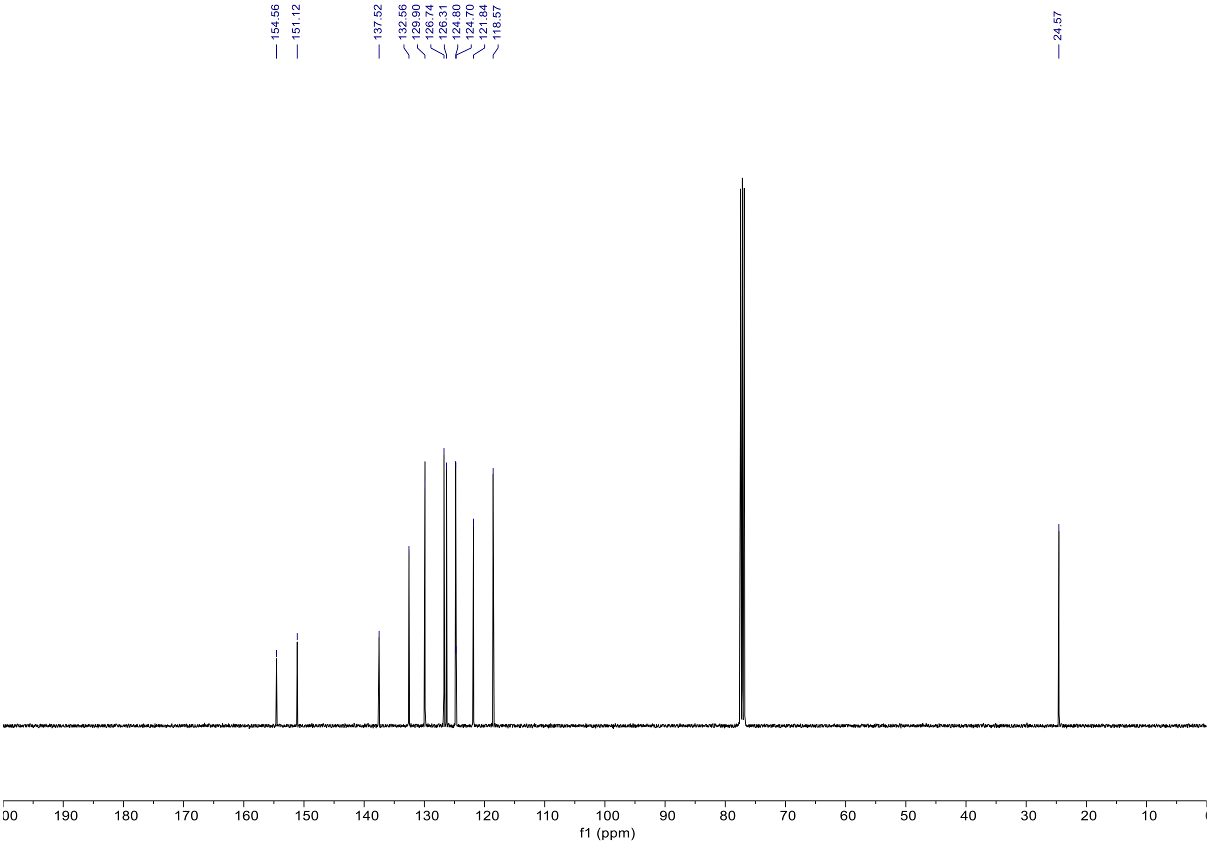

3-phenyl-1-vinylisoquinoline (4c).

600 MHz, ^{1}H NMR in Chloroform-*d*

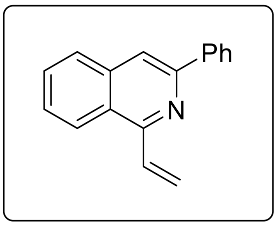

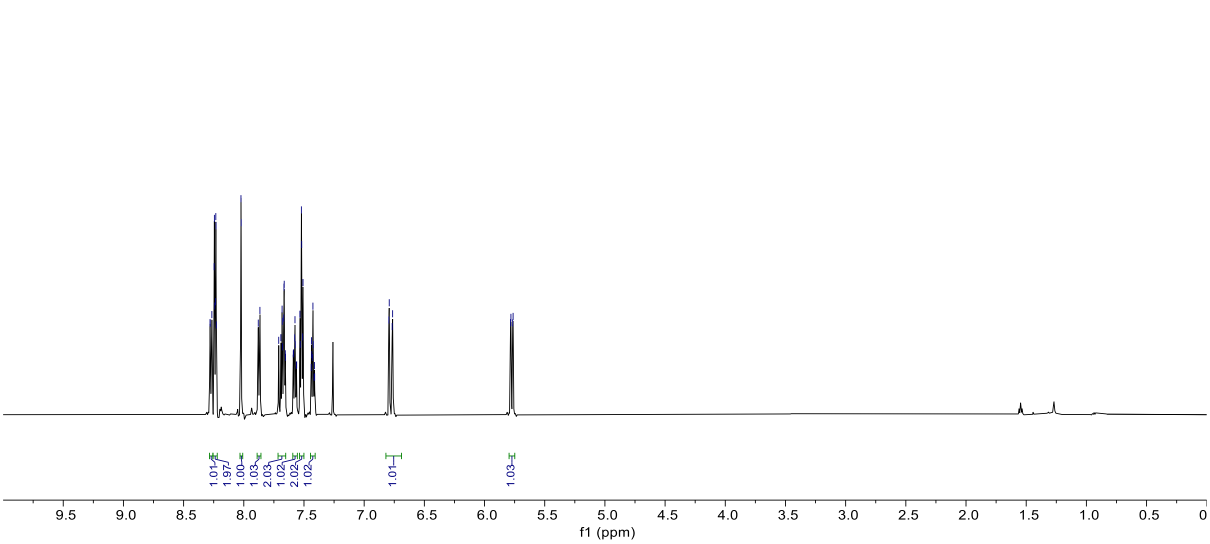

101 MHz, ^{13}C NMR in Chloroform-*d*

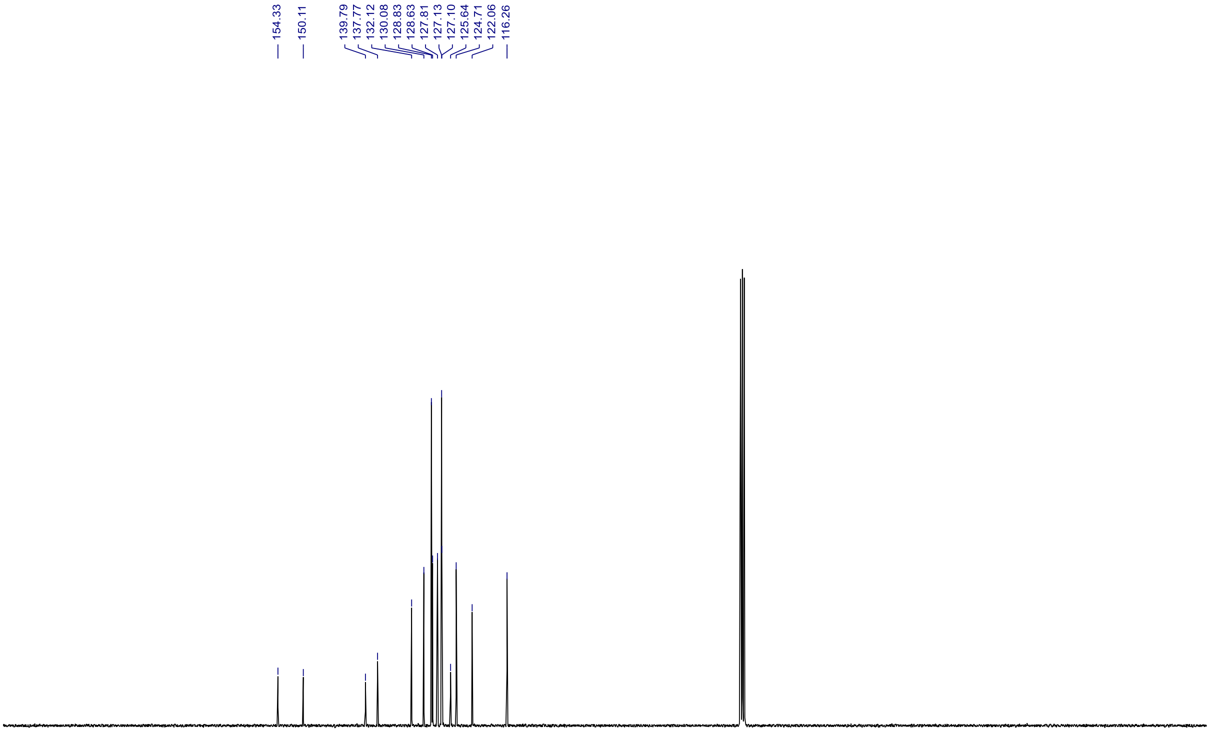

methyl 1-vinylisoquinoline-3-carboxylate (4d).

600 MHz, ^{1}H NMR in Chloroform-*d*

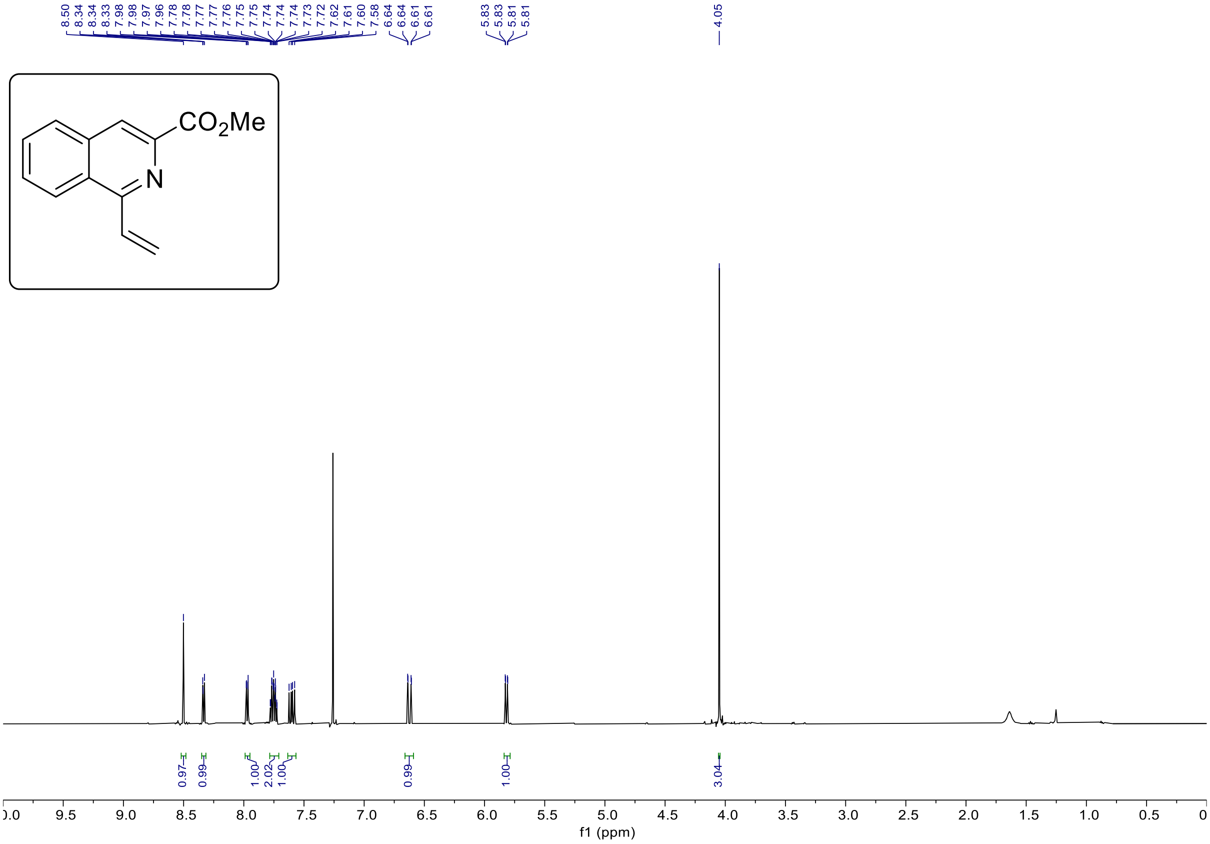

101 MHz, ^{13}C NMR in Chloroform-*d*

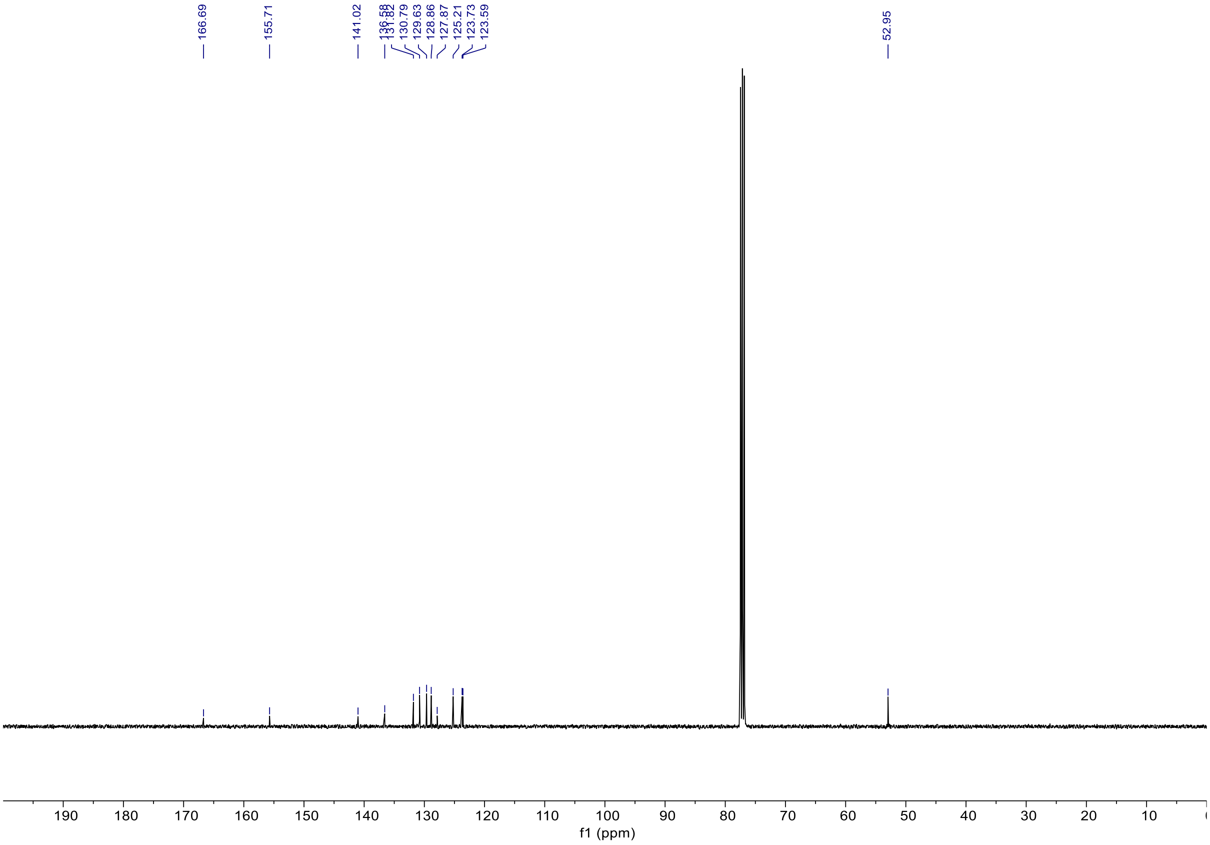

6-methyl-1-vinylisoquinoline (4e).

600 MHz, ^{1}H NMR in Chloroform-*d*

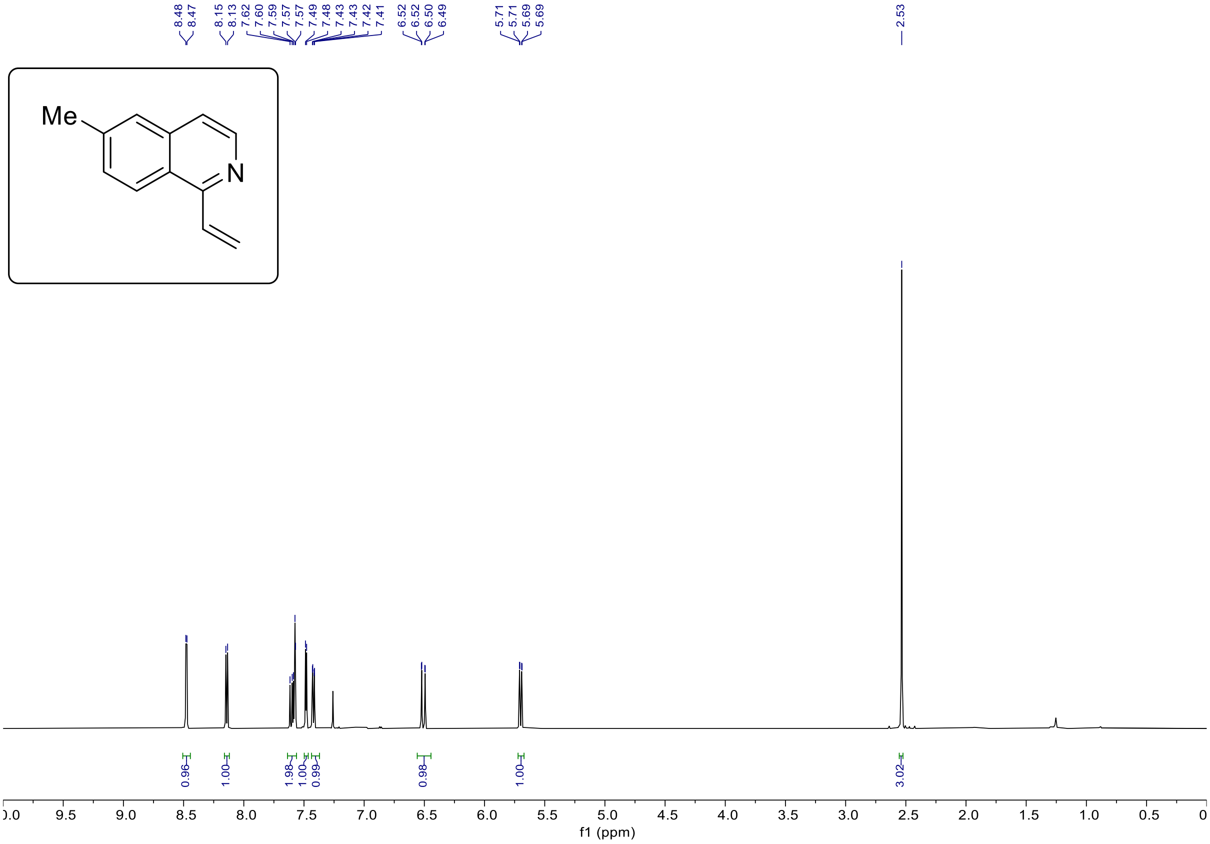

101 MHz, ^{13}C NMR in Chloroform-*d*

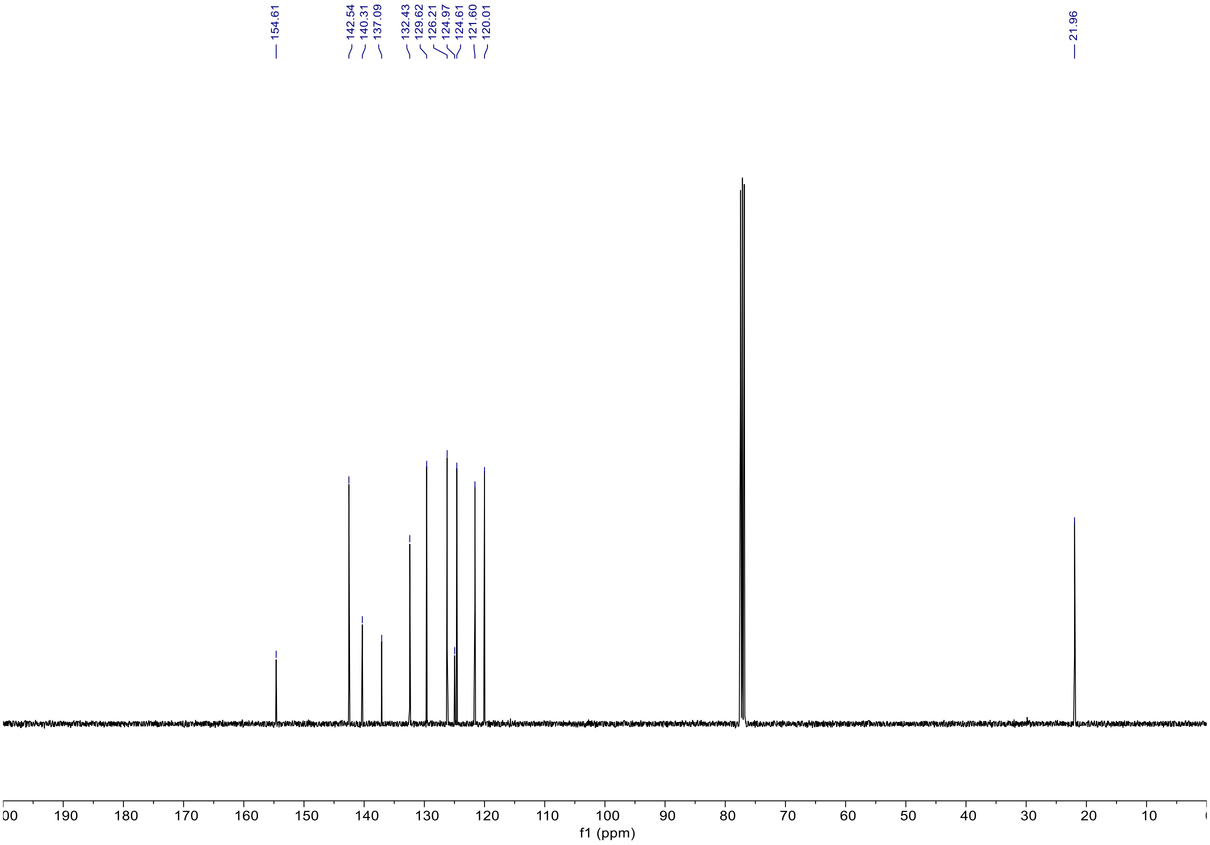

1-vinylisoquinoline-6-carbonitrile (4f).

400 MHz, ^{1}H NMR in Chloroform-*d*

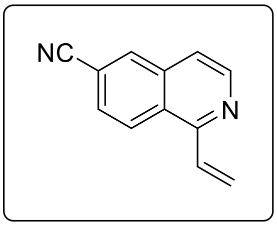

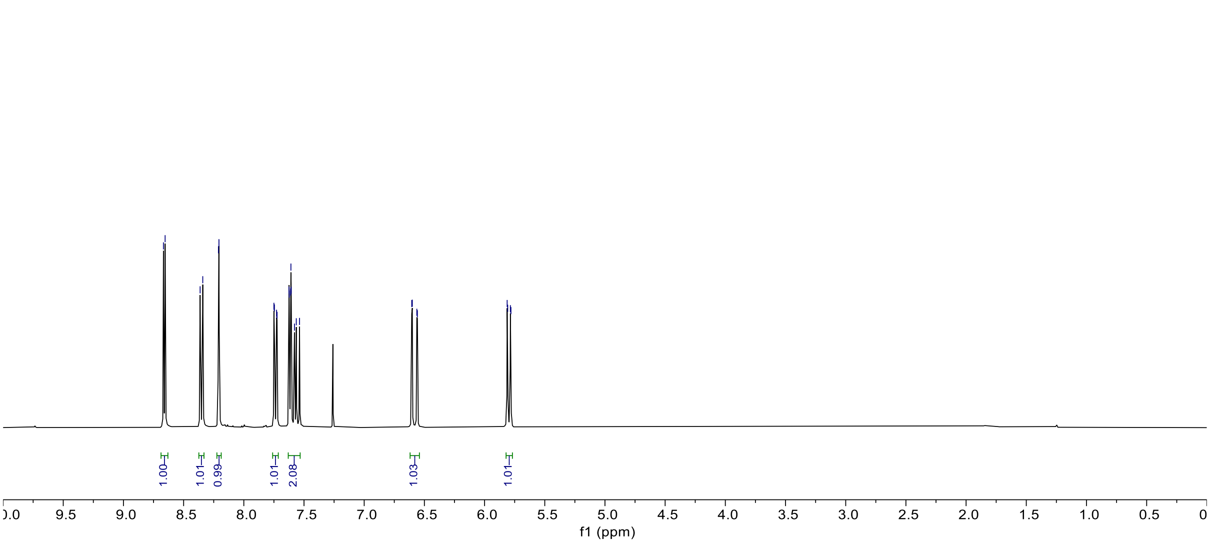

101 MHz, ^{13}C NMR in Chloroform-*d*

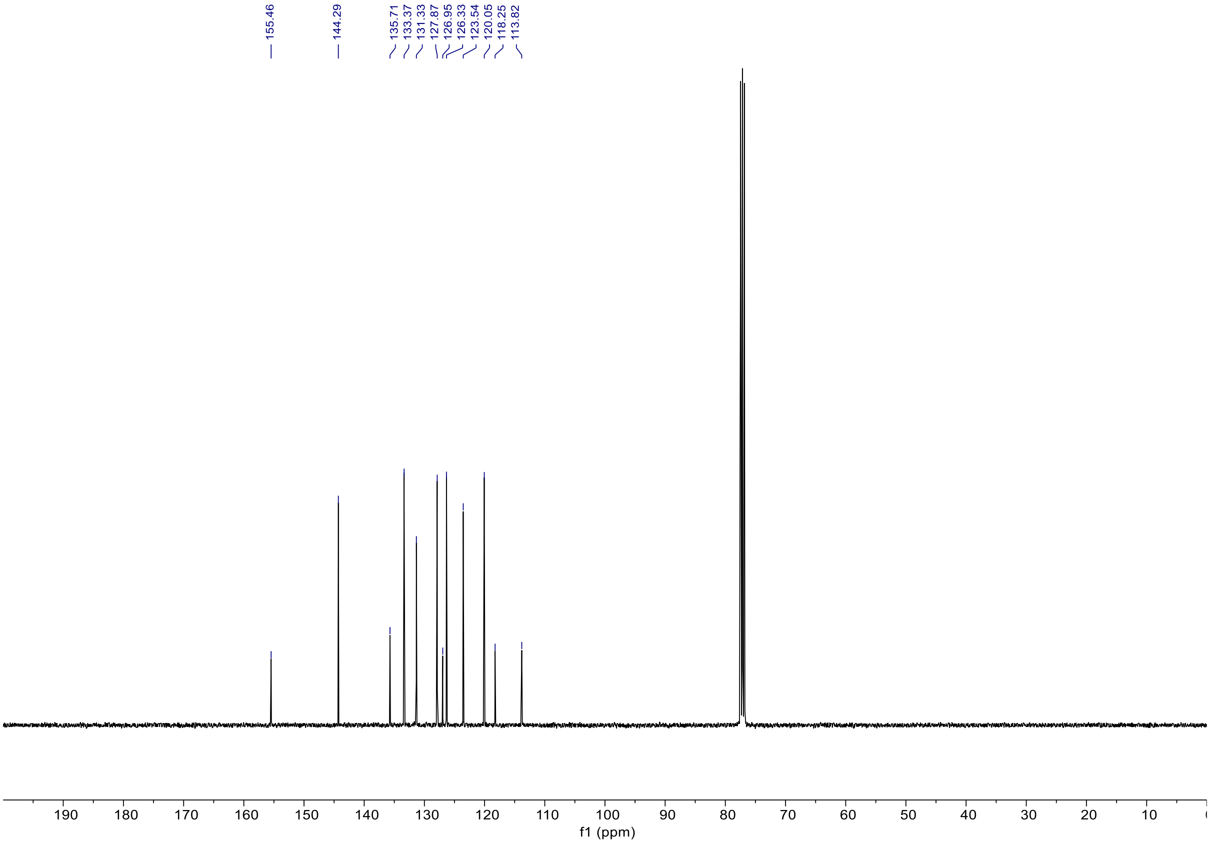

6-methoxy-1-vinylisoquinoline (4g).

600 MHz, ^{1}H NMR in Chloroform-*d*

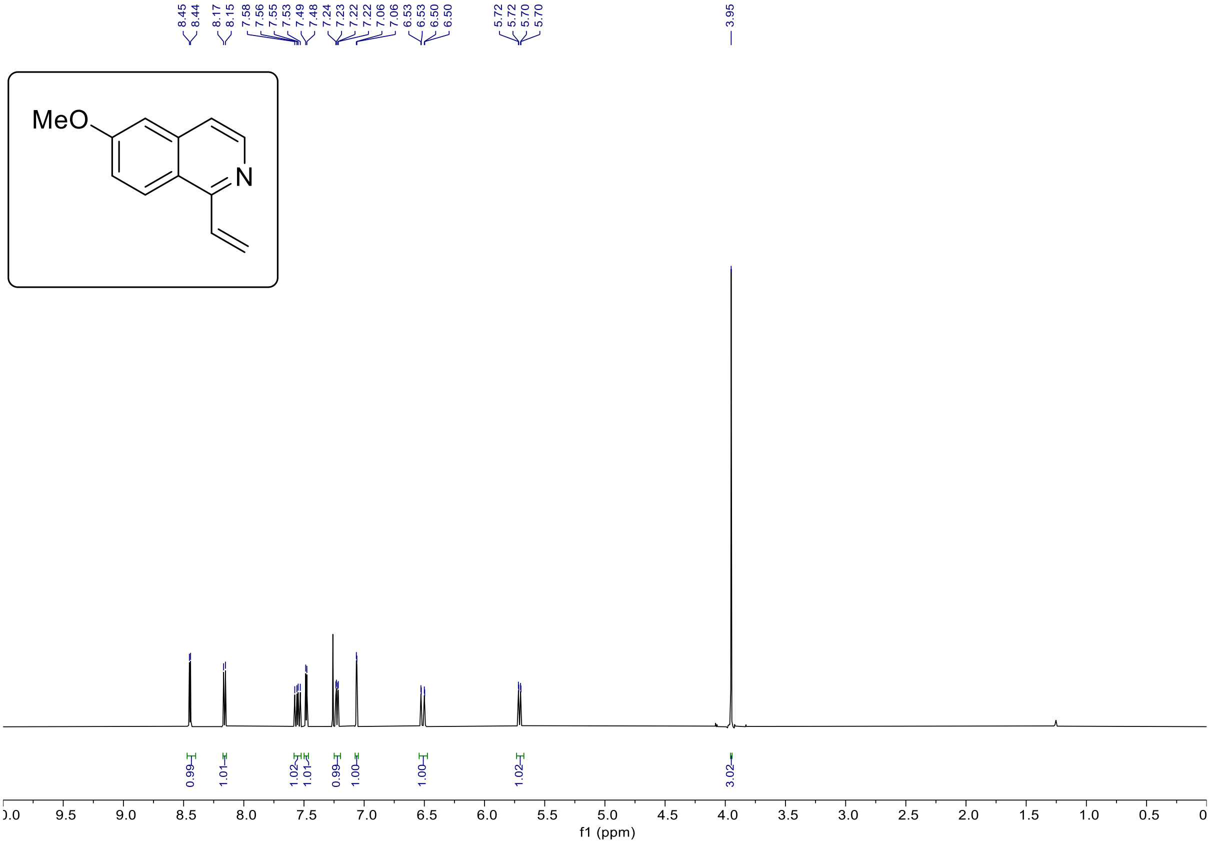

101 MHz, ^{13}C NMR in Chloroform-*d*

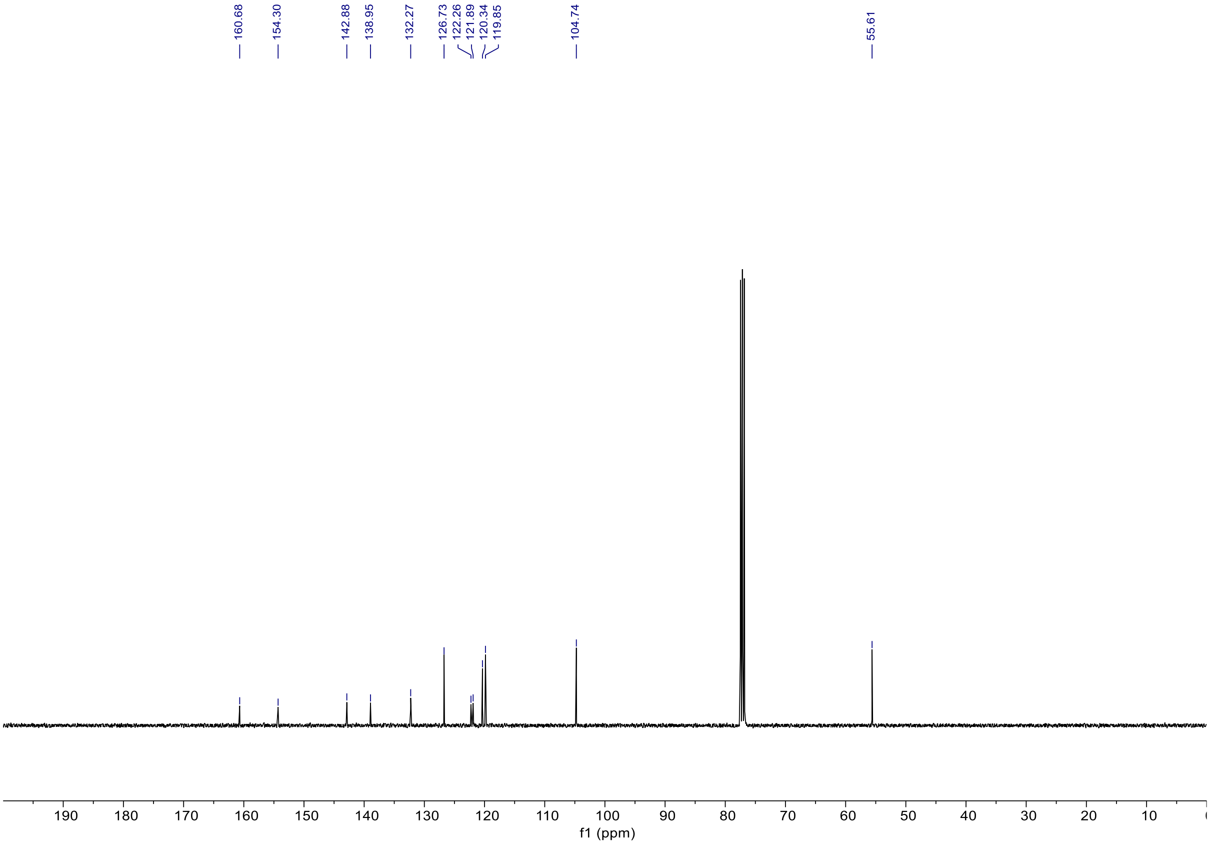

4-bromo-1-vinylisoquinoline (4h).

400 MHz, ^{1}H NMR in Chloroform-*d*

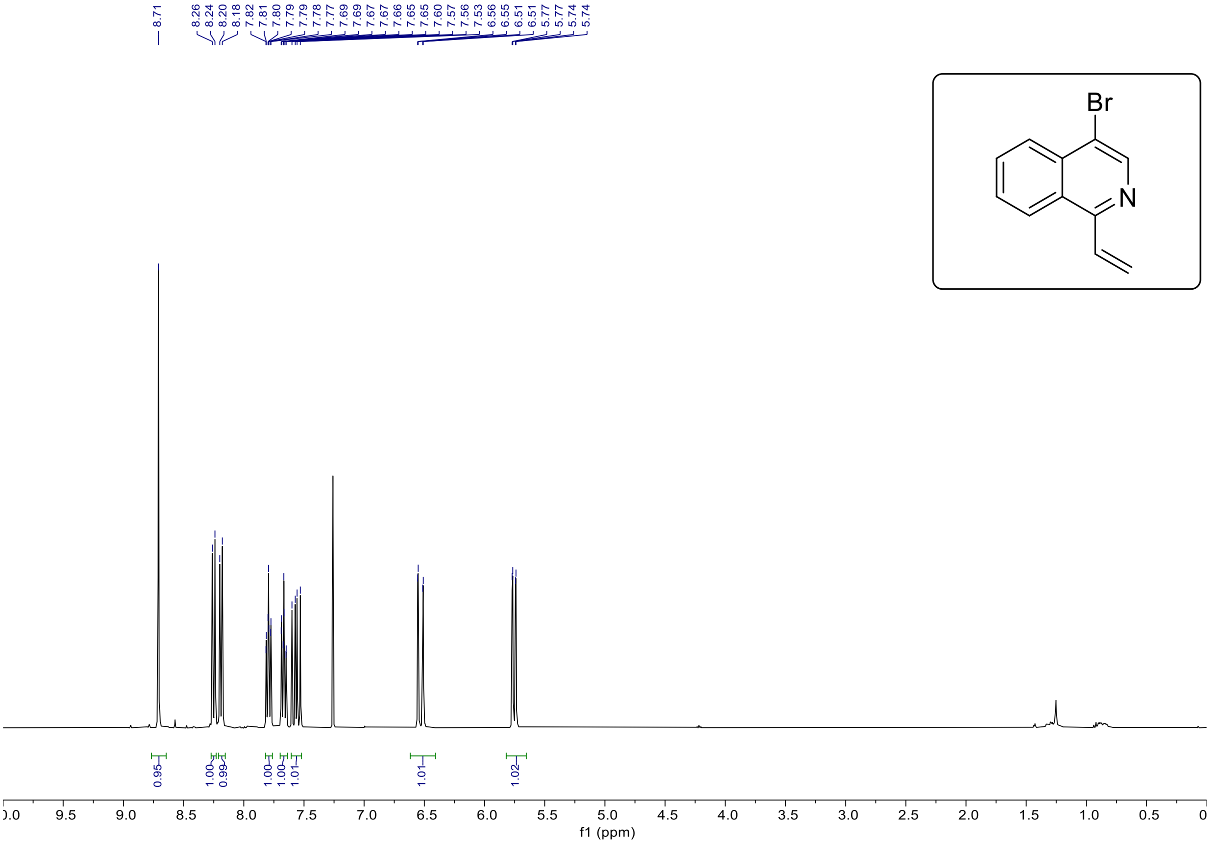

101 MHz, ^{13}C NMR in Chloroform-*d*

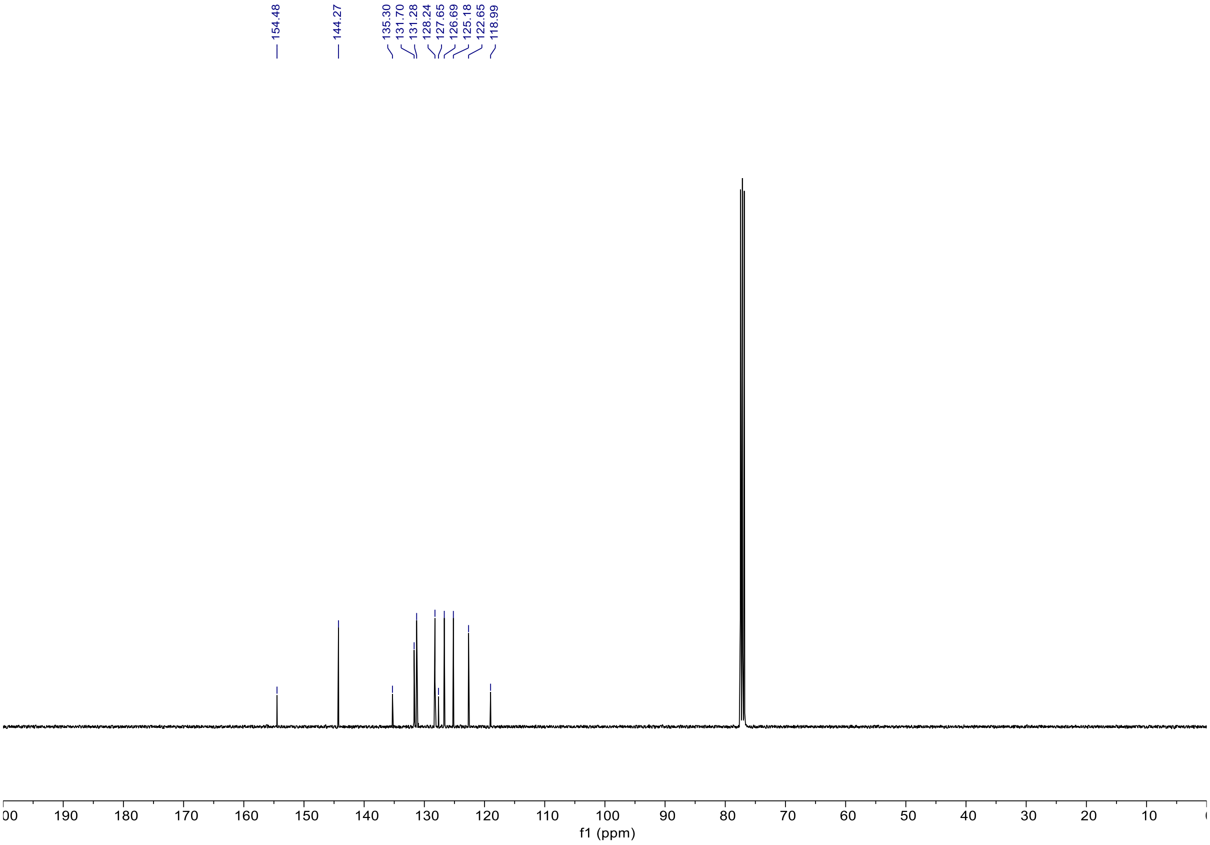

5-chloro-1-vinylisoquinoline (4i).

400 MHz, ^{1}H NMR in Chloroform-*d*

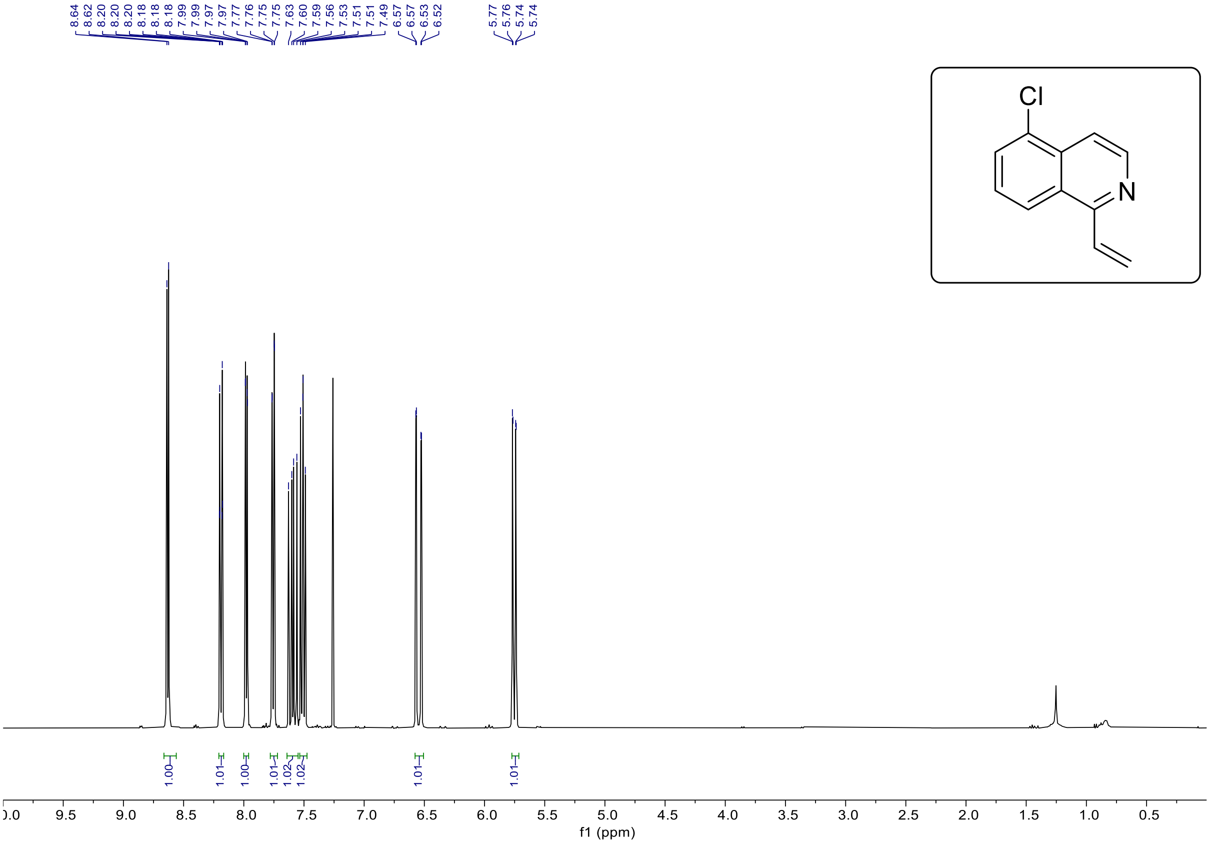

101 MHz, ^{13}C NMR in Chloroform-*d*

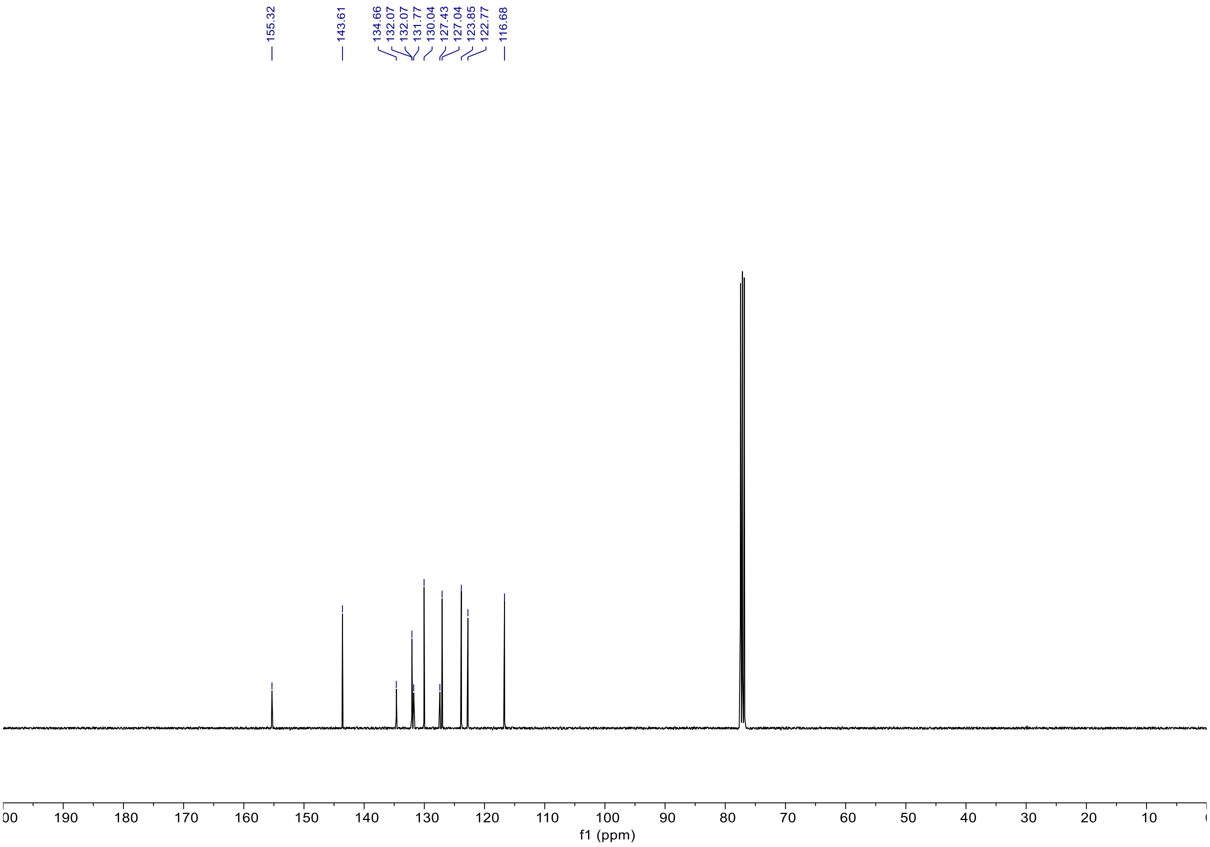

5-bromo-1-vinylisoquinoline (4j).

600 MHz, ^{1}H NMR in Chloroform-*d*

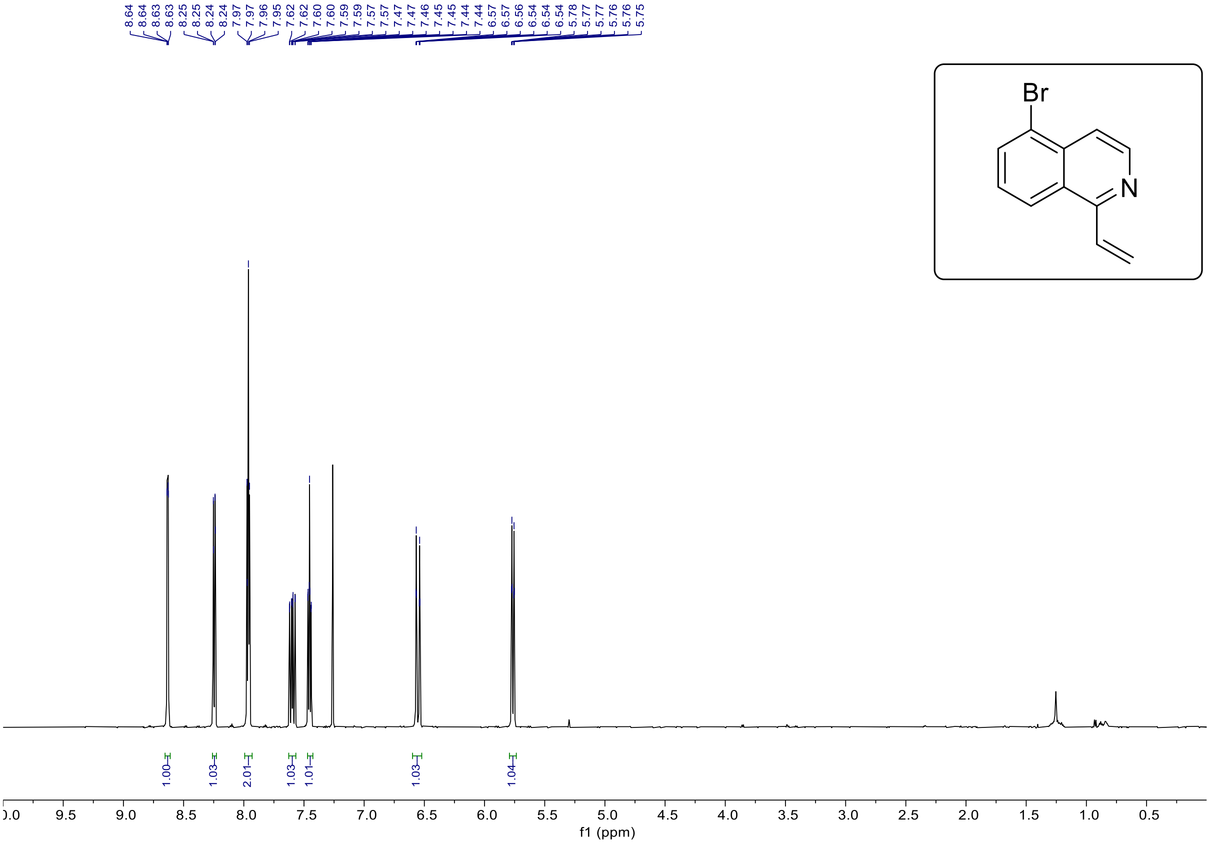

101 MHz, ^{13}C NMR in Chloroform-*d*

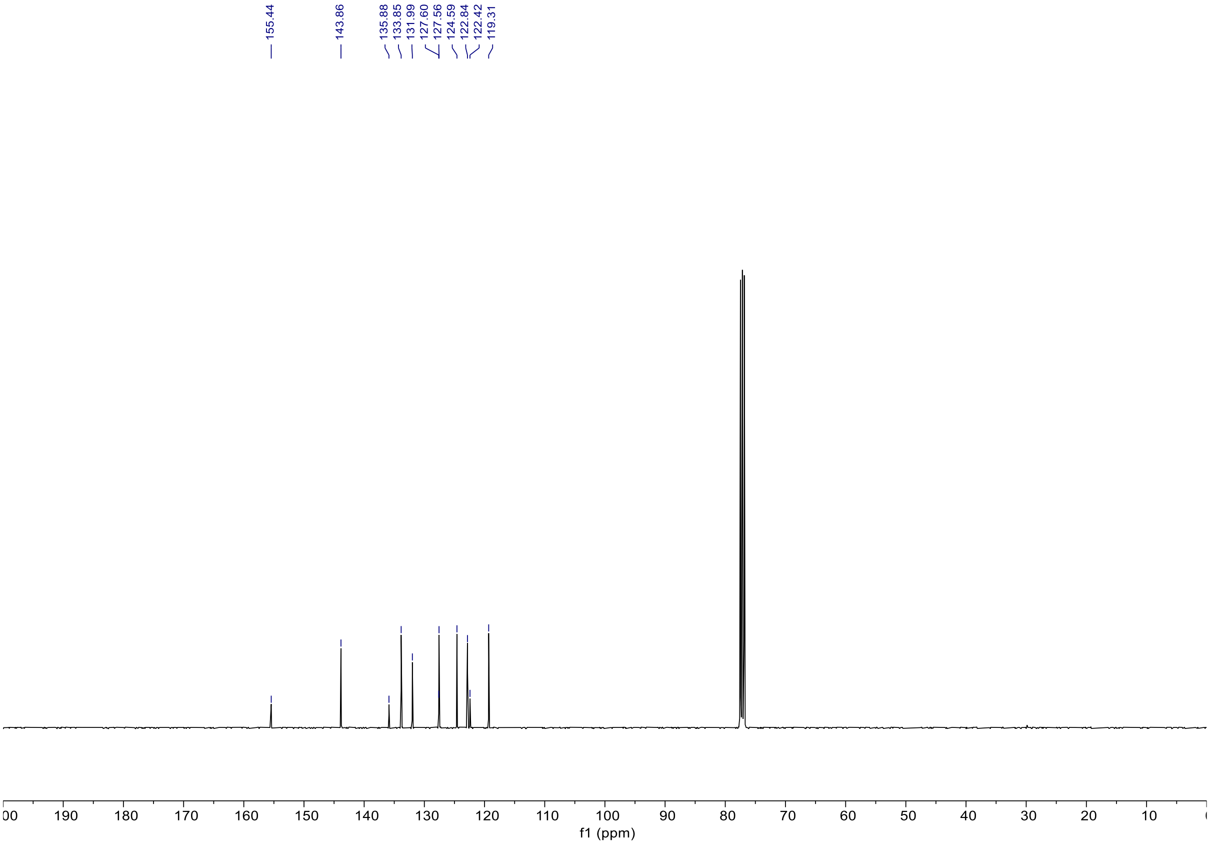

6-bromo-1-vinylisoquinoline (4k).

400 MHz, ^{1}H NMR in Chloroform-*d*

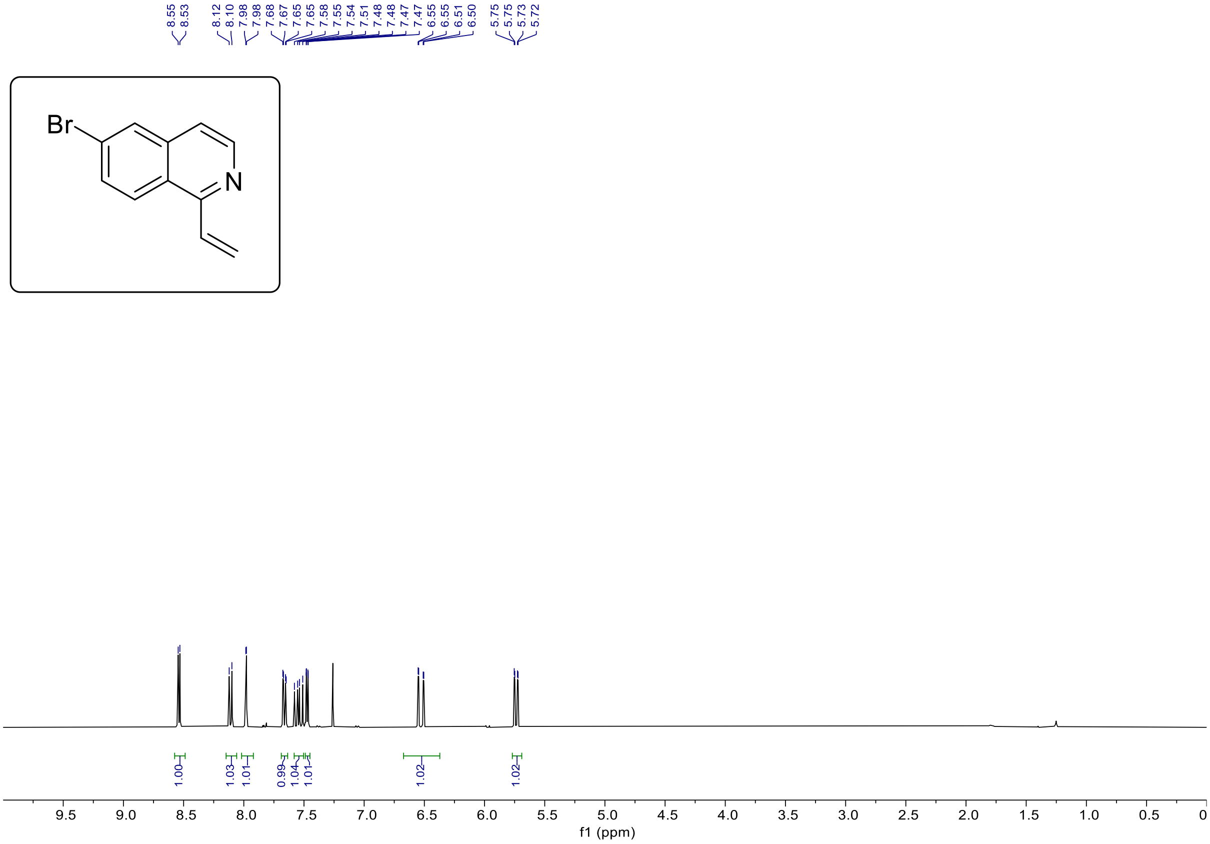

101 MHz, ^{13}C NMR in Chloroform-*d*

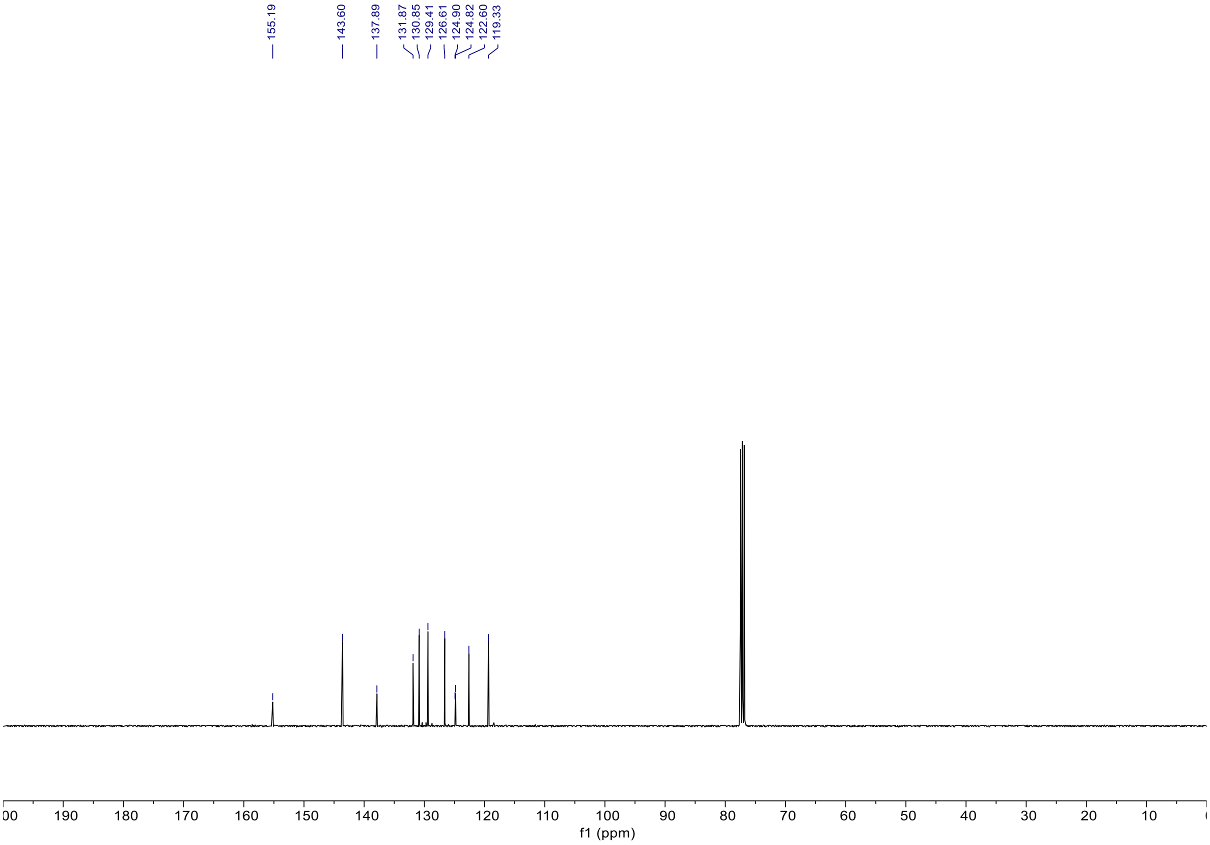

7-bromo-1-vinylisoquinoline (4l).

400 MHz, ^{1}H NMR in Chloroform-*d*

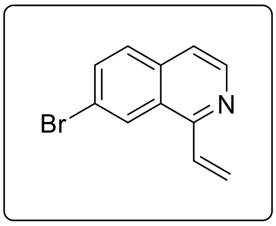

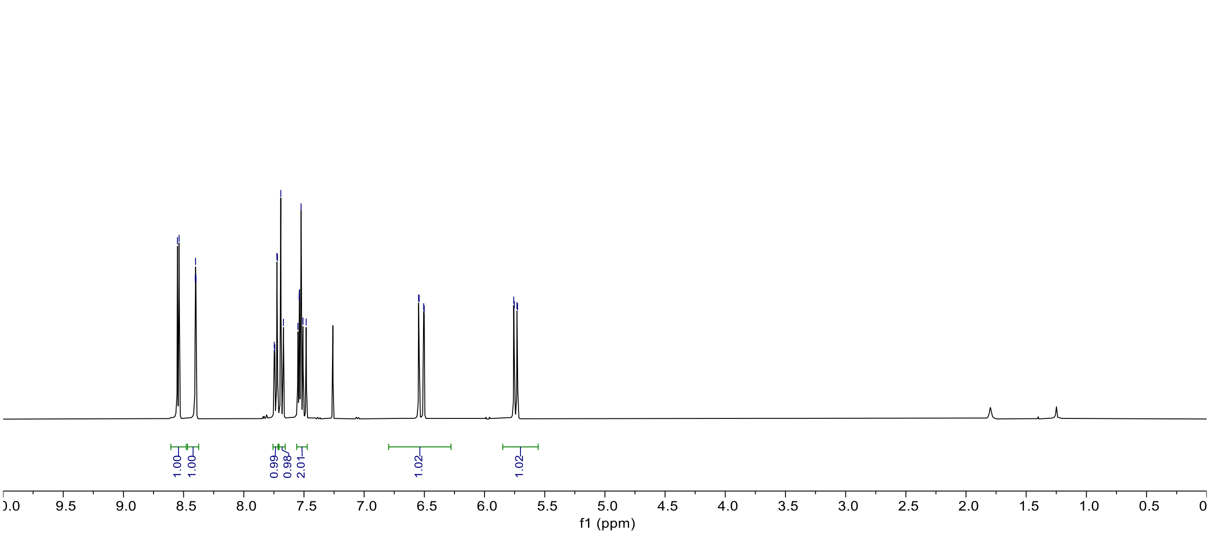

101 MHz, ^{13}C NMR in Chloroform-*d*

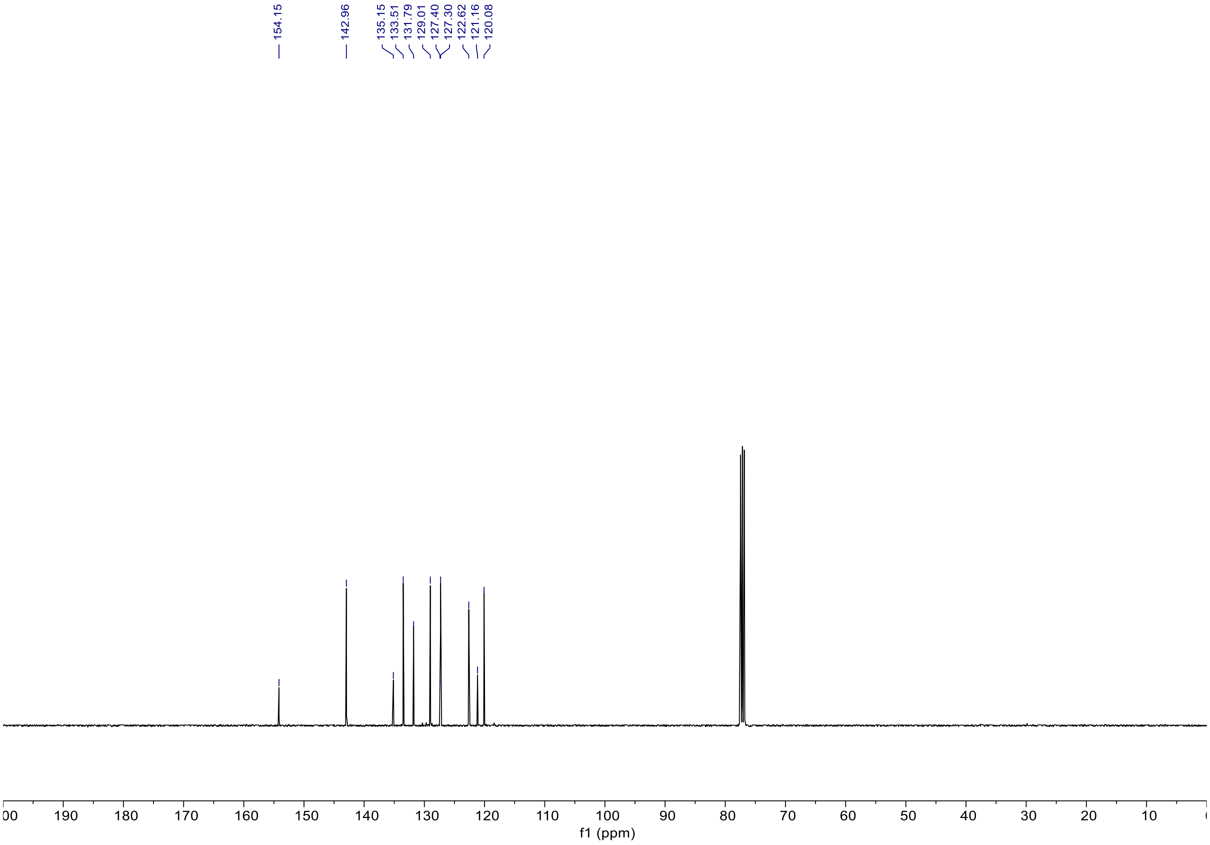

4-methyl-2-vinylquinoline (4m).

400 MHz, ^{1}H NMR in Chloroform-*d*

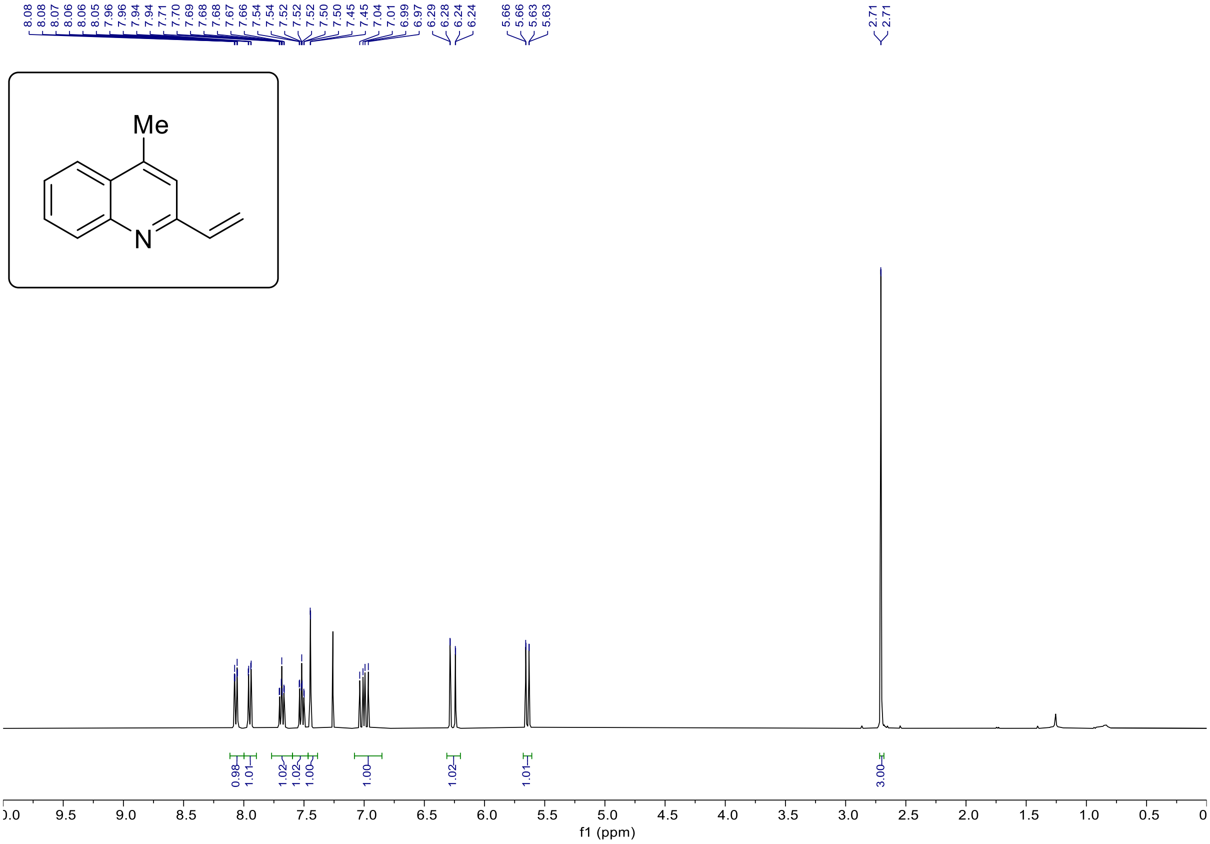

101 MHz, ^{13}C NMR in Chloroform-*d*

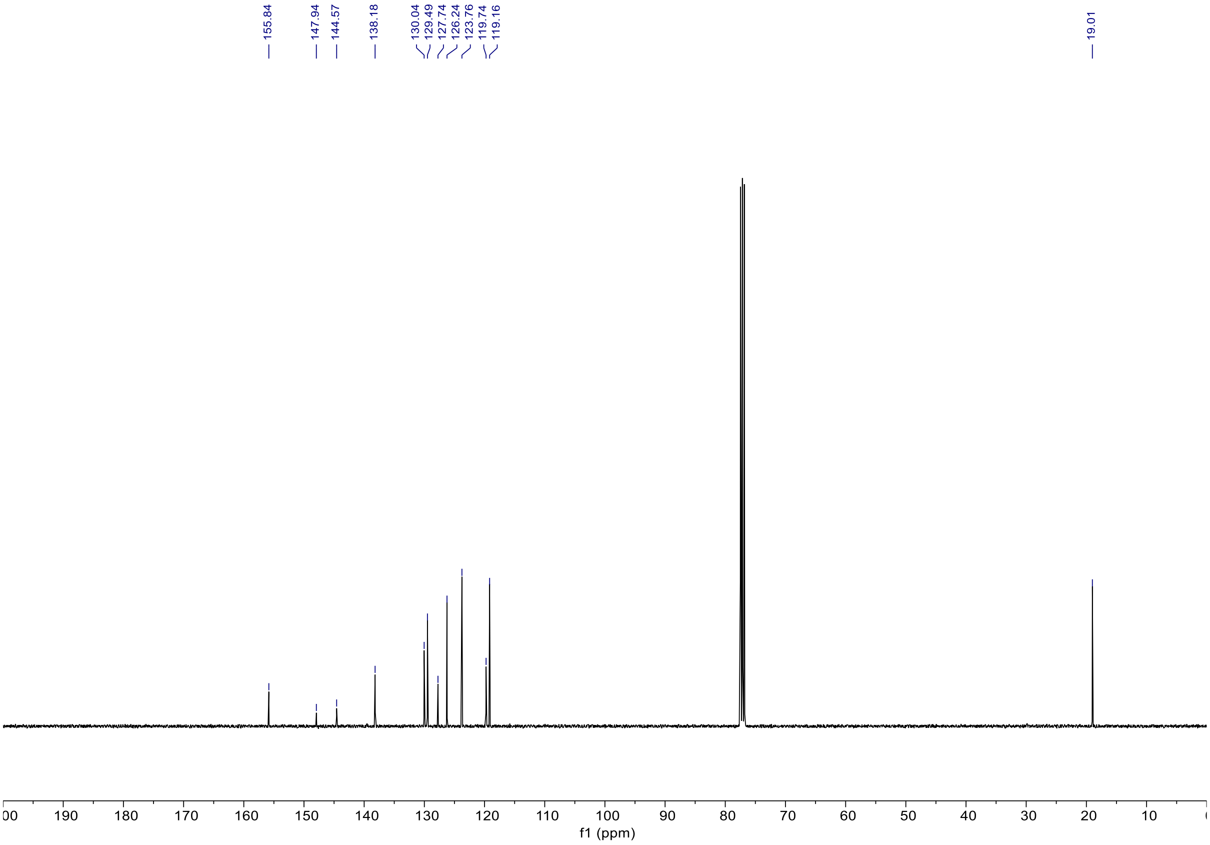

2-methyl-4-vinylquinoline (4n).

400 MHz, ^{1}H NMR in Chloroform-*d*

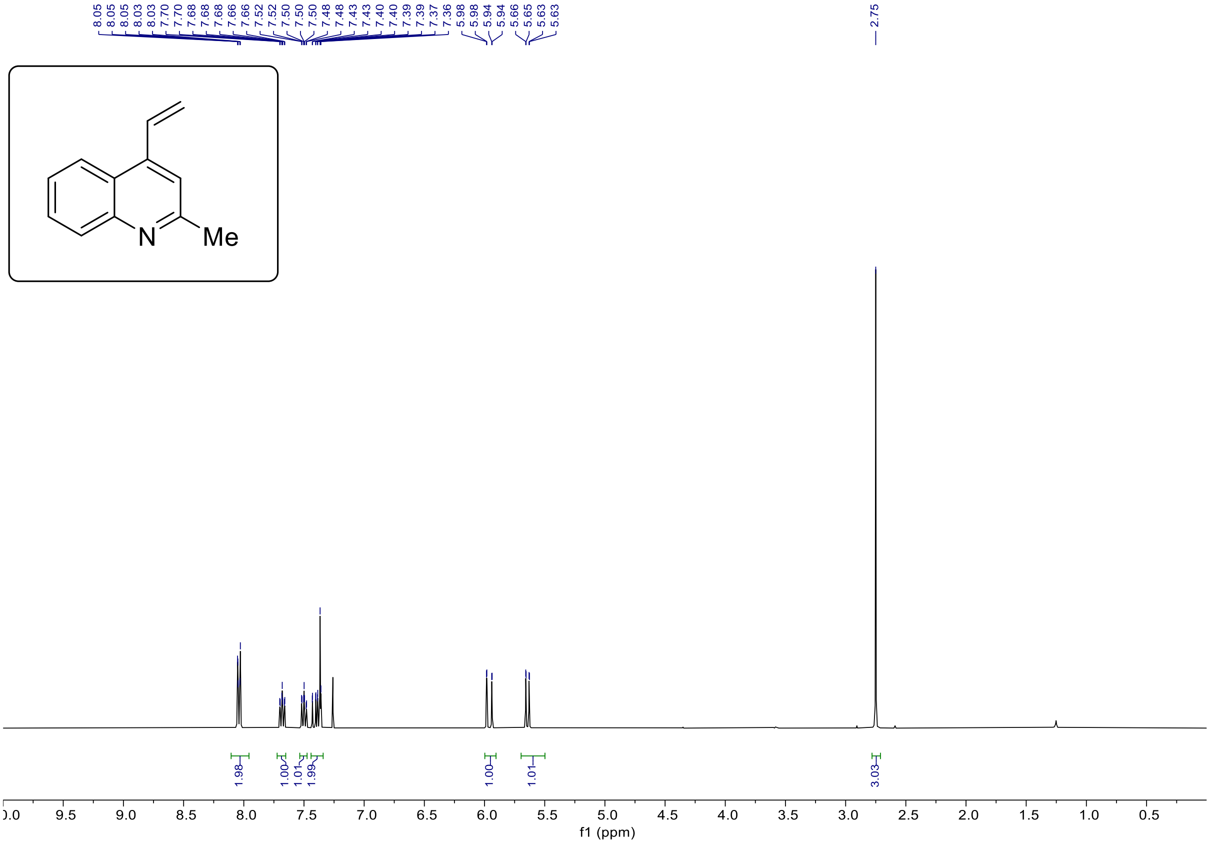

101 MHz, ^{13}C NMR in Chloroform-*d*

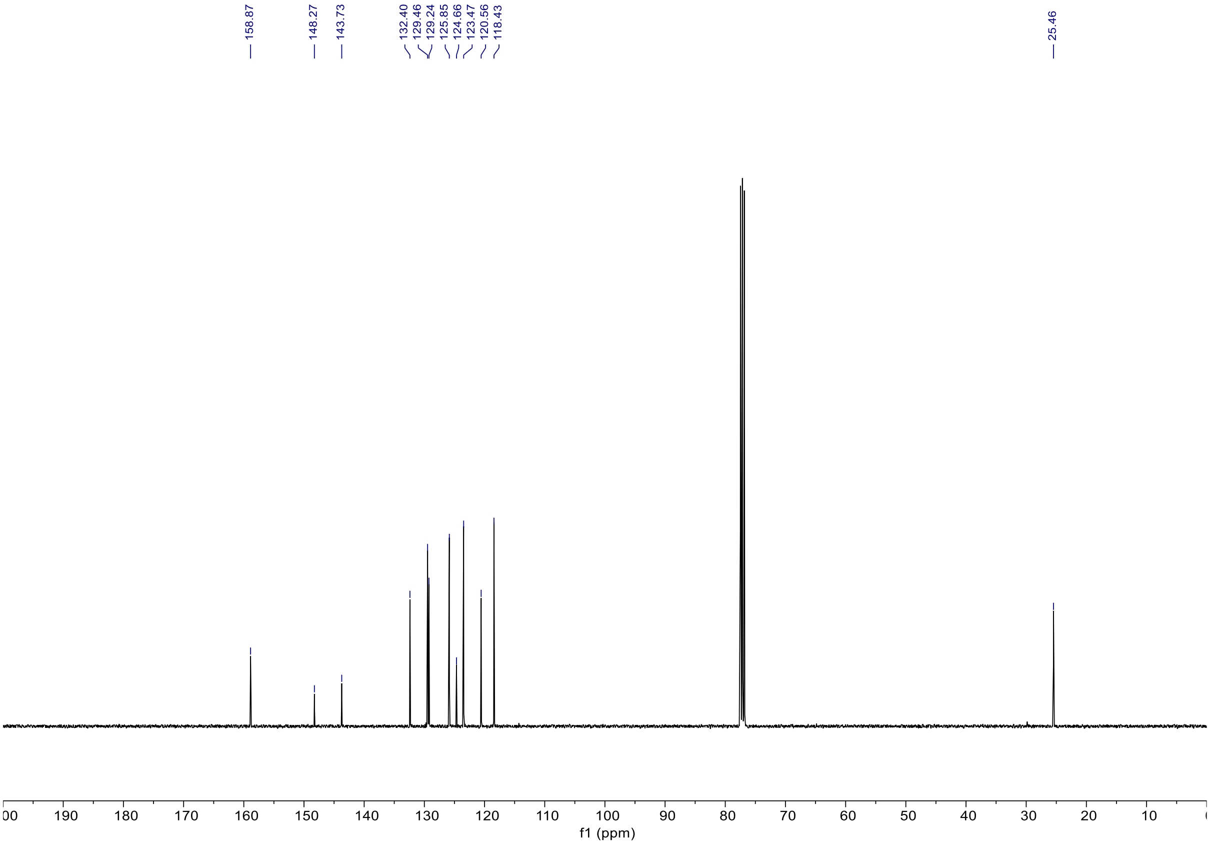

2-phenyl-4-vinylquinoline (4o).

400 MHz, ^{1}H NMR in Chloroform-*d*

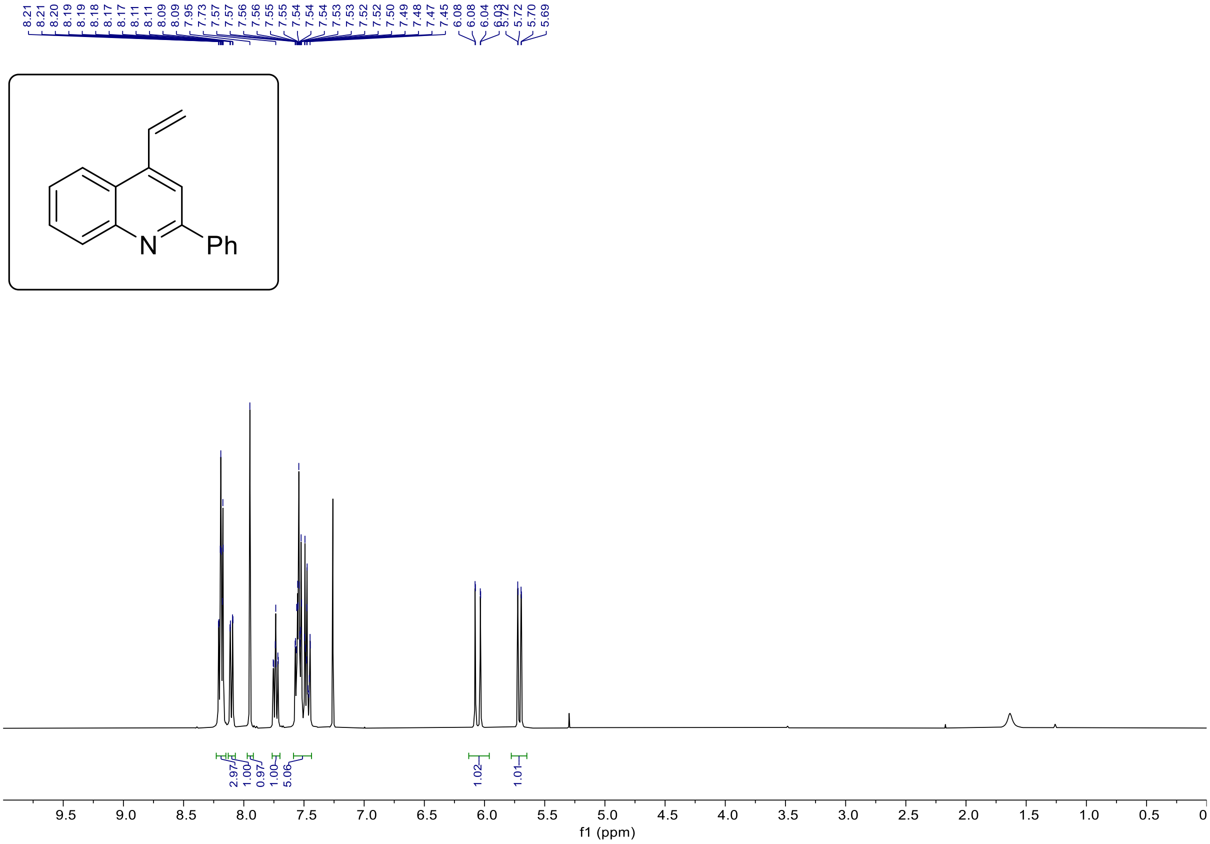

101 MHz, ^{13}C NMR in Chloroform-*d*

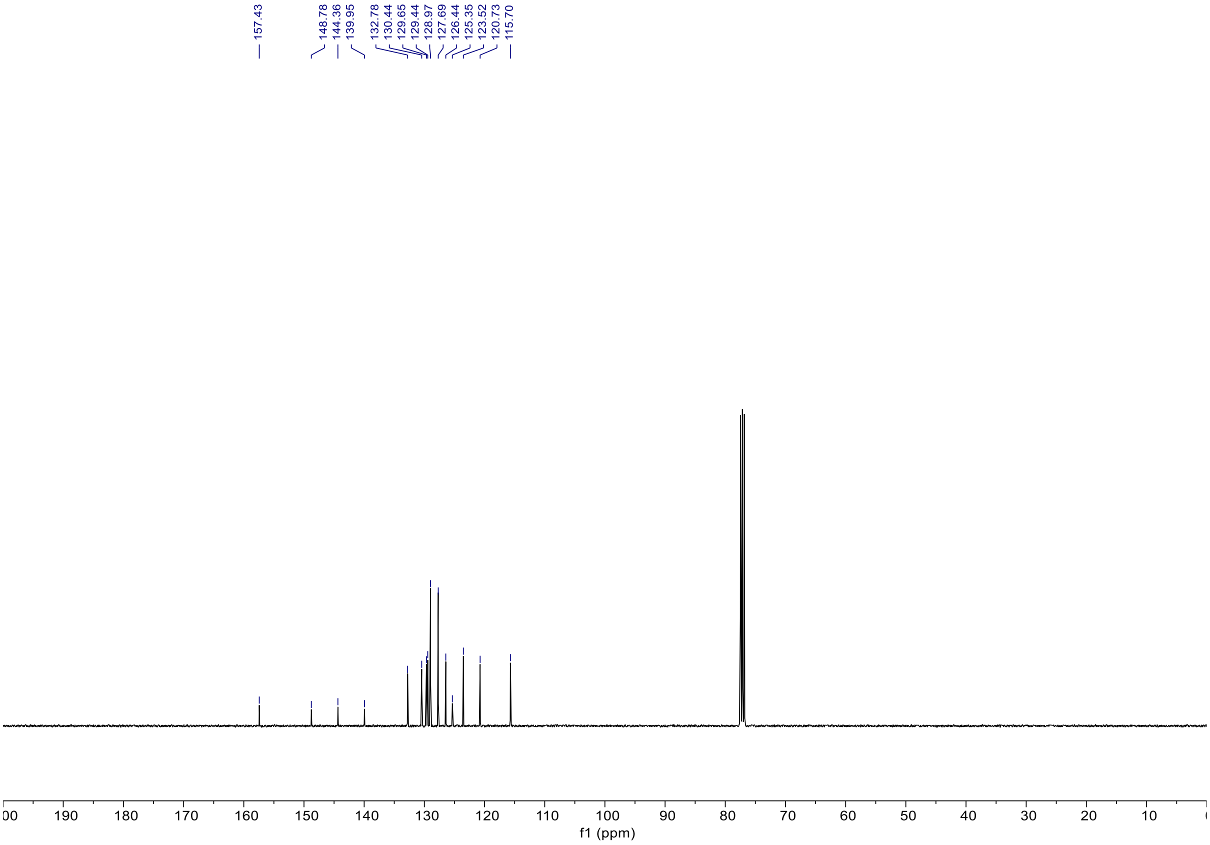

7-fluoro-2-methyl-4-vinylquinoline (4p).

600 MHz, ^{1}H NMR in Chloroform-*d*

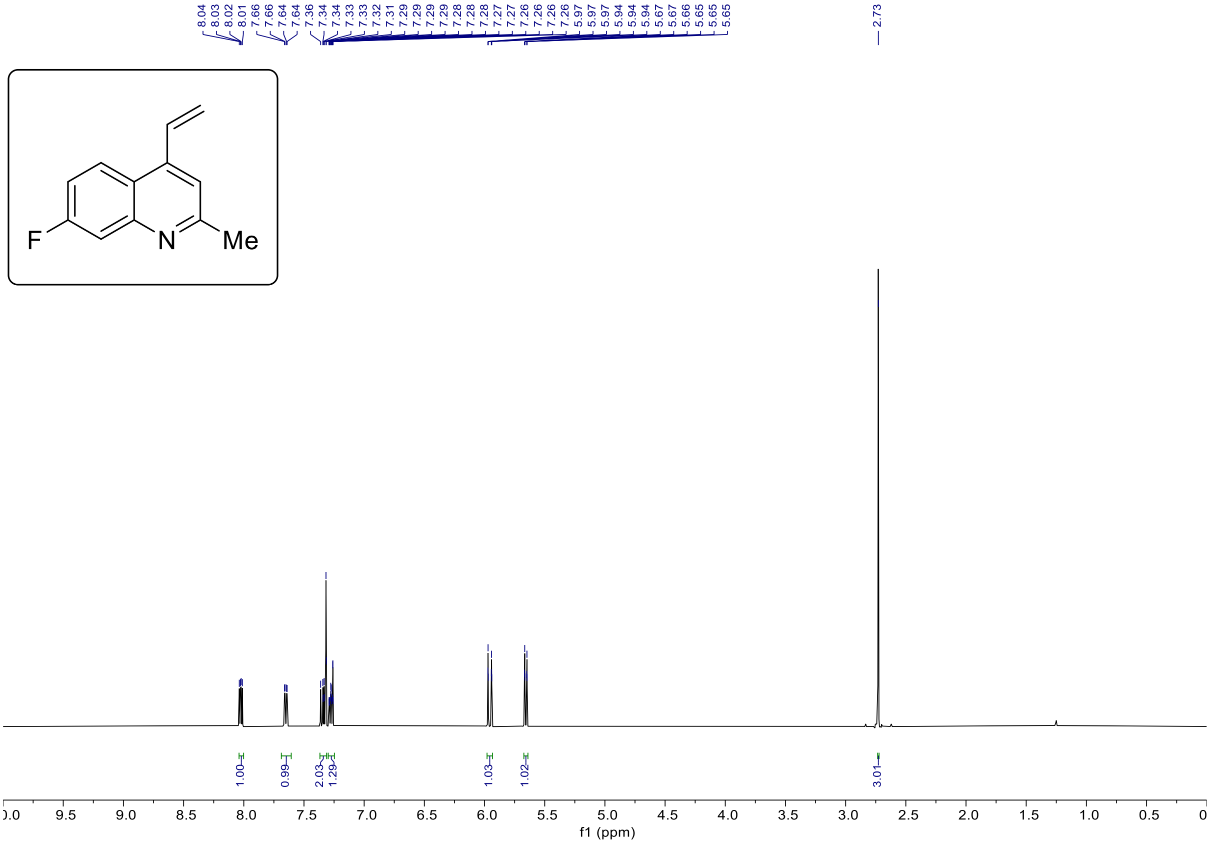

101 MHz, ^{13}C NMR in Chloroform-*d*

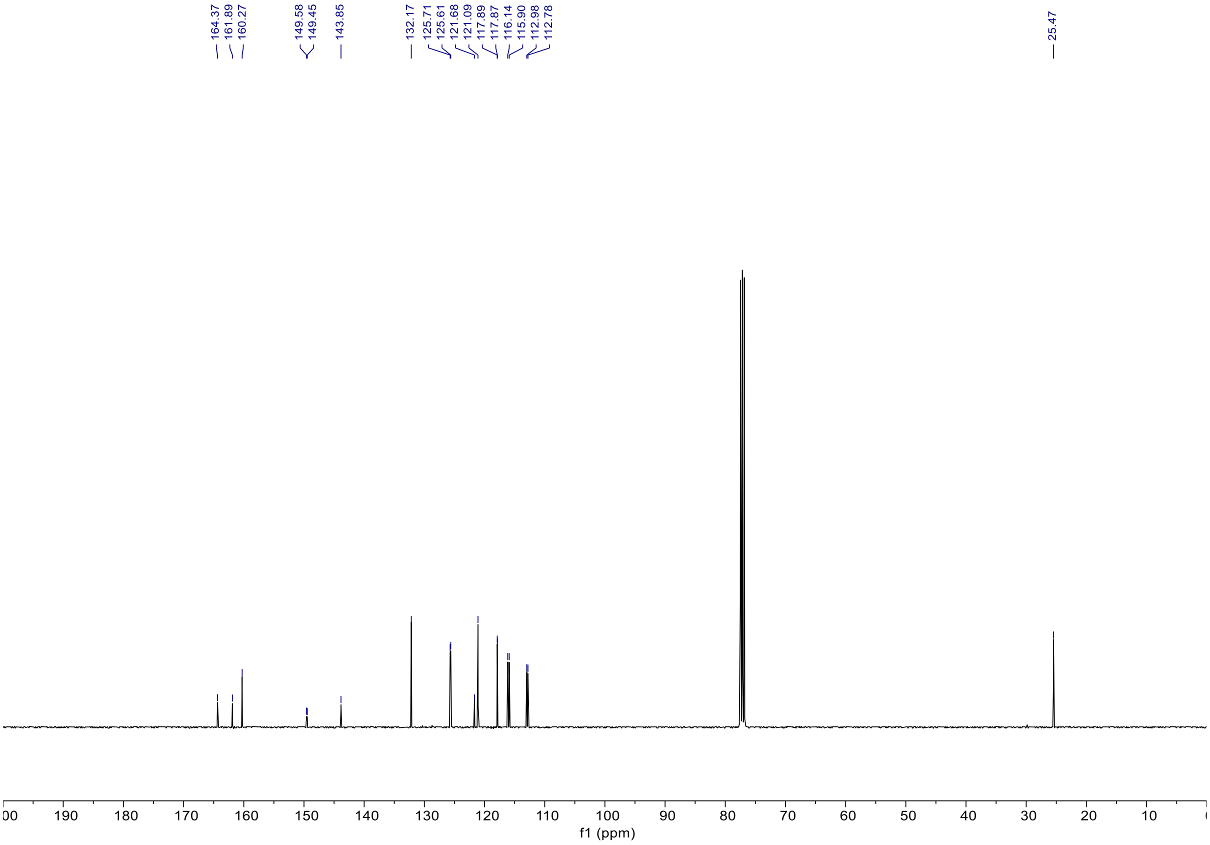

376 MHz, ^{19}F NMR in Chloroform-*d*

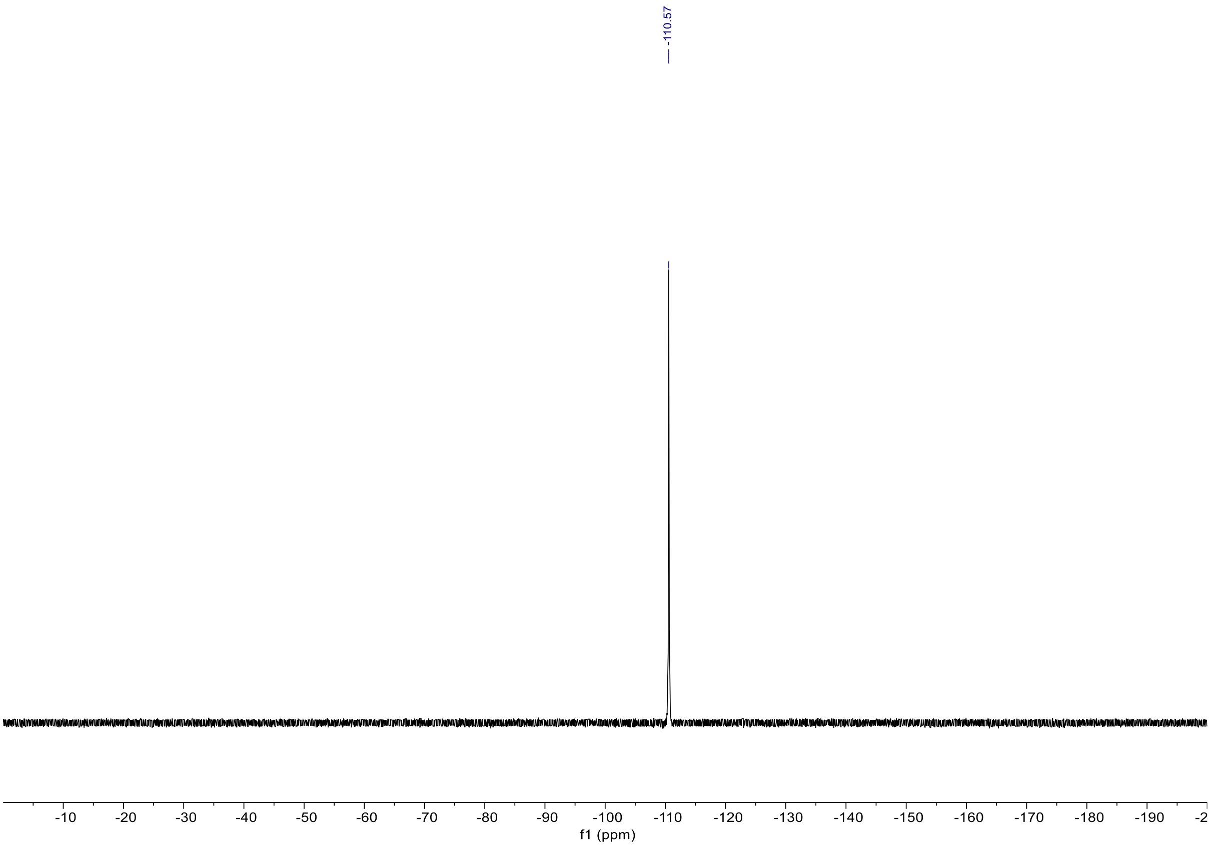

8-methoxy-2-methyl-4-vinylquinoline (4q).

400 MHz, ^{1}H NMR in Chloroform-*d*

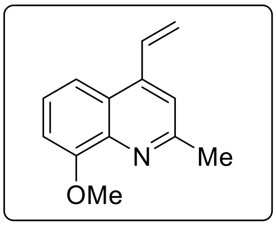

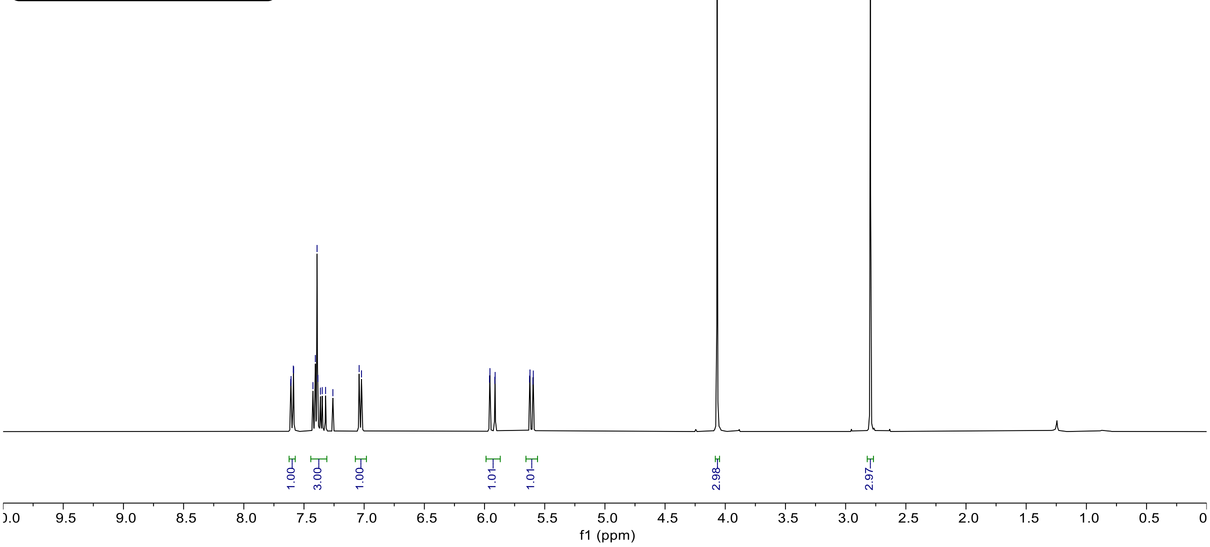

101 MHz, ^{13}C NMR in Chloroform-*d*

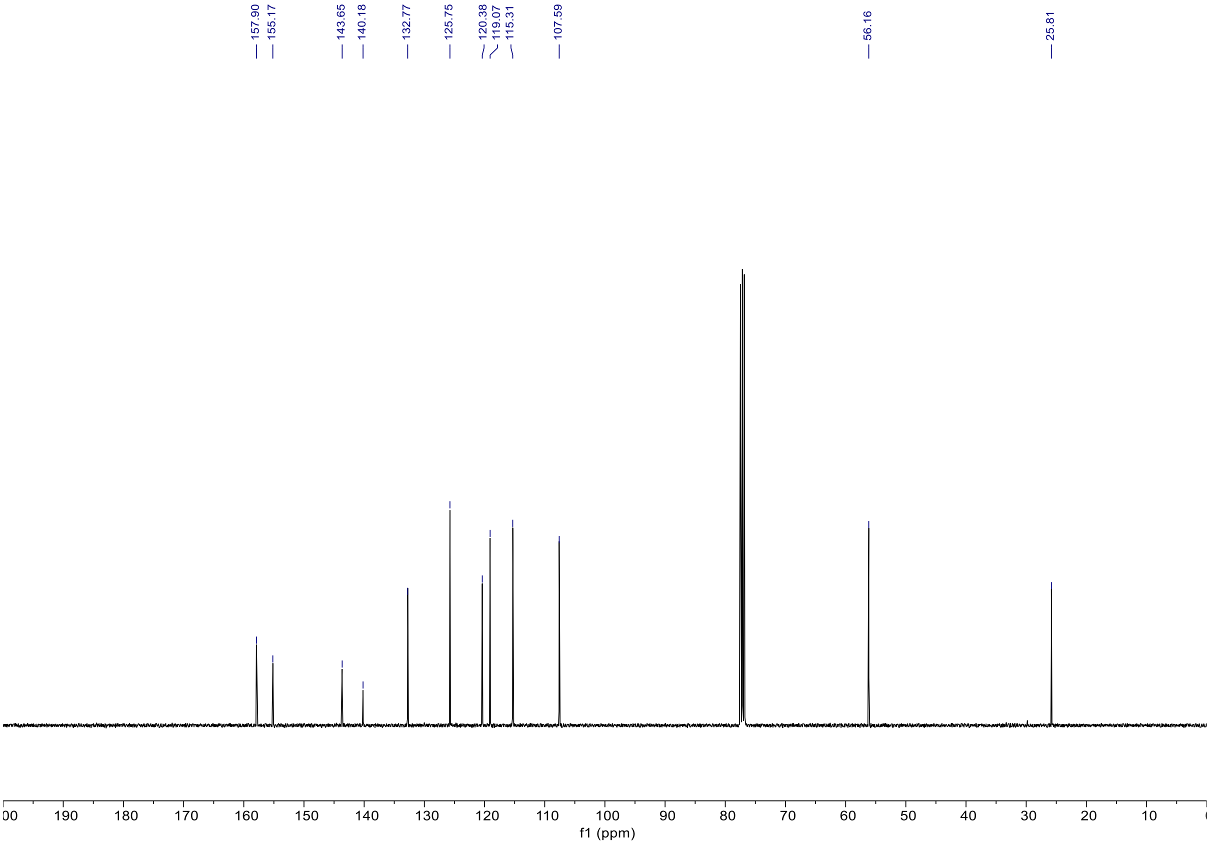

9-vinylacridine (4r).

400 MHz, ^{1}H NMR in Chloroform-*d*

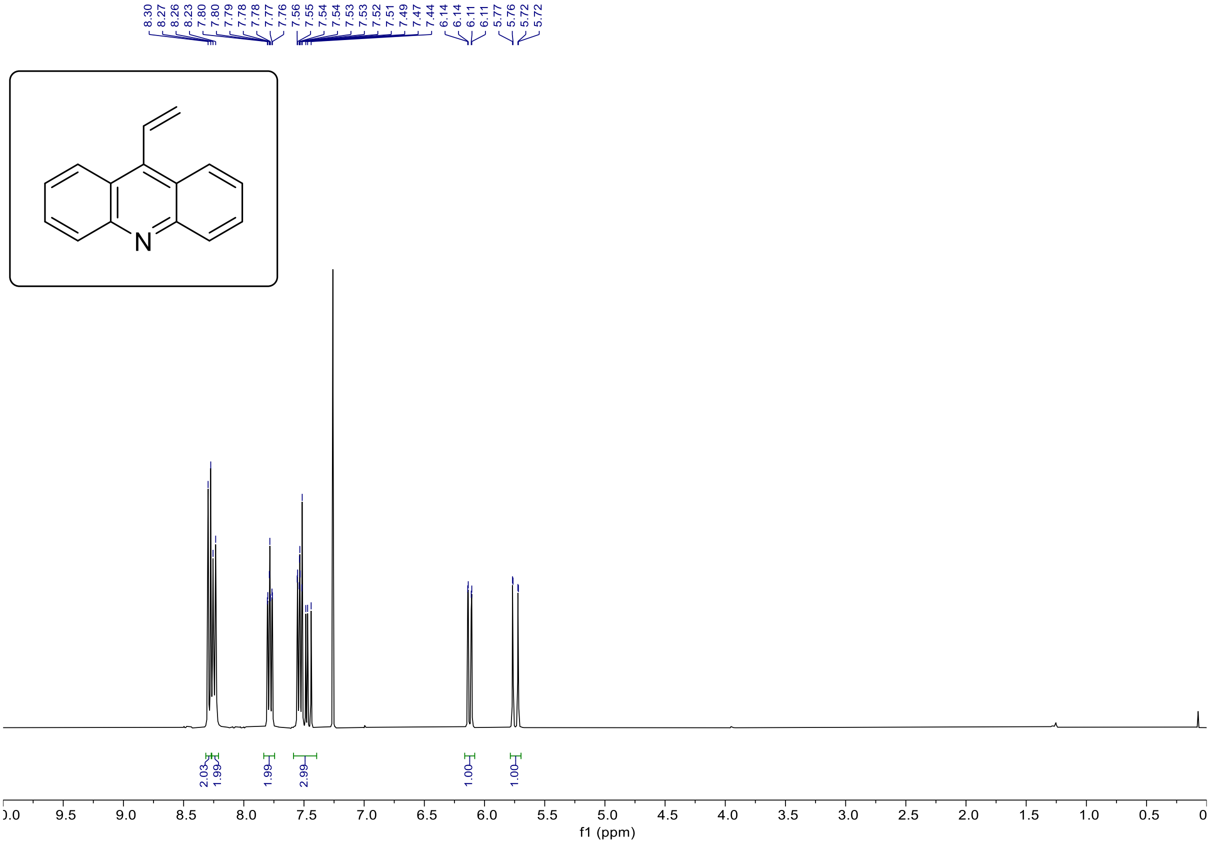

126 MHz, ^{13}C NMR in Chloroform-*d*

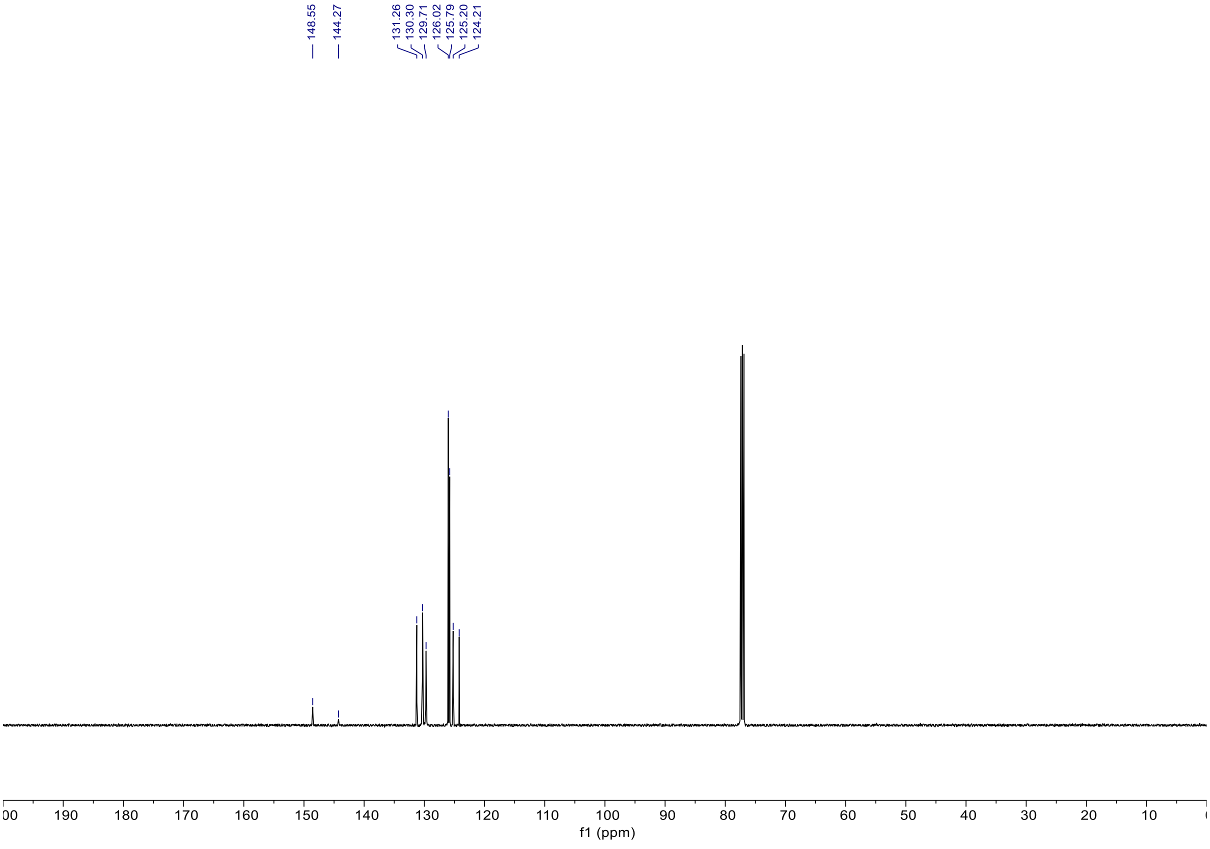

6-vinylphenanthridine (4s).

400 MHz, ^{1}H NMR in Chloroform-*d*

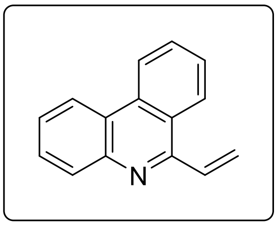

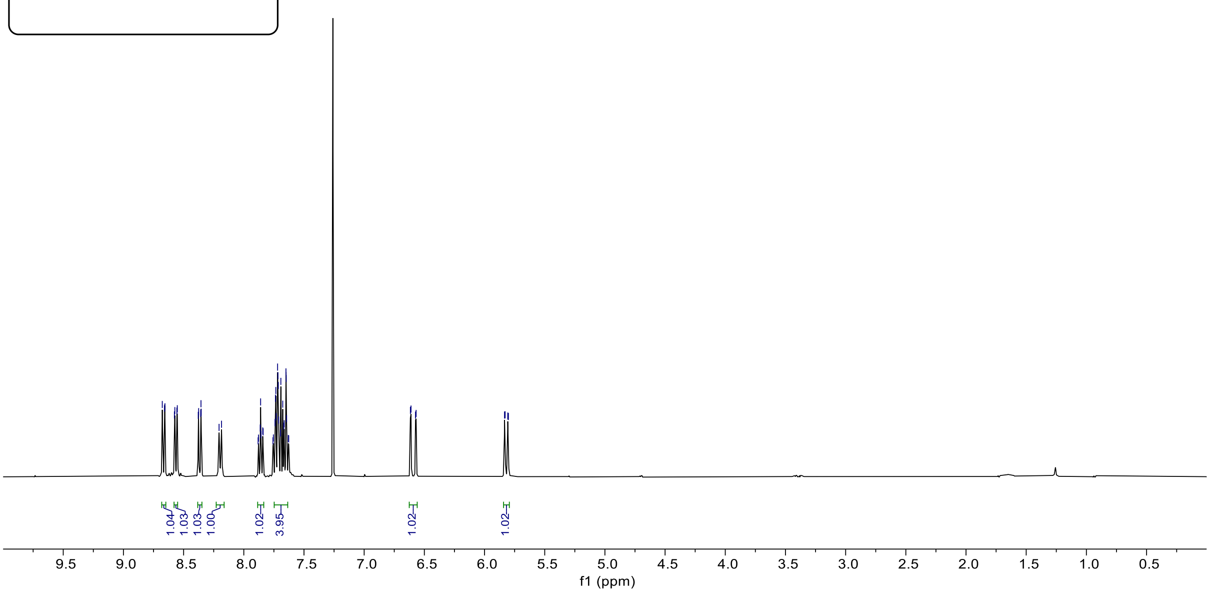

101 MHz, ^{13}C NMR in Chloroform-*d*

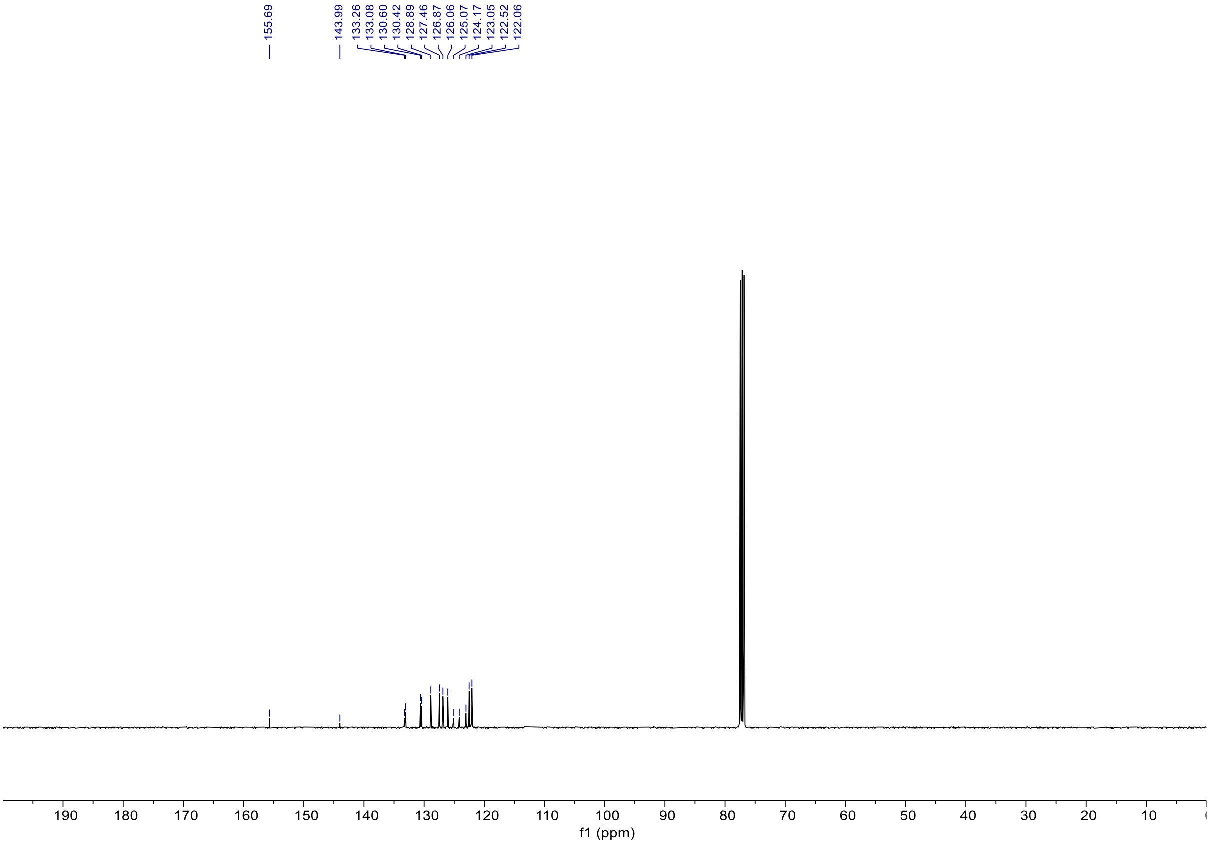

1-methoxy-4-vinylphthalazine (4t).

400 MHz, ^{1}H NMR in Chloroform-*d*

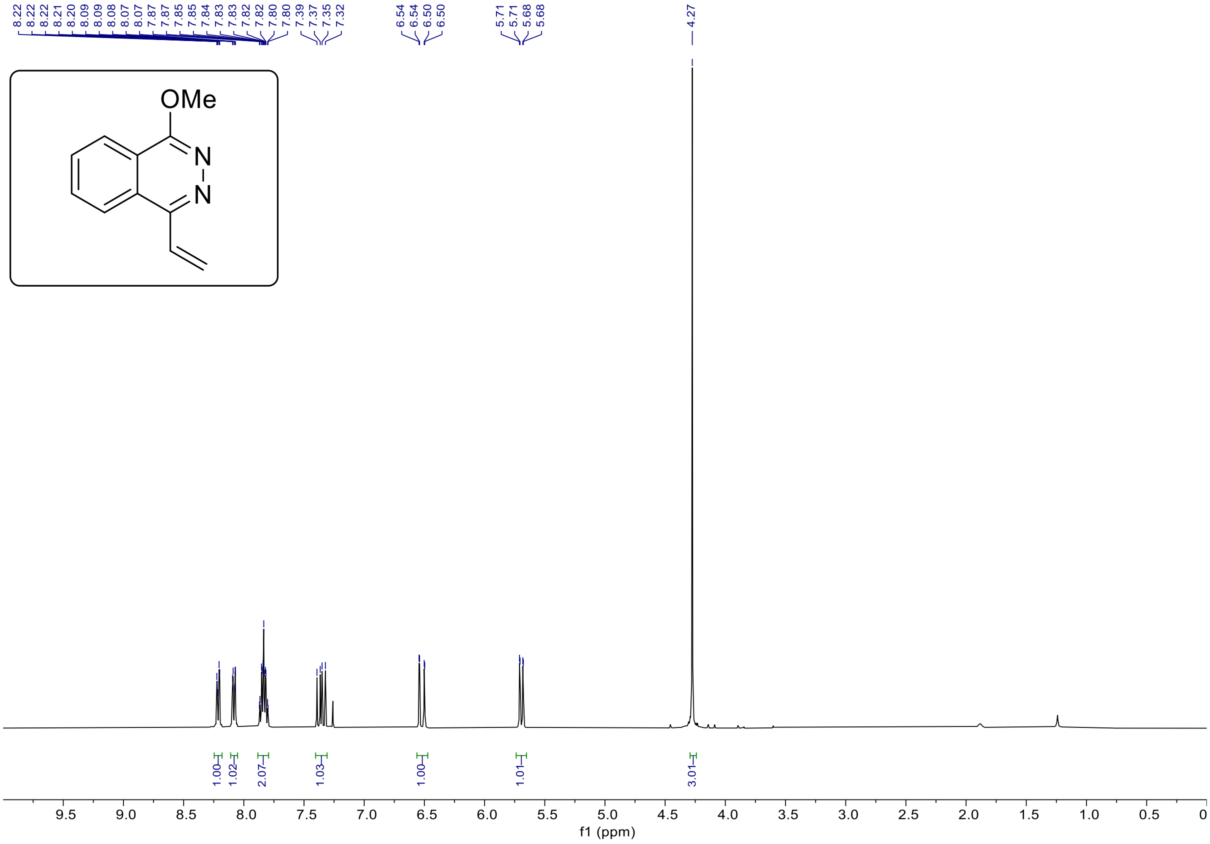

101 MHz, ^{13}C NMR in Chloroform-*d*

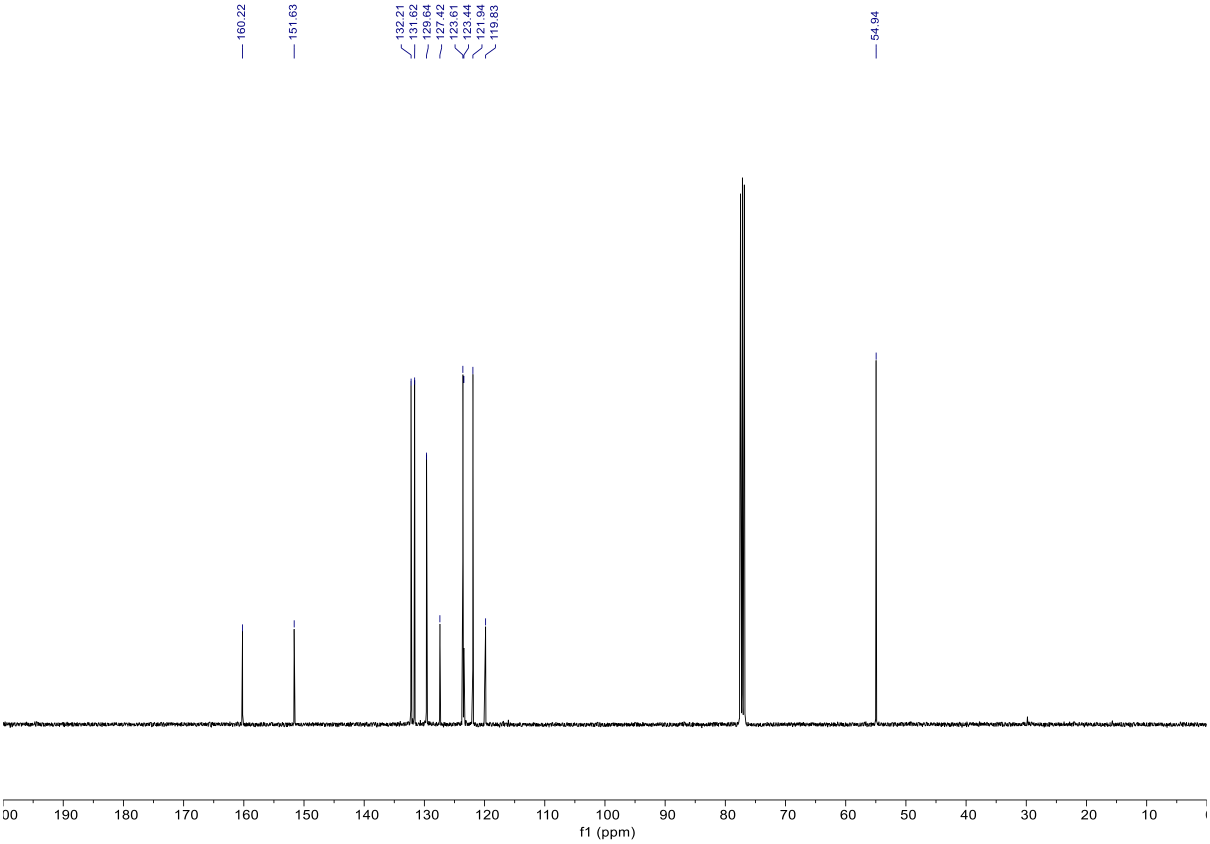

2-vinylbenzo[d]thiazole (4u).

400 MHz, ^{1}H NMR in Chloroform-*d*

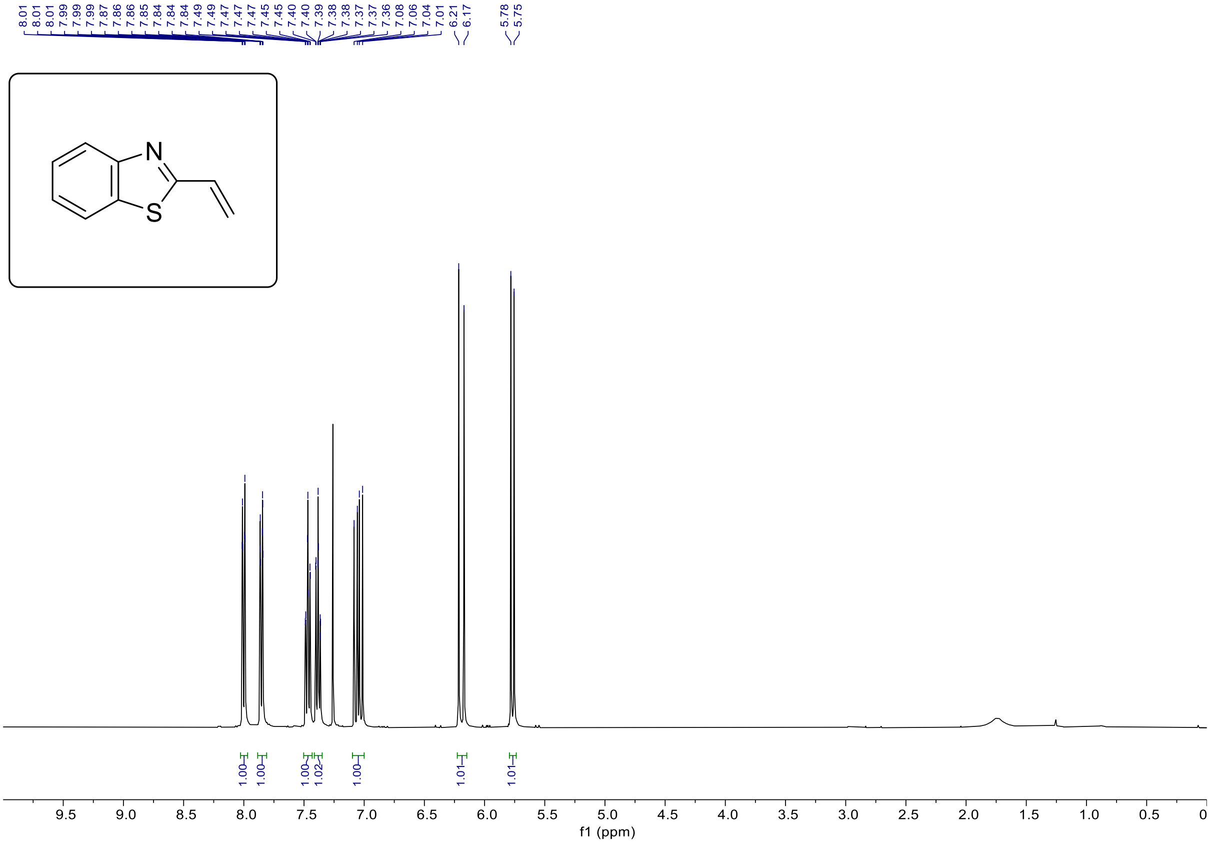

101 MHz, ^{13}C NMR in Chloroform-*d*

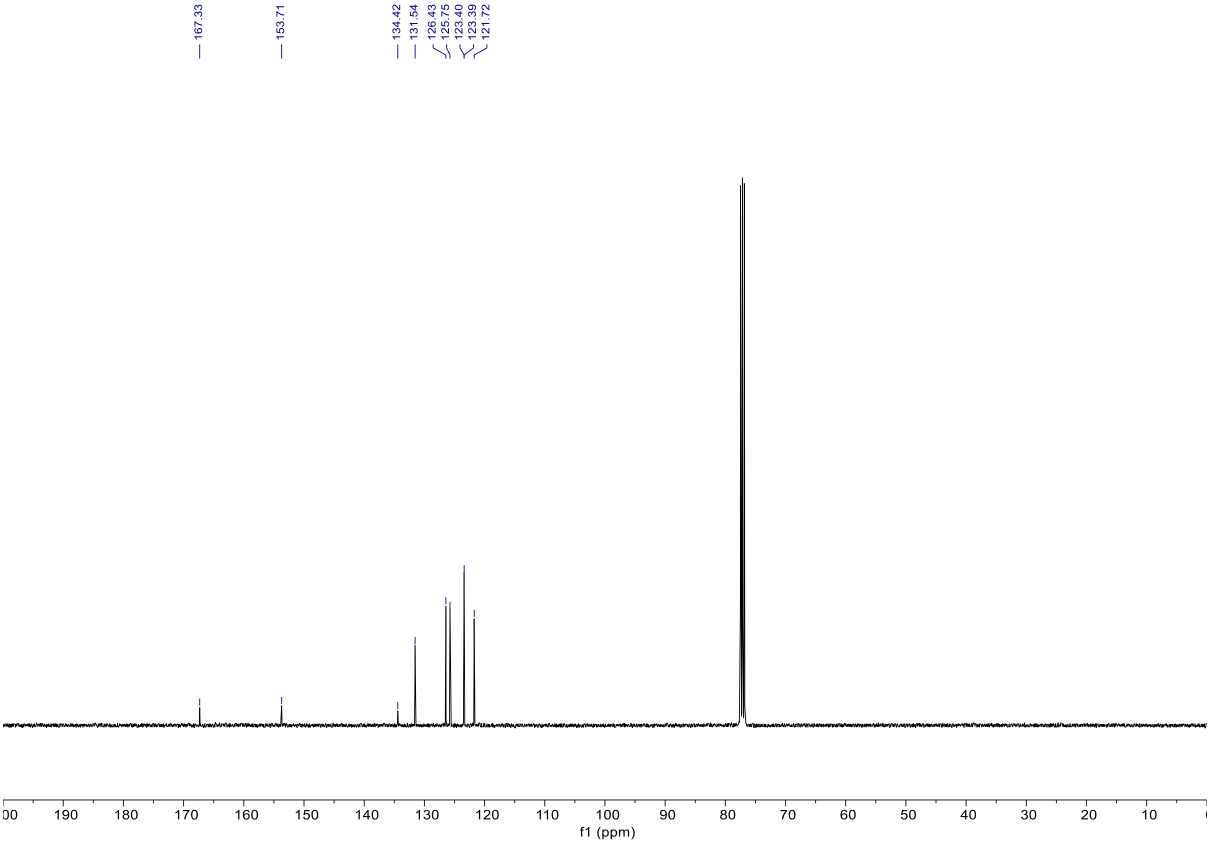

4-methyl-6-vinylnicotinonitrile (4v).

600 MHz, ^{1}H NMR in Chloroform-*d*

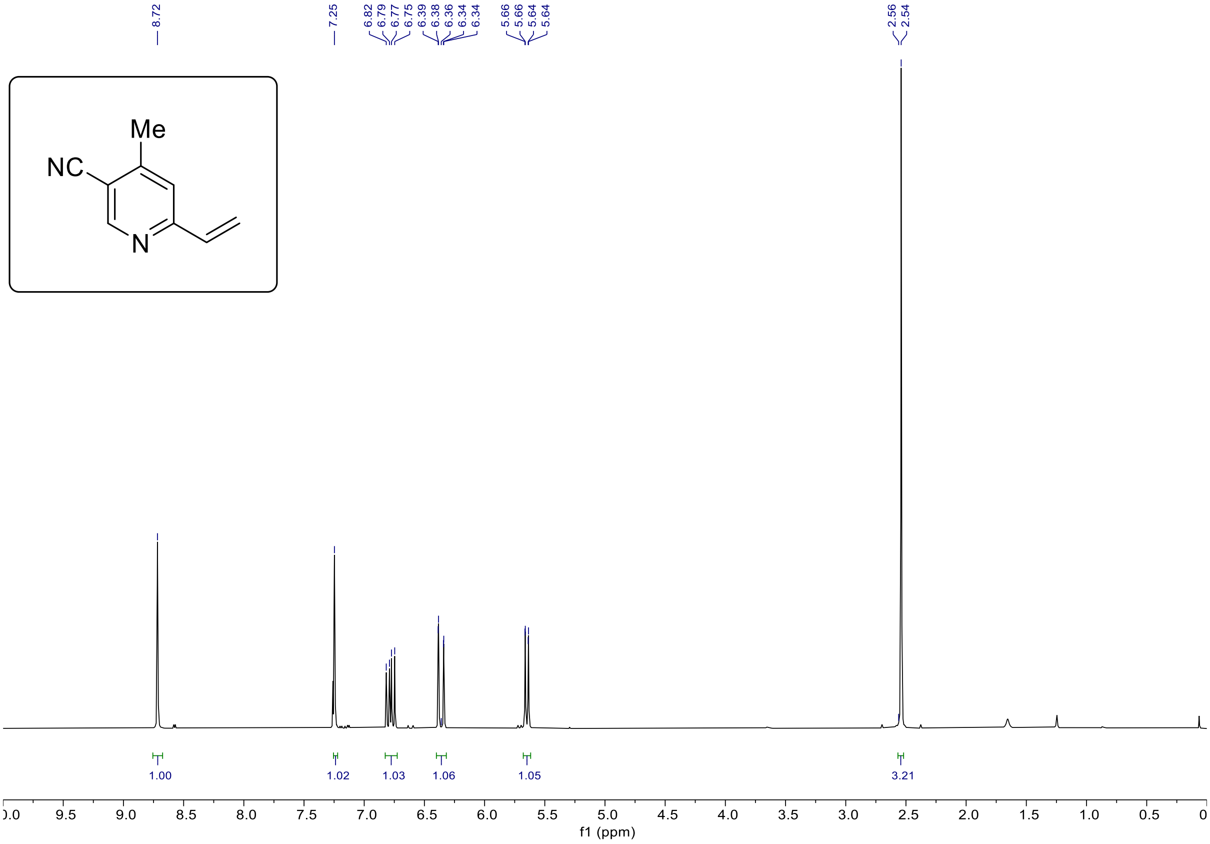

101 MHz, ^{13}C NMR in Chloroform-*d*

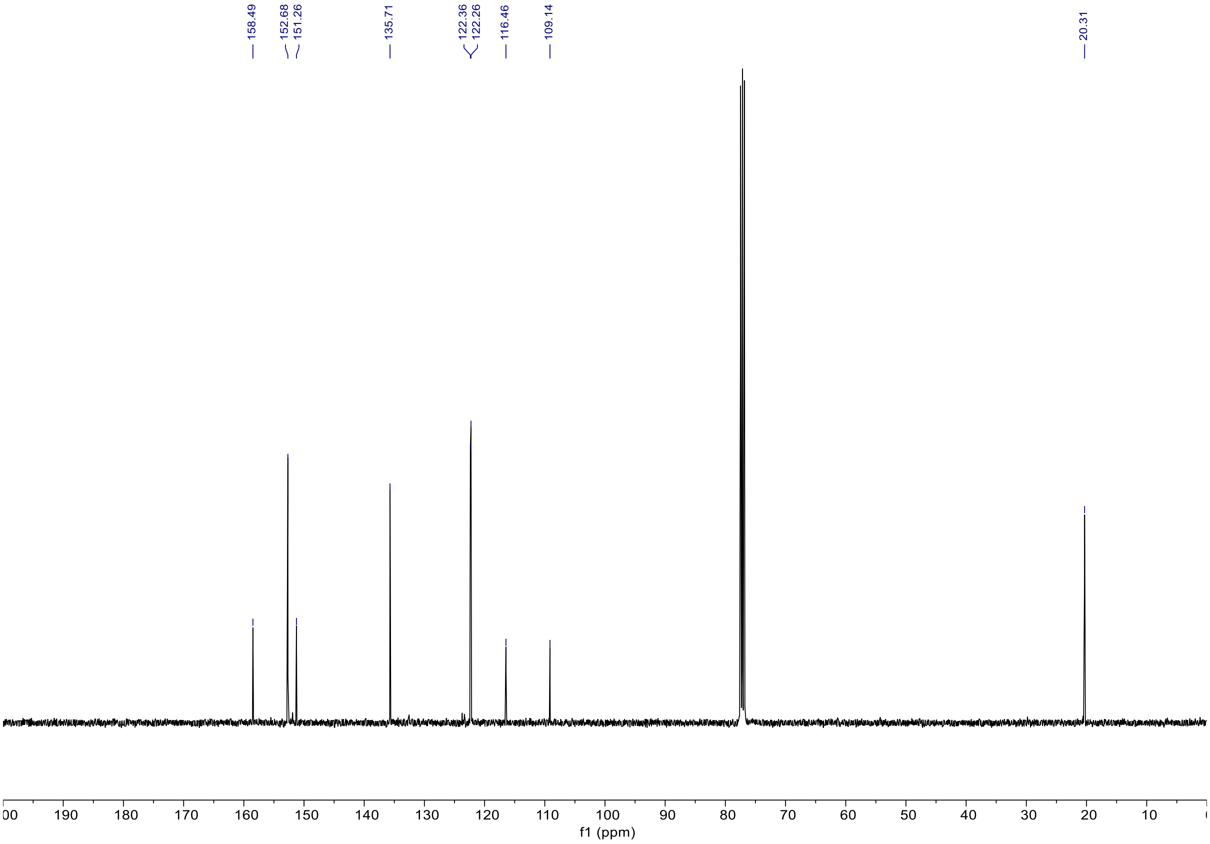

methyl 4-methyl-6-vinylnicotinate (4w).

400 MHz, ^{1}H NMR in Chloroform-*d*

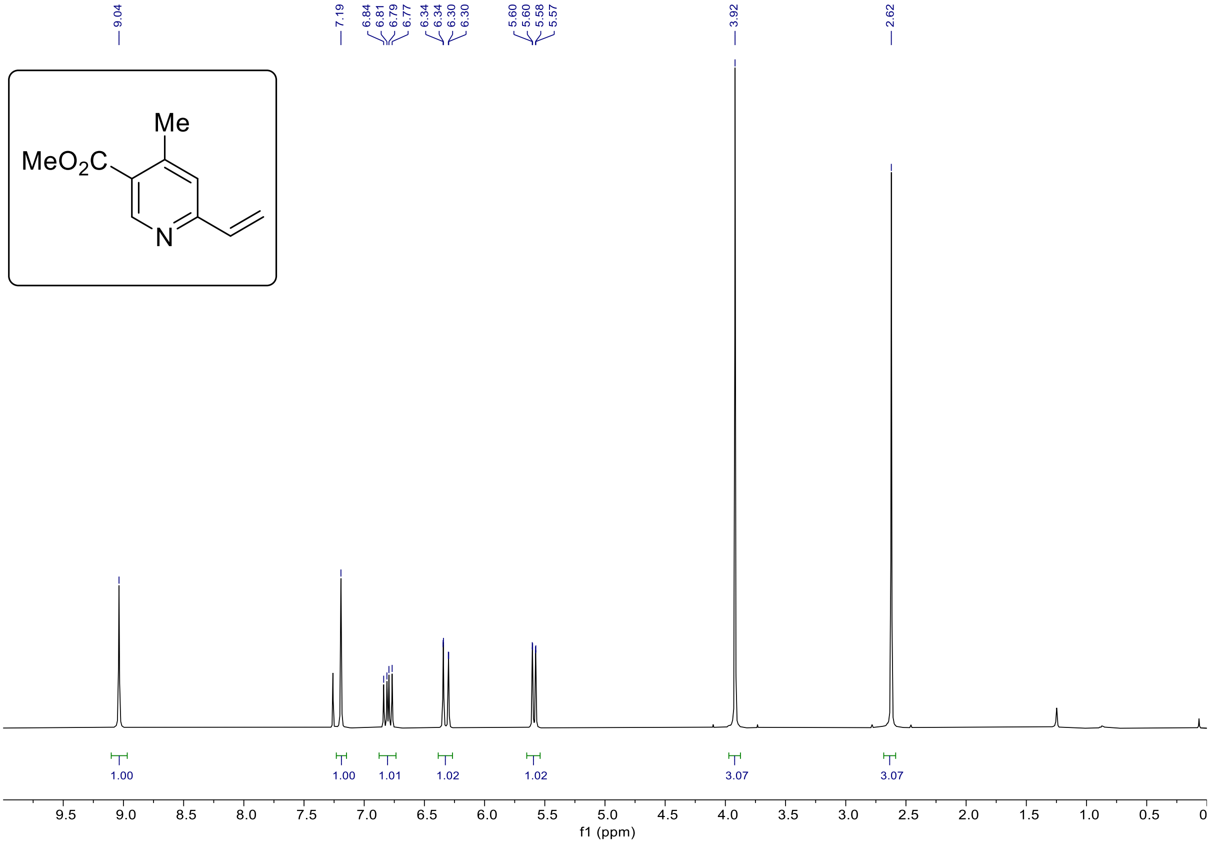

101 MHz, ^{13}C NMR in Chloroform-*d*

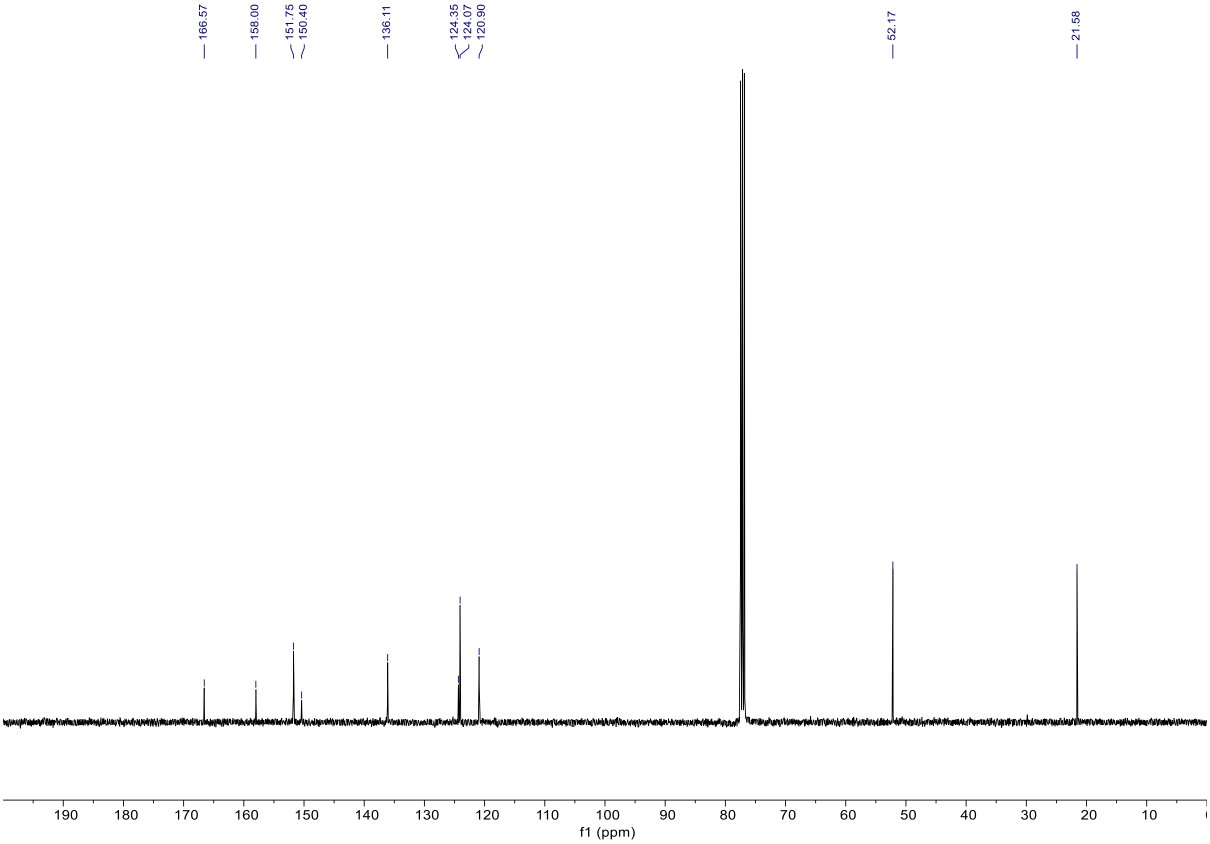

methyl 6-methyl-4-vinylnicotinate (4x-C4).

400 MHz, ^{1}H NMR in Chloroform-*d*

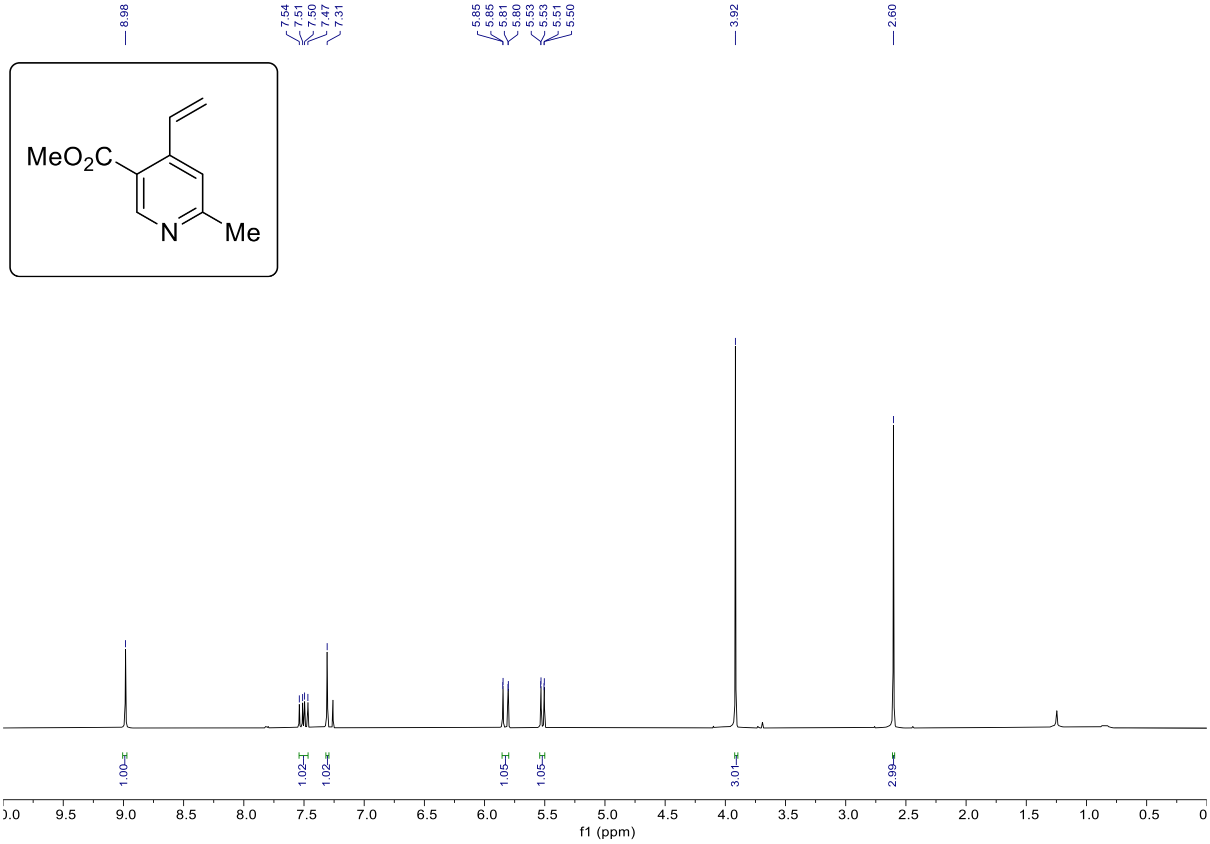

101 MHz, ^{13}C NMR in Chloroform-*d*

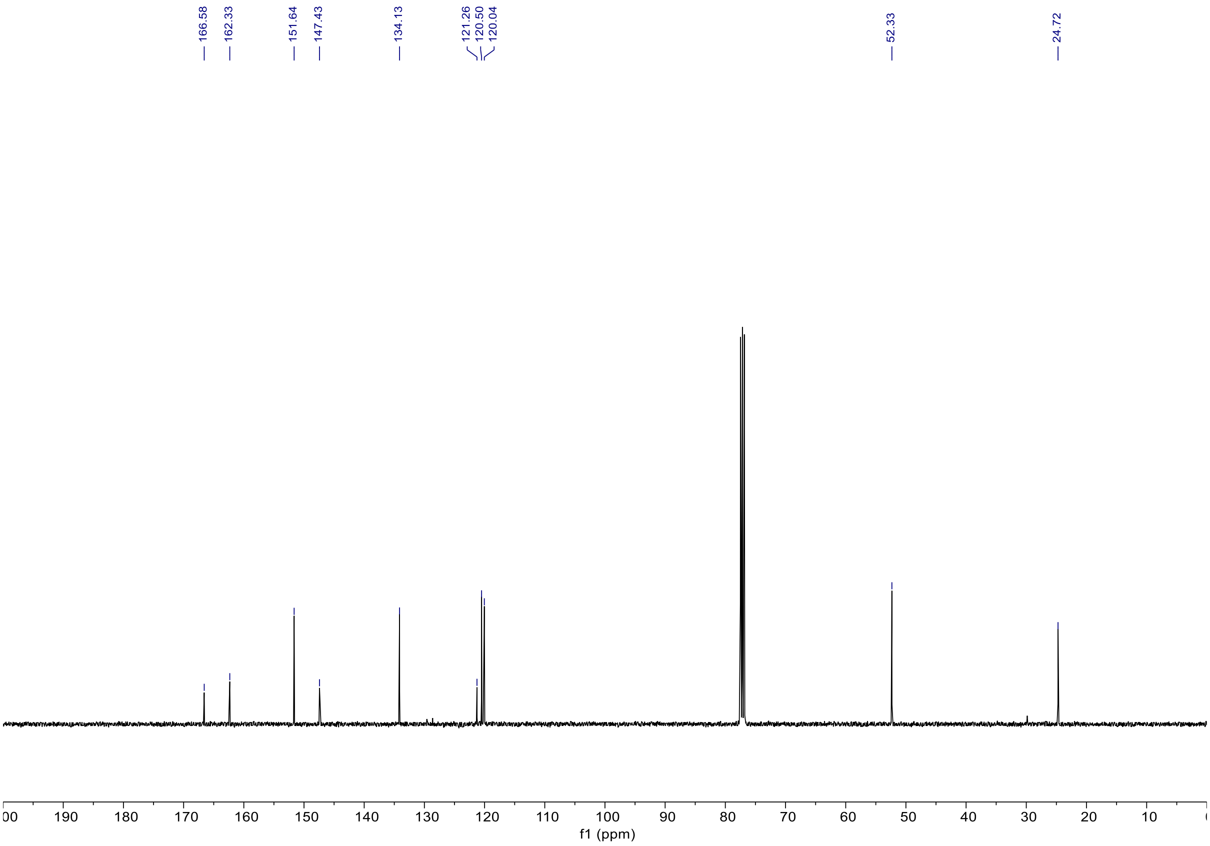

methyl 6-methyl-2-vinylnicotinate (4x-C6).

400 MHz, ^{1}H NMR in Chloroform-*d*

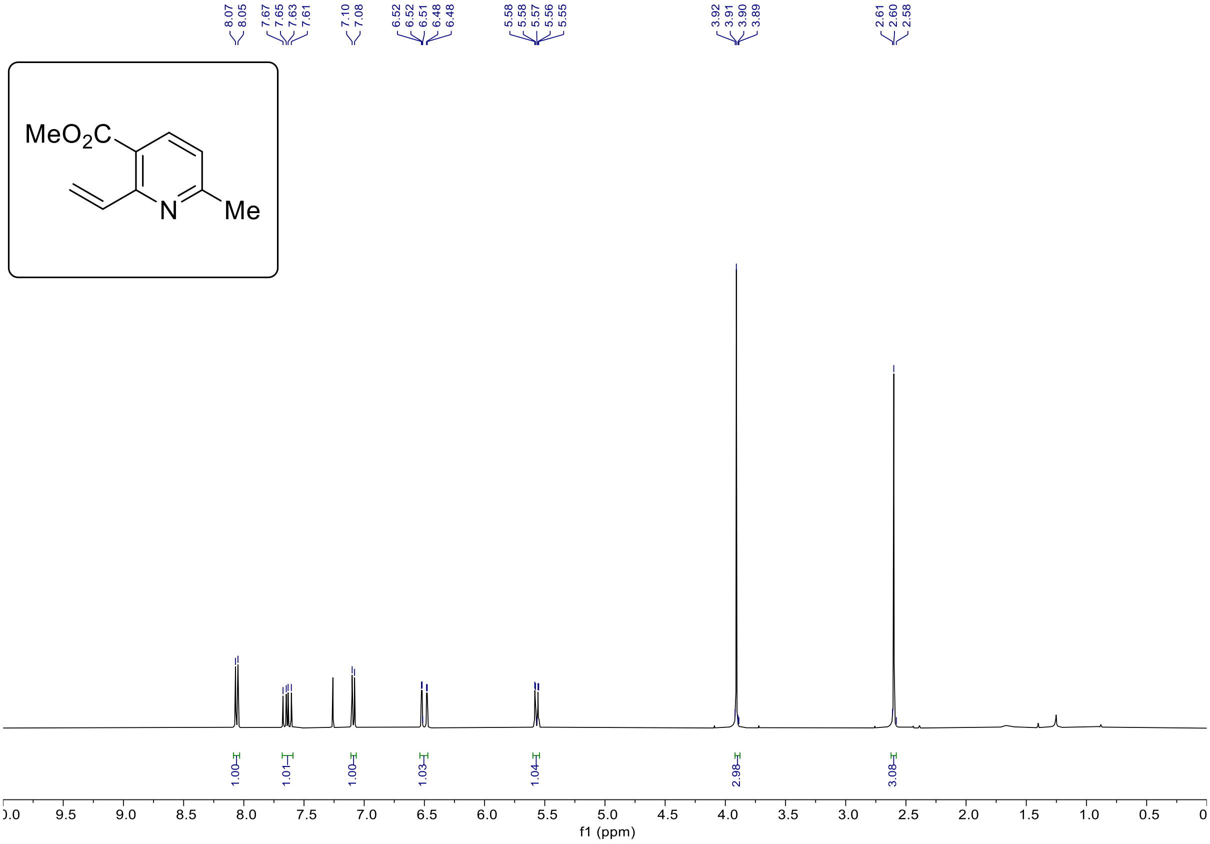

101 MHz, ^{13}C NMR in Chloroform-*d*

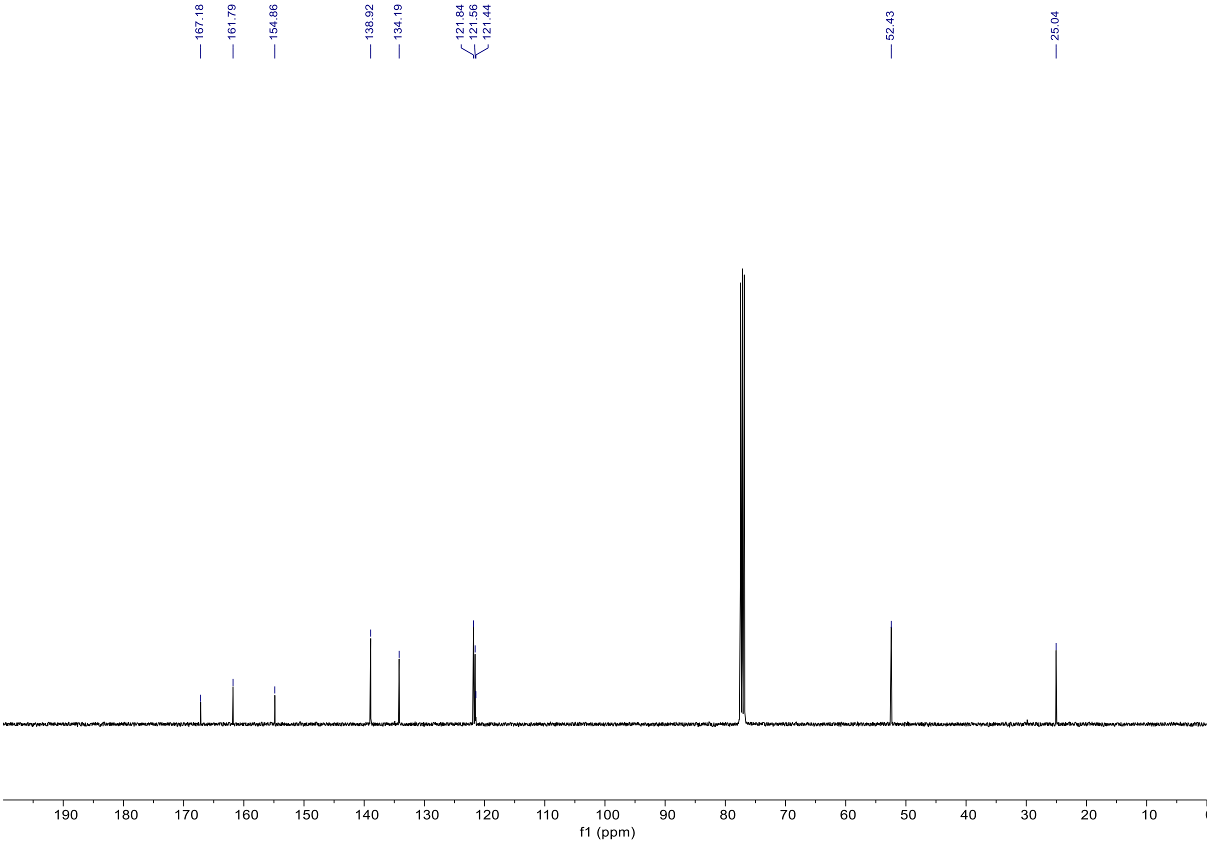

1-(4-((1-vinylisoquinolin-5-yl)sulfonyl)-1,4-diazepan-1-yl)ethan-1-one (4y).

400 MHz, ^{1}H NMR in Chloroform-*d*

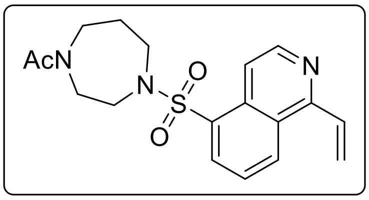

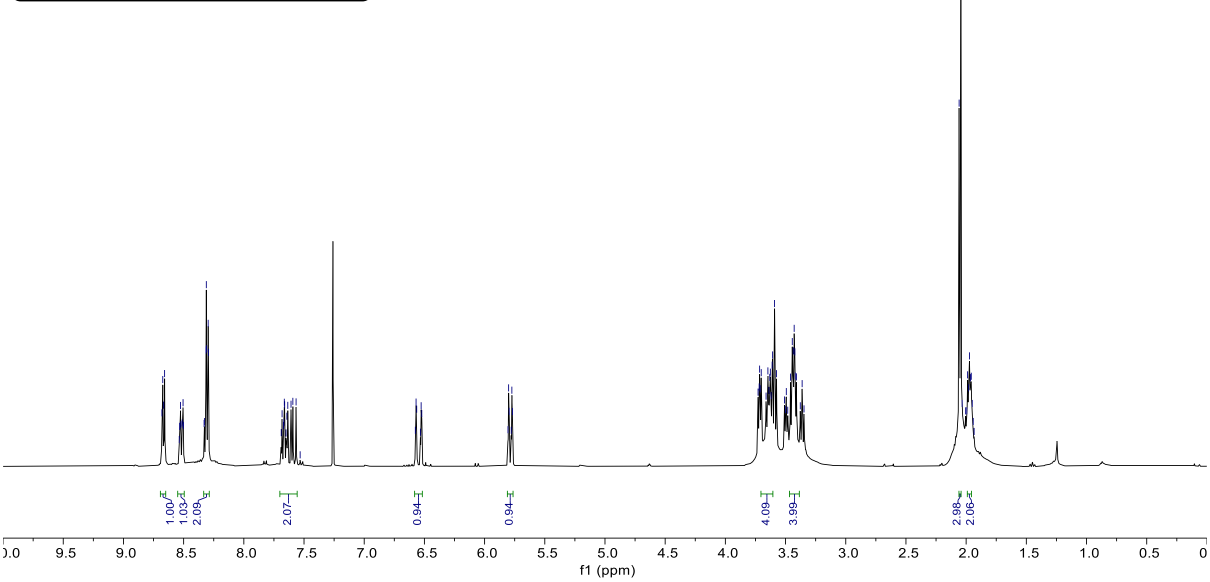

101 MHz, ^{13}C NMR in Chloroform-*d*

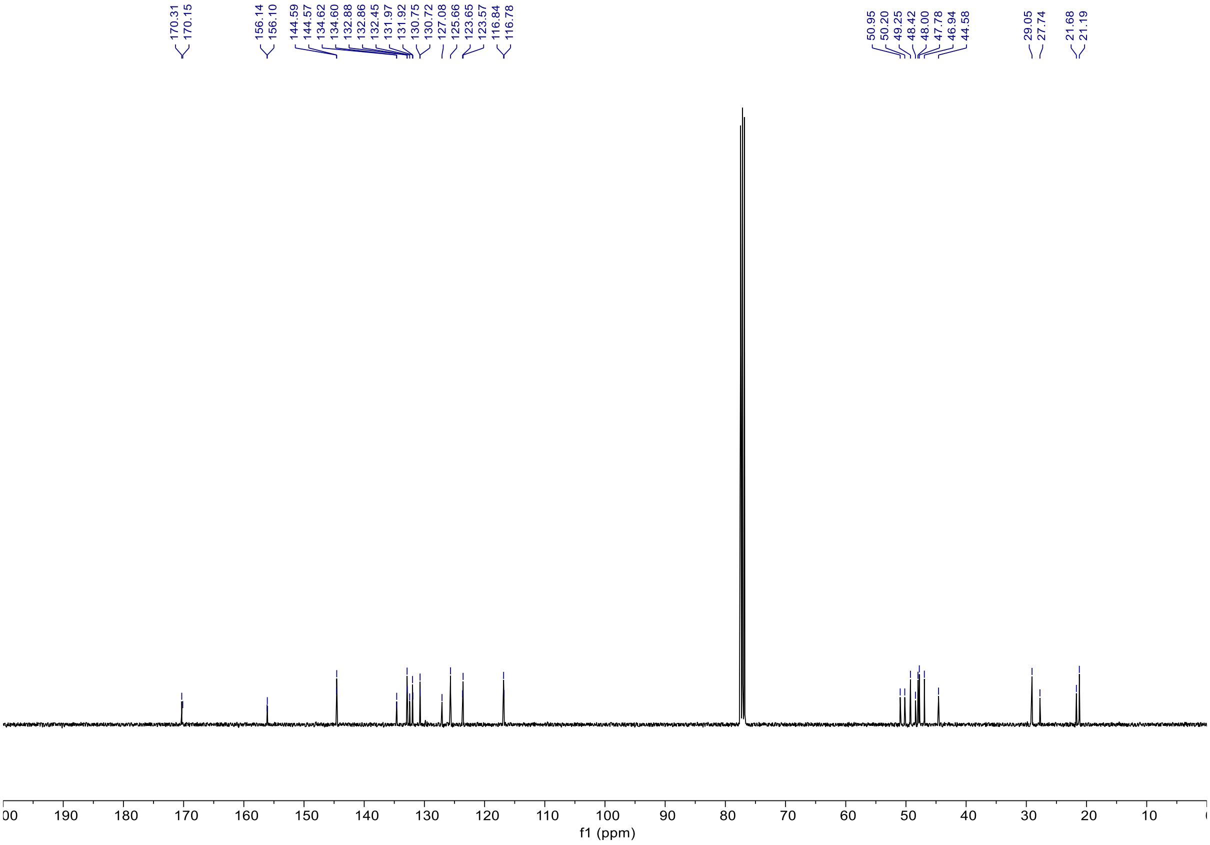

5-(isoquinolin-1-yl)-3-phenyl-4,5-dihydroisoxazole (5a).

400 MHz, ^{1}H NMR in Chloroform-*d*

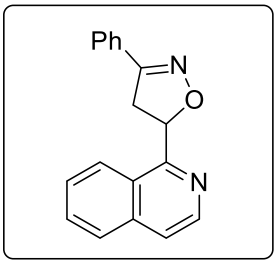

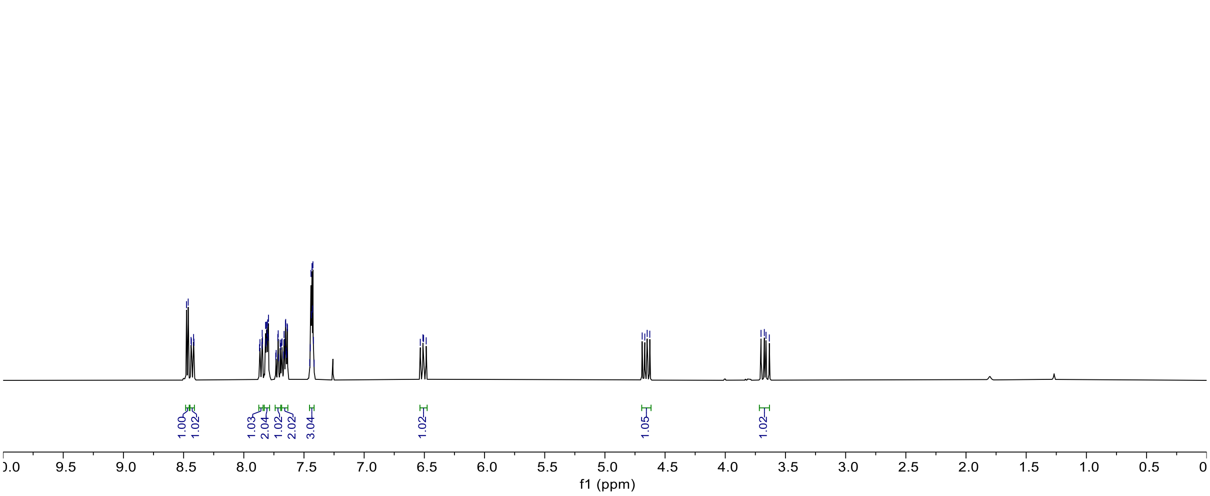

101 MHz, ^{13}C NMR in Chloroform-*d*

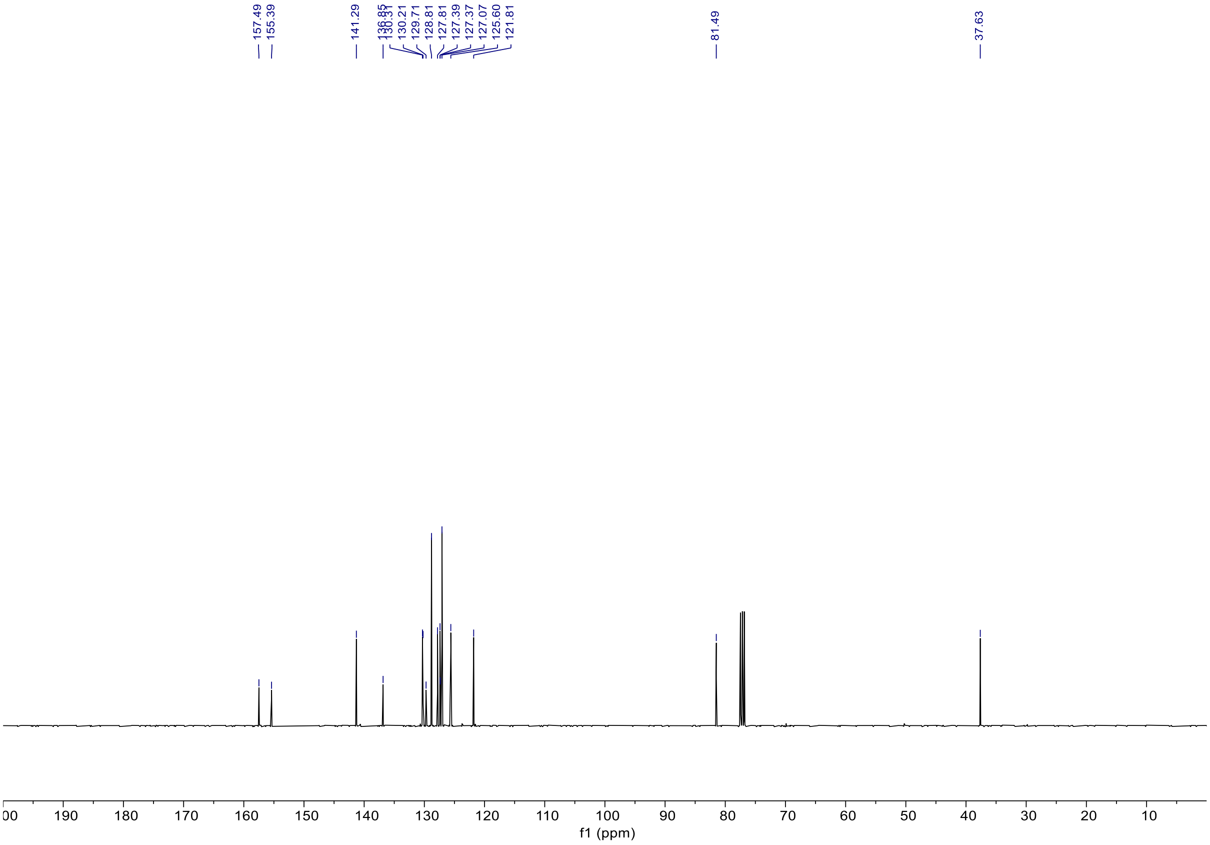

1-(1H-pyrrol-3-yl)isoquinoline (5b).

400 MHz, ^{1}H NMR in Chloroform-*d*

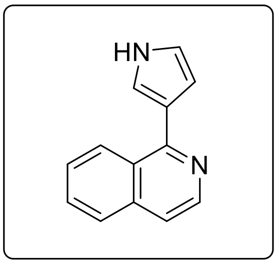

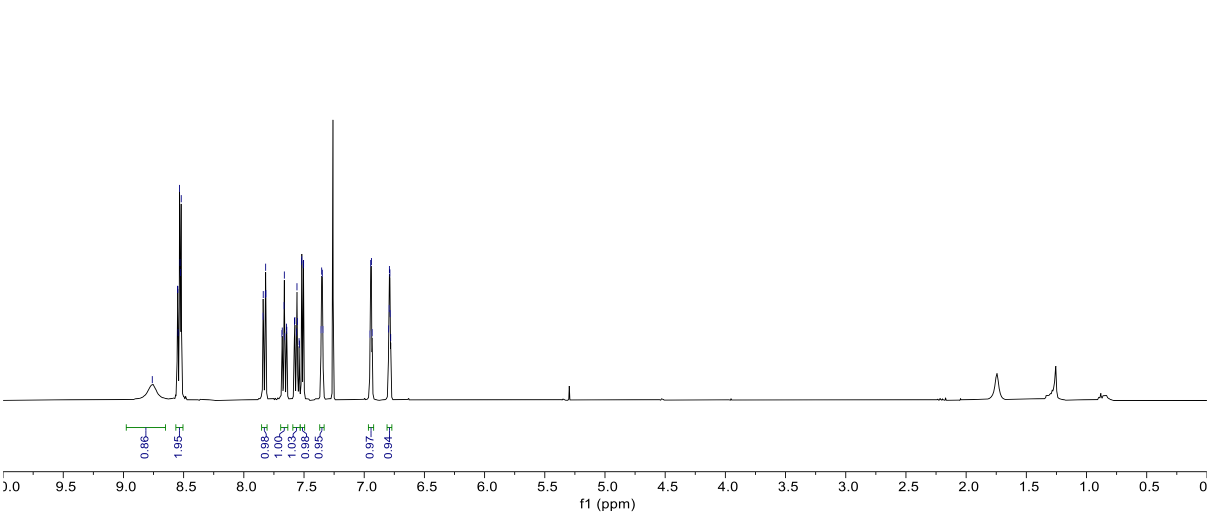

101 MHz, ^{13}C NMR in Chloroform-*d*

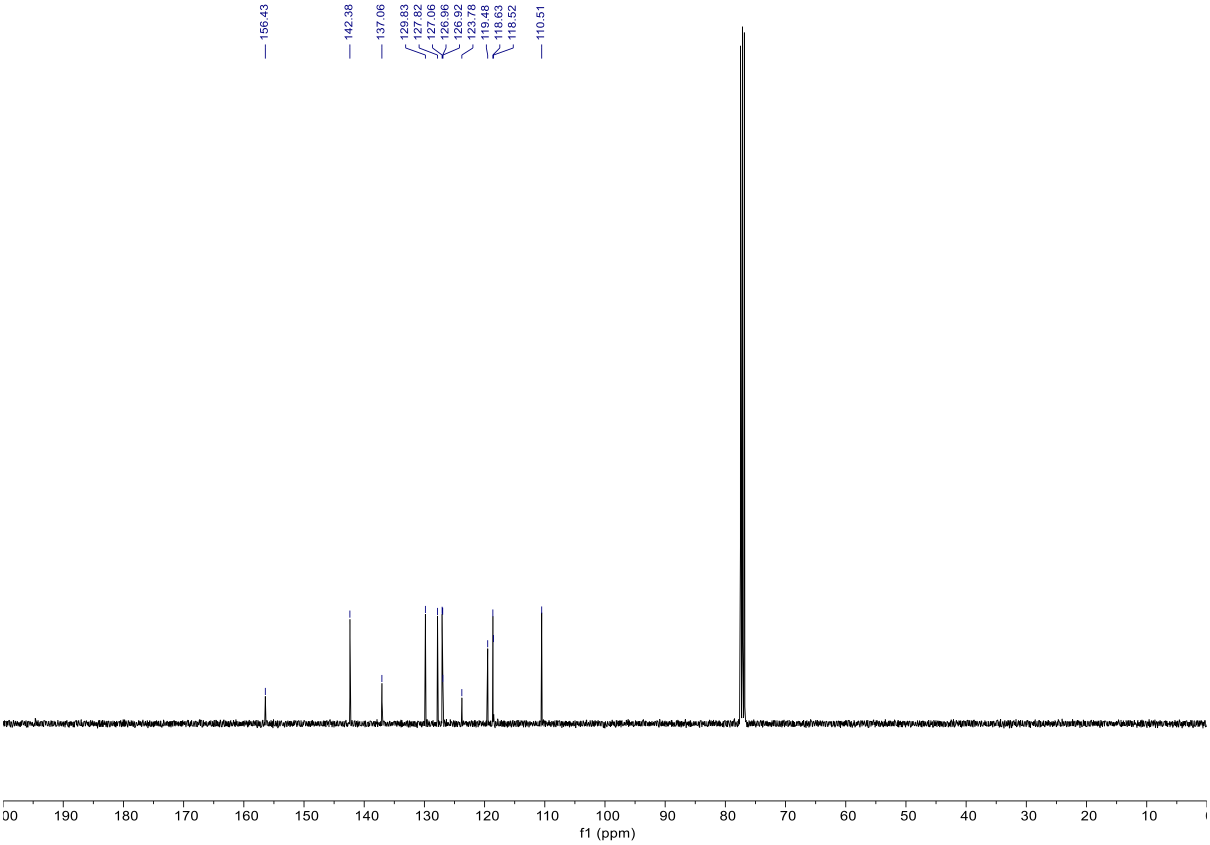

1-(1,4,5,6,7-pentamethylbicyclo[2.2.1]hept-5-en-2-yl)isoquinoline (5c).

600 MHz, ^{1}H NMR in Chloroform-*d*

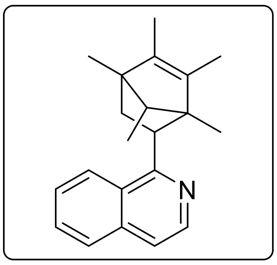

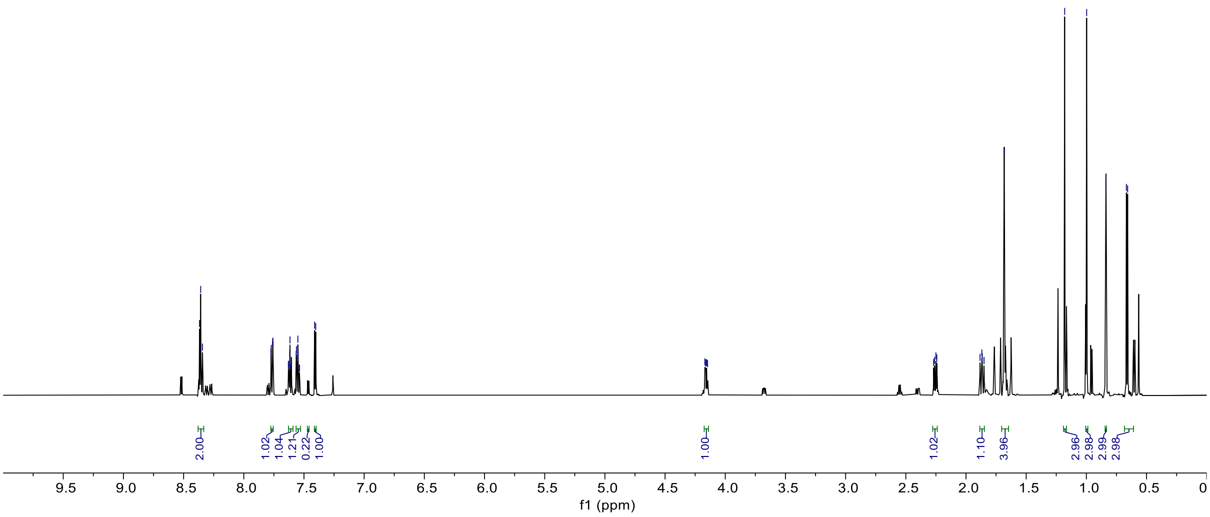

101 MHz, ^{13}C NMR in Chloroform-*d*

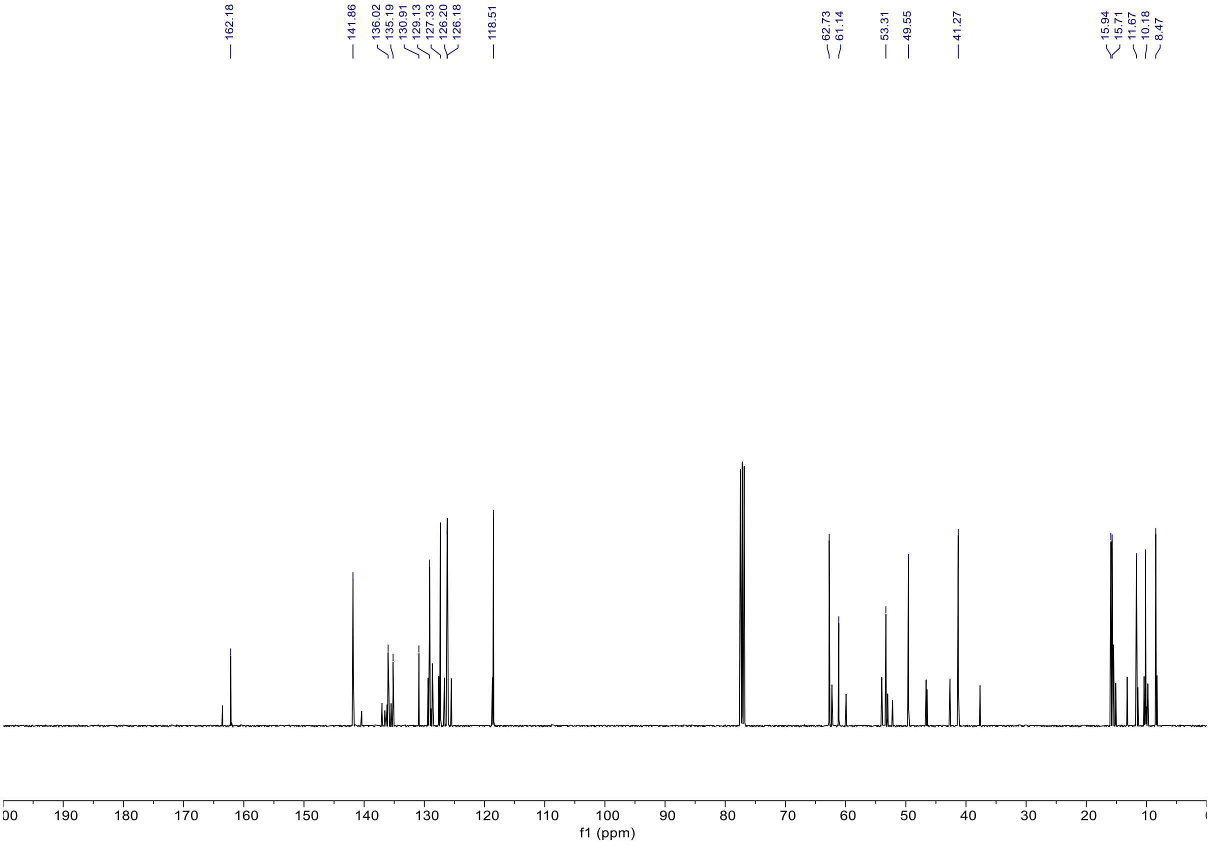

4-(2-(isoquinolin-1-yl)ethyl)morpholine (5d).

400 MHz, ^{1}H NMR in Chloroform-*d*

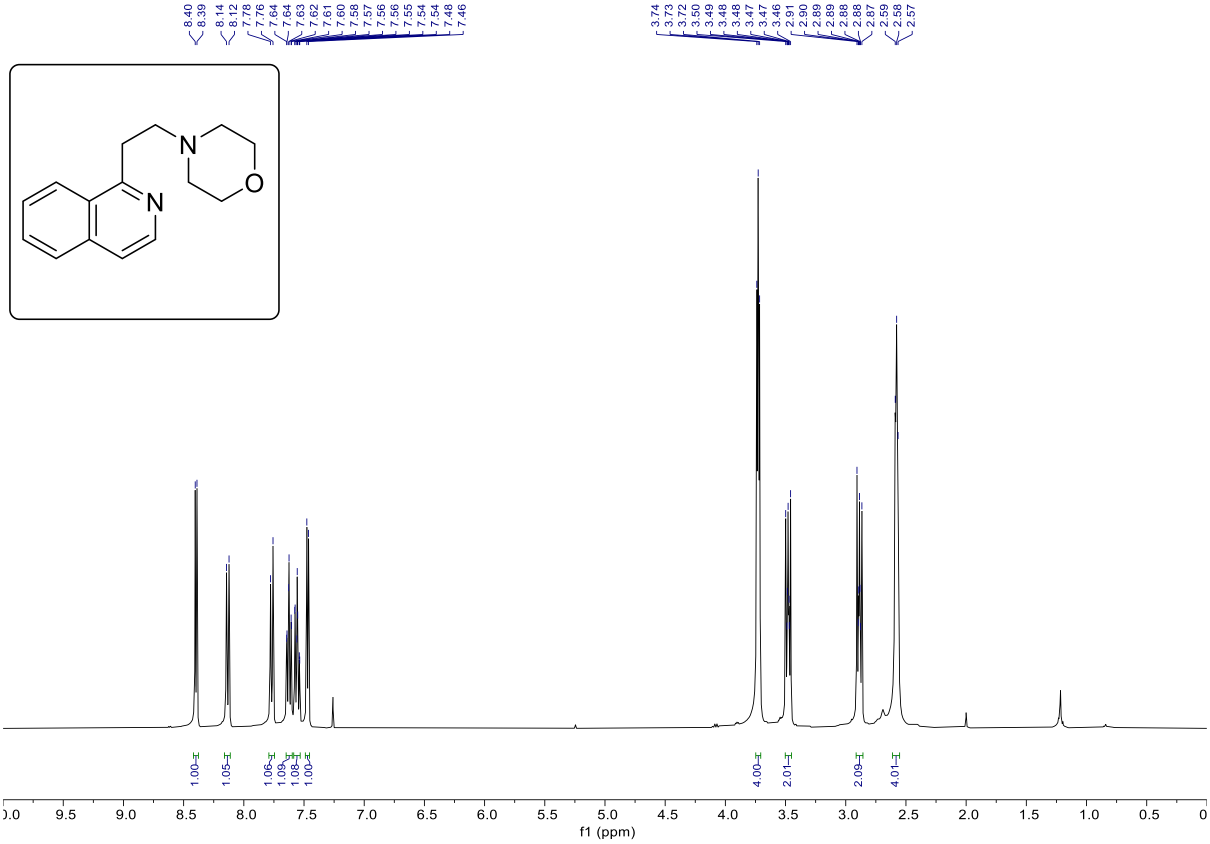

101 MHz, ^{13}C NMR in Chloroform-*d*

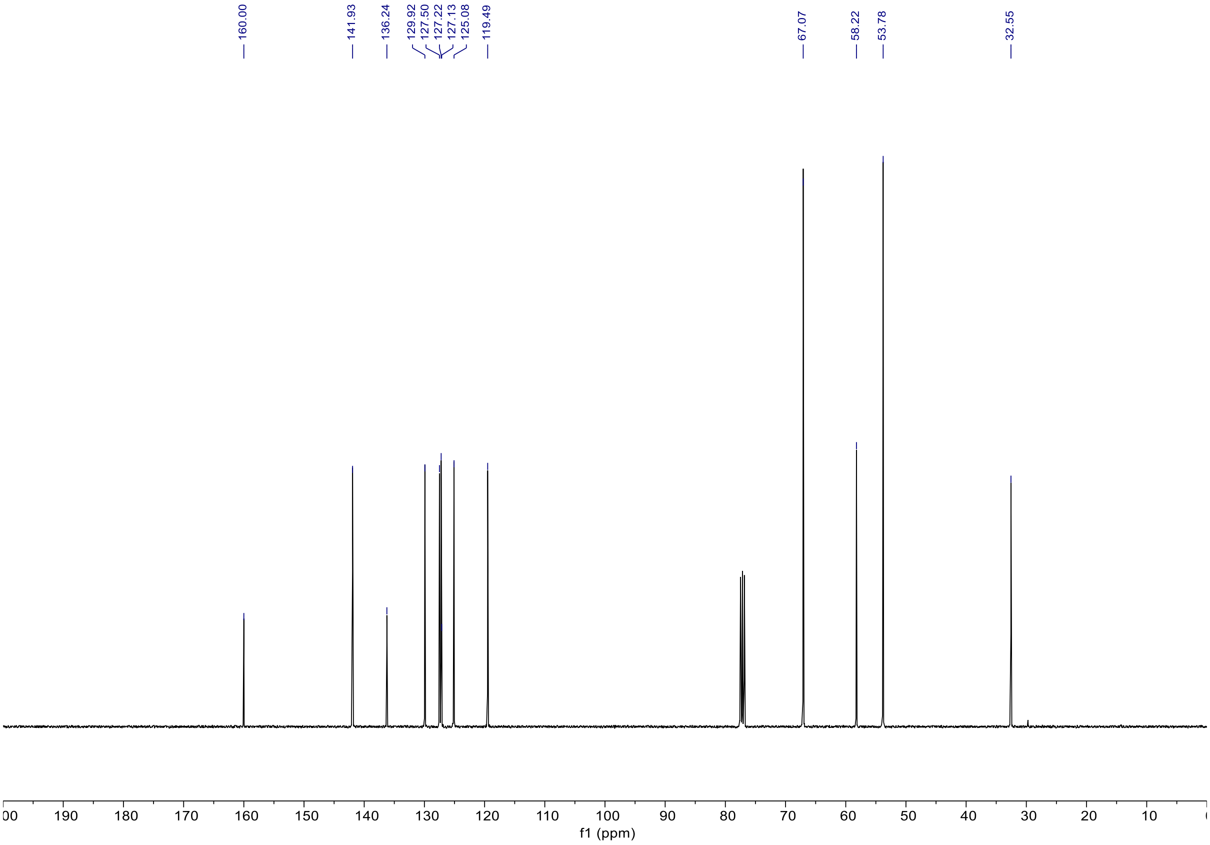

1-(2-(naphthalen-2-yl)-1-tosylazetidin-3-yl)isoquinoline (5e).

600 MHz, ^{1}H NMR in Chloroform-*d*

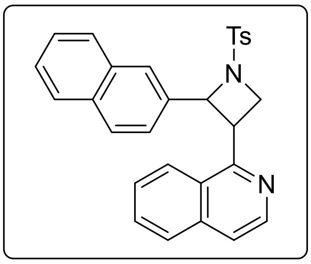

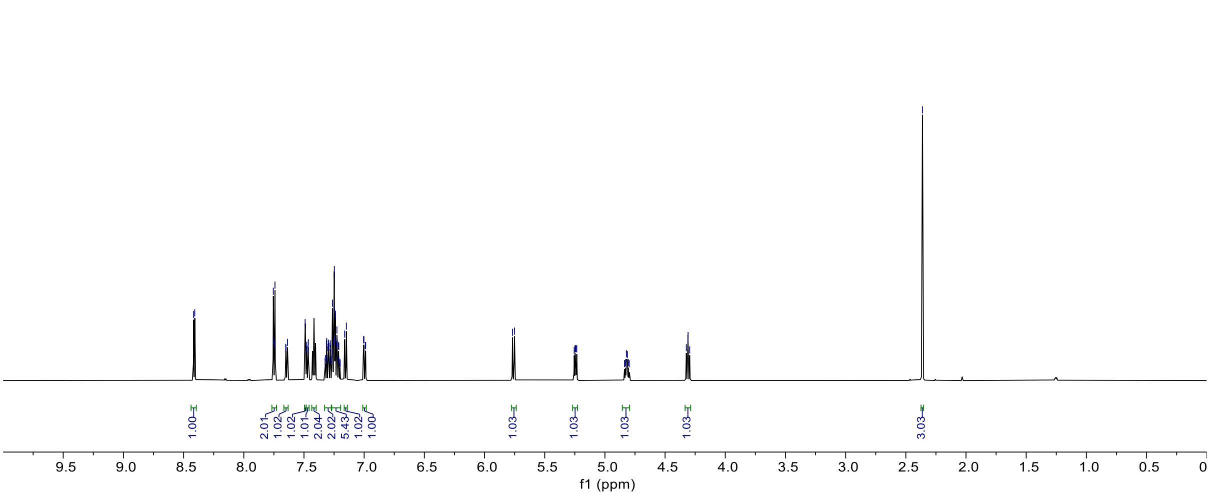

101 MHz, ^{13}C NMR in Chloroform-*d*

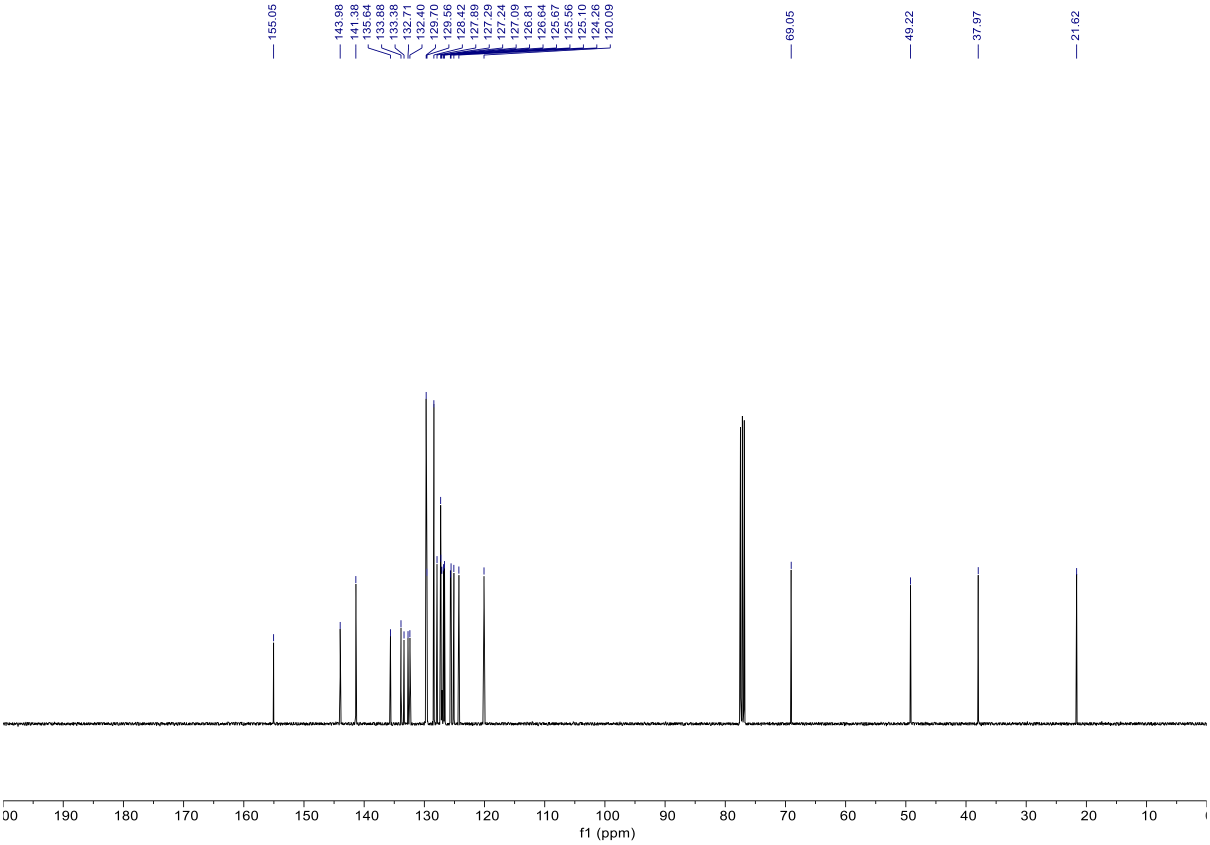

1-(oxiran-2-yl)isoquinoline (5f).

400 MHz, ^{1}H NMR in Chloroform-*d*

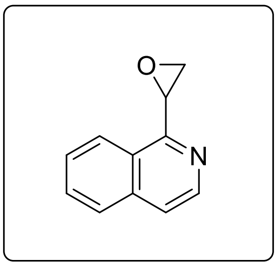

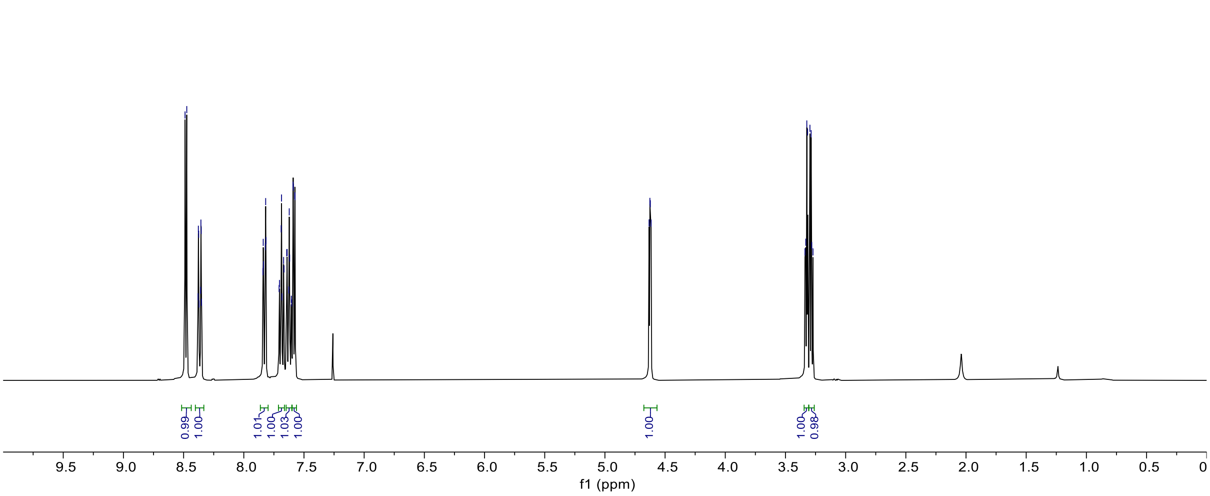

101 MHz, ^{13}C NMR in Chloroform-*d*

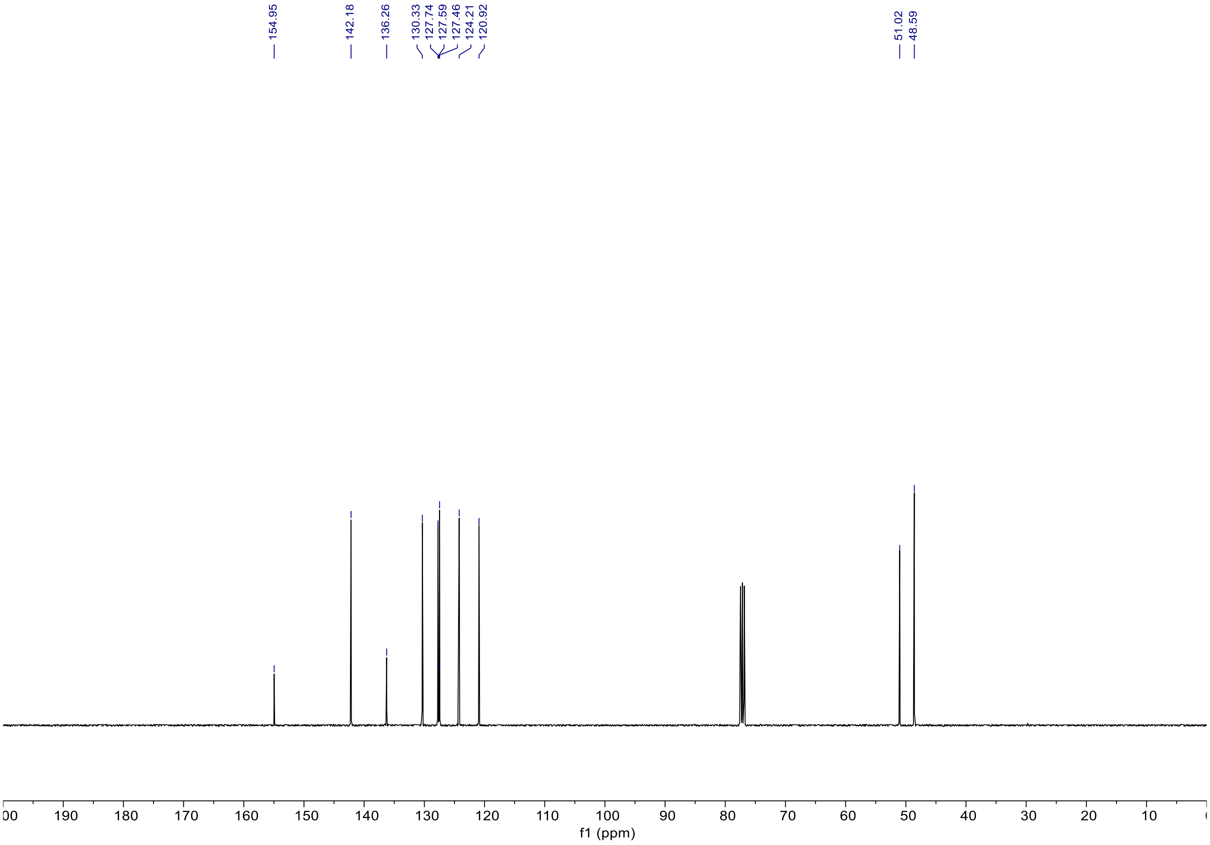

Supplement: Supplementary file 1 — Supporting Information [file ADVS-11-2309800-s001.pdf]
